# Site-selective chlorination of pyrrolic heterocycles by flavin dependent enzyme PrnC

**DOI:** 10.1038/s42004-023-01083-1

**Published:** 2024-01-05

**Authors:** GuangRong Peh, Terence Tay, Lee Ling Tan, Elaine Tiong, Jiawu Bi, Yi Ling Goh, Suming Ye, Fu Lin, Cheryl Jia Xin Tan, Yong Zi Tan, Joel Wong, Huimin Zhao, Fong Tian Wong, Ee Lui Ang, Yee Hwee Lim

**Affiliations:** 1 Institute of Sustainability for Chemicals, Energy and Environment (ISCE2), Agency for Science, Technology and Research (A*STAR), Singapore, Republic of Singapore; 2 Singapore Institute of Food and Biotechnology Innovation (SIFBI), Agency for Science, Technology and Research (A*STAR), Singapore, Republic of Singapore; 3 Institute of Molecular and Cell Biology (IMCB), Agency for Science, Technology and Research (A*STAR), Singapore, Republic of Singapore; 4 Department of Biological Sciences, National University of Singapore, Singapore, Singapore; 5 Disease Intervention Technology Laboratory (DITL), Agency for Science, Technology and Research (A*STAR), Singapore, Republic of Singapore; 6 Department of Chemical and Biomolecular Engineering, Carl R. Woese Institute for Genomic Biology, University of Illinois at Urbana-Champaign, Urbana, IL USA; 7 Synthetic Biology Translational Research Program, Yong Loo Lin School of Medicine, National University of Singapore, Singapore, Republic of Singapore

**Keywords:** Biocatalysis, Biocatalysis, Enzyme mechanisms, Cryoelectron microscopy

## Abstract

Halogenation of pyrrole requires strong electrophilic reagents and often leads to undesired polyhalogenated products. Biocatalytic halogenation is a highly attractive approach given its chemoselectivity and benign reaction conditions. While there are several reports of enzymatic phenol and indole halogenation in organic synthesis, corresponding reports on enzymatic pyrrole halogenation have been lacking. Here we describe the in vitro functional and structural characterization of PrnC, a flavin-dependent halogenase that can act on free-standing pyrroles. Computational modeling and site mutagenesis studies identified three key residues in the catalytic pocket. A moderate resolution map using single-particle cryogenic electron microscopy reveals PrnC to be a dimer. This native PrnC can halogenate a library of structurally diverse pyrrolic heterocycles in a site-selective manner and be applied in the chemoenzymatic synthesis of a chlorinated analog of the agrochemical fungicide Fludioxonil.

## Abstract (web summary)

Pyrroles are an important scaffold in medicinal chemistry with various bioactivities; however, the selective chemical halogenation of pyrroles remains challenging. Here, the authors develop an enzymatic site-selective chlorination of pyrrolic heterocycles by a flavin-dependent halogenase PrnC and apply it to the chemoenzymatic synthesis of a chlorinated analogue of the fungicide Fludioxonil.

## Introduction

**Fig. 1:**
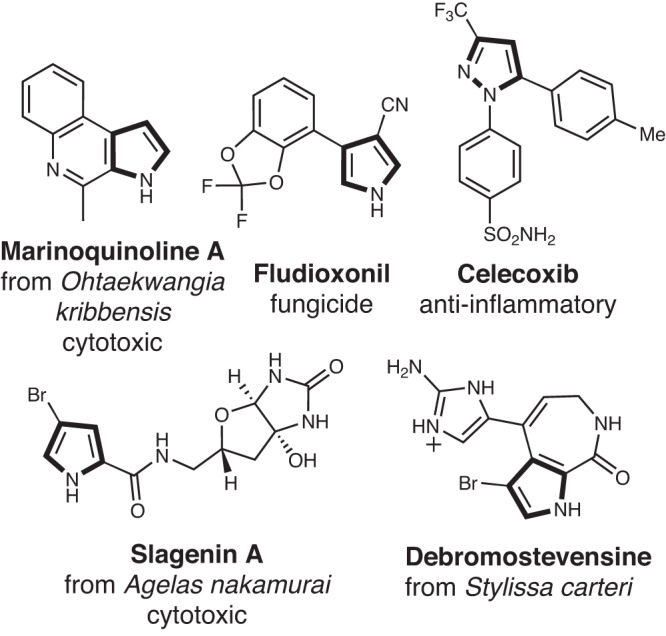
Prevalence of the privilege pyrrolic framework. Pyrrole-based structures are evident in different domains, including Fludioxonil (agrochemical), celecoxib (pharmaceutical), and alkaloids such as slagenin A and debromostevensine (natural products).

**Fig. 2:**
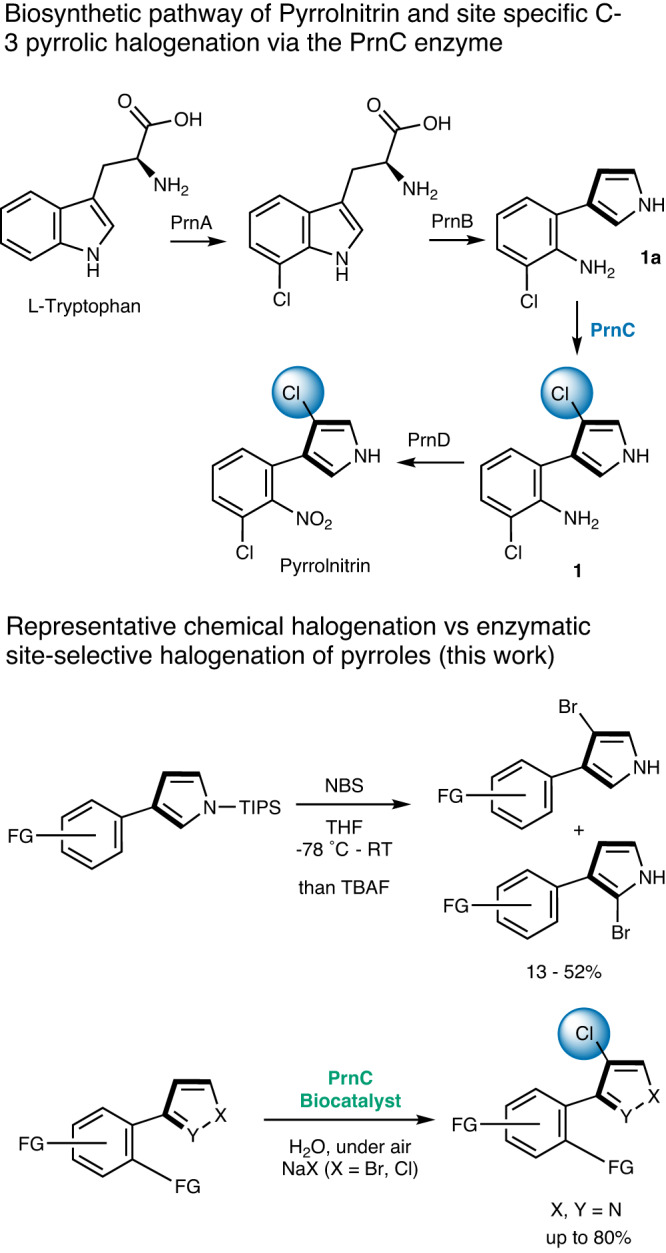
The identification of the PrnC enzyme gene cluster and its application as a biocatalyst for the regioselective halogenation of pyrroles. **Top:** Biosynthetic pathway of Pyrrolnitrin involving PrnABCD. **Bottom:** Comparison between chemical halogenation^63^ and enzymatic halogenation of pyrroles (this work). NBS N-bromosuccinimide, TBAF tetra-n-butylammonium fluoride.

Pyrroles are privileged structures in medicinal chemistry due to the bioactivities (antifungal, antibacterial, and anti-cancer) that are often associated with natural products possessing such scaffold (Fig. 1)^1^. In addition to their use as drugs, pyrrolic compounds have also found applications in agrochemicals^2–7^, in advanced materials^8,9^ (e.g., organic semiconductors, and solar cells), or as organic catalysts^10,11^. The high electron density of the pyrrole ring makes it susceptible to air oxidation when it is not substituted with electron-withdrawing substituents or part of a larger conjugated system^12,13^. As such, chemical halogenation of unsubstituted pyrroles often leads to uncontrolled poly-halogenation. Instead, halogenation of *N*-protected pyrroles with *N*-halosuccinimides^14^ is a common approach though this method is notoriously unreactive, especially with less reactive *N*-chlorosuccinimide. Due to the higher resonance stability of the C2(C5) pyrrolic intermediate carbocation, Hammond’s postulate implies pyrroles are more likely to undergo C2 halogenation (Fig. 2, also see Supplementary Materials and Methods, Section 1.8, Fig. S1). Therefore, direct C3-selective halogenation is even more challenging^15^ in the absence of electrophilic-directing and/or steric hindering groups in the C2 position. In contrast, enzymatic pyrrole halogenation can predominantly yield site-selective mono-halogenation under mild buffer conditions without the need for any protective and/or directing groups^16–19^, and avoids toxic halogenating reagents. The mono-halogenation feature further provides a useful handle for late-stage functionalization through a plethora of metal-catalyzed coupling reactions^20^.

In nature, pyrrolic halogenases belong to the family of flavin-dependent halogenases (FDHs)^21–23^. Most of the well-studied flavin-dependent halogenases to-date focuses broadly on phenolic^24,25^, aryl, or tryptophan-based^26–28^ substrates corresponding to their native substrates. Of the known pyrrole halogenases, there are very limited reported examples of flavin-dependent halogenase acting on free-standing pyrrole substrates^29–32^ (variant A FDHs) while most pyrrole halogenases act on carrier protein-tethered substrates (variant B FDHs)^33–37^. Consequently, PrnC is an attractive enzyme for investigation because it can halogenate an unconfined pyrrole substrate with the potential to be applied in a flexible manner to other enzymatic or chemical transformations.

The *prnC* gene encoding PrnC is part of the *prnABCD* gene cluster responsible for the biosynthesis of a natural product, pyrrolnitrin (Fig. 2). Pyrrolnitrin was first reported by Arima et al.^38^ in 1964. Its biosynthesis from l-tryptophan was proposed by Lively^39^ and others^40^, and finally confirmed by Van Pée and co-workers^41^ through isotopic labeling using the *Pseudomonas fluorescens* strain BL915. In the proposed mechanism, the two chlorine atoms in **1** are introduced sequentially by the tryptophan halogenase PrnA, and the pyrrole halogenase PrnC that is hypothesized to regioselectively chlorinate the C3 position of the pyrrolic backbone. While PrnA is well studied^42^, there are no reports of characterization and application of PrnC to date. Here, we describe the first characterization of PrnC’s activity and specificity towards structurally diverse pyrrolic heterocycles, expanding our understanding of pyrrolic halogenases. Effective protein engineering facilitated the establishment of a robust heterologous expression of PrnC, enabling us to conduct comprehensive in vitro characterization, including cryo-electron microscopy (cryo-EM) to visualize the apo-PrnC which revealed indications of a dimeric arrangement. A chemoenzymatic synthesis of a chlorinated analog of the agrochemical fungicide, Fludioxonil, was also demonstrated.

## Results and discussion

Our early attempts to express PrnC heterologously in *Escherichia coli* (*E. coli*) yielded largely insoluble proteins with yields generally <1 mg/L. Initial optimization by co-expression with chaperone proteins such as GroES-GroEL (encoded in the pGro7 plasmid) only led to marginal improvements to the yields. Subsequent extensive optimization of the expression construct along with the addition of a recently described *N*-terminal (11 amino acids) solubility tag^43^ finally enabled consistent production ( ~ 9 mg/L of culture, see Supplementary Materials and Methods, section 1.8.1, Fig. S2a, b) of soluble functional PrnC for in vitro functional assay. As the soluble protein yield increased, we also observed a slight enhancement in activity, which could be attributed to improved enzyme folding (see Supplementary Materials and Methods, section 1.8.2, Fig. S3a).

### Reaction development

To determine the optimal conditions to characterize PrnC against a library of substrates, we investigated the impact of various co-factor combinations (Table 1). Our initial assay using the native substrate monodechloroaminopyrrolnitrin (**1a**) with purified PrnC, along with the co-factors FAD (50 mol% relative to PrnC), NADH (5.0 equiv) and *E. coli* flavin reductase (Fre, 0.5 mol%) at 30 °C for 18 h gave only 9% conversion to aminopyrrolnitrin (**1**) (entry 1). Introduction of the glucose dehydrogenase (GdHi)-NADH regeneration system with the intent to improve the turnover of the enzyme resulted in an appreciable improvement (33% conversion), suggesting that other parameters should be evaluated (entry 2). Interestingly, a ten-fold decrease in FAD concentration led to a substantial increase in conversion by 2.5-fold to 81% (entry 3). We rationalize that the lower FAD concentration could help in the following two ways: (1) to modulate the rate of generation of the reactive hypohalous acid intermediate for the site-selective halogenation, thus preventing large accumulation of highly reactive intermediates in the active site or HOCl leakage^44–46^, (2) to modulate the rate of formation of PrnC:FADH^-^ complex reducing the likelihood of enzyme inactivation^47^. Further investigation was conducted to determine whether there is a fine balance between the co-factors’ ratio for optimal mono-chlorination conversion. Indeed, doubling the Fre concentration (2×) to increase FADH_2_ turnover led to a slight drop in mono-chlorination conversion (entry 4) along with small amount of di-chlorinated product. Adding sub-stoichiometric amounts of NADH (0.05 equiv) (entry 5) does not adversely affect the conversion process, demonstrating efficient coupling of the (GdHi)-NADH regeneration system with other cofactors. Interestingly, doubling the loading of PrnC (entry 6) with the intention to limit non-specific HOCl within the substrate binding site resulted in higher production of di-chlorinated products. While the mechanism of the second halogenation event^48^ is unclear at this moment, this observation along with other control experiments (see Supplementary Materials and Methods, section 1.8.2, Fig. S3c–d) suggest that the second halogenation is also facilitated by catalytic residues within the enzyme active site. The second halogenation event appears to be less energetically favorable, likely due to the reduced electron density on the pyrrolic ring after the initial halogenation. To ascertain that the halogenation event was not caused by the formation of HOCl in futile cycles, another control experiment (entry 7) where co-factor catalase was introduced to the best conditions (entry 3) was conducted (also see Supplementary Materials and Methods, section 1.8.2, Fig. S3e, f). The similar conversion results (85%) to the conditions without catalase addition suggest that halogenation is indeed catalyzed by the enzyme PrnC. Removal of the glucose dehydrogenase (GdHi)-NADH regeneration system led to worse outcome, suggesting there is a tight kinetic balance between the PrnC enzyme and all its co-factors. Expectedly, the native PrnC enzyme has the highest preference for chloride followed by bromide. It does not accept iodide (see Supplementary Materials and Methods, section 1.8.2, Fig. S3g).

**Table 1:** Optimization study of PrnC biocatalyst on the native substrate **1a**.


Entry	Variation from standard conditions						Conversion of 1^a^(%)
	FAD (mol%)	GdHi (mol%)	Glucose (Equiv)	Fre (mol%)	NADH (Equiv)	PrnC (mol%)	
1.	2.0 (10×)	**✖**	**✖**	✓	✓	✓	9
2.	2.0 (10×)	✓	✓	✓	✓	✓	33
3.	✓	✓	✓	✓	✓	✓	81^b,c^
4.	✓	✓	✓	1.0 (2×)	✓	✓	77^d^
5.	✓	✓	✓	✓	0.05 (Less 100×)	✓	77
6.	✓	✓	✓	✓	0.05 (Less 100×)	8.0 (2×)	74^e^
7.^f^	✓	✓	✓	✓	✓	✓	85^c^

^a^Conversion is calculated based on calibration curves of mono-chlorinated product (**1**) and starting material (**1a**)(Calibration curve in Supplementary Materials and Methods, section 1.8.2, Fig. S3b).

^b^Standard conditions: **1a** (0.5 mM), PrnC Biocatalyst (3 mol%), GdHi (0.5 mol%), FAD (0.2 mol%), Fre (0.5 mol%), NADH (5.0 equiv), Glucose (10.0 equiv), MgCl_2_ (20.0 equiv), phosphate buffer (10 mM, pH 7.4).

^c^≤5% di-chlorinated product.

^d^*~*8% di-chlorinated product.

^e^~12% di-chlorinated product.

^f^Catalase (70 U/mL) added. Conversion of di-chlorinated product calculated based on relative HPLC trace between mono and di-chlorinated product peaks at 254 nm. Symbols: ✓ (tick) = as per standard conditions, **✖** (cross) = reagent/co-factor not used. FAD = flavin adenine dinucleotide, GdHi = glucose dehydrogenase, Fre = flavin reductase, NADH = nicotinamide adenine dinucleotide (reduced form).

To explore the sequence space around PrnC, a sequence similarity network was generated, which led to the identification of four other homologs with conserved active site residues and genomic neighborhoods of PrnAB (see Supplementary Materials and Methods, section 1.8.2, Fig. S4a–c). Similar to PrnC, these homologs exhibited solubility issues when heterologously expressed and optimized in *E. coli* hosts. Despite these challenges, we were able to obtain soluble expression of the homologs, and they were subsequently characterized against the native PrnC (see Supplementary Materials and Methods, section 1.8.2, Fig. S4d, e). Through these investigations, native PrnC was identified as the most effective halogenase, and therefore was utilized for the remainder of the study.

Native PrnC was found to have a relatively low *k*_cat_ of 0.46 min^−1^ against its native substrate **1a** (Table S1). This is largely similar to other wild-type tryptophan-based FDHs such as RebH and PyrH (Table S1). PrnC also exhibits a moderate affinity for **1a**, with *K*_m_ = 15.8 ± 0.7 μM. The catalytic efficiency of this native PrnC enzyme (*K*_cat_/*K*_m_) was found to be modest at 0.029 ± 0.002 μM^−1^ min^−1^ (see Supplementary Materials and Methods, section 1.8, Table S1 and section 1.8.2, Fig. S5).

### Molecular docking and Cryo-EM analysis

**Fig. 3:**
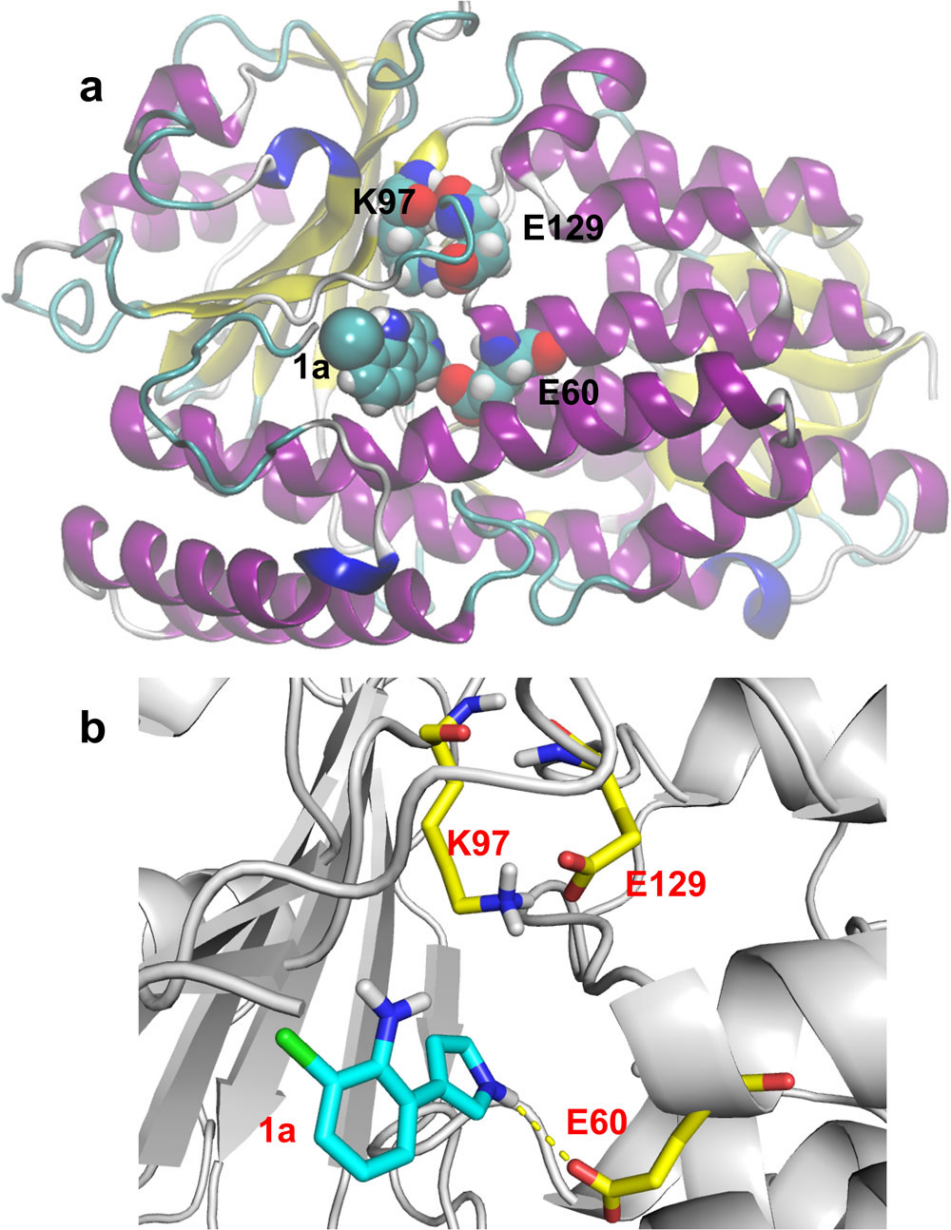
The homology model of PrnC reveals the catalytic triad accountable for its site-specific halogenation function. **a** The optimized homology model for PrnC is based on the template crystal structure of halogenase PltM (PDB code: 6BZA). **b** The substrate binding site of the PrnC-**1a** complex from molecular modeling and docking. K97, E129, and E60 are proposed to be the key residues.

To determine the residues that are essential for the catalytic reaction of the PrnC enzyme, a systematic computational modeling of the enzyme was performed and a three-dimensional model for the PrnC/substrate complex (see method: homology and docking models of PrnC) was achieved. A PrnC/substrate complex model (Fig. 3) indicates that K97 is close to **1a** and may initiate catalysis either directly by reacting with HOCl to form a chloramine intermediate^49^ or indirectly by stabilizing the chloroamine intermediate through hydrogen bonds^50^; E129 is also postulated to form a salt bridge with K97 to stabilize its sidechain conformation and E60 may also form a hydrogen bond with **1a**. To validate the computationally proposed key residues, the site-directed mutagenesis of all three residues was conducted, and its results are shown in Fig. S6 (see Supplementary Materials and Methods, section 1.8.2). Mutation of either residue (K97, E60, or E129) to alanine abolished or significantly reduced the enzyme activity, indicating that these residues are important for catalysis, as suggested by the above hypothesis. Among, mutation of K97 resides also led to a significant drop in protein expression yield.

**Fig. 4:**
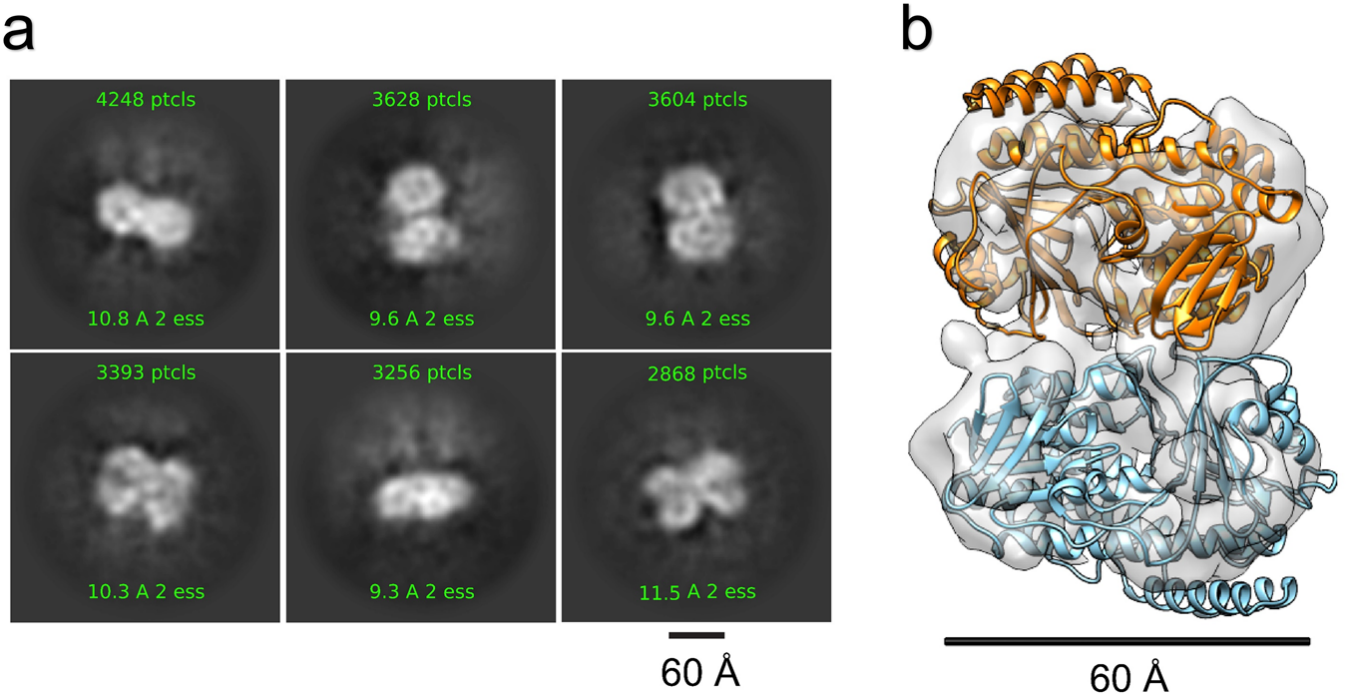
Cryo-electron microscopy for the empirical structural elucidation of PrnC. Single-particle cryo-EM analysis of PrnC reveals that the protein exists as dimers in solution, as seen from both **a** the 2D class averages and **b** docking of the homology model into the 3D reconstruction.

At the outset of our study (2020), we employed a closely related crystal structure of a FAD-dependent halogenase, PltM, for homology modeling, albeit with only ~30% sequence identity with PrnC (see Supplementary Materials and Methods, section 1.8.2, Fig. S7a, b). Subsequently, a more closely resembling apo structure of a halogenase (CtCP) has been reported^51^. Notably, this apo-CtCP structure exhibits significant structural resemblance to the initial PItm crystal structure employed in our modeling and optimization study. Consequently, the prediction utilizing PItM retains its validity. Both of these closely related halogenases, CtcP and PltM adopt a dimeric crystalline structure. The conserved dimeric interface observed in these structures also corroborates with the predicted structures generated by AlphaFold-Multimer (see Supplementary Materials and Methods, section 1.8.2, Fig. S7c). We also employed single-particle cryogenic electron microscopy (cryo-EM^52^) to determine the experimental structure of PrnC. When the apo-structure of PrnC was imaged, 2D class averages resembling dimers were observed (Fig. 4a). Based on the overall medium resolution 3D reconstruction obtained at around 9 Å, two copies of PrnC can be fitted into the density map when it is docked with the model (Fig. 4b). Several prior examples^49,50,53–55^ have suggested that tryptophan based flavin-dependent halogenases exist as homodimers in crystalline state and as monomers in solution. Examination of chemically cross-linked NT11-PrnC in solution also suggests that PrnC exists as multi-mers (see Supplementary Materials and Methods, section 1.8.2, Fig. S8). However, we were not able to unambiguously assign the dimer interfaces at this resolution and thus, it cannot be ascertained at this stage whether this dimeric form of PrnC is critical for function.

### Substrate scope

**Fig. 5:**
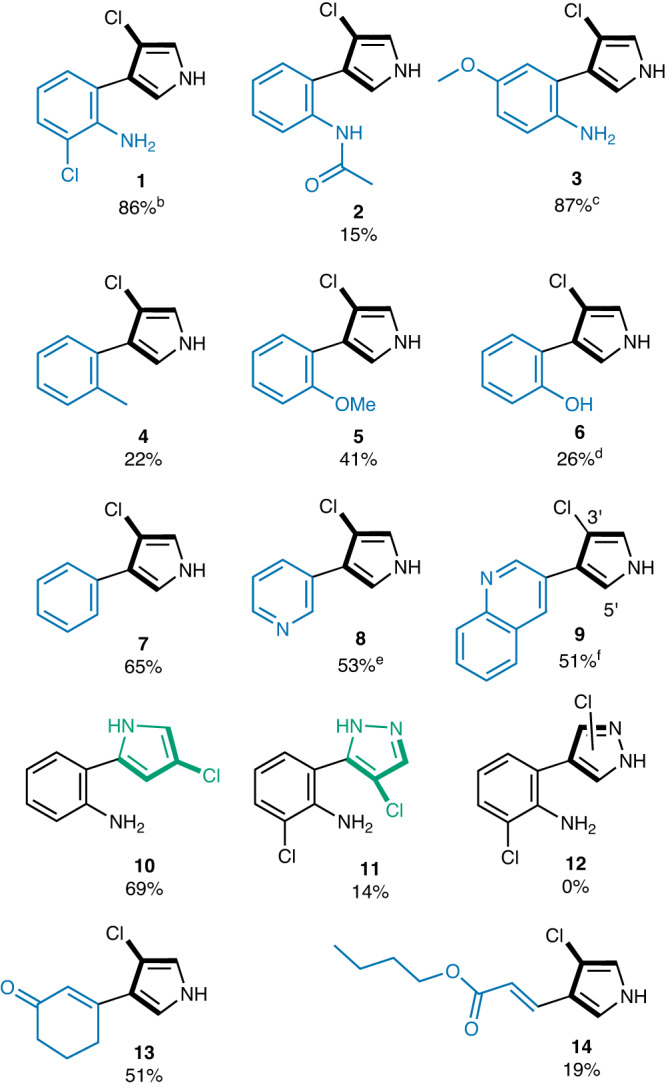
Scope of PrnC mediated in vitro chlorination^a^. [a] Conditions:Substrate (0.5 mM), PrnC Biocatalyst (4 mol%), GdHi (0.5 mol%), FAD (0.2 mol%), Fre (0.5 mol%), NADH (5.0 equiv), Glucose (10.0 equiv), MgCl_2_ (20.0 equiv), phosphate buffer (10 mM, pH 7.4). The conversion (%) represents the area under the peak for desired product and remaining starting material (SM) relative to the area of a control SM standard. Average of duplicate measurements determined by LC/MS from UV absorbance at 254 nm. [b] Based on product standard calibration curve. [c] Mono-Chlorinated isomer: Di-Chlorinated isomer (7.3:1). [d] Mono-Chlorinated isomer: Di-Chlorinated isomer (4.2:1). [e] Mono-Cl: Di-Cl isomers (17:1). [f] Major isomer shown. Mono-Chlorinated 3’-isomer and 5’-isomer (1.9:1).

To determine the scope and specificity of PrnC, we tested against a panel of substituted pyrroles (Fig. 5) featuring (a) different substituted aryl or heteroaryl groups, or cyclic and acyclic alkyl groups at C-3 to the pyrrolic fragment (see Supplementary Materials and Methods, section 1.7.1, scheme S1-2), (b) C-2 positional linkage of the pyrrolic fragment instead of C-3 (see Supplementary Materials and Methods, section 1.7.1, scheme S3), and c) other five-membered heterocycles instead of pyrroles (see Supplementary Materials and Methods, section 1.7.1, scheme S4). By employing the optimized conditions (Table 1, entry 3), PrnC was found to be able to accommodate a range of aryl or heteroaryl substituents at the C-3 of the pyrrole (products **2**–**9**) giving conversion ranging between 15% and 87% (see Supplementary Materials and Methods, section 1.8.2, Fig. S10 and section 1.8.3 Figs S11–24). Protecting the free amino group with acetanilide in product **2** significantly reduces the conversion yield (15%), which may be due to the limited space for an extra acetyl group in the active site (Fig. 3). For **4,** **5**, and **6**, the simultaneous replacement of the ortho-phenyl -NH2 group and removal of the phenyl 3-Cl atom had a moderate impact on the conversion (22%, 41%, and 26%), respectively. These results also confirm that prior installation of the 3-Cl atom by PrnA has no bearing on the subsequent step of halogenation. The total removal of substituents from the aryl fragment **7** (65%) or substitution with a heterocycle pyridine **8** (53%) or quinoline **9** (51%) yielded good conversion, indicating that the hydrophobic interaction of these phenyl groups embedded in the hydrophobic patch is highly relevant (see Supplementary Materials and Methods, section 1.8.2, Fig. S7a). These findings further suggest that the amino (–NH_2_) moiety on the adjacent aryl ring does not appear to play an active directing function in the halogenation step. Interestingly, positional isomeric or isosteric alternatives in the pyrrolic fragment were also accepted with 2′-pyrrole **10** and 5′-pyrazole **11** giving moderate conversions of 69% and 14%, respectively. Other five-membered heterocycles such as 4′-pyrazole **12**, furan, and thiophene or *N*-protected pyrroles (see Supplementary Materials and Methods, section 1.8.2, Fig. S9), however, were not tolerated by the PrnC enzyme. It is hypothesized that substrates without the free pyrrolic (N-H) functionality group are not favored by PrnC due to abrogating the effective hydrogen bond with the crucial residue E60, as illustrated in Fig. 3, despite having electron-rich rings that are susceptible to electrophilic substitution. Surprisingly, replacing the aromatic group on C3 of the pyrroles with a non-aryl cyclohexanone (see Supplementary Materials and Methods, section 1.7.1, scheme S5) is well-tolerated by the PrnC enzyme, suggesting the feasibility of accommodating alternative polar groups (entry 13, 51%). Notwithstanding, complete replacement of the aromatic substituent to an acyclic acrylate side chain (see Supplementary Materials and Methods, section 1.7.1, scheme S5) is also accepted, albeit with diminished conversion (entry 14, 19%). The regioselectivity of the bio-halogenation has been analogously assigned to be on the backbone of the pyrrolic fragment based on our computational model with further collaboration by 2D-NMR structural confirmation of the products (see Supplementary Data 1).

**Fig. 6:**
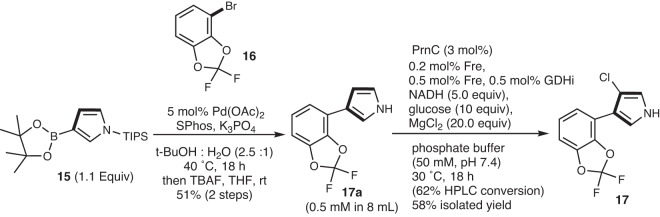
Synthesis of a chlorinated analog 17 of Fludioxonil. Our chemoenzymatic approach involves a palladium-mediated Suzuki coupling to construct the biaryl pyrrolic precursor **17a**, which then undergoes a late-stage site-selective chlorination to yield an analogue of the Fludioxonil fungicide, **17**.

Typically, substituted pyrroles are synthesized through the Van Leusen [3 + 2] cycloadddition reaction between tosylmethyl isocyanides (TosMICs)^56,57^ and pre-functionalized electron-deficient substrates, followed by deprotection. To showcase the applicability and scalability of the biocatalytic halogenation by PrnC, we applied the developed protocol towards the late-stage protecting-group free chlorination of a decyano-derivative **15** of an agrochemical fungicide, Fludioxonil. Substrate **15** was synthesized from **13** in two-steps using a Pd-catalyzed Suzuki coupling followed by tetra-*n*-butylammonium fluoride deprotection. Under both optimized as well as in vivo biosynthesis conditions, substrate **15** was selectively chlorinated at the C-3 position to give the chlorinated analog **16** of Fludioxonil in an isolated yield of 58% (Fig. 6).

## Conclusions

In conclusion, we report the first characterization of the activity and specificity of the pyrrolic halogenase PrnC towards its native substrate and a mini library of substituted pyrroles. This in vitro work was made possible by robust heterologous expression of PrnC *via* an 11-amino acid NT-11 solubility tag. Using cryo-EM, we also obtained the apo-structure of this enzyme as a dimeric state in solution though its significance is yet to be fully understood. The key residues that are responsible for PrnC’s catalytic activity have been identified by molecular modeling and validated by mutagenesis experiments. Leveraging the PrnC site-selective chlorination on the pyrrole fragment of the substrate, we applied this halogenase towards the chemoenzymatic synthesis of a C-3 chlorinated analog of the agrochemical fungicide, Fludioxonil. This work highlights the complementarity between chemical and biocatalytic methodologies to access certain chemical spaces or reactive positions. Further directed evolution studies are being conducted in our laboratories to improve the enzyme’s stability and promiscuity.

## Methods

### Halogenase cloning, expression, and purification

NT11-PrnC-6His construct in pET-28a(+) was ordered from Twist Biosciences as a clonal construct and transformed into T7 Express *E. coli* (NEB). The resulting strain was cultured in 1 L LB media at 37 °C. When OD600 reached 0.4, 0.1 mM IPTG was used to induce for overnight expression at 16 °C. After expression, the cultures were centrifuged at 10,000 g for 10 min at 4 °C. The resulting pellets were resuspended in 20 mL of 100 mM sodium phosphate pH 7, 10 mM imidazole, 150 mM sodium chloride before sonication. After sonication, the resulting lysate was then centrifuged at 19,000 g for 1 h at 4 °C. The supernatant was incubated with Ni-NTA agarose for 1 h at 4 °C. The resin was washed with 20 mL 100 mM sodium phosphate pH 7, 80 mM imidazole, 150 mM sodium chloride and the bound protein was eluted with 5 mL of 100 mM sodium phosphate pH 7, 500 mM imidazole, 50 mM sodium chloride. The elution was buffer exchanged and concentrated with 50 mM sodium phosphate pH 7, 10% glycerol.

### Homology and docking models of PrnC

Homology modeling was performed in Modeler v10 program and generated 100,000 homology models based on the template crystal structure of halogenase PltM (PDB code: 6BZA) whose sequence was aligned with that of PrnC (Fig. S5) with the sequence identity of 36%. Subsequently, all the models were subjected to the backbone, sidechain, and loop optimization, and then the top 50 optimized models with the lowest DOPE scores were used for the subsequent molecular docking with the native substrate (**1**) by using GOLD v2018 program with the optimal docking parameters (the binding pocket is defined by the ligand copied from the crystal structure of 6BZA with the spherical radius of 8.0 Angstrom; scoring function is GoldScore; population size is 500; the number of operations is 500000; number of island is 10; crossover frequency is 95%; mutation frequency is 95%; migration frequency is 20%; the number of output docking solutions is 3), which afforded 150 docking solutions in total.

In order to achieve an optimal model for the PrnC/**1a** complex, 150 docking solutions were subjected to further inspection based on the following two criteria: (1) there must be a nearby lysine residue stretching towards the pyrrole ring in **1a**, because a lysine is required for the catalysis; (2) the hydrogen on the pyrrolic nitrogen of **1a** should form a hydrogen bond with the hydrogen acceptor of a residue to stabilize the intermediate during the catalytic reaction. After the manual and visual examination, a reasonable model of PrnC/**1a** complex, which satisfied both criteria above, was harvested and shown in Fig. 3 and Fig. S7a (in Supplementary Materials and Methods, section 1.8.2).

### Cryo-EM

Single-particle Cryo-EM sample vitrification. Purified PrnC (in the buffer of 50 mM sodium phosphate pH 7, 10% glycerol) was concentrated using a 10-kDa molecular weight cutoff filter concentrator to 2.8 mg mL^−1^. A total of 2 μL of sample was added to a glow discharged (JEOL DATAM HDT-400) was applied onto the copper side of nanofabricated gold grids^58^ and blotted using filter paper on one side for 2 s using the Leica GP plunger system before plunging immediately into liquid ethane for vitrification. The plunger was operating at 5 °C with >80% humidity to minimize evaporation and sample degradation.

Single-particle Cryo-EM Data Collection and Processing. Images were recorded on a Titan Krios electron microscope (FEI) equipped with a BioQuantum K3 direct detector with energy filter operating at 0.8341 Å per pixel in electron counting mode using the SerialEM software package^59^. Pixel size was calibrated using apoferritin. Slit width was 20 eV. Data collection was performed using a dose of ~64 e^−^ Å^−2^ across 48 frames (125 ms per frame) at a dose rate of ~7.4 e^–^ pix^−1^ s^−1^, using a set defocus range of −0.8 to −1.8 μm. In all, 100-μm objective aperture was used. A total of 2763 micrographs were recorded over one day using an image beam shift data collection strategy^60^. Data processing was done using cryoSPARC 2.0^61^. Patch motion correction was applied to the movies, and patch CTF estimation was done. Blob picker was used to pick out the particles, and 2D classification was done to remove junk particles. Thereafter, particles looking like protein were put through ab initio with multiple models to further clean up the good particles. A final stack of 65,768 good particles was put through local motion correction, and then homogenous refinement to obtain a resolution of around 9 Å. The homology models of PrnC were docked into this final map using UCSF Chimera^62^.

### Analytical scale biotransformations

In a solution containing the pyrrolic derivative starting material (0.5 mM), MgCl_2_/MgBr_2_ (10 mM), glucose (5.0 mM), FAD (1.0 µM), NT-11 PrnC (12.5 µM), Fre (2.5 µM) and Gdhi (2.5 µM) in 10 mM potassium phosphate buffer, NADH (2.5 mM) was added to a total volume of 200 µL. After an overnight incubation of 30 °C and orbital shaking at 350 rpm, reactions were quenched with an equivalent volume of MeOH, pelleted by centrifugation (15000 rpm for 10 min), and the supernatant analyzed by HPLC-MS using the analytical HPLC method.

### Determination of kinetic parameters for NT-11 PrnC and PrnC mutant assay

Kinetic analysis of PrnC (2.5 μM) activity against MDA was performed over a 5-250 µM substrate concentration range. The assay reaction was supplemented with Fre (2.5 μM), FAD (1 μM), and MgCl_2_ (10 mM) in 20 mM Tris buffer, pH 7.4. NADH (2.5 mM) was added last for reaction initiation. The products formed were measured at 120, 300, and 600 s via a Kinetex XB-C18 reversed-phased column (2.6 µm, 150 × 4.6 mm) on a Shimadzu LC-20AD HPLC. Absorbance at λ = 254 nm was used to monitor product formation during an isocratic flow rate of 0.6 mL/min (50% MeCN/H_2_O + 0.1% TFA) over 10 min. Kinetic parameters were determined by nonlinear fitting of a Michaelis-Menten curve using the GraphPad Prism software. Activity assays for PrnC mutants were performed with the respective over-expressed protein variant. Wild-type PrnC enzyme was used as a positive control and reaction conditions were similar to the 18 h assay method described above.

### General HPLC and LC-MS methods

General considerations. Spectroscopic grade solvents were purchased from Sigma Aldrich. Low-resolution LC-MS spectra were recorded on an Agilent LCMS machine with dual MM-APCI-ES. High-resolution mass spectra (HRMS) were recorded on an Agilent ESI-TOF mass spectrometer at 3500 V emitter voltage. Exact m/z values are reported in Daltons.

Semi-Preparative HPLC method. 900 µL of the crude mixture dissolved in H_2_O/MeCN was injected into a Phenomenex Jupiter® semi-preparative C18 HPLC column (90 Å, 5 µm packing, 250 × 10 mm) and purified using reverse phase chromatography. Gradient starting conditions of 5% MeCN/H_2_O ( + 0.1% Formic acid) to 25% MeCN/H_2_O over 10 min, followed by 25% MeCN/H_2_O into 50% MeCN/H_2_O over 20 min, followed by 50% MeCN/H_2_O into 75% MeCN/H_2_O over 10 min, followed by 75% MeCN/H_2_O into 95% MeCN/H_2_O over 5 min, followed by a hold at 95% MeCN/H_2_O for 5 min. Column condition was equilibrated back to starting conditions over 2 min post-run. Flow rates were kept constant at at 3 mL/min. UV absorbance was monitored at 220 nm, 254 nm, and 280 nm.

Analytical HPLC Method. 10 µL of the supernatant injected onto SecurityGuard™ column (KJ0-4282) with a (4.0 mm × 3.0 mm) guard cartridge before separation using a Phenomenex Gemini® C18 analytical column (5 µm packing, 150 mm × 4.6 mm). Gradient starting conditions of 5% MeCN/H_2_O ( + 0.1% Formic acid) were held for 1 min before development into 50% MeCN/H_2_O over 3 min, followed by development into 95% MeCN/H_2_O over 3 min. 95% MeCN/H_2_O was held for 1 min before equilibration back to starting conditions over 1 min. Starting conditions were held for 1 min followed by another 2 min post-run. Flow rates were kept constant at 1 mL/min. Column temperature was kept constant at 30 °C. UV absorbance was detected at 220 nm, 254 nm, and 210 nm throughout the run.

General LC-MS Method. 10 µL of the supernatant was separated using the appropriate analytical HPLC method described above. Detection was performed using an Agilent® single quadrupole LC/MSD system.

### Reporting summary

Further information on research design is available in the Nature Portfolio Reporting Summary linked to this article.

### Supplementary information

Supplementary Information

Supplementary Data 1

Description of Additional Supplementary Files

Reporting Summary

## Data Availability

Electronic Supplementary Information contains comprehensive experimental procedures for the synthesis and characterization of new compounds as well as kinetic studies and LCMS analysis. Supplementary Data 1 contains the ^1^H, ^13^C, and 2D NMR data spectra of the isolated new compounds.
