# Supplementary material for: Site-selective chlorination of pyrrolic heterocycles by flavin dependent enzyme PrnC
Source: Commun Chem. 2024 Jan 5;7:7. doi: 10.1038/s42004-023-01083-1 (PMC10770391; doi:10.1038/s42004-023-01083-1)
Supplement: Supplementary file 1 — Supplementary Information [file 42004_2023_1083_MOESM1_ESM.pdf]

Supporting Information

Site-selective Chlorination of Pyrrolic Heterocycles by Flavin Dependent Enzyme PrnC

GuangRong Peh,[a] Terence Tay,[b] Lee Ling Tan,[c] Elaine Tiong,[c] Jiawu Bi,[c] Yi Ling Goh,[a] Suming Ye,[a] Fu Lin,[b] Cheryl Jia Xin Tan,[d] Yong Zi Tan,[d,e,f] Joel Wong,[a] Huimin Zhao,[b,g] Fong Tian Wong,*[a, c] Ee Lui Ang,*[b,h] and Yee Hwee Lim*[a,h]

Affiliations:

a) Institute of Sustainability for Chemicals, Energy and Environment (ISCE2), Agency for Science, Technology and Research (A*STAR), 8 Biomedical Grove, Neuros #07-01, Singapore 138665, Republic of Singapore.

b) Singapore Institute of Food and Biotechnology Innovation (SIFBI), Agency for Science, Technology and Research (A*STAR), 31 Biopolis Way, Nanos #01-02, Singapore 138669, Republic of Singapore.

c) Institute of Molecular and Cell Biology (IMCB), Agency for Science, Technology and Research (A*STAR), 61 Biopolis Dr, Proteos #07-01, Singapore 138673, Republic of Singapore.

d) Department of Biological Sciences, National University of Singapore. 16 Science Drive 4, Singapore 117558, Singapore.

e) Disease Intervention Technology Laboratory (DITL), Agency for Science, Technology and Research (A*STAR), 8A Biomedical Grove, Singapore 138648, Republic of Singapore.

f) Institute of Molecular and Cell Biology (IMCB), Agency for Science, Technology and Research (A*STAR), 61 Biopolis Dr, Singapore 138673, Republic of Singapore.

g) Department of Chemical and Biomolecular Engineering, Carl R. Woese Institute for Genomic Biology. University of Illinois at Urbana-Champaign, Urbana, IL 61801, USA.

h) Synthetic Biology Translational Research Program, Yong Loo Lin School of Medicine, National University of Singapore, 10 Medical Drive, Singapore 117597, Republic of Singapore.

Corresponding authors:

FTW: wongft@imcb.a-star.edu.sg;

ELA: ang_ee_leui@sifbi.a-star.edu.sg;

YHL: lim_yee_hwee@isce2.a-star.edu.sg

1. Supplementary Materials and Methods 3
1.1 Sequences – NT11-PrnC – nt 4
1.2 Homology models of PrnC. 5
1.3 Sequence Similarity Network (SSN) for PrnC homologs[2] 6
1.4 Cryo-EM 6
1.5 Biotransformations 7
1.5.1 Analytical Scale 7
1.5.2 Preparative Scale Cell Lysate 8
1.6 General HPLC and LC-MS methods 8
1.7 Synthesis and Characterization 9
1.7.1 Synthesis of Substrates & Standards 9
1.7.2 Characterization of chlorinated products. 14
1.7.3 Synthesis of substrate 17a and chlorinated Fludioxonil analog 17 17
1.8 Supplementary Tables and Figures 19
1.8.1 Gels – labelled and annotated 20
1.8.2 HPLC traces and Charts/graphs 22
1.8.3 LC-MS analytical data for substrates panel using purified NT11-PrnC 33
2. Supplementary References 47

1. Supplementary Materials and Methods

Halogenase cloning, expression and purification. NT11[1]-PrnC-6His construct in pET-28a(+) was ordered from Twist Biosciences as a clonal construct and transformed into *T7 Express E. coli* (NEB). The resulting strain was cultured in 1 L LB media at 37 °C. When OD_{600} reached 0.4, 0.1 mM IPTG was used to induce for overnight expression at 16 °C. After expression, the cultures were centrifuged at 10, 000 *g* for 10 minutes at 4°C. The resulting pellets were resuspended in 20 mL of 100mM sodium phosphate pH 7, 10 mM imidazole, 150 mM sodium chloride before sonication. After sonication, the resulting lysate was then centrifuged at 19,000 *g* for 1 hour at 4°C. The supernatant was incubated with Ni-NTA agarose for 1 hour at 4°C. The resin was washed with 20 mL 100mM sodium phosphate pH 7, 80 mM imidazole, 150 mM sodium chloride and the bound protein was eluted with 5 mL of 100mM sodium phosphate pH 7, 500 mM imidazole, 50 mM sodium chloride. The elution was buffer exchanged and concentrated with 50 mM sodium phosphate pH 7, 10% glycerol.

Gdhi. Purchased from Sigma-aldrich with activity units ⩾200 U/mg.

Method for Fre cloning, expression and purification. The nucleic acid sequence that encodes for Flavin reductase Fre was purchased as a gBlock from Integrated DNA Technologies. Fre sequence was cloned into pET-28a(+) vector via NEBuilder® HiFi DNA Assembly method and transformed into *E. coli Acella* (EdgeBio). *E. Coli* strain expressing Fre was cultured in 1 L of LB Kan^{50} media at 37 °C. At OD_{600} 0.4-0.6, 0.1 mM IPTG was used to induce protein expression at 16 °C over 18 h. Cell culture was harvested by centrifugation at 4000 rcf for 10 min at 4 °C. After media was decanted, cell pellet was resuspended in 30 ml of 50 mM tris pH 7.4, 300 mM sodium chloride, 10 mM imidazole and lysed by cell disruption. Cell lysate was centrifuged at 33,600 rcf for 45 min at 4 °C to differentiate supernatant from insoluble debris. Fre proteins from lysate supernatant were purified using immobilized metal affinity chromatography via TALON resins interaction with N-terminus His-tag Fre. After lysate supernatant was applied, 10 ml of 50 mM tris pH 7.4, 300 mM sodium chloride, 10 mM imidazole was used to wash the resins. Fre proteins were eluted from the resins using 5 ml of 50 mM tris pH 7.4, 300 mM sodium chloride, 200 mM imidazole. Eluted samples were buffer exchanged and concentrated with 50 mM tris pH 7.4, 100 mM NaCl, 10% glycerol

Method for PrnC mutants construction in mutagenesis studies. Single-site mutations of the *prnC* gene were constructed via overlap extension PCR, using PrimeSTAR Max DNA Polymerase, on the pET-28a(+) NT11-PrnC-6His plasmid template. The mutagenesis primers used are as shown: E60A forward primer 5'-CGAAAGTTCCATCCCGGCGACTTCGTTGATGAATC-3'; E60A reverse primer 5'-GATTCATCAACGAAGTCGCCGGGATGGAACTTTCG-3'; K97A forward primer 5'-CATCGTCAACCGGAATTGCGCGTAATTTCGGCTTTG-3'; K97A reverse primer 5'-

CAAAGCCGAAATTACGCGCAATTCCGGTTGACGATG-3’; E129A forward primer 5’-GCTTCCCTGGGGACCTGCGTCACATTATTATCGTC-3’; E129A reverse primer 5’-GACGATAATAATGTGACGCAGGTCCCCAGGGAAGC-3’. Standard flanking primers for *prnC* gene were used for amplification of the respective PCR segments in each mutant construct: prnC forward primer 5’-GGAGATATACCATGGTAAGTGAACCCCACGACTATAATTATG-3’; prnC reverse primer 5’-GTGGTGGTGCTCGAGTTTTTTCAGCGCTAATCCAATACGC-3’. PCR segments and linearized plasmids were ligated using NEBuilder Hifi DNA assembly protocol and the mutations were confirmed by DNA sequencing.

1.1 Sequences – NT11-PrnC – nt

ATGGTAAGTGAACCCCACGACTATAATTATGAGAAAGCTAGCGCGTCTATGACACAAAAATCTCCCG
CCAACGAGCACGATTCAAATCACTTTGATGTCATTATTCTGGGATCGGGCATGTCAGGTACGCAGAT
GGGCGCTATTTTGGCTAAACAACAATTTCGTGTGTTAATTATTGAAGAGTCCAGCCACCCACGTTTTA
CCATTGGCGAAAGTTCCATCCCGGAAACTTCGTTGATGAATCGTATCATCGCAGACCGCTATGGTAT
CCCAGAACTGGACCATATTACCTCCTTTTATAGCACACAACGTTACGTCGCATCGTCAACCGGAATTA
AACGTAATTTCGGCTTTGTATTTCACAAACCAGGTCAAGAGCACGATCCTAAGGAATTCACACAGTGT
GTGATTCCGGAGCTTCCCTGGGGACCTGAATCACATTATTATCGTCAGGACGTAGATGCATATTTATT
GCAGGCGGCGATCAAGTACGGGTGTAAAGTGCACCAAAAGACAACTGTGACGGAGTATCATGCTGA
TAAGGATGGCGTAGCGGTCACAACAGCTCAAGGGGAACGCTTCACAGGTCGCTACATGATCGATTG
CGGTGGACCCCGCGCGCCCTTGGCTACTAAATTCAAGTTACGTGAAGAGCCATGCCGTTTTAAAACG
CACAGCCGTTCTCTGTATACTCACATGTTAGGGGTCAAGCCATTCGACGACATTTTTAAAGTAAAGGG
GCAGCGCTGGCGTTGGCATGAAGGAACCCTTCATCACATGTTCGAAGGAGGATGGCTGTGGGTGAT
TCCCTTTAATAACCATCCCCGTAGCACAAATAACTTAGTCTCGGTTGGTTTGCAACTTGACCCACGTG
TCTACCCCAAGACCGACATCTCCGCTCAACAAGAATTCGACGAGTTCCTTGCCCGTTTTCCGTCAAT
CGGAGCCCAGTTTCGCGACGCCGTTCCAGTTCGCGATTGGGTGAAAACTGATCGCCTTCAATTTTCT
AGTAATGCATGCGTCGGAGACCGTTACTGTTTAATGTTACACGCGAACGGATTTATTGATCCGTTGTT
TTCGCGTGGGCTTGAGAATACTGCGGTCACGATCCACGCCTTAGCTGCGCGTCTTATCAAGGCCCT
GCGCGACGATGACTTCTCTCCAGAACGCTTCGAGTATATCGAGCGTTTGCAACAGAAGTTGTTGGAC
CACAATGACGACTTCGTGTCATGTTGCTACACAGCTTTTTCGGATTTTCGTCTTTGGGATGCCTTTCA
CCGCCTTTGGGCCGTGGGGACTATCCTTGGACAATTTCGTCTTGTGCAGGCCCACGCACGCTTTCG
CGCATCGCGTAACGAGGGTGATCTGGATCATTTAGATAACGACCCACCCTATTTGGGGTATCTGTGC
GCTGATATGGAAGAGTACTATCAGCTTTTCAATGACGCCAAGGCTGAGGTGGAAGCAGTATCAGCG
GGACGTAAGCCTGCCGACGAGGCTGCCGCGCATTCATGCCTTGATCGACGAACGTGACTTCGCC
AAACCTATGTTCGGGTTCGGTTACTGCATTACAGGAGATAAACCACAGTTGAATAATTCCAAGTACTC
TTTATTGCCTGCGATGCGCTTGATGTACTGGACGCAGACCCGTGCCCCAGCGGAAGTCAAGAAGTA

TTTCGATTATAACCCTATGTTTGCGTTGCTTAAGGCATATATTACTACGCGTATTGGATTAGCGCTGAA
AAAACTCGAGCACCACCACCACCACCACTGA

Sequences – NT11-PrnC - aa

MVSEPHDYNYEKASASMTQKSPANEHDSNHFDVIILGSGMSGTQMGAILAKQQFRVLIIEESSHPRFTIGE
SSIPETSLMNRIIADRYGIPELDHITSFYSTQRYVASSTGIKRNFGFVFHKPGQEHDPKEFTQCVIPELPWG
PESHYYRQDVDAYLLQAAIKYGCKVHQKTTVTEYHADKDGVAVTTAQGERFTGRYMIDCGGPRAPLATK
FKLREEPCRFKTHSRSLYTHMLGVKPFDDIFKVKGQRWRWHEGTLHHMFEGGWLWVIPFNNHPRSTNN
LVSVGLQLDPRVYPKTDISAQQEFDEFLARFPSIGAQFRDAVPVRDWVKTDRLQFSSNACVGDRYCLML
HANGFIDPLFSRGLENTAVTIHALAARLIKALRDDDFSPERFEYIERLQQKLLDHNDDFVSCCYTAFSDFRL
WDAFHRLWAVGTILGQFRLVQAHARFRASRNEGDLDHLDNDPPYLGYLCADMEEYYQLFNDAKAEVEA
VSAGRKPADEAAARIHALIDERDFAKPMFGFGYCITGDKPQLNNSKYSLLPAMRLMYWTQTRAPAEVKKY
FDYNPMFALLKAYITTRIGLALKKLEHHHHHH*

1.2 Homology models of PrnC.

Homology modelling was performed in Modeller v10 program and generated 100,000 homology models based on the template crystal structure of halogenase PltM (PDB code: 6BZA) whose sequence was aligned with that of PrnC (*Fig. S5*) with the sequence identity of 36%. Subsequently, all the models were subjected to the backbone, sidechain and loop optimization, and then the top 50 optimized models with the lowest DOPE scores were used for the subsequent molecular docking with the native substrate (**1**) by using GOLD v2018 program with the optimal docking parameters (the binding pocket is defined by the ligand copied from the crystal structure of 6BZA with the spherical radius of 8.0 Angstrom; scoring function is GoldScore; population size is 500; the number of operations is 500000; number of island is 10; crossover frequency is 95%; mutation frequency is 95%; migration frequency is 20%; the number of output docking solutions is 3), which afforded 150 docking solutions in total.

In order to achieve an optimal model for the PrnC/**1a** complex, 150 docking solutions were subjected to the further inspection based on the following two criteria: (1) there must be a nearby lysine residue stretching towards the pyrrole ring in **1a**, because a lysine is required for the catalysis; (2) the hydrogen on the pyrrolic nitrogen of **1a** should form a hydrogen bond with the hydrogen acceptor of a residue to stabilize the intermediate during the catalytic reaction. After the manual and visual examination, a reasonable model of PrnC/**1a** complex**,** which satisfied both criteria above, was harvested and shown in **Figure 3** and **Figure S12**.

1.3 Sequence Similarity Network (SSN) for PrnC homologs[2]

EFI-EST tool for generating SSN

The online EFI-Enzyme Similarity Tool offered by Illinois Carl R. Woese Institute for Genomic Biology was used to generate the SSN for PrnC. The amino acid sequence of native PrnC (Uniprot: P95482) was used as the query sequence to generate the SSN for a single protein compared to its closest homologues in the UniProtKB database. An all-by-all BLAST was performed to obtain the similarities between each sequence pair to calculate edge values to generate the SSN. The BLAST retrieval options selected were the default values of 5 E-value and 1000 maximum number of retrieved sequences. SSN edge calculation E-value used was the default value of 5. After SSN generation, the alignment score of 185 (corresponding to 60% protein identity) was selected as the threshold value to filter the nodes and reduce complexity. A representative node network of 80% ID (highly identical proteins were collapsed into a single node to deconvolute the network) was created from the filtered SSN and visualized using the software Cytoscape. The final node count is 492.

[Cytoscape SSN visualization]

Within Cytoscape, yFiles Organic Layout was selected as the SSN display layout. Stray, unclustered proteins were removed from the SSN to remove clutter and increase resolution. Node-filters was used to differentiate between the various cluster of proteins. The "phylum" descriptor was used as the grouping criteria to separate the individual nodes of the network into 7 major protein clusters ("actinobacteria", "verrucomicrobia", "proteobacteria", "chloroflexi", "planctomycetes", "cyanobacteria", "euryarchaeota") and were colour-coded for easier viewing. Uncoloured nodes did not belong to any of the major clusters or had insufficient data for their database entries. Native PrnC, homologs 1D2, 1E2, 1F2 and 5.7 had Uniprot ascension codes of P95482, A0A250ITU6, Q9RPG1, A0A150PA41 and I1V4X2 respectively. These ascension codes were input into the Cytoscape search function to select and highlight their respective nodes in the SSN. NCBI Protein BLAST tool was used to carry out pairwise protein alignment to compare the degree of similarity between native PrnC protein against the 4 chosen homologs.

1.4 Cryo-EM

Single-particle Cryo-EM sample vitrification

Purified PrnC (in buffer of 50 mM sodium phosphate pH 7, 10% glycerol) was concentrated using a 10-kDa molecular weight cutoff filter concentrator to 2.8 mg mL^{-1}. 2 µL of sample was added to a glow discharged (JEOL DATAM HDT-400) was applied onto the copper side of nanofabricated gold grids[3] and blotted using filter paper on one side for 2 s using the Leica GP plunger system before plunging immediately into liquid ethane for vitrification. The plunger was operating at 5 °C with >80% humidity to minimize evaporation and sample degradation.

Single-particle Cryo-EM Data Collection and Processing

Images were recorded on a Titan Krios electron microscope (FEI) equipped with a BioQuantum K3 direct detector with energy filter operating at 0.8341 Å per pixel in electron counting mode using the SerialEM software package[4]. Pixel size was calibrated using apoferritin. Slit width was 20 eV. Data collection was performed using a dose of ~64 e^- $Å^{-2}$ across 48 frames (125 ms per frame) at a dose rate of ~7.4 e^- pix^{-1} s^{-1}, using a set defocus range of −0.8 to −1.8 µm. In all, 100-µm objective aperture was used. A total of 2,763 micrographs were recorded over one day using an image beam shift data collection strategy[5].

Data processing was done using cryoSPARC 2.0[6]. Patch motion correction was applied to the movies, and patch CTF estimation was done. Blob picker was used to pick out the particles, and 2D classification was done to remove junk particles. Thereafter, particles looking like protein was put through *ab initio* with multiple models to further clean up the good particles. A final stack of 65,768 good particles were put through local motion correction, then homogenous refinement to obtain a resolution of around 9 Å. The homology models of PrnC was docked into this final map using UCSF Chimera[7].

1.5 Biotransformations

1.5.1 Analytical Scale

NT-11 PrnC-Catalyzed halogenation. In a solution containing the pyrrolic derivative starting material (0.5 mM), $MgCl_2$/ $MgBr_2$ (10 mM), glucose (5.0 mM), FAD (1.0 µM), NT-11 PrnC (12.5 µM), Fre (2.5 µM) and Gdhi (2.5 µM) in 10mM potassium phosphate buffer, NADH (2.5 mM) was added to a total volume of 200 µL. After an overnight incubation of 30°C and orbital shaking at 350 rpm, reactions were quenched with an equivalent volume of MeOH, pelleted by centrifugation (15000 rpm for 10 min) and the supernatant analyzed by HPLC-MS using the analytical HPLC method.

Determination of Kinetic Parameters for NT-11 PrnC and PrnC mutant assay. Kinetic analysis of PrnC (2.5 µM) activity against MDA was performed over a 5-250 µM substrate concentration range. The assay reaction was supplemented with Fre (2.5 µM), FAD (1 µM) and $MgCl_2$ (10 mM) in 20 mM Tris buffer, pH 7.4. NADH (2.5 mM) was added last for reaction initiation. The products formed were measured at 120, 300 and 600 seconds via a Kinetex XB-C18 reversed-phased column (2.6 µm, 150 x 4.6 mm) on a Shimadzu LC-20AD HPLC. Absorbance at λ = 254 nm was used to monitor product formation during an isocratic flow rate of 0.6 mL/min (50% $MeCN/H_2O$ + 0.1% TFA) over 10 min. Kinetic parameters were determined by nonlinear fitting of a Michaelis-Menten curve using the GraphPad Prism software. Activity assays for PrnC mutants were performed in lysates with the respective over-expressed protein variant. Wild-type PrnC enzyme was used as a positive control and reaction conditions were similar to the 18 h assay method described above.

1.5.2 Preparative Scale Cell Lysate

Method for PrnC lysate preparation. *E. Coli* strain expressing PrnC was cultured in 1 L of LB Kan^{50} media at 37 °C. At OD_{600} 0.4-0.6, 0.1 mM IPTG was used to induce protein expression at 16 °C over 18 h. Cell culture was harvested by centrifugation at 4000 rcf for 10 min at 4 °C. After media was decanted, cell pellet was resuspended in 30 ml of 50 mM tris pH 7.4, 300 mM sodium chloride, 10 mM imidazole and lysed by cell disruption. Cell lysate was centrifuged at 33,600 rcf for 45 min at 4 °C to differentiate supernatant from insoluble debris. Lysate supernatant was buffer exchanged into 50 mM tris pH 7.4 in preparation for activity assay.

PrnC-Cell lysate Chlorination. In a solution of the PrnC cell lysate (0.01 mM, enzyme loading ~2.0 mol%, total volume 20 mL), the pyrrolic derivative starting material (0.5 mM), $MgCl_2$ (10 mM), glucose (5.0 mM), FAD (1.0 µM), Fre (2.5 µM), GDH2 (2.5 µM), NADH (2.5 mM) was added and allowed to stir at 30°C with a stir bar in a petri dish at 30 rpm. After an overnight incubation of 25°C and stir-bar shaking at 30 rpm, reactions were quenched with an equivalent volume of (1:1) MeOH-brine solution. The aqueous layer was 3x extracted with ethyl acetate pelleted by centrifugation (40 rpm for 10 min). The combined organic layers were dried over Na_2SO_4, filtered, and concentrated before purification by semi-preparative HPLC.

PrnC-Cell *In-vivo* biosynthetic Chlorination. PrnC was overexpressed in *E. coli* T7 Express (DE3) cells (NEB). LB medium containing 50ug/mL kanamycin was used for the preparation of a starter culture grown overnight at 37°C. The cultures were diluted 1:100 and cultured in LB at 37 °C, 200 rpm until OD600 reached 0.4-0.6. 100 µM isopropyl β-D-1-thiogalactopyranoside (IPTG) was then added to induce the expression of proteins. The pyrrolic derivative starting material (ca. 0.01-0.08 mM) were added three hours after IPTG induction, and the cultures were maintained at 25°C, 200 rpm for another 45 hours. Using a centrifuge maintained at 4°C, the culture was spun down at 10,000xg to separately harvest the supernatant and pellet for analysis. The aqueous LB layer was 3x extracted with 300 mL of ethyl acetate while the pellets were vortex, sonicated and extracted with additional (3 x 50mL) of MeOH. The combined organic layers were filtered through a plug of celite and dried over anhydrous sodium sulphate. The solvent was concentrated *in vacuo* followed by before purification by preparative thin-layer chromatography.

1.6 General HPLC and LC-MS methods

Analytical methods: Spectroscopic grade solvents were purchased from Sigma Aldrich. Low-resolution LC-MS spectra were recorded on an Agilent LCMS machine with dual MM-APCI-ES. High-resolution mass spectra (HRMS) were recorded on an Agilent ESI-TOF mass spectrometer at 3500 V emitter voltage. Exact m/z values are reported in Daltons.

Semi-Preparative HPLC method. 900 µL of the crude mixture dissolved in H_2O/MeCN was injected onto a Phenomenex Jupiter® semi-preparative C18 HPLC column (90Å, 5 µm packing, 250 x 10 mm) and purified using reverse phase chromatography. Gradient starting conditions of 5% MeCN/H_2O (+0.1% Formic acid) to 25% MeCN/H_2O over 10 min, followed by 25% MeCN/H_2O into 50% MeCN/H_2O over 20 min, followed by 50% MeCN/H_2O into 75% MeCN/H_2O over 10 min, followed by 75% MeCN/H_2O into 95% MeCN/H_2O over 5 min, followed by a hold at 95% MeCN/H_2O for 5 min. Column condition was equilibrated back to starting conditions over 2 mins post-run. Flow rates were kept constant at at 3 mL/ min. UV absorbance was monitored at 220 nm, 254 nm and 280 nm.

Analytical HPLC Method. 10 µL of the supernatant injected onto SecurityGuard™ column (KJ0-4282) with a (4.0 mm x 3.0 mm) guard cartridge before separation using a Phenomenex Gemini® C18 analytical column (5 µm packing, 150 mm x 4.6 mm). Gradient starting conditions of 5% MeCN/H_2O (+0.1% Formic acid) were held for 1 min before development into 50% MeCN/H_2O over 3 min, followed by development into 95% MeCN/H_2O over 3 min. 95% MeCN/H_2O was held for 1 min before equilibration back to starting conditions over 1 min. Starting conditions was held for 1 min followed by another 2 min post-run. Flow rates were kept constant at 1 mL/ min. Column temperature was kept constant at 30 °C. UV absorbance was detected at 220 nm, 254 nm and 210 nm throughout the run.

General LC-MS Method. 10 µL of the supernatant was separated using the appropriate analytical HPLC method described above. Detection was performed using an Agilent® single quadrupole LC/MSD system.

1.7 Synthesis and Characterization

1.7.1 Synthesis of Substrates & Standards

All chemicals were purchased from Sigma-Aldrich, Alfa Aesar, Merck and TCI and were used as received. Chemicals and anhydrous solvents were obtained from Sigma Aldrich and were used without further purification. Spectroscopic grade solvents were purchased from Sigma Aldrich. Proton (^{1}H NMR) and carbon (^{13}C NMR) nuclear magnetic resonance spectra were recorded on a Bruker Avance 400 spectrometer with CryoProbe at 400 MHz and 100 MHz. The chemical shifts are reported in parts per million (ppm) on the delta (δ) scale. The solvent peak was used as a reference value, for ^{1}H NMR: $CDCl_3$ = 7.27 ppm, CD_3OD = 3.31 ppm, CD_2Cl_2 = 5.32 ppm for ^{13}C NMR: $CDCl_3$ = 77.23, CD_3OD = 49.0 ppm, CD_2Cl_2 = 53.50 ppm. NMR spectra were processed using MestReNova 10.0.2. Data are reported as follows: (s = singlet; d = doublet; t = triplet; q = quartet; sept = septet; dd = doublet of doublets; ddd = doublet of doublet of doublets; dddd = doublet of doublet of doublet of doublet; td = triplet of doublets; dtd = doublet of triplet of doublets; br = broad). Analytical TLC was performed on E. Merck pre-coated (25 mm) silica gel 60F-254 plates. Visualization was done under UV (254 nm). Flash column chromatography was carried out using

Merck 60 F254, 0.040-0.063 μm silica gel. Preparative TLC chromatography was carried out using Merck 60 F254, 0.25 μm silica gel plates. High-resolution mass spectra (HRMS) were recorded on an Agilent ESI-TOF mass spectrometer at 3500 V emitter voltage.

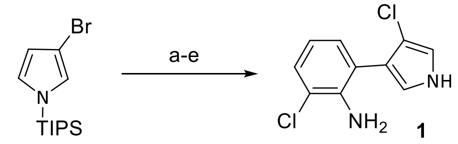

Reagents and conditions

a) t-BuLi, THF, -78 °C, than C_2Cl_6/ THF, -78 °C to rt, 68%. b) NIS, Acetone, rt, 76%.
c) HBPin, $PdCl_2(CH_3CN)_2$,Sphos, PhMe, 90 °C. d) 2-bromo-6-chloroaniline, $PdAc_2$, K_3PO_4,
Sphos, BuOH/H_2O, 40 °C, 38% (2 steps).e) TBAF, THF, rt, 68%

Scheme S1. Synthesis of **1** [Note: Substrates **2a**, **3a**, **4a**, **5a**, **6a**, **7a** and **9a** were prepared through similar sequences].

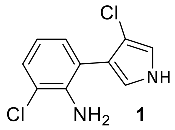

2-chloro-6-(4-chloro-1H-pyrrol-3-yl)aniline (1)[8, 9]

^{1}H NMR (400 MHz, CDCl3) δ 8.30 (s, 1H), 7.24 (dd, J = 8.0, 1.5 Hz, 1H), 7.09 (dd, J = 7.6, 1.5 Hz, 1H), 6.86 (ddd, J = 15.4, 2.9, 2.3 Hz, 2H), 6.71 (t, J = 7.8 Hz, 1H), 4.16 (s, 2H). ^{13}C NMR (100 MHz, CDCl3) δ 141.7, 130.0, 128.5, 120.1, 119.4, 119.3, 117.92, 117.2, 116.2, 112.2. This is in accordance with literature data.

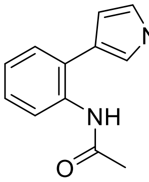

2a

N-(2-(1H-pyrrol-3-yl)phenyl)acetamide (2a)

^{1}H NMR (400 MHz, $CDCl_3$) δ 8.52 (s, 1H), 8.31 (d, *J* = 8.2 Hz, 1H), 7.71 (s, 1H), 7.34 – 7.23 (m, 2H), 7.14 – 7.06 (m, 1H), 6.96 – 6.88 (m, 2H), 6.36 (q, *J* = 2.4 Hz, 1H), 2.10 (s, 3H). ^{13}C NMR (101 MHz, $CDCl_3$) δ=168.2, 135.2, 129.9, 127.3, 126.2, 124.0, 120.7, 120.7, 119.2, 116.7, 108.8, 24.8. IR (neat) 3245, 1624, 1602, 1527, 1373, 1298, 1084, 759 cm^{-1}. HRMS (ESI) calcd. for $C_{12}H_{13}N_2O$ m/z $(M+H)^+$: 201.1022, found: 201.1016 .

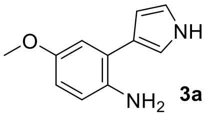

4-methoxy-2-(1H-pyrrol-3-yl)aniline (3a)

^{1}H NMR (400 MHz, $CDCl_3$) δ=8.36 (s, 1H), 7.02 (dt, *J* = 2.6, 1.8 Hz, 1H), 6.94 – 6.84 (m, 2H), 6.75 – 6.64 (m, 2H), 6.46 (td, *J* = 2.7, 1.6 Hz, 1H), 3.77 (s, 3H), 3.70 (s, 2H). ^{13}C NMR (101 MHz, $CDCl_3$) δ=152.7, 137.5, 123.5, 122.0, 118.5, 116.7, 116.4, 115.3, 112.9, 108.7, 55.8. IR (neat) 3351, 3284, 2963, 1615, 1508, 1475, 1284, 1241, 1207, 871, 806, 746, 726 cm^{-1}. HRMS (ESI) calcd. for $C_{11}H_{13}N_2O$ m/z $(M+H)^+$: 189.1022, found: 189.1027.

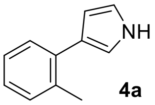

2-(o-tolyl)-1H-pyrrole (4a)[10]

^{1}H NMR (400 MHz, $CDCl_3$) δ 8.29 (s, 1H), 7.39 (dd, *J* = 7.5, 1.7 Hz, 1H), 7.27 – 7.11 (m, 3H), 6.90 (dt, *J* = 2.6, 1.8 Hz, 1H), 6.85 (td, *J* = 2.7, 2.0 Hz, 1H), 6.42 (td, *J* = 2.7, 1.6 Hz,

1H), 2.44 (s, 3H). ^{13}C NMR (101 MHz, $CDCl_3$) δ=135.8, 135.3, 130.5, 129.3, 125.9, 125.8, 124.4, 117.6, 116.6, 109.5, 21.4. HRMS (ESI) calcd. for $C_{11}H_{12}N$ m/z $(M+H)^+$: 158.0961, found: 158.0970. This is in accordance with literature data.

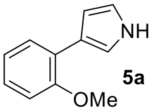

5a

2-(2-methoxyphenyl)-1H-pyrrole (5a)[11]

^{1}H NMR (400 MHz, $CDCl_3$) δ=8.27 (s, 1H), 7.56 (dd, *J* = 7.6, 1.7 Hz, 1H), 7.36 (dt, *J* = 2.7, 1.8 Hz, 1H), 7.17 (ddd, *J* = 8.2, 7.4, 1.8 Hz, 1H), 7.03 – 6.91 (m, 2H), 6.84 (td, *J* = 2.8, 2.0 Hz, 1H), 6.64 (td, *J* = 2.8, 1.5 Hz, 1H), 3.90 (s, 3H). ^{13}C NMR (101 MHz, $CDCl_3$) δ 156.1, 128.0, 126.3, 124.6, 120.8, 120.4, 117.9, 117.7, 111.1, 108.0, 55.4. HRMS (ESI) calcd. for $C_{11}H_{12}NO$ m/z $(M+H)^+$: 174.0913, found: 174.0919. This is in accordance with literature data.

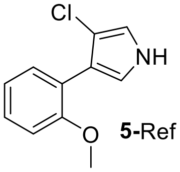

5-Ref

3-chloro-4-(2-methoxyphenyl)-1H-pyrrole (5-Ref): Synthetic reference standard for **5**
^{1}H NMR (400 MHz, Methanol-d_4) δ 7.43 (dd, *J* = 7.5, 1.8 Hz, 1H), 7.24 (ddd, *J* = 8.3, 7.4, 1.8 Hz, 1H), 7.02 (dd, *J* = 8.2, 1.2 Hz, 1H), 6.96 (td, *J* = 7.5, 1.2 Hz, 1H), 6.89 (d, *J* = 2.4 Hz, 1H), 6.78 (d, *J* = 2.4 Hz, 1H), 3.82 (s, 3H). ^{13}C NMR (101 MHz, Methanol-D_4) δ 159.2, 132.9, 129.4, 125.4, 122.1, 120.1, 119.5, 117.2, 113.1, 112.9, 56.8. HRMS (ESI) calcd. for $C_{11}H_{11}ClNO$ m/z $(M+H)^+$: 208.0524, found: 208.0534.

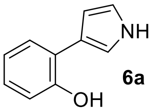

6a

2-(1H-pyrrol-2-yl)phenol (6a)

^{1}H NMR (400 MHz, $CDCl_3$) δ 8.44 (s, 1H), 7.33 (dd, *J* = 7.5, 1.8 Hz, 1H), 7.17 (ddd, *J* = 8.1, 7.3, 1.7 Hz, 1H), 7.05 (q, *J* = 2.0 Hz, 1H), 7.00 – 6.89 (m, 3H), 6.44 (td, *J* = 2.7, 1.6 Hz, 1H), 5.60 (s, 1H). ^{13}C NMR (101 MHz, $CDCl_3$) δ=152.7, 129.4, 127.7, 122.6, 120.5, 119.5, 119.4, 116.4, 115.2, 108.4. IR (neat) 3418, 1452, 753 cm^{-1}. HRMS (ESI) calcd. for $C_{10}H_{10}NO$ m/z $(M+H)^+$: 160.0757, found: 160.0762.

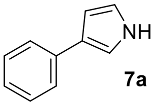

7a

3-phenyl-1H-pyrrole (7a)[12]

^{1}H NMR (400 MHz, $CDCl_3$) δ=8.25 (s, 1H), 7.60 – 7.52 (m, 2H), 7.40 – 7.31 (m, 2H), 7.24 – 7.15 (m, 1H), 7.10 (dt, *J* = 2.6, 1.8 Hz, 1H), 6.84 (td, *J* = 2.7, 2.0 Hz, 1H), 6.57 (td, *J* = 2.7, 1.6 Hz, 1H). ^{13}C NMR (101 MHz, $CDCl_3$) δ 135.8, 128.6, 125.5, 125.3, 125.0, 118.9, 114.6, 106.6. HRMS (ESI) calcd. for $C_{10}H_{10}N$ m/z $(M+H)^+$: 144.0808, found: 144.0804. This is in accordance with literature data.

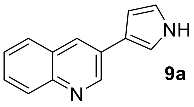

9a

3-(1H-pyrrol-3-yl)quinolone (9a)

^{1}H NMR (400 MHz, $CDCl_3$) δ 9.16 (d, J = 2.3 Hz, 1H), 8.59 (s, 1H), 8.24 – 8.14 (m, 1H), 8.07 (dq, J = 8.5, 0.9 Hz, 1H), 7.81 (ddt, J = 8.1, 1.4, 0.5 Hz, 1H), 7.63 (ddd, J = 8.4, 6.9, 1.5 Hz, 1H), 7.52 (ddd, J = 8.1, 6.9, 1.2 Hz, 1H), 7.29 (dt, J = 2.8, 1.8 Hz, 1H), 6.94 (td, J = 2.8, 2.0 Hz, 1H), 6.70 (td, J = 2.7, 1.6 Hz, 1H). ^{13}C NMR (101 MHz, $CDCl_3$) δ 149.42, 146.55, 129.80, 129.15, 128.97, 128.55,

128.21, 127.53, 126.72, 121.71, 119.59, 115.39, 106.70. IR (neat) 3168, 2924, 2854, 1602, 1489, 1339, 1165, 1083, 902, 789, 748 cm^{-1}. HRMS (ESI) calcd. for $C_{13}H_{11}N_2$ m/z $(M+H)^+$: 195.0917, found: 195.0913.

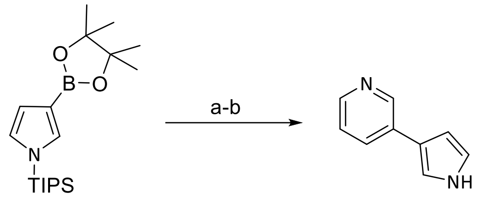

Reagents and conditions
a) 3-iodopyridine, $Pd(PPh_3)_4$, Na_2CO_3, $MeOH/H_2O/PhMe$ (1:1:5), 100 °C, 83%.
b) TBAF, THF, rt, 98%

Scheme S2. Synthesis of **8a**

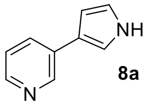

3-(1H-pyrrol-3-yl)pyridine (8a)[13]

^{1}H NMR (400 MHz, $CDCl_3$) δ=9.08 (s, 1H), 8.81 (dd, *J* = 2.3, 0.9 Hz, 1H), 8.40 (dd, *J* = 4.8, 1.6 Hz, 1H), 7.80 (ddd, *J* = 7.9, 2.3, 1.6 Hz, 1H), 7.30 – 7.22 (m, 1H), 7.15 (dt, *J* = 2.7, 1.8 Hz, 1H), 6.88 (td, *J* = 2.7, 1.9 Hz, 1H), 6.55 (td, *J* = 2.7, 1.6 Hz, 1H). ^{13}C NMR (101 MHz, $CDCl_3$) δ 146.6, 146.4, 132.4, 131.9, 123.6, 121.3, 119.5, 115.2, 106.3, 77.3. HRMS (ESI) calcd. for $C_9H_9N_2$ m/z $(M+H)^+$: 145.0760, found: 145.0754. This is in accordance with literature data.

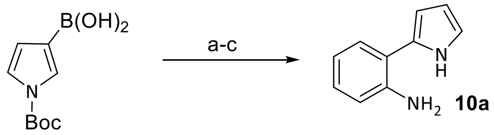

Reagents and conditions
a) 3-iodo-2-nitrobenzene, $Pd(OAc)_2$, SPhos, K_3PO_4, $Dioxane/H_2O$, 60 °C, 95%.
b) NaOMe, MeOH, 78 °C, 83%. c) Pd/C, MeOH, rt, 50%.

Scheme S3. Synthesis of **10a**

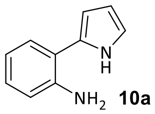

2-(1H-Pyrrol-2-yl)aniline (10a)[14]

^{1}H NMR (400 MHz, $CDCl_3$) δ=8.60 (s, 1H), 7.26 (dd, *J* = 7.7, 1.5 Hz, 1H), 7.10 (ddd, *J* = 7.9, 7.4, 1.6 Hz, 1H), 6.88 (td, *J* = 2.7, 1.5 Hz, 1H), 6.86 – 6.74 (m, 2H), 6.43 (ddd, *J* = 3.4, 2.6, 1.5 Hz, 1H), 6.33 (dt, *J* = 3.4, 2.6 Hz, 1H), 3.96 (s, 2H). ^{13}C NMR (101 MHz, $CDCl_3$) δ 143.4, 129.6, 128.5, 127.9, 119.7, 119.2, 118.0, 116.5, 109.4, 107.4. HRMS (ESI) calcd. for $C_{10}H_{11}N_2$ m/z $(M+H)^+$: 159.0917, found: 159.0911. This is in accordance with literature data.

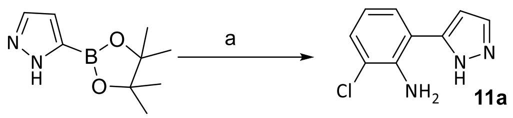

Reagents and conditions
a) 2-bromo-6-chloroaniline, Pd(dppf)Cl_2.CH_2Cl_2, Na_2CO_3, Dioxane/H_2O, Reflux, 30%.

Scheme S4. Synthesis of **11a**.

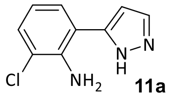
11a

2-chloro-6-(1-methyl-1H-pyrazole-3-yl)aniline (11a)

^{1}H NMR (400 MHz, $CDCl_3$) δ=7.62 (d, *J* = 2.5 Hz, 1H), 7.46 (dd, *J* = 7.8, 1.5 Hz, 1H), 7.24 (dd, *J* = 7.9, 1.5 Hz, 1H), 6.74 – 6.62 (m, 2H), 5.96 (s, 2H). ^{13}C NMR (101 MHz, $CDCl_3$) δ=151.4, 141.4, 129.3, 128.6, 126.8, 120.1, 117.3, 116.9, 103.6. IR (neat) 3426 3310 2924 1611 1458 1074 1045 747 cm^{-1}. HRMS (ESI) calcd. for $C_9H_9ClN_3$ m/z $(M+H)^+$: 194.0480, found: 194.0485.

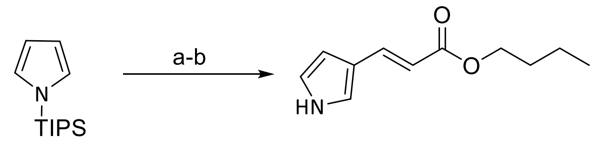

Reagents and conditions
a) butyl acrylate, Pd(OAc)$_2$, *tert*-Butyl hydroperoxide, AcOH/Dioxane/DMSO (3:9:1), 35 °C, 62%.
b) TBAF, THF, 0 °C to rt, 71%

Scheme S5. Synthesis of **14a** [Note: Substrates **13a** was prepared through similar sequences[15]].

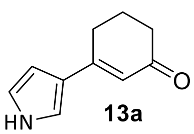
13a

3-(1H-pyrrol-3-yl)cyclohex-2-en-1-one (13a): ^{1}H NMR (400 MHz, Chloroform-*d*) δ 8.53 (s, 1H), 7.14 (dt, *J* = 2.9, 1.8 Hz, 1H), 6.87 – 6.81 (m, 1H), 6.51 (td, *J* = 2.8, 1.6 Hz, 1H), 6.33 (t, *J* = 1.4 Hz, 1H), 2.69 (td, *J* = 6.1, 1.4 Hz, 2H), 2.45 (dd, *J* = 7.4, 5.9 Hz, 2H), 2.19 – 2.03 (m, 2H). ^{13}C NMR (101 MHz, $CDCl_3$) δ 200.3, 155.5, 124.0, 120.6, 120.1, 118.8, 106.8, 37.6, 27.7, 22.8. IR (neat) 3206, 2957, 2600, 1619, 1578, 1505, 1437, 1351, 1306, 1266, 1248, 1194, 1165, 1135, 1083, 1006, 964, 883, 778, 659. HRMS *m/z* (ESI) cald for $C_{10}H_{12}NO$ ($[M+H]^+$) 162.0913, found: 162.0909.

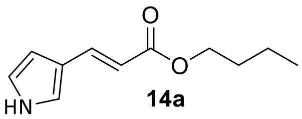
14a

butyl (*E*)-3-(1H-pyrrol-3-yl)acrylate (14a): ^{1}H NMR (400 MHz, Chloroform-*d*) δ 8.44 (s, 1H), 7.65 (d, *J* = 15.9 Hz, 1H), 7.03 (dt, *J* = 3.2, 1.7 Hz, 1H), 6.79 (q, *J* = 2.5 Hz, 1H), 6.46 (q, *J* = 2.5 Hz, 1H), 6.13 (d, *J* = 15.7 Hz, 1H), 4.17 (t, *J* = 6.7 Hz, 2H), 1.71 – 1.61 (m, 2H), 1.43 (dq, *J* = 14.6, 7.3 Hz, 2H), 0.95 (t, *J* = 7.4 Hz, 3H). ^{13}C NMR (101 MHz, $CDCl_3$) δ 168.4, 138.9, 121.5, 121.1, 120.1, 113.8, 106.8, 64.2, 31.1, 19.4, 14.0. IR (neat) 3273, 2959, 2871, 1670, 1622, 1543, 1477, 1445, 1398, 1357, 1302, 1282, 1263, 1211, 1155, 1083, 1065, 1046, 974, 860, 796, 764, 741, 673, 627. HRMS *m/z* (ESI) cald for $C_{11}H_{14}NO_2$ ($[M-H]^-$) 192.1030, found: 192.1038.

1.7.2 Characterization of chlorinated products.

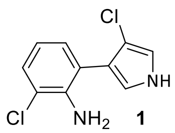

1

2-chloro-6-(4-chloro-1H-pyrrol-3-yl)aniline (1): ^{1}H NMR (400 MHz, Methylene Chloride-*d*$_2$) δ 8.48 (s, 1H), 7.21 (dd, *J* = 8.0, 1.5 Hz, 1H), 7.05 (dd, *J* = 7.6, 1.5 Hz, 1H), 6.91 – 6.85 (m, 1H), 6.83 (dd, *J* = 3.1, 2.3 Hz, 1H), 6.68 (t, *J* = 7.8 Hz, 1H), 4.24 (s, 2H). This is in accordance with synthetic standard data. SM loaded (0.050300 mmol, 9.7 mg), isolated yield: (0.023339 mmol, 5.3 mg), 46.4%.

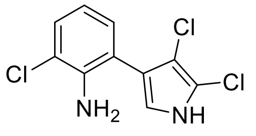

2Cl-1

2-chloro-6-(4,5-dichloro-1H-pyrrol-3-yl)aniline (2Cl-1): ^{1}H NMR (400 MHz, Methanol-*d*$_4$) δ 7.36 (dd, *J* = 8.0, 1.5 Hz, 1H), 7.18 (dd, *J* = 7.6, 1.5 Hz, 1H), 6.96 (s, 1H), 6.84 (t, *J* = 7.8 Hz, 1H), 4.76 (s, 2H). ^{13}C NMR (101 MHz, Methanol-*D*$_4$) δ 144.2, 132.0, 130.3, 122.6, 121.3, 121.1, 119.5, 117.8, 114.2, 109.8. HRMS *m/z* (ESI) cald for $C_{10}H_8Cl_3N_2$ ([M+H]$^+$) 260.9748 found: 260.9760. SM loaded (0.050300 mmol, 9.7 mg), Isolated yield: 0.5 mg (0.001988 mmol), 4.0%.

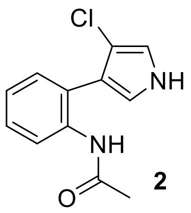

2

N-(2-(4-chloro-1H-pyrrol-3-yl)phenyl)acetamide (2): ^{1}H NMR (400 MHz, Methanol-*d*$_4$) δ 8.55 (s, 1H), 7.70 (d, *J* = 8.0 Hz, 1H), 7.31 (d, *J* = 6.8 Hz, 1H), 7.29 (t, *J* = 7.0 Hz, 1H), 7.19 (t, *J* = 7.5 Hz, 1H), 6.85 (d, *J* = 2.4 Hz, 1H), 6.79 (d, *J* = 2.3 Hz, 1H), 2.04 (s, 3H). ^{13}C NMR (101 MHz, Methanol-*D*$_4$) δ 172.1, 136.7, 132.5, 129.2, 128.3, 126.3, 125.8, 119.6, 118.7, 117.3, 112.1, 23.5. HRMS *m/z* (ESI) cald for $C_{12}H_{12}ClN_2O$ ([M+H]$^+$) 235.0633 found: 235.0637. SM loaded (0.04175 mmol, 8.4 mg), Isolated yield: 0.9 mg (0.003963 mmol), 9.5%.

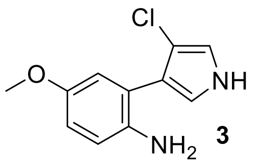

3

2-(4-chloro-1H-pyrrol-3-yl)-4-methoxyaniline (3): ^{1}H NMR (400 MHz, Methanol-*d*$_4$) δ 7.40 – 7.32 (m, 1H), 7.09 – 7.02 (m, 2H), 6.98 (d, *J* = 1.4 Hz, 2H), 3.88 (s, 3H). ^{13}C NMR (101 MHz, Methanol-*D*$_4$) δ 161.1, 131.7, 125.5, 123.3, 119.3, 119.1, 118.5, 117.5, 115.3, 112.1, 56.5. HRMS *m/z* (ESI) cald for $C_{11}H_{12}N_2OCl$ ([M+H]$^+$) 223.0633, found: 223.0657. SM loaded (0.032992 mmol, 6.2 mg), Isolated yield: 2.6 mg (0.011452 mmol), 34.7%.

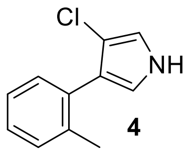

4

3-chloro-4-(o-tolyl)-1H-pyrrole (4): ^{1}H NMR (400 MHz, Methylene Chloride-d2) δ 8.36 (s, 1H), 7.31 – 7.14 (m, 4H), 6.85 (q, J = 2.8 Hz, 1H), 6.72 (q, J = 2.9 Hz, 1H), 2.28 (d, J = 2.8 Hz, 3H). ^{13}C NMR (101 MHz, CD_2Cl_2) δ 137.5, 133.2, 131.1, 130.0, 127.3, 125.3, 122.4, 117.0, 115.4, 112.1, 20.1. HRMS *m/z* (ESI) cald for $C_{11}H_{11}ClN$ ([M+H]$^+$) 192.0575, found: 192.0574. SM loaded (0.043889 mmol, 6.9 mg), Isolated yield: 1.1 mg (0.005948mmol), 13.6%.

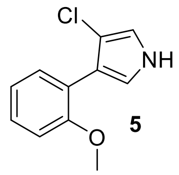

5

3-chloro-4-(2-methoxyphenyl)-1H-pyrrole (5): ^{1}H NMR (400 MHz, Methanol-d_4) δ 7.58 (ddd, *J* = 7.6, 1.8, 0.4 Hz, 1H), 7.44 – 7.35 (m, 1H), 7.21 – 7.14 (m, 1H), 7.11 (td, *J* = 7.5, 1.2 Hz, 1H), 7.04 (d, *J* = 2.4 Hz, 1H), 6.93 (d, *J* = 2.4 Hz, 1H), 3.97 (s, 3H). HRMS *m/z* (ESI) cald for $C_{11}H_{11}ClNO$ ([M+H]$^+$) 208.0524, found: 208.0523. SM loaded (0.009237mmol, 1.6 mg), Isolated yield: 0.43 mg (0.002071mmol), 22.4%. Due to the limited quantity of material, **5** was checked against and conforms with synthetic reference **5-Ref**.

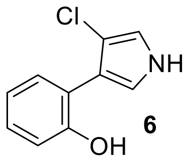

6

2-(4-chloro-1H-pyrrol-3-yl)phenol (6): ^{1}H NMR (400 MHz, Methanol-d4) δ 7.41 (dd, J = 7.6, 1.7 Hz, 1H), 7.11 (ddd, J = 8.1, 7.3, 1.7 Hz, 1H), 6.97 (d, J = 2.1 Hz, 1H), 6.88 (dd, J = 8.2, 1.2 Hz, 1H), 6.86 (td, J = 7.4, 1.3 Hz, 1H), 6.81 (d, J = 2.4 Hz, 1H). ^{13}C NMR (101 MHz, Methanol-D4) δ 155.9, 132.4, 128.7, 122.8, 120.6, 119.6, 118.6, 116.9, 116.7, 112.2, 49.3. HRMS *m/z* (ESI) cald for $C_{10}H_9ClNO$ ([M+H]$^+$) 194.0367, found: 194.0365. SM loaded (0.031284 mmol, 5.0 mg), Isolated yield: 1.0 mg (0.005319 mmol), 17.0%.

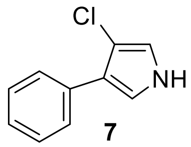

7

3-chloro-4-phenyl-1H-pyrrole (7): ^{1}H NMR (400 MHz, Methylene Chloride-d_2) δ 8.42 (s, 1H), 7.61 – 7.54 (m, 2H), 7.41 – 7.33 (m, 2H), 7.29 – 7.22 (m, 1H), 6.95 – 6.89 (m, 1H), 6.85 (t, *J* = 2.6 Hz, 1H). ^{13}C NMR (101 MHz, CD_2Cl2) δ 133.9, 128.4, 128.4, 127.6, 127.6, 126.4, 122.4, 116.7, 116.3, 110.6. HRMS *m/z* (ESI) cald for $C_{10}H_9ClN$ ([M+H]$^+$) 178.0418, found: 178.0417. SM loaded (0.034779 mmol, 5.0 mg), Isolated yield: 3.2 mg (0.018184 mmol), 52.3%.

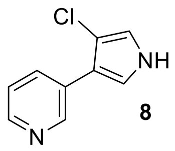

8

3-(4-chloro-1H-pyrrol-3-yl)pyridine (8): ^{1}H NMR (400 MHz, Methanol-d_4) δ 8.92 (dd, *J* = 2.3, 0.9 Hz, 1H), 8.55 (dd, *J* = 4.9, 1.6 Hz, 1H), 8.23 (ddd, *J* = 8.0, 2.3, 1.6 Hz, 1H), 7.60 (ddd, *J* = 8.0, 4.9, 0.9 Hz, 1H), 7.23 (d, *J* = 2.3 Hz, 1H), 7.04 (d, *J* = 2.4 Hz, 1H). ^{13}C NMR (101 MHz, Methanol-d_4) δ 149.2, 147.8, 137.4, 133.6, 125.9, 119.7, 119.3, 118.9, 111.6, 49.9. HRMS *m/z* (ESI) cald for $C_9H_8N_2Cl$ [M+H]$^+$ 179.0371, found: 179.0390. SM loaded (0.028160 mmol, 4.1 mg), Isolated yield: 1.6 mg (0.009182 mmol), 32.6%.

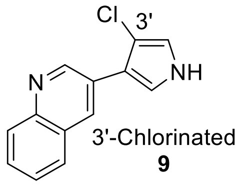

3'-Chlorinated
9

3-(4-chloro-1H-pyrrol-3-yl)quinoline (3'-Cl-9): ^{1}H NMR (400 MHz, Methylene Chloride-d_2) δ 9.10 (d, *J* = 2.3 Hz, 1H), 8.77 (s, 2H), 8.43 (d, *J* = 2.3 Hz, 1H), 8.07 (d, *J* = 8.5 Hz, 1H), 7.87 (dd, *J* = 8.2, 1.4 Hz, 1H), 7.68 (ddd, *J* = 8.5, 6.9, 1.5 Hz, 1H), 7.56 (ddd, *J* = 8.1, 6.8, 1.3 Hz, 1H), 7.13 (t, *J* = 2.8 Hz, 1H), 6.95 (t, *J* = 2.6 Hz, 1H). ^{3}C NMR (101 MHz, CD_2Cl2) δ 151.0, 147.2, 133.2, 129.6, 129.3, 128.6, 128.4, 127.7, 127.3, 119.6, 117.8, 117.4, 111.5. HRMS *m/z* (ESI) cald for $C_{13}H_{10}ClN_2$ ([M+H]$^+$) 229.0527, found: 229.0531. SM loaded (0.077225 mmol, 15.0 mg), Isolated yield: 3.2 mg (0.013993 mmol), 18.1%.

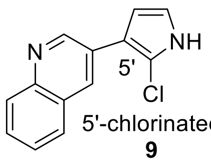

5'-chlorinated **9**

3-(2-chloro-1H-pyrrol-3-yl)quinoline (5'-Cl-9): ^{1}H NMR (400 MHz, Methylene Chloride-d_2) δ 9.19 (d, *J* = 2.3 Hz, 1H), 8.65 (s, 2H), 8.38 (dd, *J* = 2.4, 0.8 Hz, 1H), 8.06 (dq, *J* = 8.5, 1.0 Hz, 1H), 7.90 – 7.83 (m, 1H), 7.68 (ddd, *J* = 8.4, 6.9, 1.5 Hz, 1H), 7.56 (ddd, *J* = 8.1, 6.9, 1.3 Hz, 1H), 6.86 (dd, *J* = 3.3, 2.7 Hz, 1H), 6.59 (t, *J* = 3.1 Hz, 1H). ^{13}C NMR (101 MHz, CD_2Cl2) δ 150.6, 147.1, 132.7, 129.6, 129.3, 128.7, 128.3, 128.1, 127.3, 118.3, 117.2, 112.5, 109.6. HRMS *m/z* (ESI) cald for $C_{13}H_{10}ClN_2$ ($[M+H]^+$) 229.0527, found: 229.0541. Isolated yield: 2.2 mg (0.009620 mmol), 12.5%.

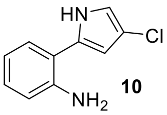

10

2-(4-chloro-1H-pyrrol-2-yl)aniline (10): ^{1}H NMR (400 MHz, Methanol-d_4) δ 7.34 – 7.25 (m, 2H), 6.99 (dd, *J* = 8.5, 1.2 Hz, 1H), 6.95 (d, *J* = 3.0 Hz, 1H), 6.91 (td, *J* = 7.6, 1.2 Hz, 1H), 6.32 (d, *J* = 3.0 Hz, 1H), 5.67 (s, 1H), 4.74 (s, 2H). ^{13}C NMR (101 MHz, Methanol-D_4) δ 147.7, 133.0, 130.9, 127.3, 119.8, 119.7, 119.5, 117.9, 111.5, 110.5. HRMS *m/z* (ESI) cald for $C_{10}H_{10}ClN_2$ ($[M+H]^+$) 193.0527, found: 193.0535. SM loaded (0.030025 mmol, 4.75 mg), Isolated yield: 2.7 mg (0.014015 mmol), 46.7%.

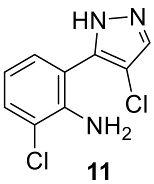

11

2-chloro-6-(4-chloro-1H-pyrazol-5-yl)aniline (11): ^{1}H NMR (400 MHz, Methylene Chloride-d_2) δ 7.76 (s, 1H), 7.59 (dd, *J* = 7.8, 1.5 Hz, 1H), 7.33 (dd, *J* = 7.9, 1.4 Hz, 1H), 6.77 (t, *J* = 7.8 Hz, 1H). ^{13}C NMR (101 MHz, CD_2Cl_2) δ 144.5, 142.0, 130.2, 129.6, 128.6, 119.8, 117.1, 115.7, 109.1. HRMS *m/z* (ESI) cald for $C_9H_8Cl_2N_3$ ($[M+H]^+$) 228.0090, found: 228.0098. SM loaded (0.035634 mmol, 6.9 mg), Isolated yield: 1.2 mg (0.005393 mmol), 15.1%.

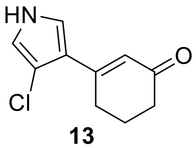

13

3-(4-chloro-1H-pyrrol-3-yl)cyclohex-2-en-1-one (13): ^{1}H NMR (400 MHz, Chloroform-*d*) δ 8.59 (s, 1H), 7.00 (dd, *J* = 3.3, 2.3 Hz, 1H), 6.83 (dd, *J* = 2.8, 2.4 Hz, 1H), 6.76 (t, *J* = 1.4 Hz, 1H), 2.69 (td, *J* = 6.1, 1.4 Hz, 2H), 2.45 (dd, *J* = 7.4, 5.9 Hz, 2H), 2.14 – 2.06 (m, 2H). ^{13}C NMR (101 MHz, $CDCl_3$) δ 200.5, 153.6, 123.3, 120.4, 119.1, 118.5, 111.8, 37.4, 28.6, 22.8. (ESI) cald for $C_{10}H_{11}ClNO$ ($[M-H]^-$) 194.0378, found: 194.0406. SM loaded (0.038647 mmol, 6.2 mg), Isolated yield: 2.8 mg (0.014465 mmol), 37.4%.

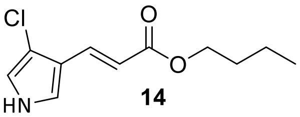

14

butyl (E)-3-(4-chloro-1H-pyrrol-3-yl)acrylate (14): ^{1}H NMR (400 MHz, Chloroform-*d*) δ 7.61 (d, *J* = 16.1 Hz, 1H), 7.01 (t, *J* = 2.8 Hz, 1H), 6.78 (t, *J* = 2.5 Hz, 1H), 6.47 (d, *J* = 16.1 Hz, 1H), 4.18 (t, *J* = 6.7 Hz, 2H), 1.68 (ddt, *J* = 8.8, 7.9, 6.5 Hz, 2H), 1.48 – 1.38 (m, 2H), 0.96 (t, *J* = 7.4 Hz, 3H). ^{13}C NMR (101 MHz, $CDCl_3$) δ 168.1, 136.0, 120.0, 118.0, 117.4, 115.5, 113.2, 64.4, 31.1, 19.4, 14.0. HRMS *m/z* (ESI) cald for $C_{11}H_{15}ClNO_2$ ($[M+H]^+$) 228.0786, found: 228.0809. SM loaded (0.046055 mmol, 8.9 mg), Isolated yield: 1.3 mg (0.005710mmol), 12.4%.

1.7.3 Synthesis of substrate 17a and chlorinated Fludioxonil analog 17

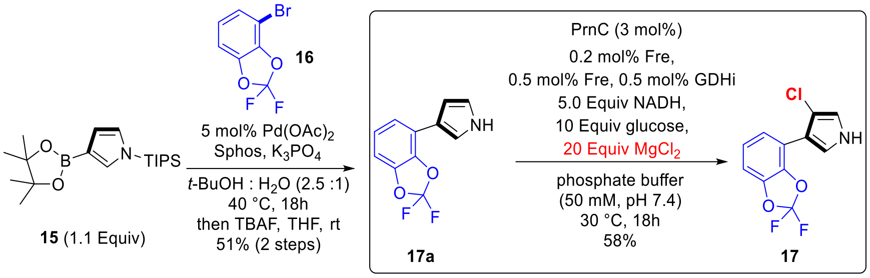

4-bromo-2,2-difluorobenzo[*d*][1,3]dioxole (**16**) (237 mg, 0.14 mL, 1 mmol), *N*-TIPS pyrrole-3-boronic acid pinacol ester (**15**) (384 mg, 1.1 mmol, 1.1 eq), Palladium acetate (12 mg, 5 mol%), SPhos (33 mg, 8 mol%) and K_3PO_4 (0.38 g, 1.8 mmol) were added to a schlenk tube containing degassed *n*-BuOH : H_2O (2.5 :1, 3 ml). The mixture was stirred at 40 °C for 18 hours. Upon complete conversion of starting material by TLC, water (5 mL) was added to the mixture and extracted with (5 x 3mL) of ethyl acetate. The organic layers were combined, filtered through a plug of celite and dried over anhydrous sodium sulphate. Concentration over reduced pressure afforded a crude colorless oil (**TIPS-17a**) (341 mg, 90%) which was used directly in the next step without purification. 1H NMR (400 MHz, $CDCl_3$) δ 7.30 – 7.26 (m, 2H), 7.03 (t, *J* = 8.0, 1H), 6.88 – 6.77 (m, 2H), 6.77 – 6.66 (m, 1H), 1.54 – 1.45 (m, 3H), 1.14 (d, *J* = 7.5, 18H).

3-(2,2-difluorobenzo[d][1,3]dioxol-4-yl)-1-(triisopropylsilyl)-1H-pyrrole (**TIPS-17a**) (341 mg, 0.9 mmol) was dissolved in THF (5.0 ml) under nitrogen. TBAF (1.0 M in THF, 1.6 ml, 1.8 eq) was added and the mixture was stirred at room temperature for 1 hour. Upon complete conversion of starting material by TLC, the mixture was evaporated to dryness. The resultant residue was directly subjected to purification by silica chromatography. Side product silanol and unreacted starting material were removed by 10:1 of (hexane: ethyl acetate) and the product **17a** (113 mg, 57%) was eluted using 3:1 of (hexane: ethyl acetate) as a red oil. ^{1}H NMR (400 MHz, $CDCl_3$) δ 8.41 (s, 1H), 7.34 (dt, *J* = 2.8, 1.8 Hz, 1H), 7.29 (dd, *J* = 8.2, 1.2 Hz, 1H), 7.05 (t, *J* = 8.0 Hz, 1H), 6.92 – 6.81 (m, 2H), 6.66 (td, *J* = 2.8, 1.6 Hz, 1H). ^{13}C NMR (101 MHz, $CDCl_3$) δ 144.0, 139.6, 131.5 ($^1J_{C\text{-}F}$ = 254.5 Hz), 123.6, 120.6, 119.4, 118.9, 117.6, 117.4, 106.9, 105.9. ^{19}F NMR (376 MHz, $CDCl_3$) δ -49.7. IR (neat) 3410 3392 1654 1453 1231 1130 1082 880 767 723 712 cm^{-1}. HRMS (ESI) calcd. for $C_{11}H_8F_2NO_2$ m/z $(M+H)^+$: 224.0518, found: 224.0514.

3-chloro-4-(2,2-difluorobenzo[d][1,3]dioxol-4-yl)-1H-pyrrole (17): In a solution containing **17a** (0.5 mM), $MgCl_2$ (10 mM), glucose (5.0 mM), FAD (1.0 µM), NT-11 PrnC (12.5 µM), Fre (2.5 µM) and Gdhi (2.5 µM) in 50mM potassium phosphate buffer, NADH (2.5 mM) was added to a total volume of 8.0 mL. After an overnight incubation of 30°C and orbital shaking at 350 rpm, reactions were quenched with an equivalent volume of MeOH, pelleted by centrifugation (15000 rpm for 10 min) and the supernatant extracted with (3 x 10mL) of ethyl acetate. The precipitated solids were vortex, sonicated and extracted with additional (3 x 10mL) of MeOH. The combined organic layers were filtered through a plug of celite and dried over

anhydrous sodium sulphate. Concentration over reduced pressure afforded a crude brown oil which was subjected over preparatory thin-layer chromatography using 3:1 of (hexane: ethyl acetate) to afford (0.6 mg, 58%) of **16** as a reddish-brown oil. ^{1}H NMR (400 MHz, Methanol-d_4) δ 7.57 (dd, J = 8.1, 1.2 Hz, 1H), 7.14 (t, J = 8.1 Hz, 1H), 7.09 (d, J = 2.3 Hz, 1H), 7.02 (dd, J = 8.0, 1.1 Hz, 1H), 6.86 (d, J = 2.3 Hz, 1H). ^{13}C NMR (101 MHz, Methanol-D_4) δ 144.9, 141.4, 132.7 ($^{1}J_{C-F}$ = 251.6 Hz), 124.7, 124.3, 119.9, 119.6, 118.2, 114.4, 111.0, 107.8. ^{19}F NMR (376 MHz, MeOD) δ -52.0. HRMS *m/z* (ESI) cald for $C_{11}H_5ClF_2NO_2$ ($[M-H]^-$) 255.9982, found: 255.9989.

1.8 Supplementary Tables and Figures

Table S1. Comparative kinetics of PrnC in biocatalytic chlorination with other related FDHs.

Enzyme	Substrate	K_{cat} (min^{-1})	K_m (μM)	K_m/K_{cat} ($μM^{-1}$ min^{-1})
PrnC	**1**	0.456 ± 0.005	15.8 ± 0.7	0.029 ± 0.002
PrnA[16]	L-Tryptophan	0.093 ± 0.03	160 ± 40	0.0006 ± 0.0003
RebH[17]	L-Tryptophan	1.4	2.0	0.7
PyrH[18]	L-Tryptophan	3.56 ± 1.1	109 ± 44	0.0325 ± 0.017

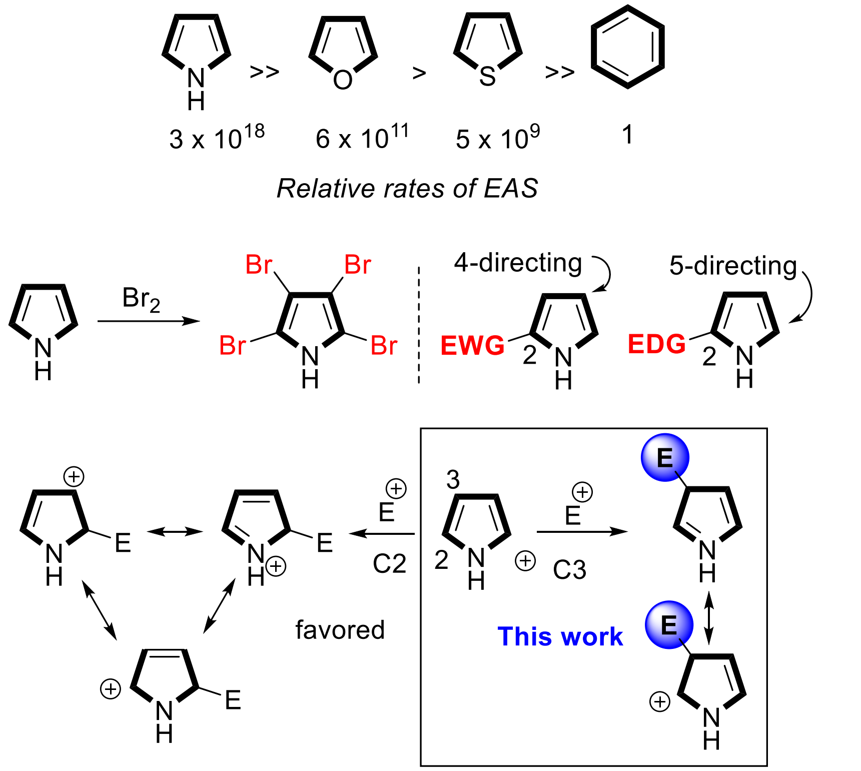

Figure S1. Relative rates of electrophilic aromatic substitution (EAS) in heterocycles and preferred C-2 site of halogenation for pyrroles.

1.8.1 Gels – labelled and annotated

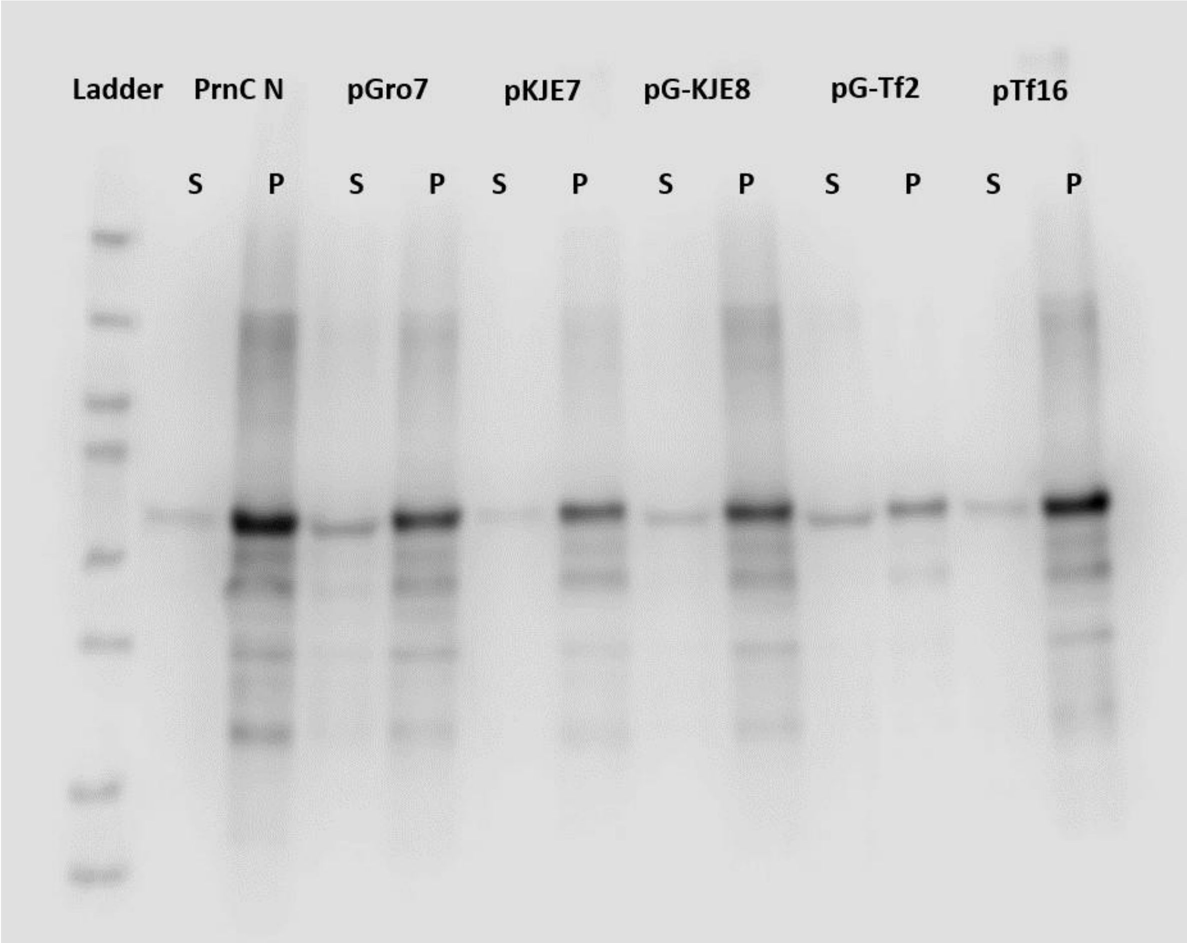

Figure S2a. Western blot of 6x N-His PrnC co-expressed with chaperone proteins; 64 kDa overexpressed PrnC protein bands observed in supernatant and pellet fractions following His-tag HRP antibody labelling and staining.

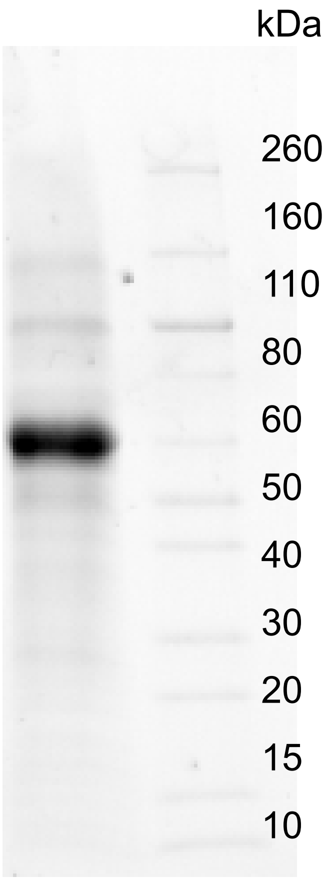

Figure S2b. Purified NT11-PrnC (left, 60 kDa) as imaged on a Biorad Stain-free Precast gel against Novex Sharp unstained standard.

1.8.2 HPLC traces and Charts/graphs

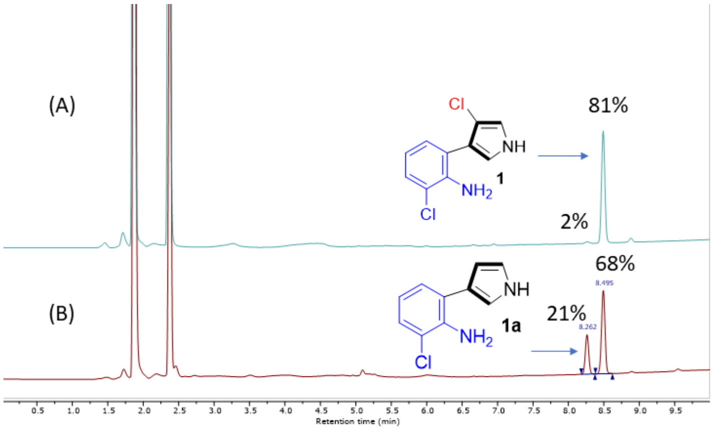

Figure S3a. Overlayed HPLC chromatograms of NT-11 tagged PrnC (A) against native un-tagged PrnC (B) under the standard optimized conditions. Both enzymes have a C-terminal His-tag. Conversion (%) was determined at 254 nm against calibrated standards.

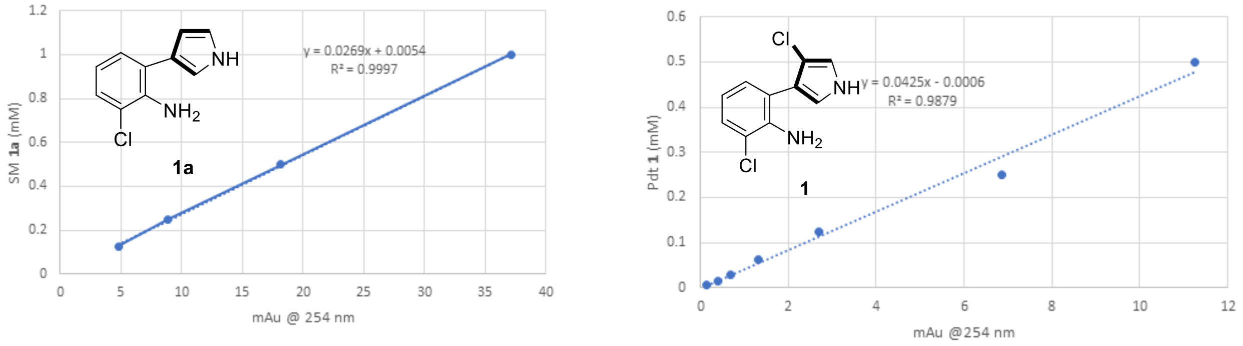

Figure S3b. Calibration Curves of MDA (**1a**) and MDACl (**1**).

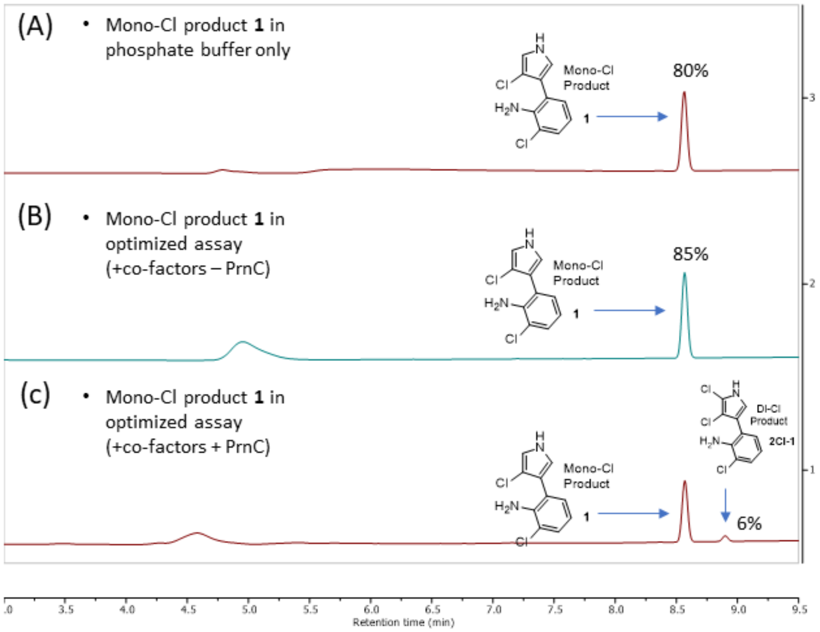

Figure S3c. Overlayed HPLC chromatograms (A) = **1** in pH 7 phosphate buffer only, (B) **1** in standard optimized conditions (SOC) without PrnC, (C) = SOC with PrnC, *ca*. 6% of di-chlorinated product **2Cl-1**.

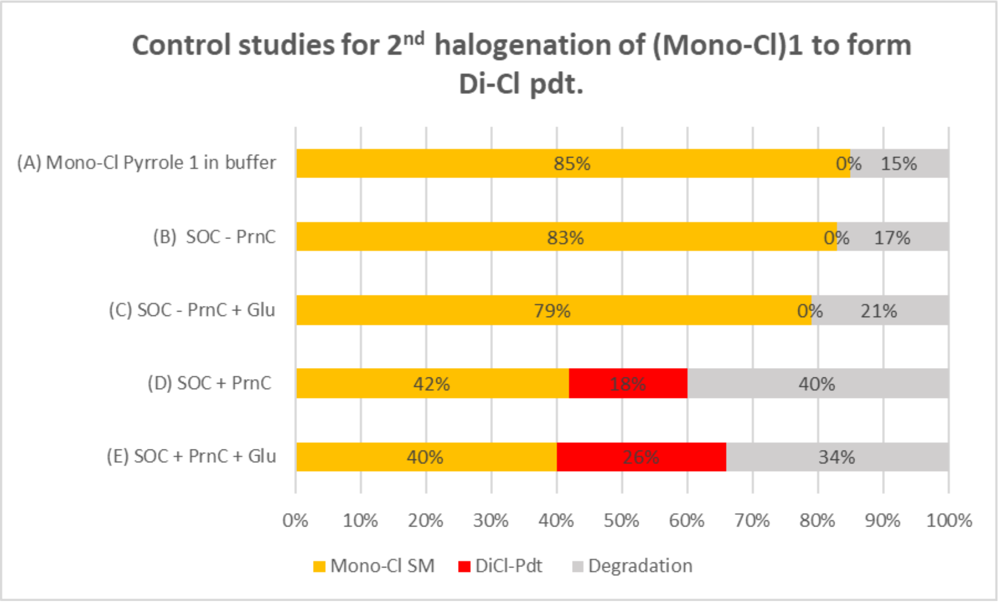

Figure S3d. Mass balance of Mono-chlorinated Product **1** (Orange), Di-chlorinated Product **2Cl-1** (red) and Degraded materials (grey) at 18h. Glu = Glutathione (1 Equiv). (A) = **1** in pH 7 phosphate buffer only, (B) = standard optimized conditions (SOC), (C) = SOC– PrnC + Glu, (D) = SOC + PrnC, (E) = SOC + PrnC + Glu.

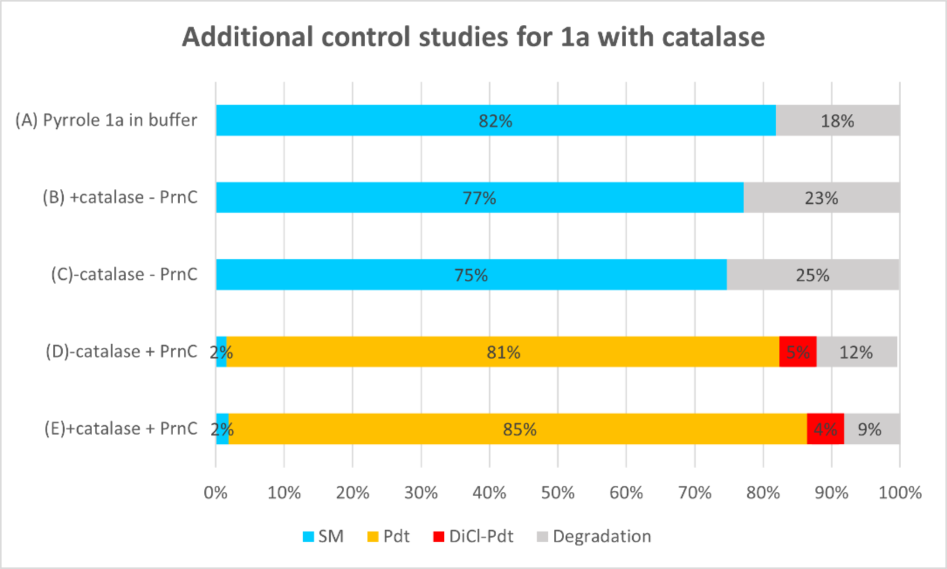

Figure S3e. Mass balance of Pyrrolic Starting material **1a** (light blue), Mono-chlorinated Product **1** (Orange), Di-chlorinated Product **2Cl-1** (red) and Degraded materials (grey) at 18h. (A) = **1a** in pH 7 phosphate buffer only, (B) = standard optimized conditions (SOC) + catalase – PrnC, (C) = SOC -catalase – PrnC, (D) = SOC -catalase + PrnC, (E) = SOC +catalase + PrnC.

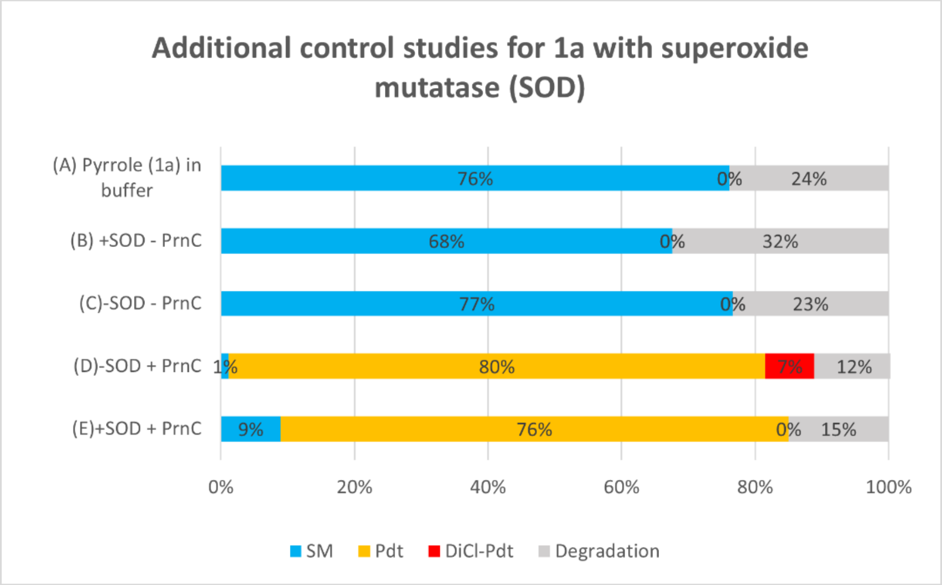

Figure S3f. Mass balance of Pyrrolic Starting material 1a (light blue), Mono-chlorinated Product **1** (Orange), Di-chlorinated Product **2Cl-1** (red) and Degraded materials (grey) at 18h. SOD = superoxide dismutase [25 mM]. (A) = **1a** in pH 7 phosphate buffer only, (B) = standard optimized conditions (SOC) + SOD – PrnC, (C) = SOC -SOD – PrnC, (D) = SOC -SOD + PrnC, (E) = SOC +SOD + PrnC.

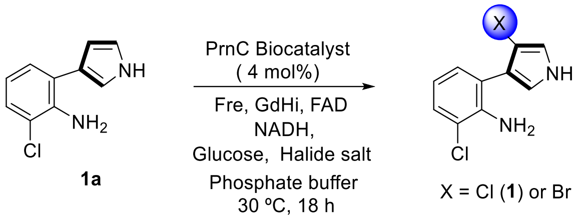

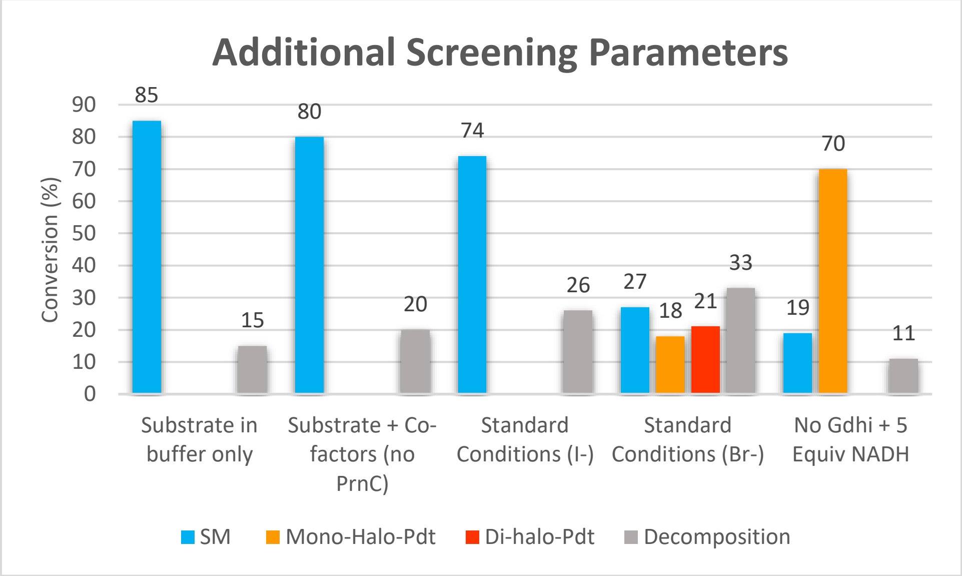

Figure S3g. Additional co-factors optimization parameters on **1a**. Standard optimized conditions: Substrate (0.5 mM), PrnC Biocatalyst (4 mol%), GdHi (0.5 mol%), FAD (0.2 mol%), Fre (0.5 mol%), NADH (5.0 equiv), Glucose (10.0 equiv), $MgCl_2$/$MgBr_2$/NaI (20.0 equiv), phosphate buffer (10 mM, pH 7.4). Chlorinated conversion determined by calibration curve. *Brominated conversions are determined by comparison of the LC peak areas between crude reaction mixtures relative to control standard. Mixture of (~1:1) of Mono-brominated and di-brominated products.

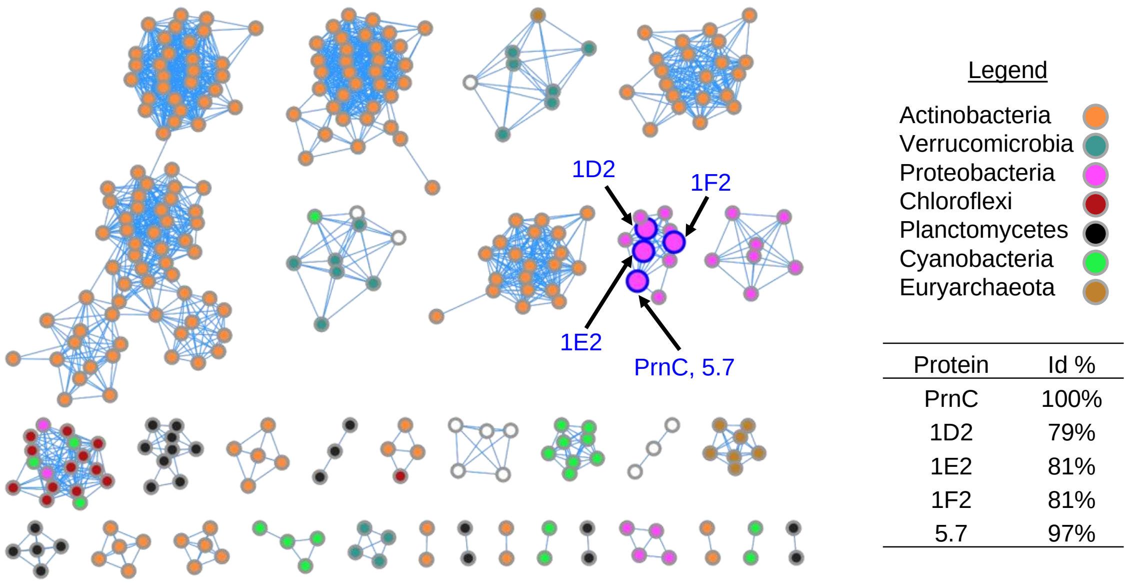

Protein	Id %
PrnC	100%
1D2	79%
1E2	81%
1F2	81%
5.7	97%

Figure S4a. Sequence Similarity Network (SSN) of PrnC and its homologs. A protein sequence identity threshold of 60% was applied to filter out irrelevant proteins from UniprotKB's protein database. Proteins that were >80% identical to each other were grouped into a single node to reduce network complexity. PrnC protein (Uniprot: P95482) shared a node with homolog 5.7 (Uniprot: I1V4X2), while the other selected homologs, 1D2, 1E2, and 1F2 (Uniprot: A0A250ITU6, Q9RPG1, A0A150PA41), were displayed as individual nodes. All homologs belonged to the same proteobacteria (purple) and their sequence identity (%) to PrnC are also indicated. Nodes are colored by phylum.

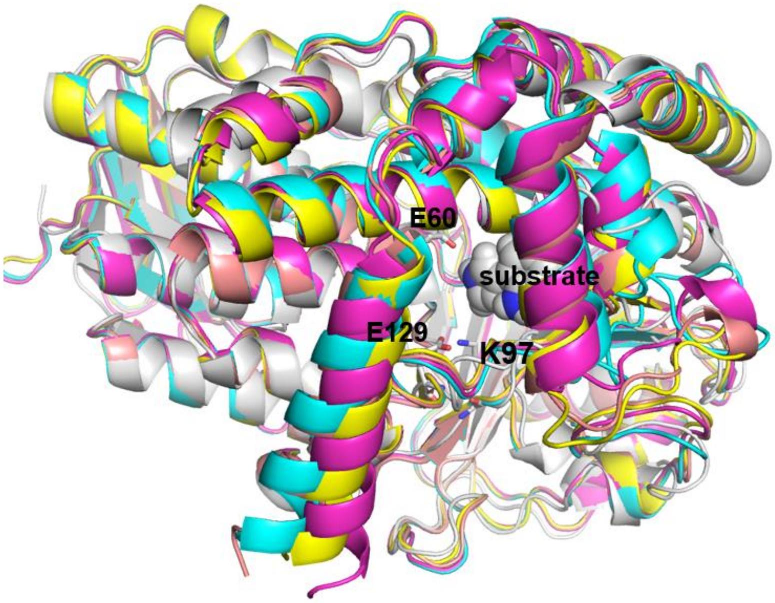

Figure S4b. The structural alignment between PrnC and four homologs (5.7, 1E2, 1D2, and 1F2). (A) the grey, pink, magenta, cyan, and yellow colours stand for PrnC, 5.7, 1E2, 1D2, and 1F2, respectively; (B) overall structures are very similar and share the same key residues K97, E129, and E60 in PrnC; (C) the substrate modelled in PrnC is accommodated in the four homologs.

PrnA sequence identity matrix

	1E2	1F2	prnC	5.7
1: 1E2__AAD46369.1	100.00	48.10	47.64	47.83
2: 1F2__KYF52561.1	48.10	100.00	71.40	71.40
3: prnC_AAB97504.1	47.64	71.40	100.00	95.35
4: 5.7__AFI26231.1	47.83	71.40	95.35	100.00

PrnB sequence identity matrix

	prnC	5.7	1E2	1F2	1D2
1: prnC_AAB97505.1	100.00	89.75	62.75	62.18	61.62
2: 5.7__AFI26232.1	89.75	100.00	61.90	61.34	61.90
3: 1E2__AAD46366.1	62.75	61.90	100.00	64.15	67.57
4: 1F2__KYF52560.1	62.18	61.34	64.15	100.00	68.07
5: 1D2__ATB34570.1	61.62	61.90	67.57	68.07	100.00

PrnC sequence identity matrix

	1E2	prnC	5.7
1: 1E2__AAD46367.1	100.00	79.47	78.76
2: prnC_AAB97506.1	79.47	100.00	96.65
3: 5.7__AFI26233.1	78.76	96.65	100.00

PrnD sequence identity matrix

	1E2	prnC	5.7
1: 1E2__AAD46368.1	100.00	63.71	64.82
2: prnC_AAB97507.1	63.71	100.00	92.84
3: 5.7__AFI26234.1	64.82	92.84	100.00

Figure S4c. Sequence identity matrices for PrnA, PrnB, PrnC, and PrnD. All the protein sequences for PrnA, PrnB, PrnC, and PrnD are retrieved according to the corresponding genes of homologs.

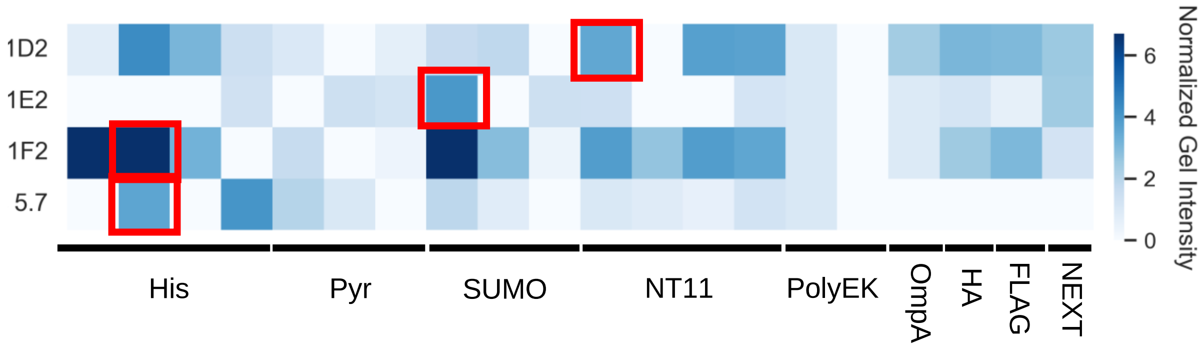

Figure S4d. Heatmap of soluble expression of PrnC homologs. Protein expression was quantified by gel electrophoresis and after accounting for protein size differences, normalized to the first PolyEK tagged protein. Across all the homologs, His, SUMO and NT11 tags performed the best. The red boxes indicated the constructs that were used for activity assays.

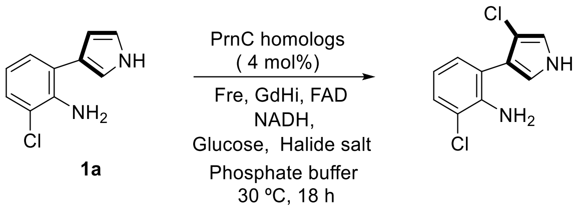

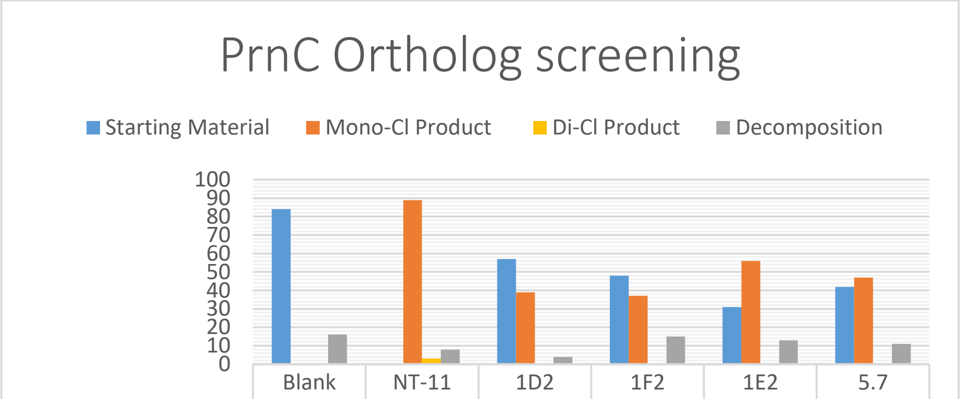

	Blank	NT-11	1D2	1F2	1E2	5.7
Starting Material	84	0	57	48	31	42
Mono-Cl Product	0	89	39	37	56	47
Di-Cl Product	0	3	0	0	0	0
Decomposition	16	8	4	15	13	11

Figure S4e. Conditions: Substrate (0.5 mM), PrnC Biocatalyst (4 mol%), GdHi (0.5 mol%), FAD (0.2 mol%), Fre (0.5 mol%), NADH (5.0 equiv), Glucose (10.0 equiv), $MgCl_2$(20.0 equiv), phosphate buffer (10 mM, pH 7.4). Average of duplicate runs. Conversion determined by calibration curve of product and starting material standards.

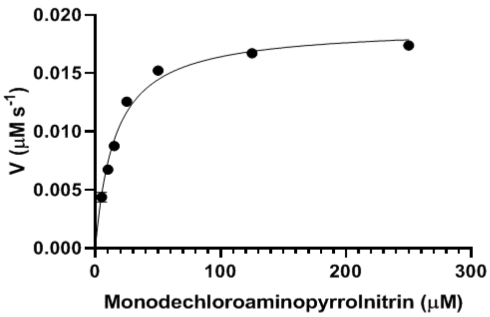

**Kinetic parameters**	**Value**
k_{cat} (s^{-1})	$7.60 \pm 0.08 \times 10^{-3}$
K_m (M)	$1.58 \pm 0.07 \times 10^{-5}$
k_{cat}/K_m (s^{-1} M^{-1})	$4.83 \pm 0.24 \times 10^{2}$

Figure S5. Michaelis-Menten plot and table of kinetic parameters for PrnC chlorination of monodechloroaminopyrrolnitrin (**1a**).

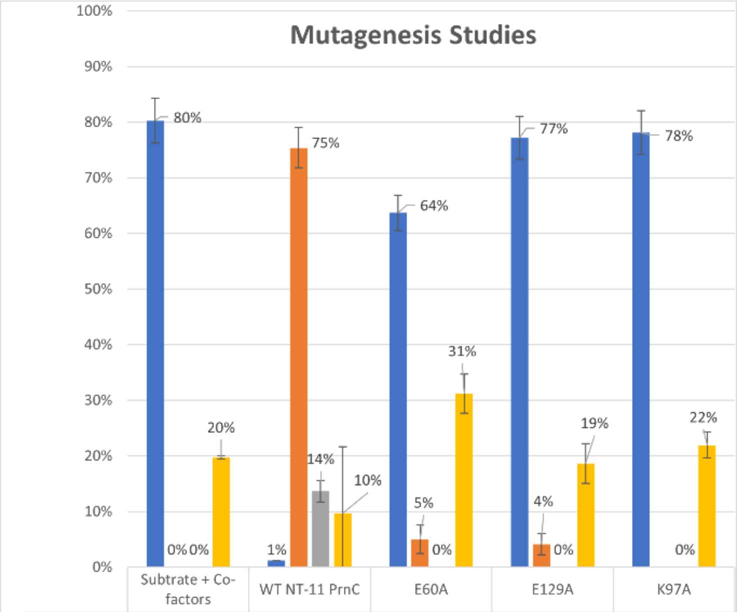

	Subtrate + Co-factors	WT NT-11 PrnC	E60A	E129A	K97A
SM (mM)	80%	1%	64%	77%	78%
Pdt (mM)	0%	75%	5%	4%	0%
di-pdt (mM)	0%	14%	0%	0%	0%
Degradation	20%	10%	31%	19%	22%

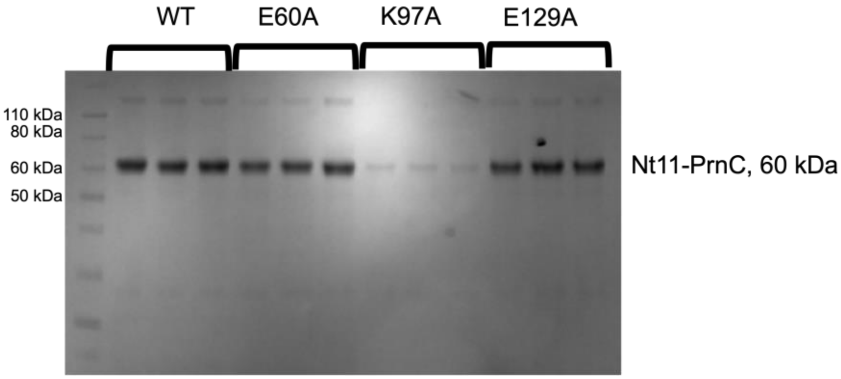

Figure S6. Top: Chart and table of MDA-Cl (**1**) product formation breakdown by PrnC enzyme variants. Bottom: SDS-PAGE analysis of NT11-PrnC and its mutants. Eluates from his-tagged purification were loaded onto a 4-12% NuPAGE Bis-Tris mini protein gel (Invitrogen), and the bands obtained were referenced to the Novex Sharp Unstained Protein Standard (Invitrogen). The protein purifications were done with biological triplicates.

MDA
(**1a**)

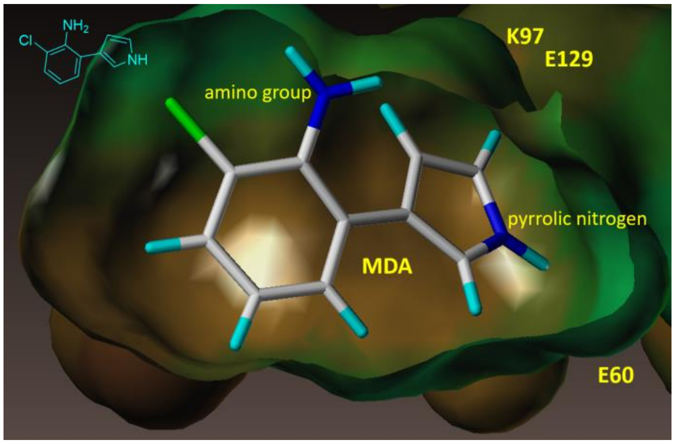

Figure S7a. The native substrate MDA (**1a**) in the binding pocket. Brown color refers to the hydrophobic residues; green color standing for the polar residues.

```
PrnC   13   NHFDVIILGSGMSGTQMGAILAKQQFRVLIIEESSHPRFTIGESSIPETSLMNRIIADRY  72
6BZA   22   NQYDVIIIGSGIAGALTGAVLAKSGLNVLILDSAQHPRFSVGEAATPESGFLLRLLSKRF  81

PrnC   73   GIPELDHITSFYSTQRYVASST-GIKRNFGFVFHKPGQEHDPKEFTQCVIPELPWGPESH  131
6BZA   82   DIPEIAYLSHPDKIIQHVGSSACGIKLGFSFAWH---QENAPSSPDHLVAPPLKV-PEAH  137

PrnC   132  YYRQDVDAYLLQAAIKYGCKVHQKTTVTEYHADKDGVAVTTAQGERFTGRYMIDCGGPRA  191
6BZA   138  LFRQDIDYFALMIALKHGAESRQNIKIESISLNDDGVEVALSNAAPVKAAFIIDAAAQGS  197

PrnC   192  PLATKFKLREEPCRFKTHSRSLYTHMLGVKPFDDIFKVKGQR---WRWHEGTLHHMFEGG  248
6BZA   198  PLSRQLGLRTTEG-LATDTCSFFTHMLNVKSYEDALAPLSRTRSPIELFKSTLHHIFEEG  256

PrnC   249  WLWVIPFNNHPRSTNNLVSVGLQLDPRVYPKTDISAQQEFDEFLARFPSIGAQFRDAVPV  308
6BZA   257  WLWVIPFNNHPQGTNQLCSIGFQFNNAKYRPTE-APEIEFRKLLKKYPAIGEHFKDAVNA  315

PrnC   309  RDWVKTDRLQFSSNACVGDRYCLMLHANGFIDPLFSRGLENTAVTIHALAARLIKALRDD  368
6BZA   316  REWIYAPRINYRSVQNVGDRFCLLPQATGFIDPLFSRGLITTFESILRLAPKVLDAARSN  375

PrnC   369  DFSPERFEYIERLQQKLLDHNDDFVSCCYTAFSDFRLWDAFHRLWAVGTILGQFRLVQAH  428
6BZA   376  RWQREQFIEVERHCLNAVATNDQLVSCSYEAFSDFHLWNVWHRVWLSGSNLGSAFLQKLL  435

PrnC   429  ARFRASRNEGDLDHLDNDPPYLGYLCADMEEYYQLFNDAKAEVEAVSAGRKPADEAAARI  488
6BZA   436  HDLEHSGDARQFDAALEAVRFPGCLSLDSPAYESLFRQSCQVMQQAREQARPVAETANAL  495

PrnC   489  HALIDERD  496
6BZA   496  HELIKEHE  503
```

Figure S7b. The sequence alignment and the template crystal structure of halogenase PltM (PDBCODE: 6BZA) were provided by BLASTP on the NCBI server.

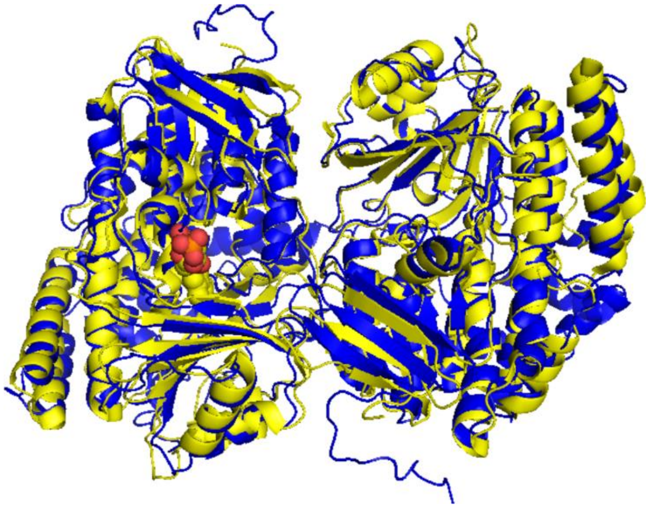

Figure S7c. Crystal structure of Pseudomonas protegens PltM (yellow, PDB 6BZA, the bound FAD cofactor is rendered in spheres), overlayed with the AlphaFold multimer model of PrnC (blue), suggesting a conserved dimer interface.

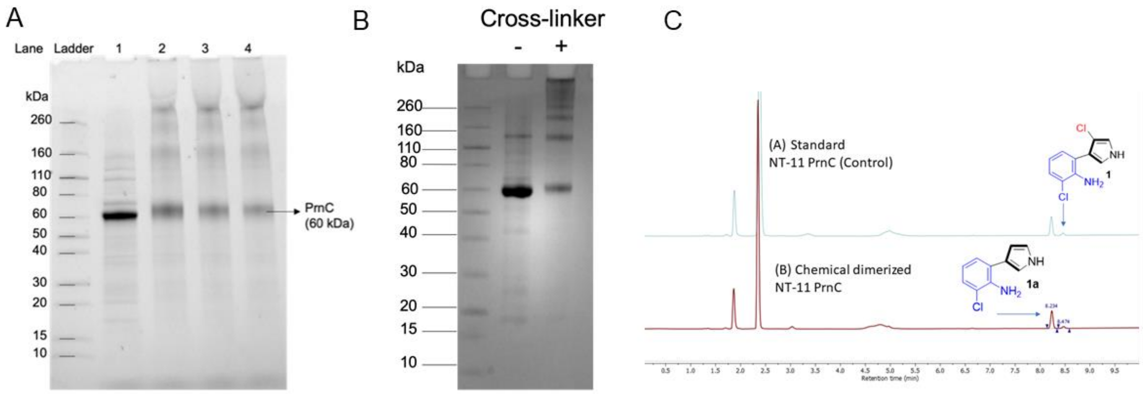

Figure S8. Chemical dimerization of NT11-PrnC in solution. (A) Upon addition of PEGylated bis(sulfosuccinimidyl)suberate (BS(PEG)5, Thermo Scientific^{TM} 21581), appearance of high molecular bands are observed, suggesting that NT11-PrnC exists as a dimer or multi-mer in solution. NT11-PrnC (20 µM) was incubated in 1 mM BS(PEG)5 and PBS, for 10 mins (Lane 2), 20 mins (Lane 3) and 30 mins (Lane 4) at room temperature. Lane 1 represents NT11-PrnC incubated with only PBS for 30 mins. (B) SDS-polyacrylamide gel electrophoresis (SDS-PAGE) image of NT11-PrnC with and without BS(PEG)5 crosslinker. Proteins were separated using a 4-20% Mini-Protean TGX Stain-Free Precast gel (Bio-rad), and the bands obtained were referenced to the Novex Sharp Unstained Protein Standard (Invitrogen). Crosslinking reaction was incubated at room temperature for 30 minutes after which samples were desalted with micro Bio-spin P6 (Bio-Rad) and concentrated with 3K MWCO centricon (Sartorius). (C) These clean-up samples were then used for halogenation reactions with native substrate **1a**, where both samples yield similar conversion (~10%) of **1**.

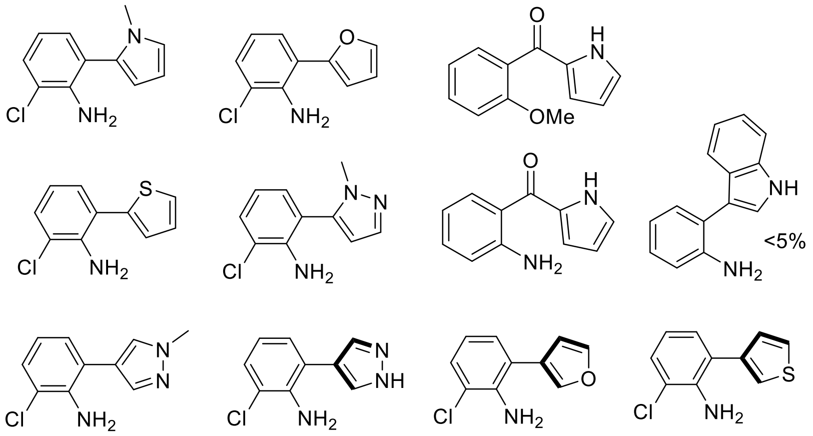

Figure S9. List of substrates not accepted by PrnC.

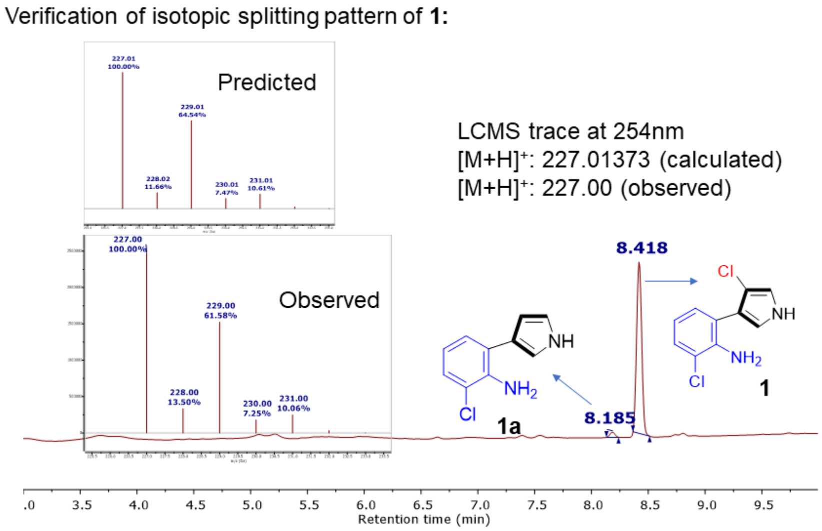

Figure S10. The identity of product **1** is confirmed by LCMS where the appearance of the product UV peak corroborates with the mass to charge ratio (m/z) of the desired chlorinated product. The relative conversion (%) was determined by the area under curve (AUC) at 254 nm where: *rel. conversion* = [AUC(product) / AUC(control)] where AUC(control) = [AUC(product) +AUC(substrate) +AUC(unaccounted mass)]. The degree of chlorination is also verified by the multimode MM-ESI-APCI where **1** yielded three $[M – H]^+$ ions at *m/z* = 227, 229 and 231 in a ~10:6:1 ratio. Other products are also similarly characterized.

1.8.3 LC-MS analytical data for substrates panel using purified NT11-PrnC

1

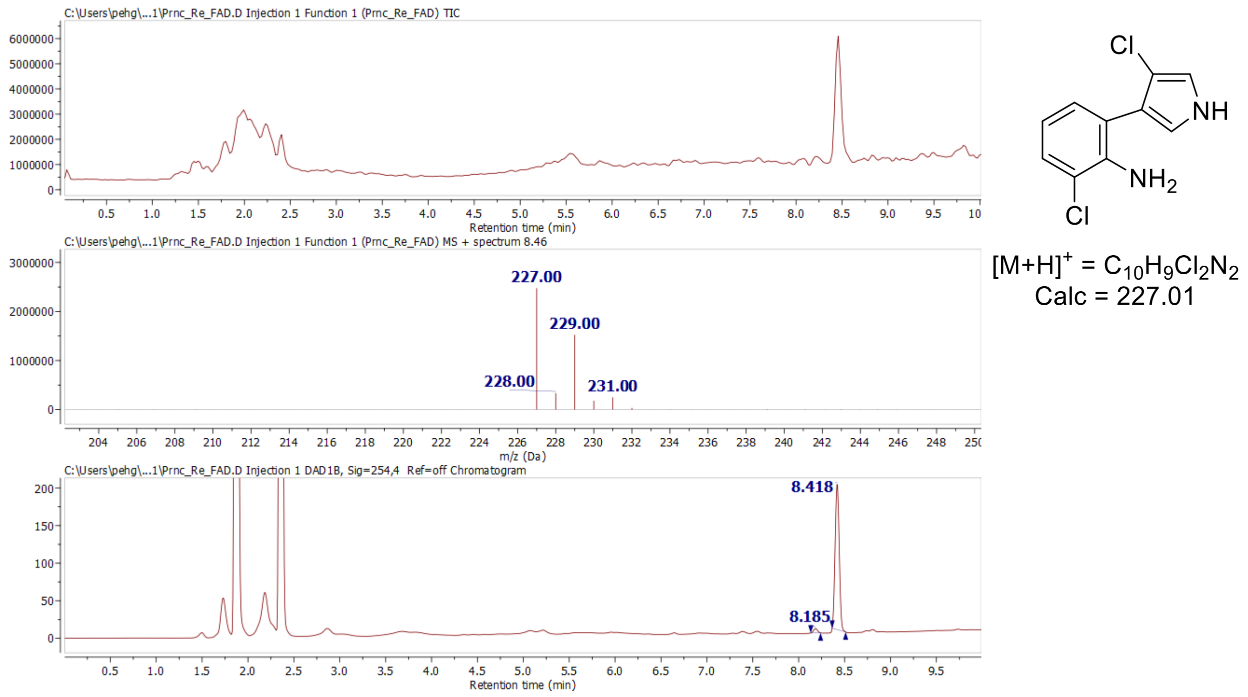

Control (no enzyme)

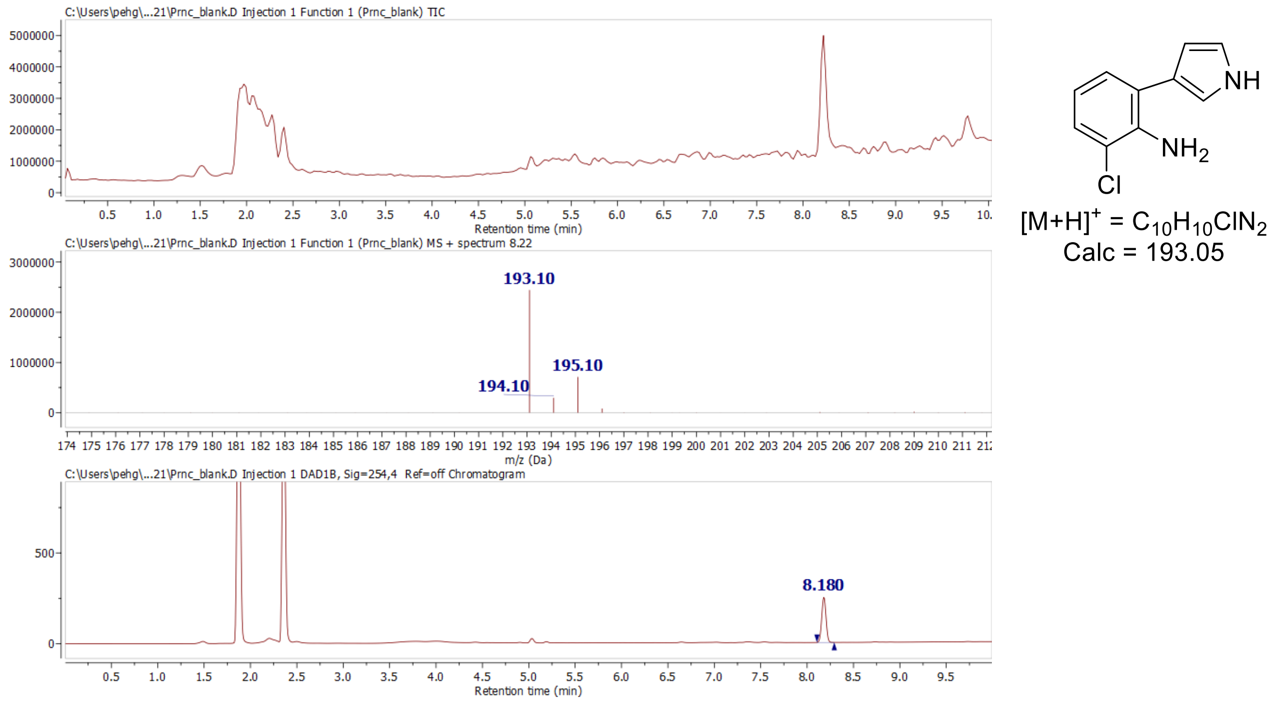

Figure S11. LCMS chromatogram for enzymatic reaction and its control for product **1**.

2

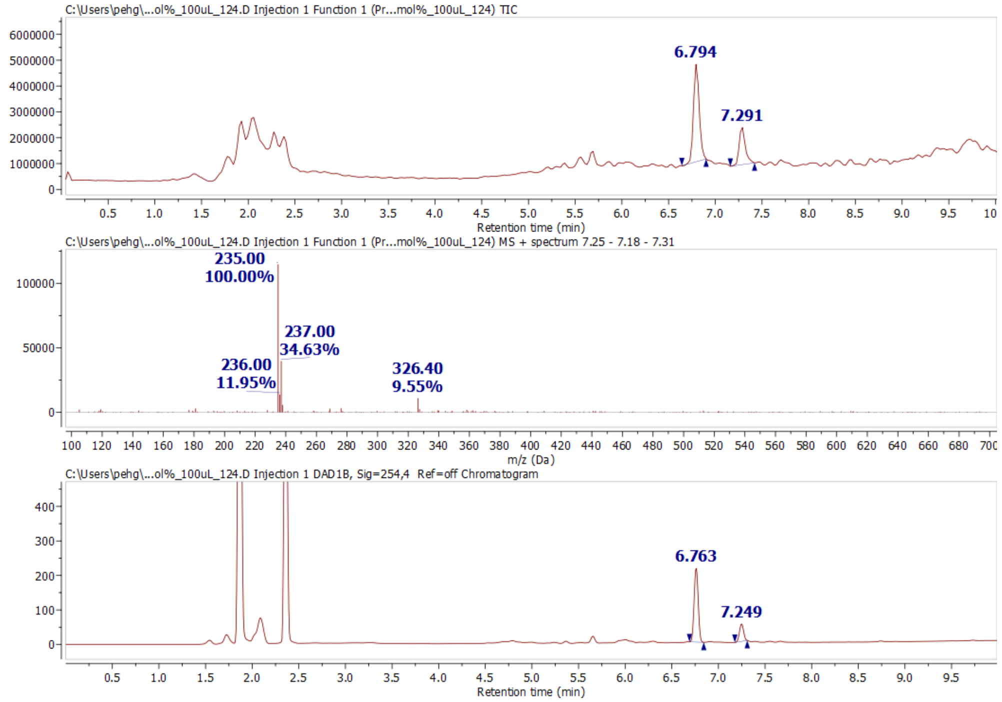

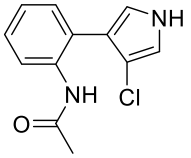

$[M+H]^+ = C_{12}H_{12}ClN_2O$
Calc = 235.06

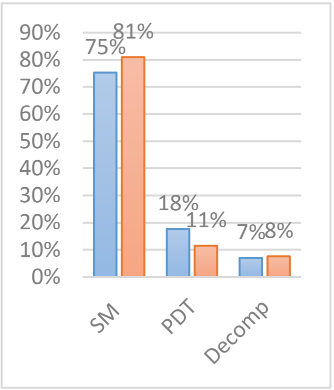

Control

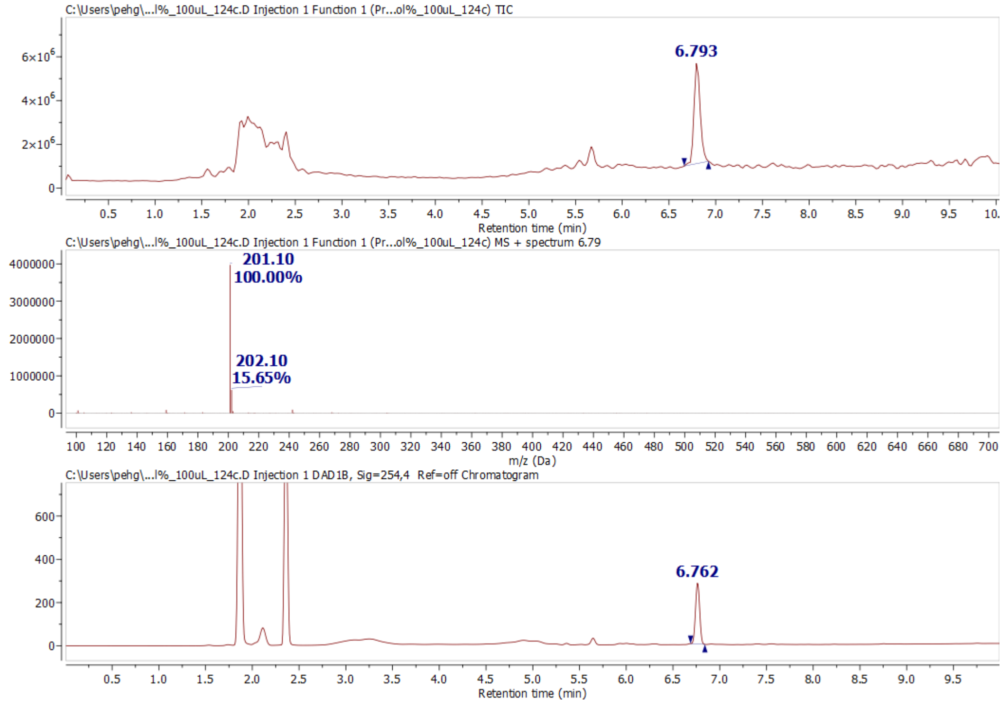

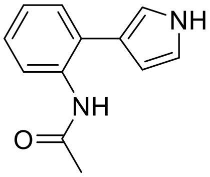

$[M+H]^+ = C_{12}H_{13}N_2O$
Calc = 201.10

Figure S12. LCMS chromatogram for enzymatic reaction and its control for product **2**.

3

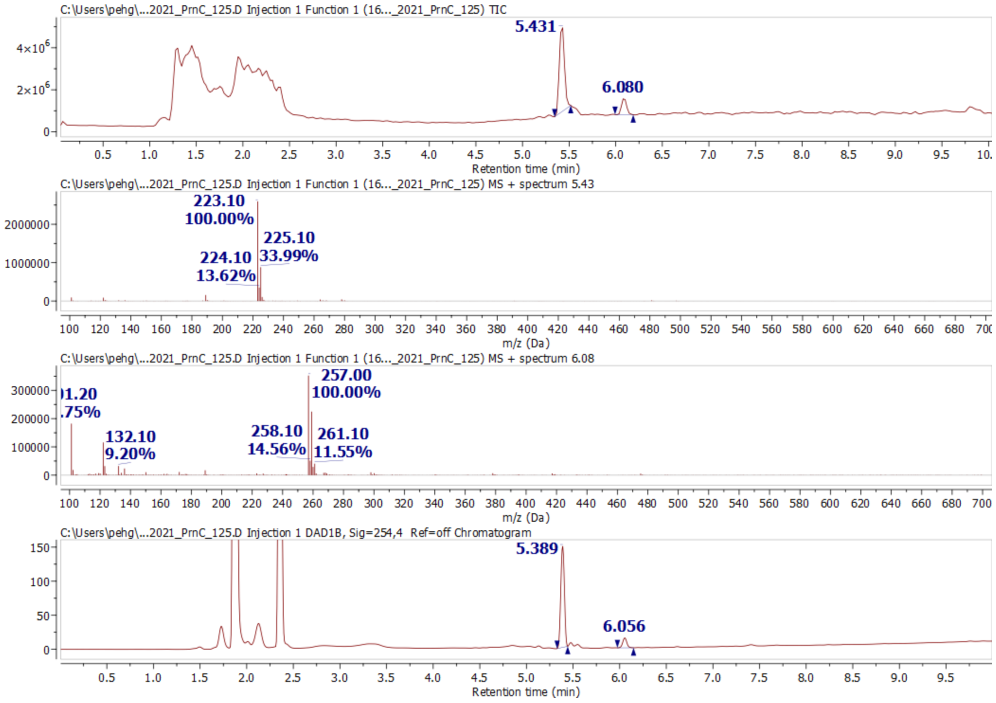

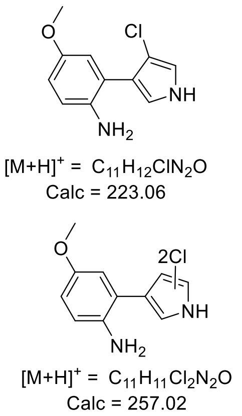

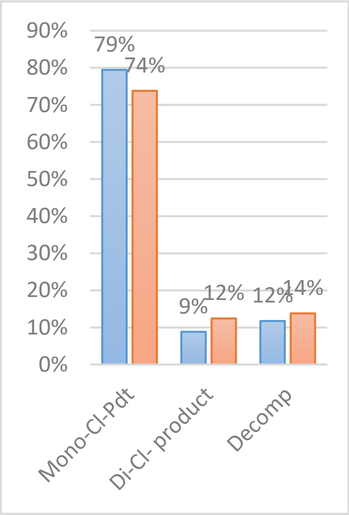

Control

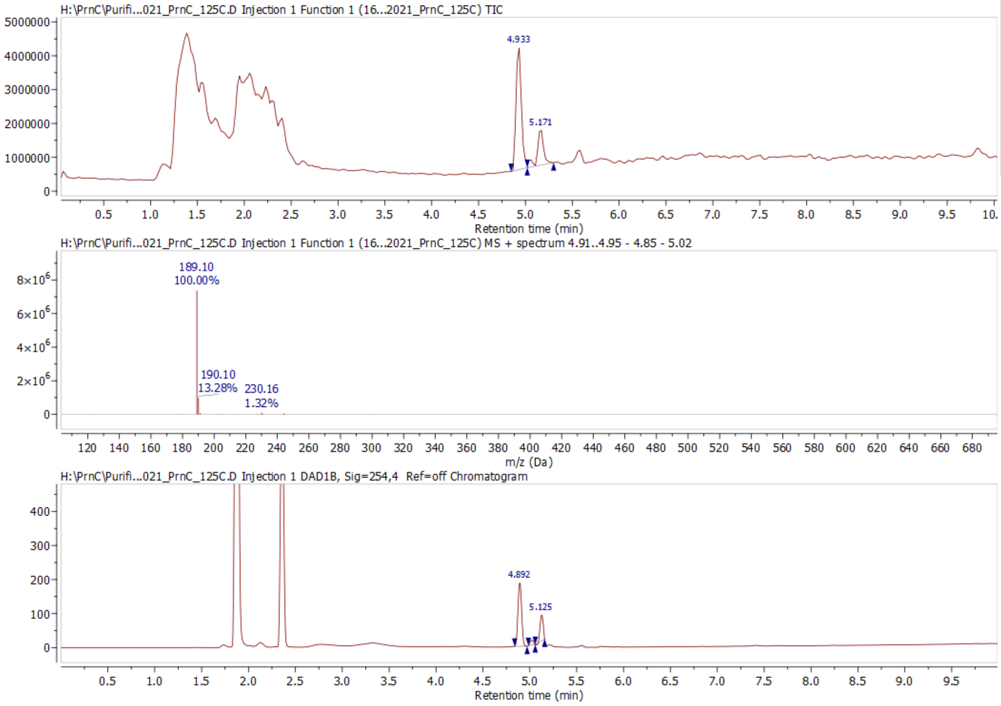

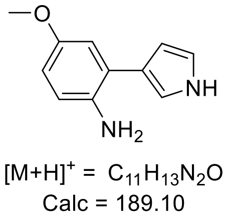

Figure S13. LCMS chromatogram for enzymatic reaction and its control for product **3**.

4

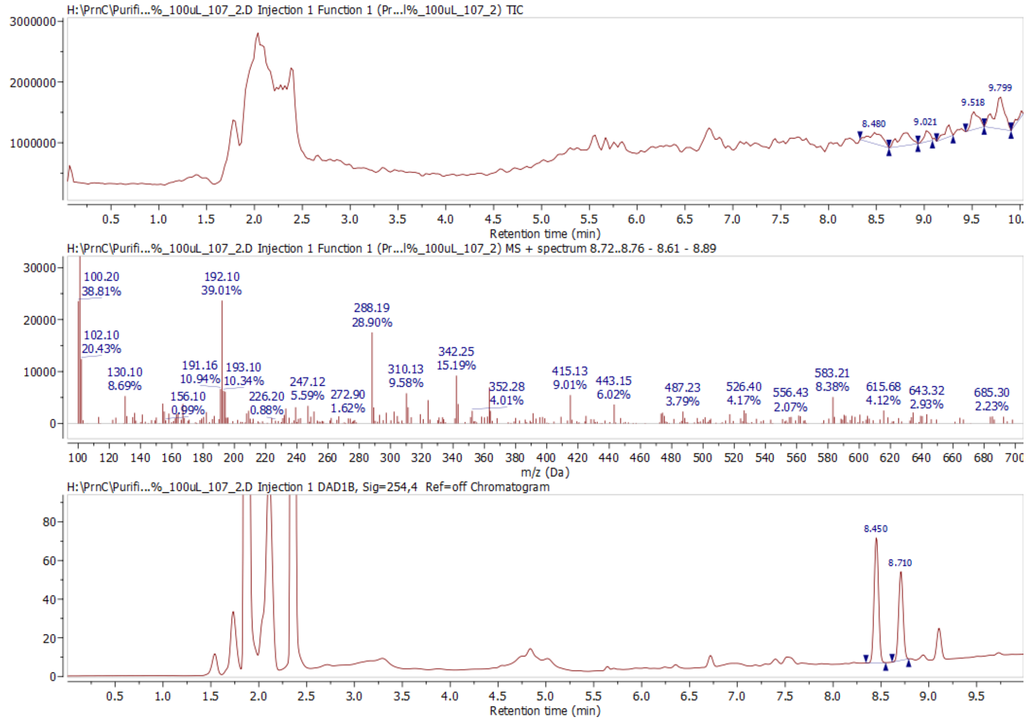

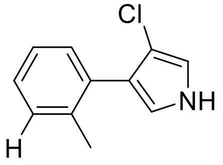

$[M+H]^+$ = $C_{11}H_{11}ClN$
Calc = 192.06

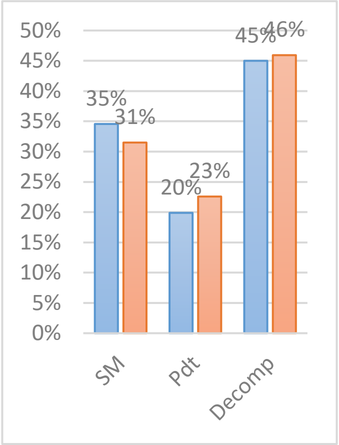

Control

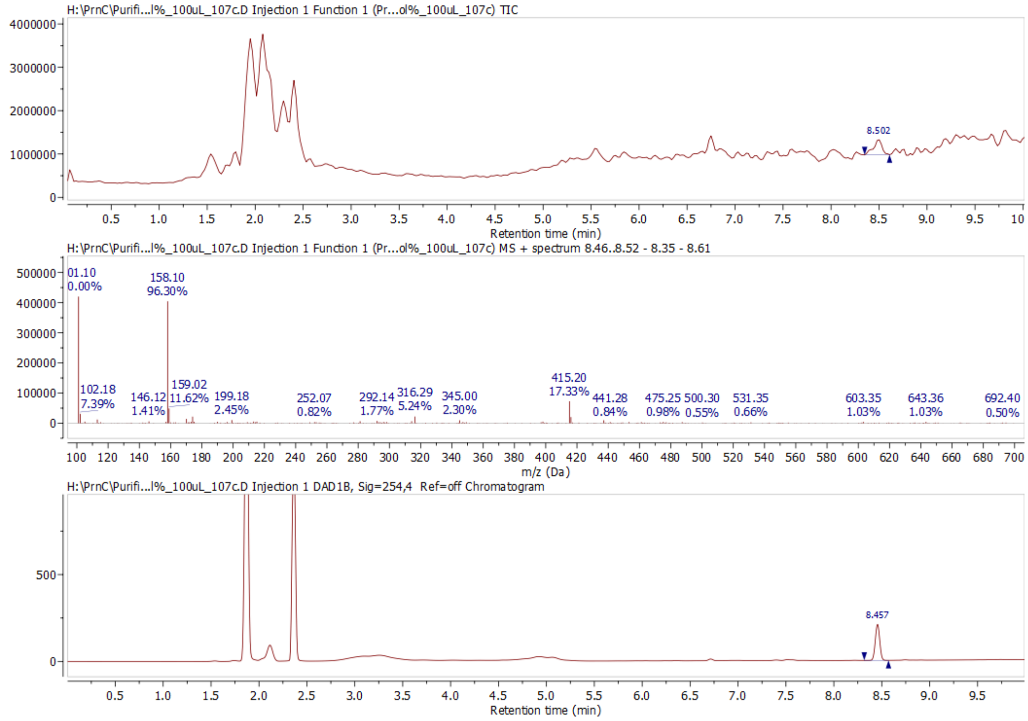

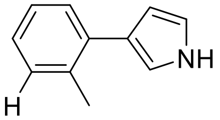

$[M+H]^+$ = $C_{11}H_{12}N$
Calc = 158.10

Figure S14. LCMS chromatogram for enzymatic reaction and its control for product **4**.

5

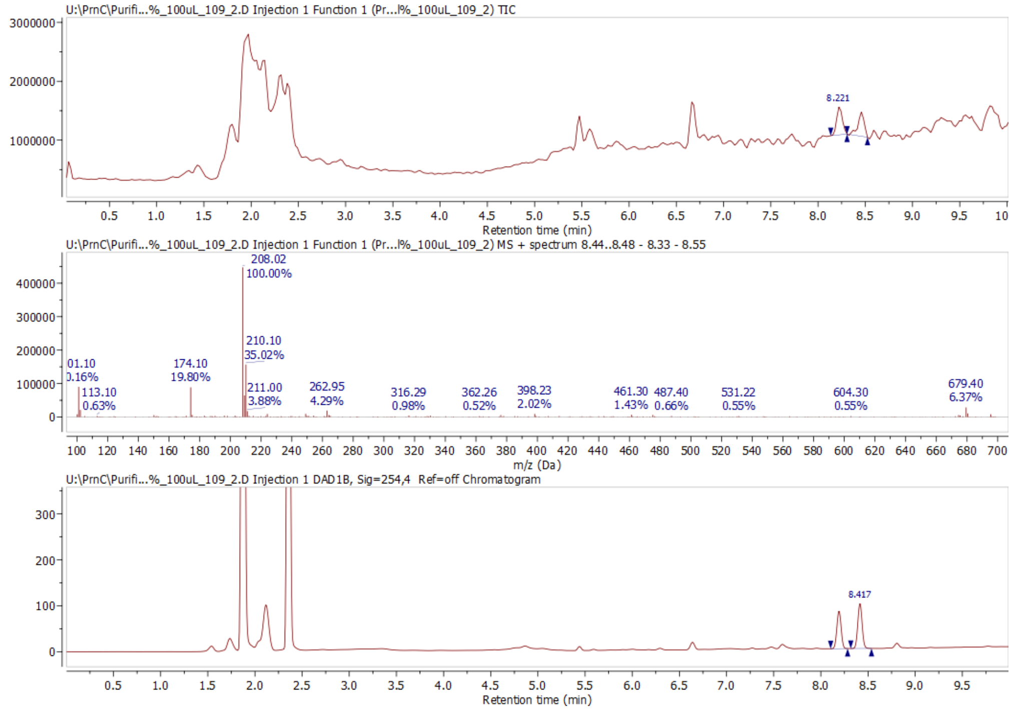

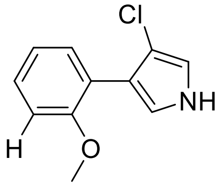

$[M+H]^+ = C_{11}H_{11}ClNO$
Calc = 208.05

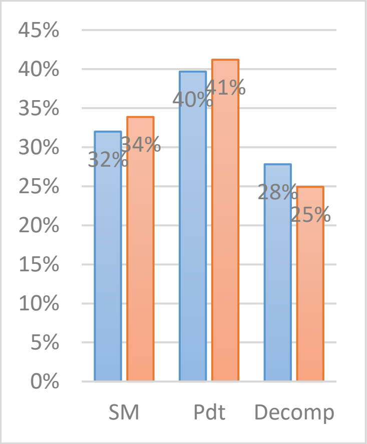

Control

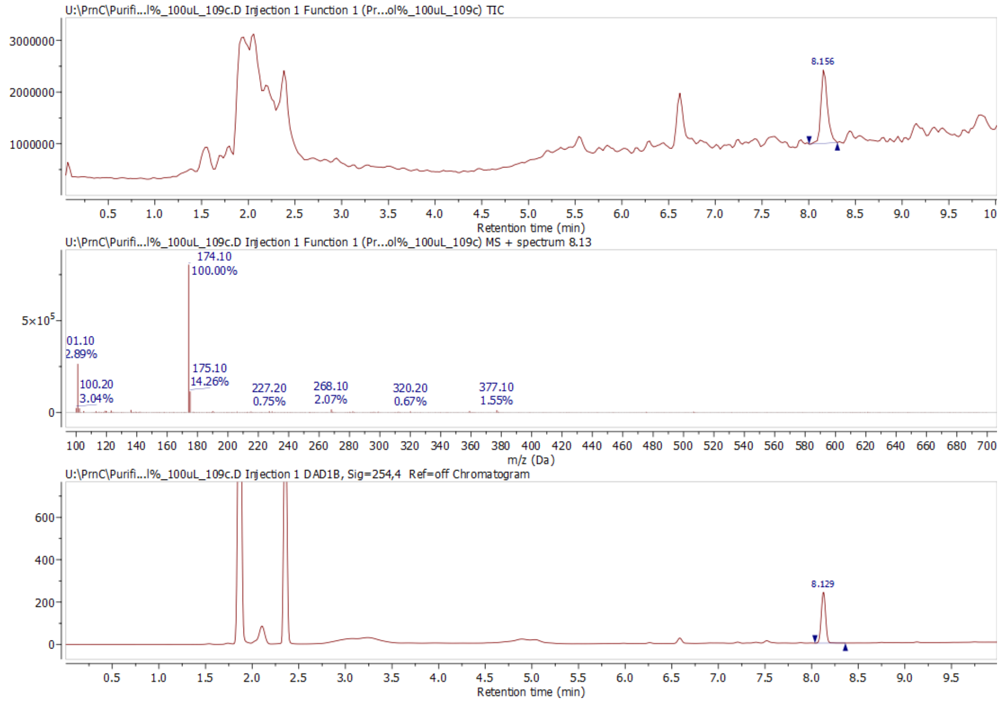

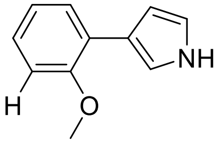

$[M+H]^+ = C_{11}H_{12}NO$
Calc = 174.09

Figure S15. LCMS chromatogram for enzymatic reaction and its control for product **5**.

6

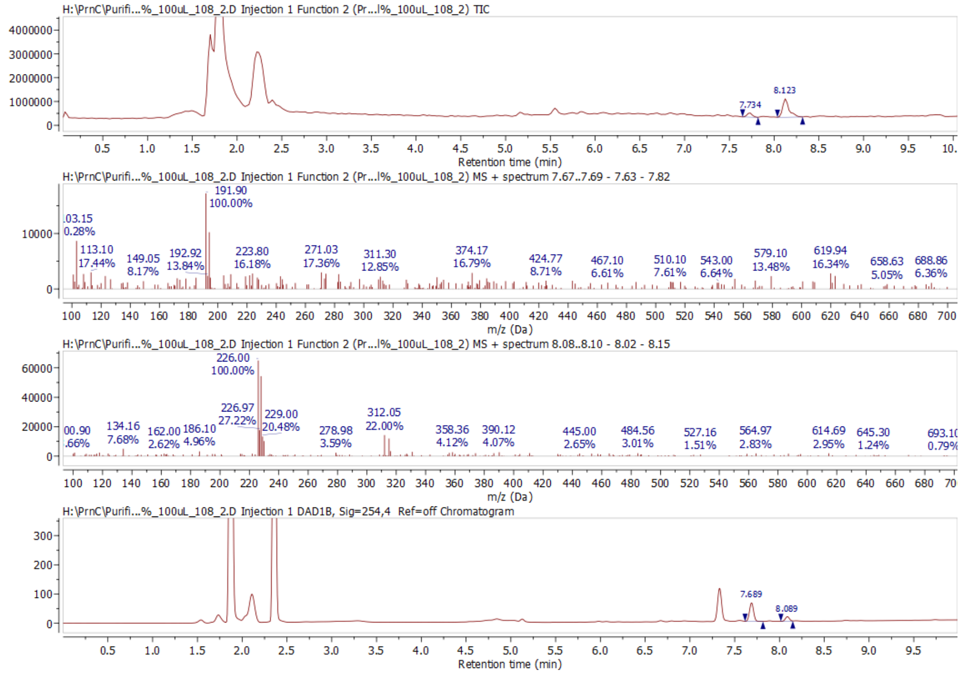

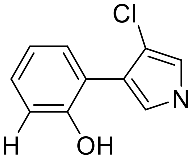

$[M\text{-}H]^- = C_{10}H_7ClNO$

Calc = 192.02

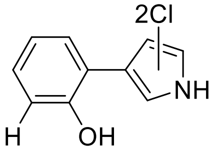

$[M\text{-}H]^- = C_{10}H_6Cl_2NO$

Calc = 225.98

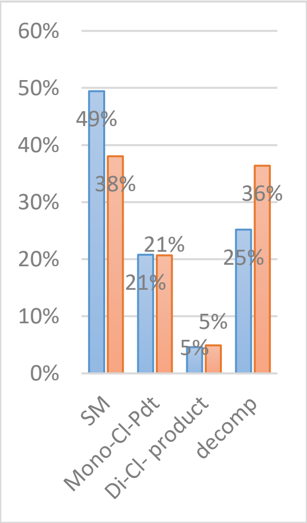

Control

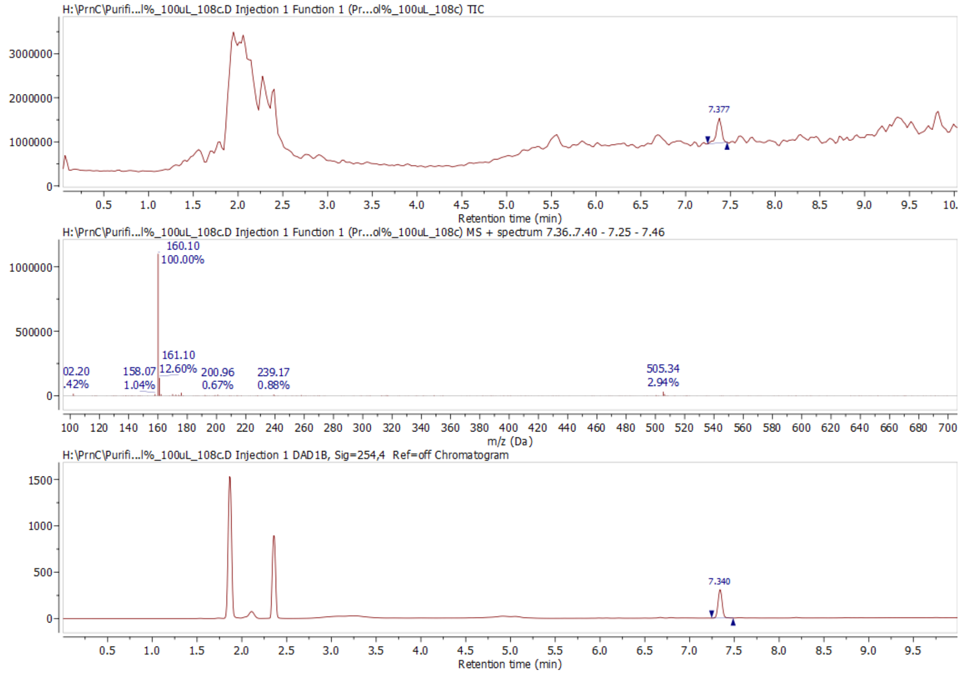

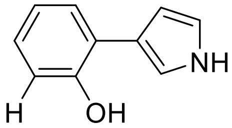

$[M+H]^+ = C_{10}H_{10}NO$

Calc = 160.08

Figure S16. LCMS chromatogram for enzymatic reaction and its control for product **6**.

7

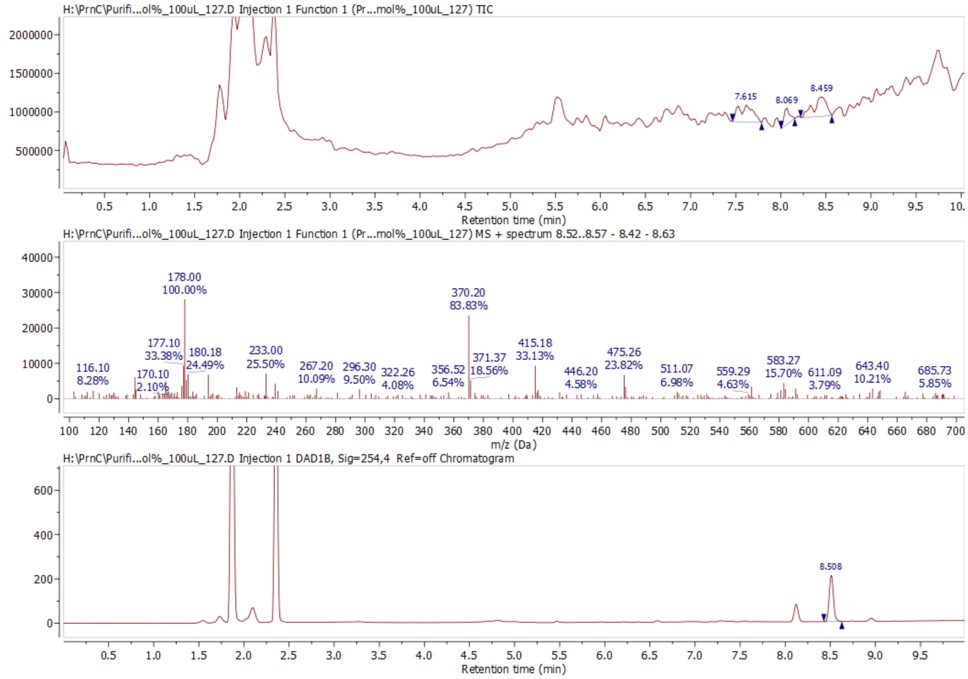

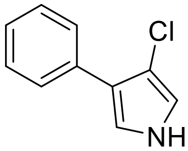

$[M+H]^+ = C_{10}H_9ClN$
Calc = 178.04

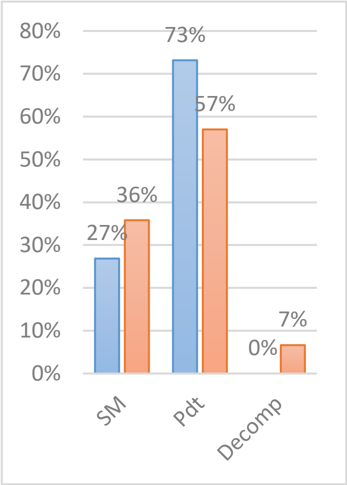

Control

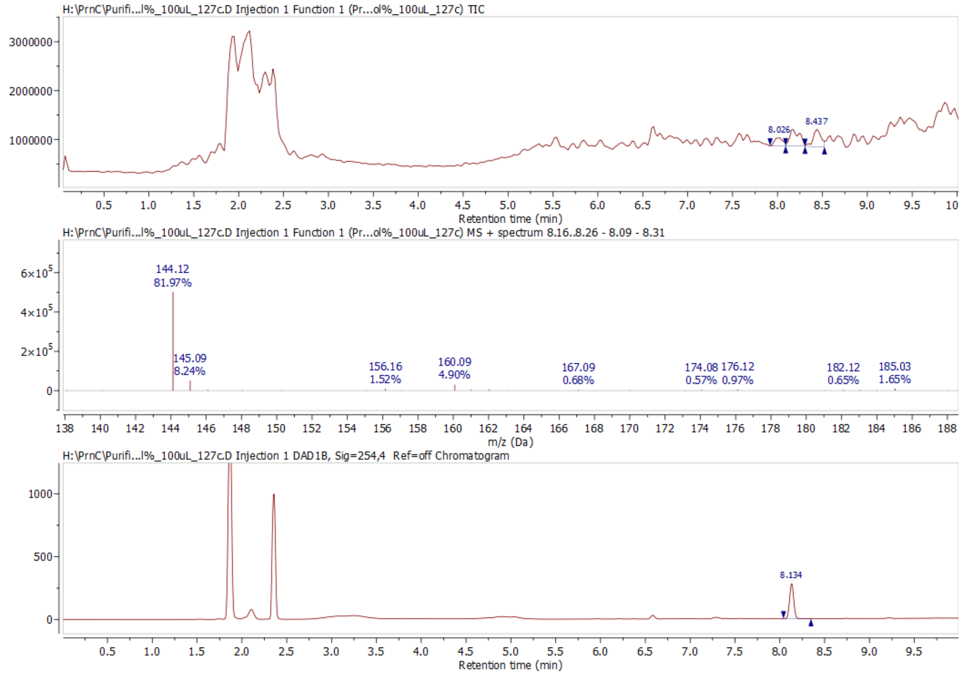

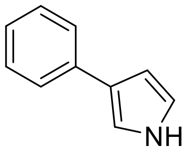

$[M+H]^+ = C_{10}H_{10}N$
Calc = 144.08

Figure S17. LCMS chromatogram for enzymatic reaction and its control for product **7**.

8

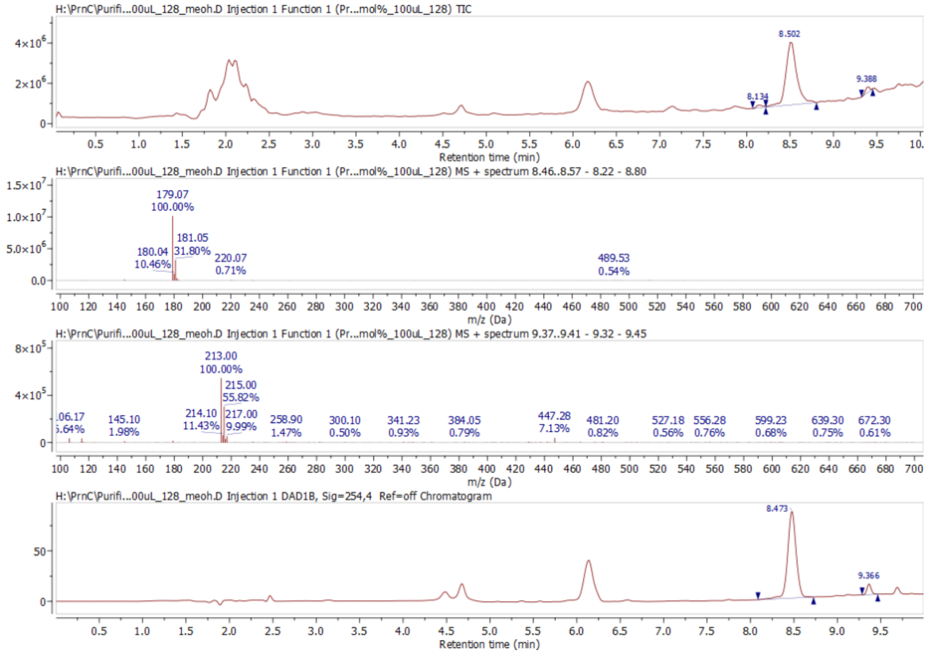

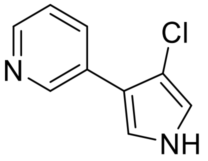

$[M+H]^+ = C_9H_8ClN_2$
Calc = 179.04

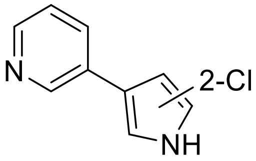

$[M+H]^+ = C_9H_7Cl_2N_2$
Calc = 213.00

Control

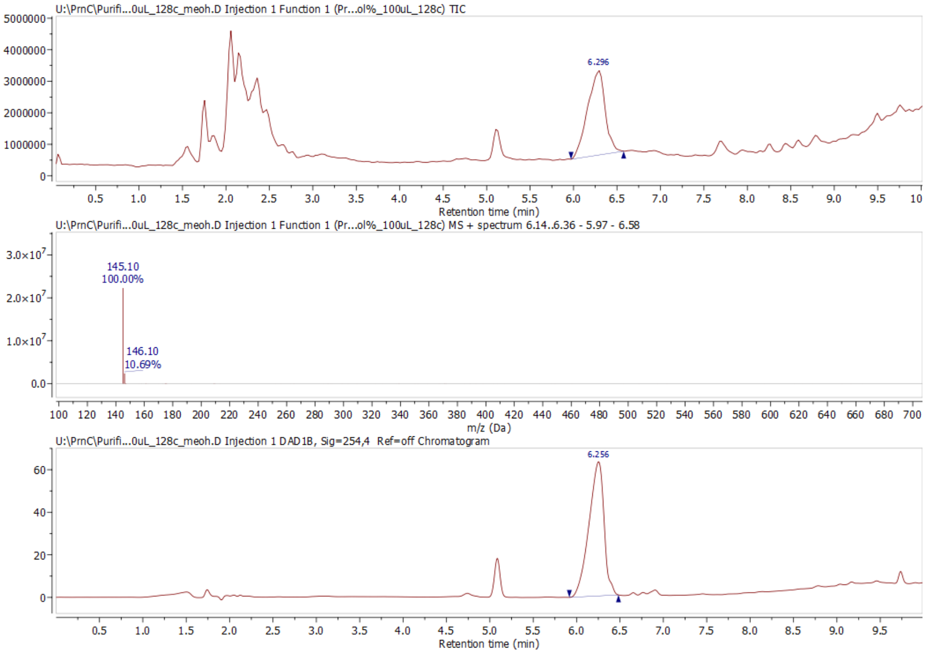

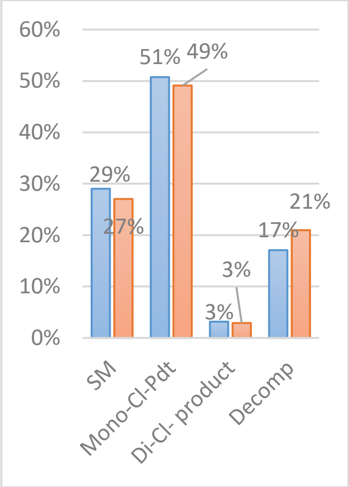

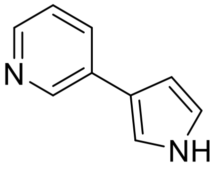

$[M+H]^+ = C_9H_9N_2$
Calc = 145.08

Figure S18. LCMS chromatogram for enzymatic reaction and its control for product **8**.

9

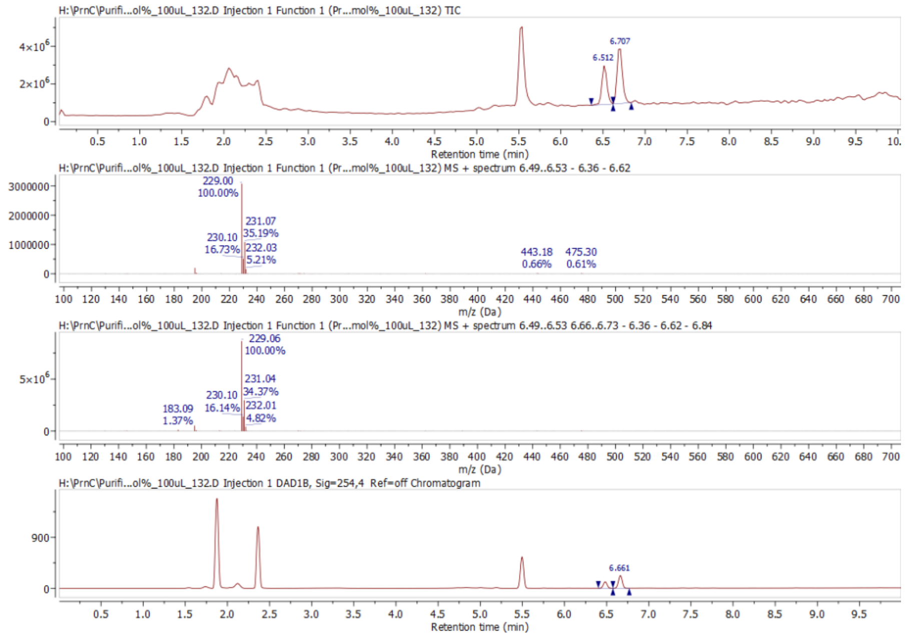

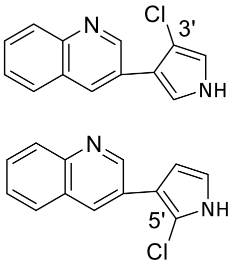

[M+H]$^+$ = $C_{13}H_{10}ClN_2$
Calc = 229.05

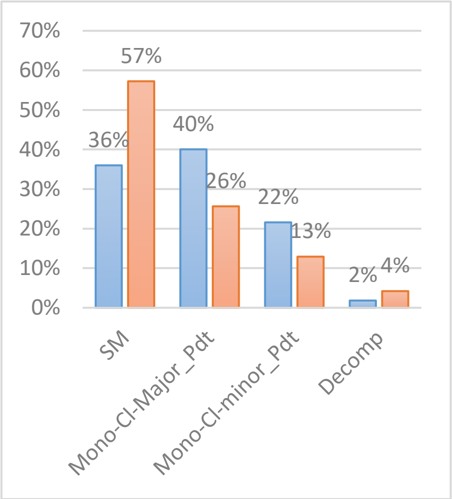

Control

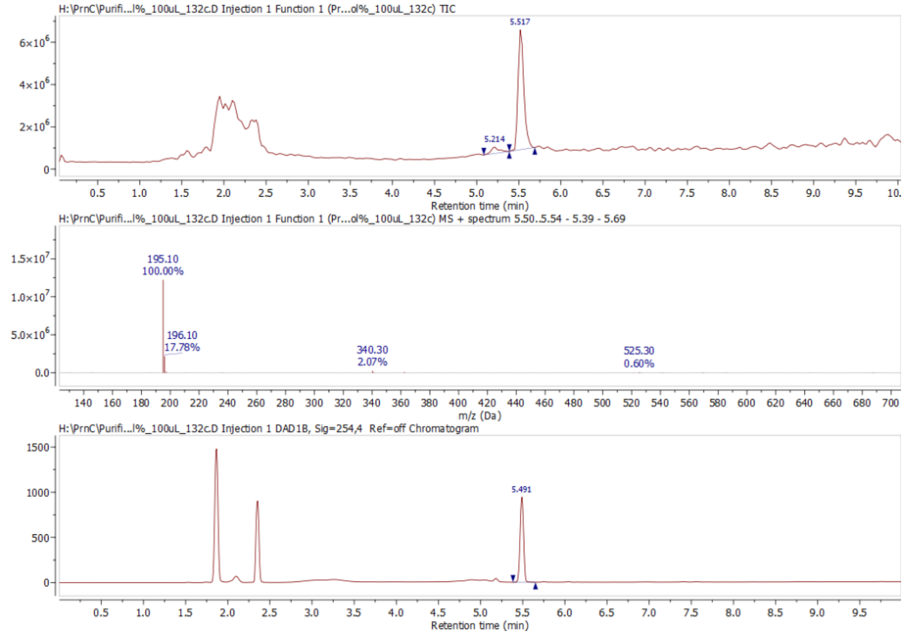

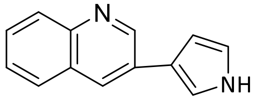

[M+H]$^+$ = $C_{13}H_{11}N_2$
Calc = 195.09

Figure S19. LCMS chromatogram for enzymatic reaction and its control for product **9**.

10

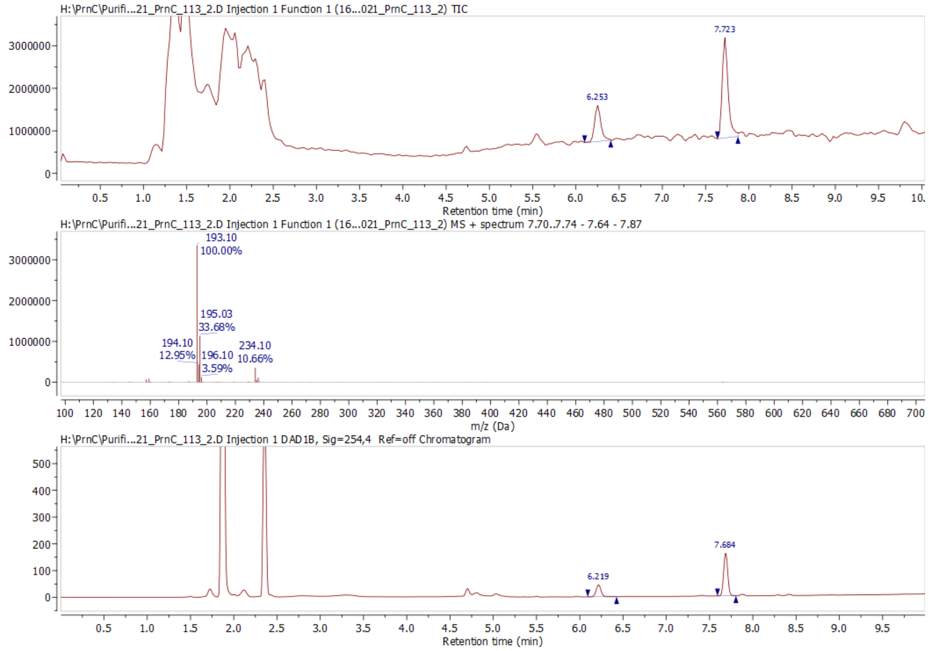

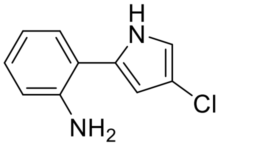

$[M+H]^+ = C_{10}H_{10}ClN_2$

Calc = 193.05

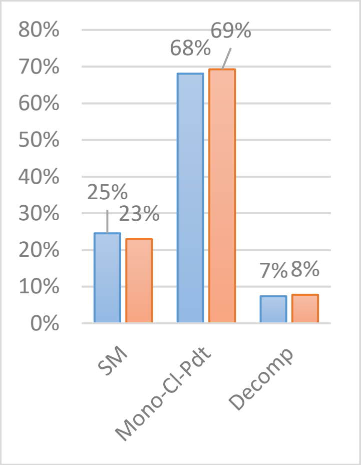

Control

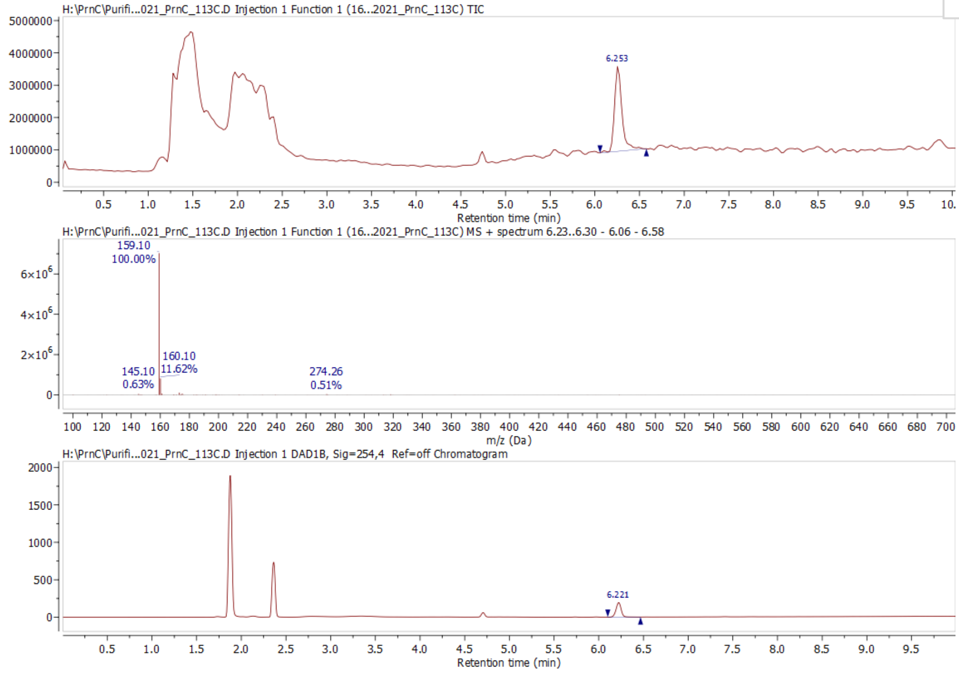

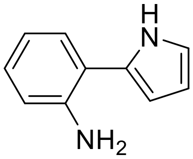

$[M+H]^+ = C_{10}H_{10}ClN_2$

Calc = 159.09

Figure S20. LCMS chromatogram for enzymatic reaction and its control for product **10**.

11

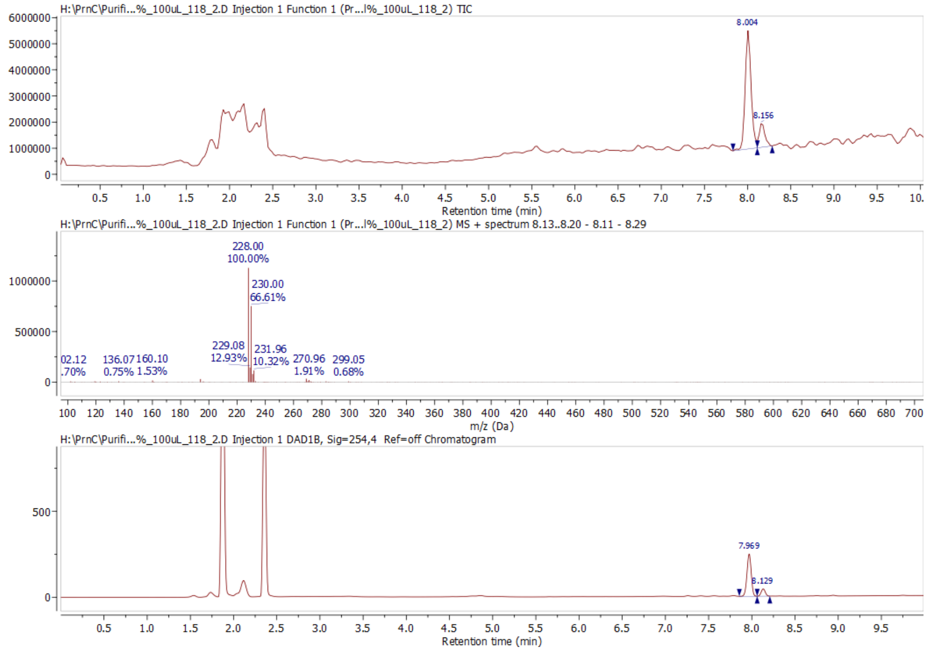

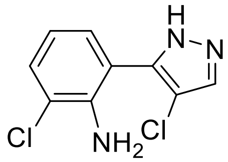

$[M+H]^+ = C_9H_8Cl_2N_3$

Calc = 228.01

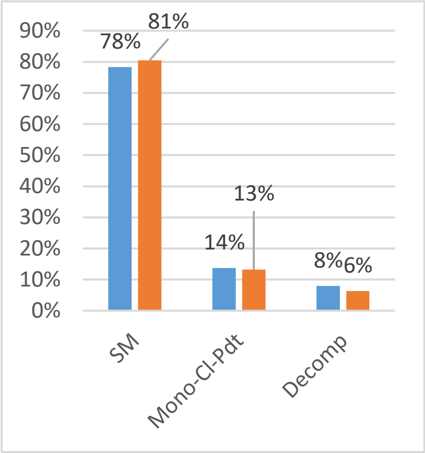

Control

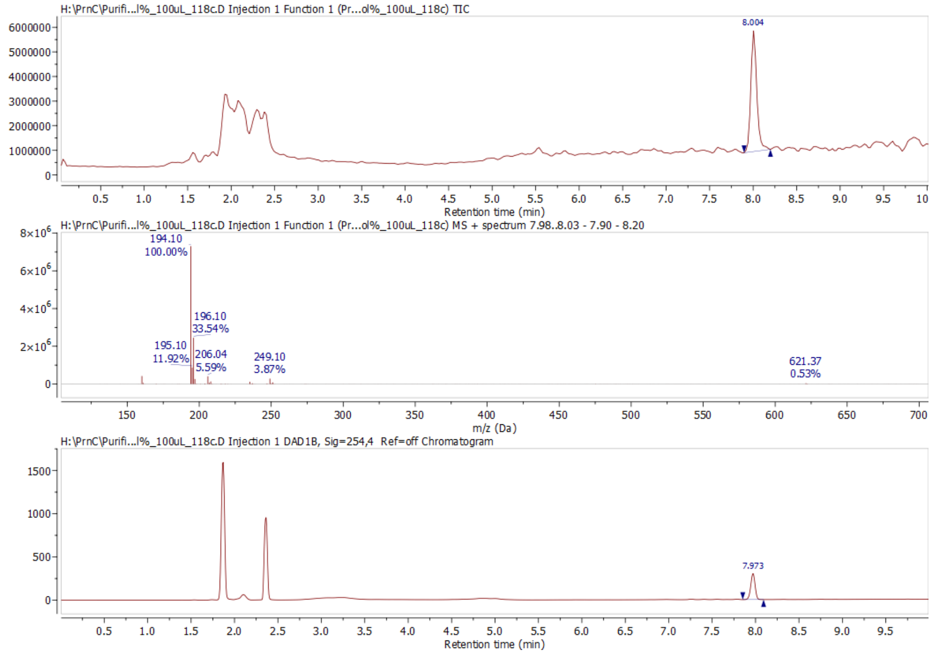

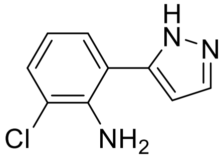

$[M+H]^+ = C_9H_9ClN_3$

Calc = 194.05

Figure S21. LCMS chromatogram for enzymatic reaction and its control for product **11**.

13

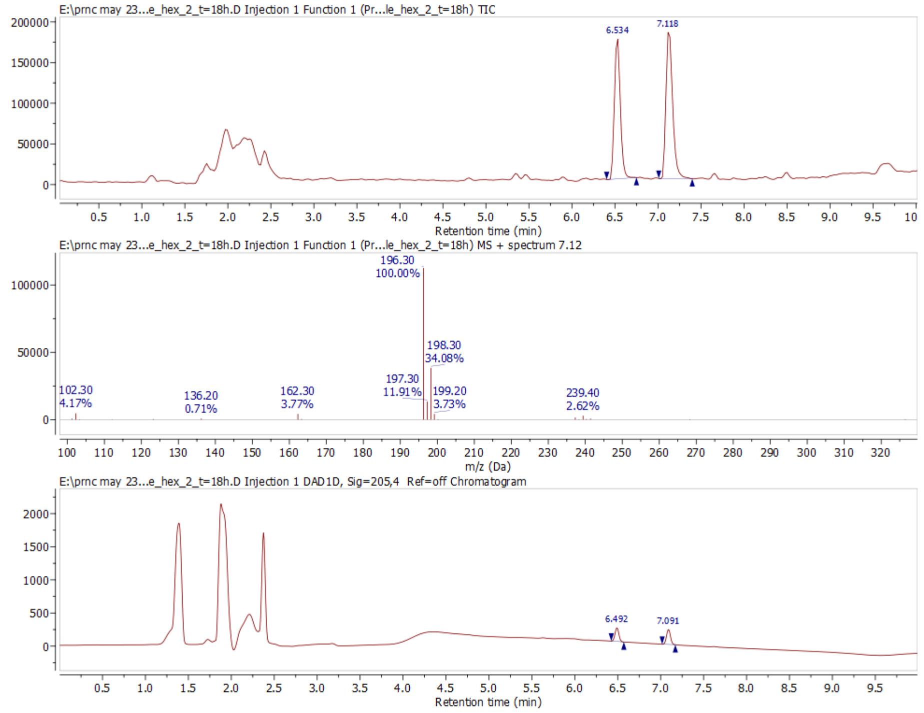

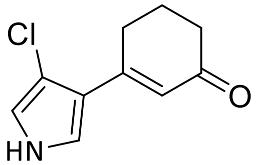

$[M+H]^{+} = C_{10}H_{11}ClNO$

Calc = 196.05

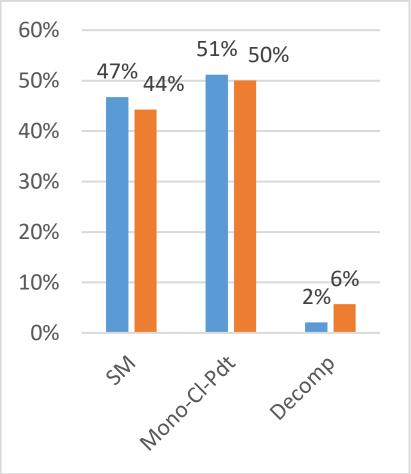

Control

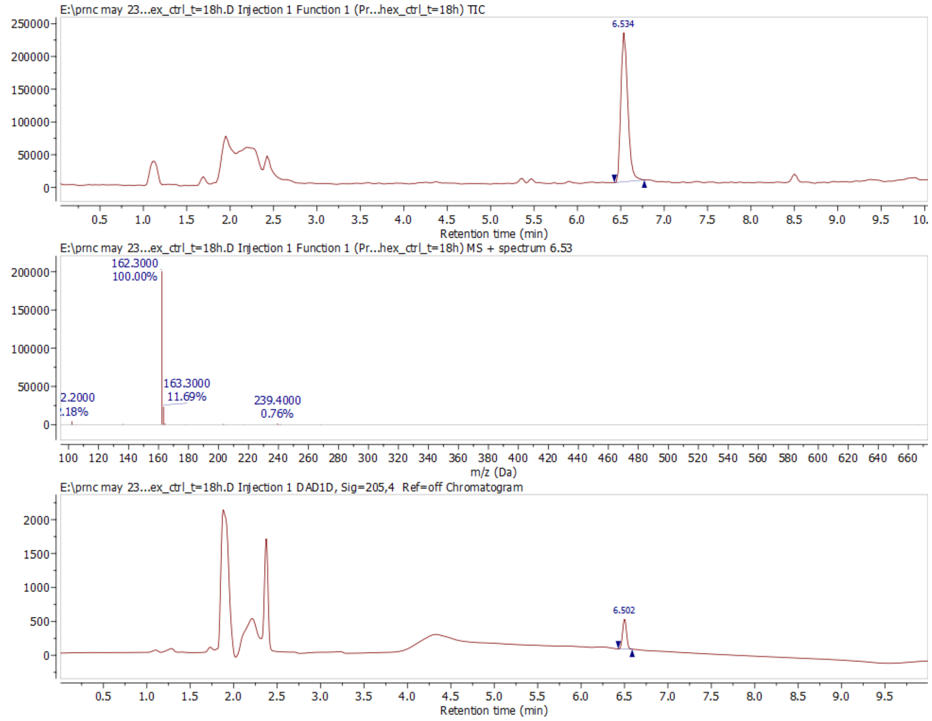

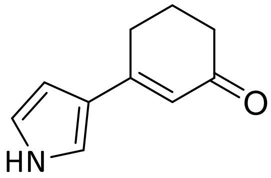

$[M+H]^{+} = C_{10}H_{12}NO$

Calc = 162.09

Figure S22. LCMS chromatogram for enzymatic reaction and its control for product **13**.

14

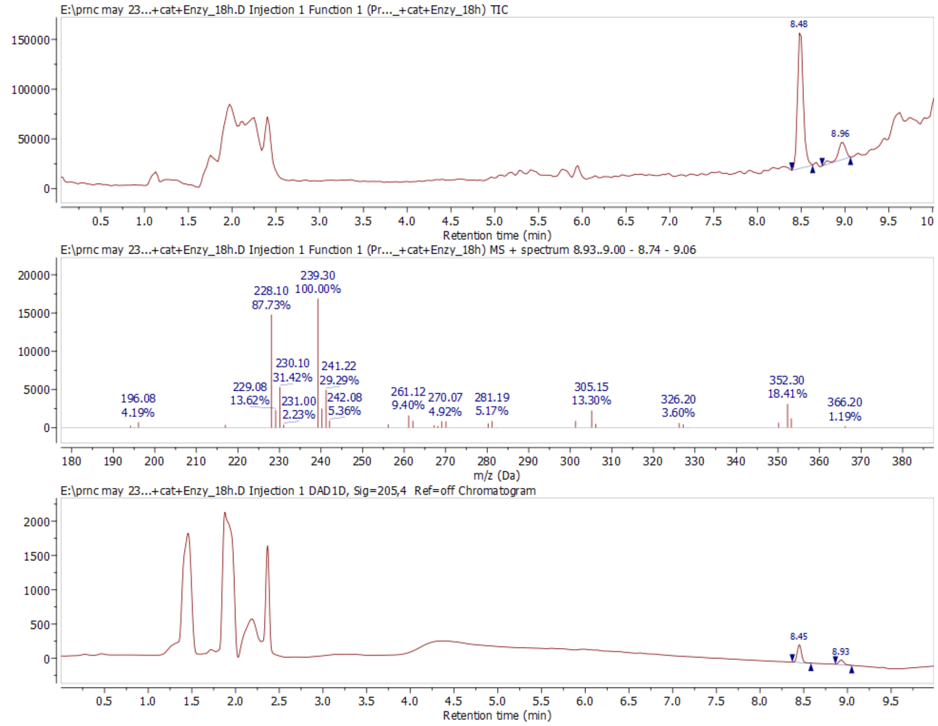

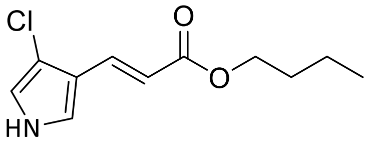

$[M+H]^+ = C_{11}H_{15}ClNO_2$

Calc = 228.08

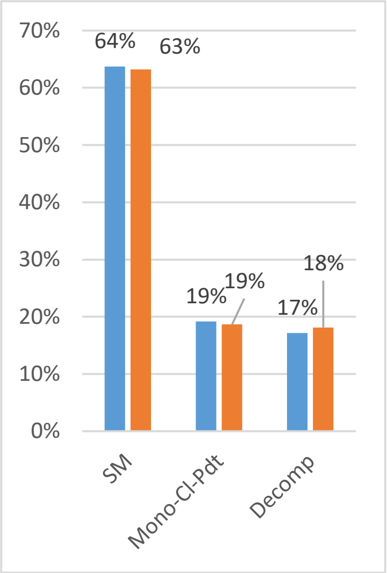

Control

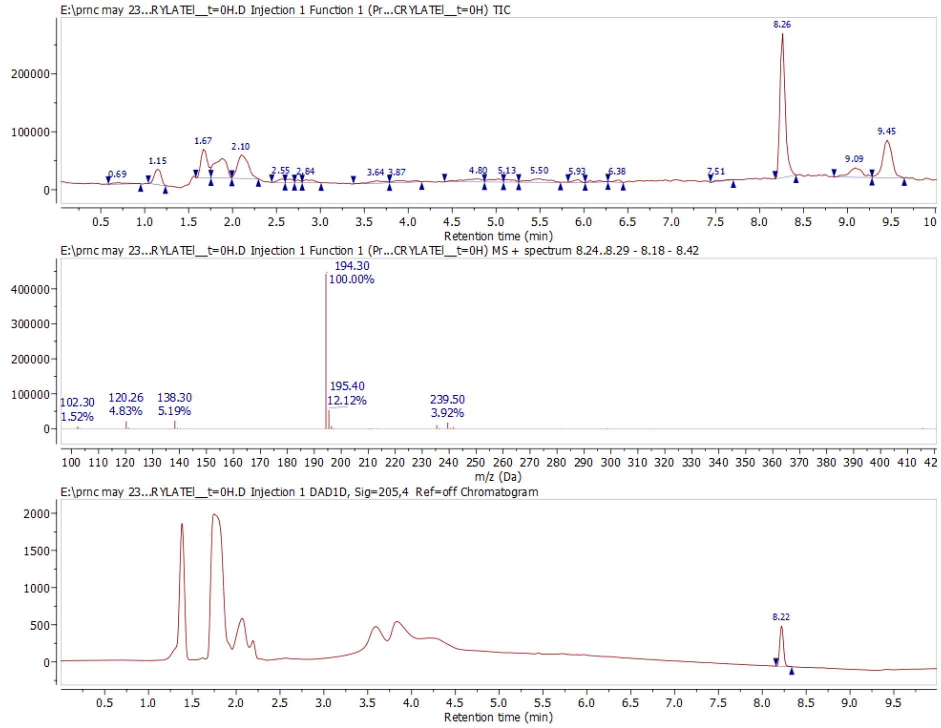

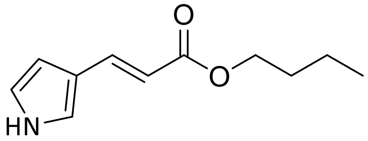

$[M+H]^+ = C_{11}H_{16}NO_2$

Calc = 194.12

Figure S23. LCMS chromatogram for enzymatic reaction and its control for product **14**.

17

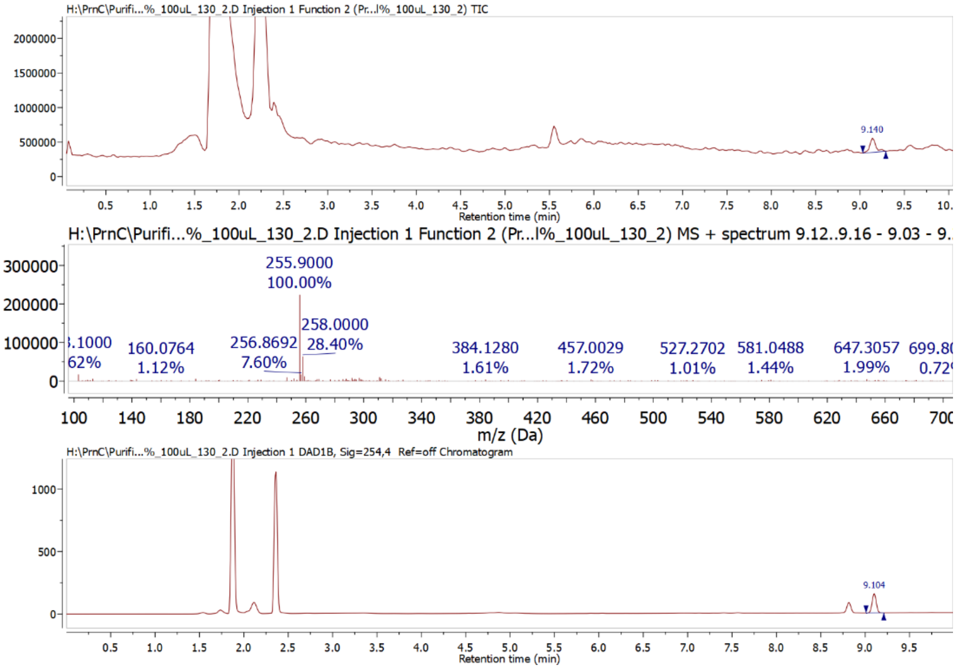

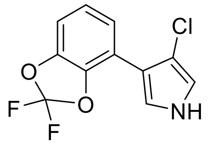

$[M\text{-}H]^- = C_{11}H_5ClF_2NO_2$

Calc = 256.00

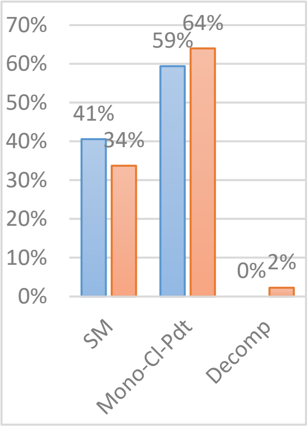

Control

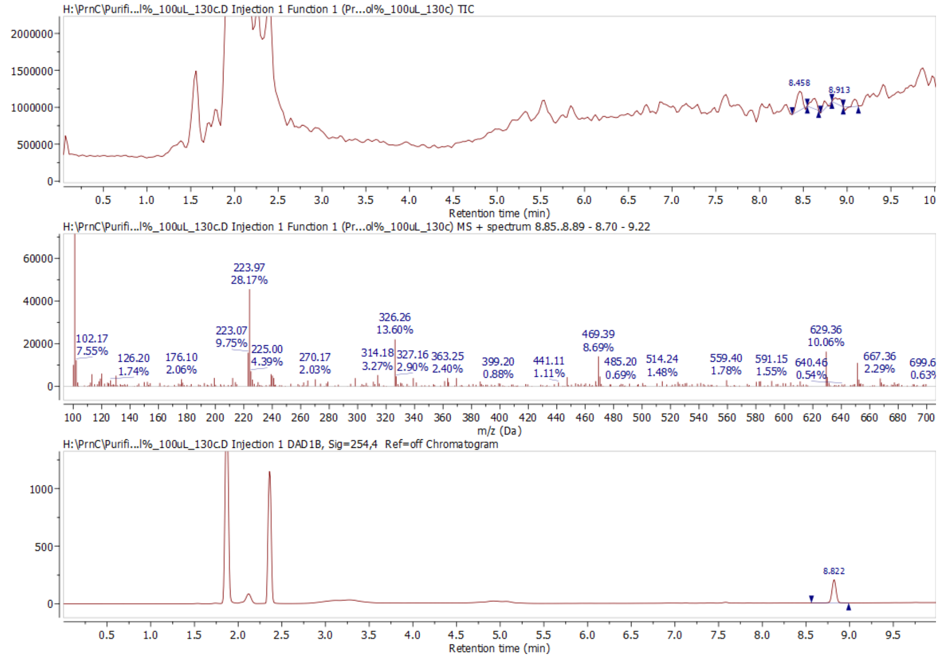

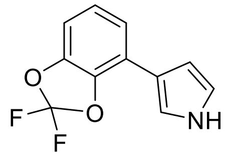

Chemical Formula: $C_{11}H_7F_2NO_2$

Exact Mass: 223.0445

Figure S24. LCMS chromatogram for enzymatic reaction and its control for product **16**.

2. Supplementary References

1. T. K. M. Nguyen, M. R. Ki, R. G. Son and S. P. Pack, *Applied Microbiology and Biotechnology*, 2019, **103**, 2205-2216.
2. R. Zallot, N. Oberg and J. A. Gerlt, *Biochemistry*, 2019, **58**, 4169-4182.
3. C. R. Marr, S. Benlekbir and J. L. Rubinstein, *Journal of Structural Biology*, 2014, **185**, 42-47.
4. D. N. Mastronarde, *Journal of structural biology*, 2005, **152**, 36-51.
5. M. Schorb, I. Haberbosch, W. J. Hagen, Y. Schwab and D. N. Mastronarde, *Nature methods*, 2019, **16**, 471-477.
6. A. Punjani, J. L. Rubinstein, D. J. Fleet and M. A. Brubaker, *Nature methods*, 2017, **14**, 290-296.
7. E. F. Pettersen, T. D. Goddard, C. C. Huang, G. S. Couch, D. M. Greenblatt, E. C. Meng and T. E. Ferrin, *Journal of computational chemistry*, 2004, **25**, 1605-1612.
8. M. D. Morrison, J. J. Hanthorn and D. A. Pratt, *Organic Letters*, 2009, **11**, 1051-1054.
9. K. H. van Pée, O. Salcher, P. Fischer, M. Bokel and F. Lingens, *J Antibiot (Tokyo)*, 1983, **36**, 1735-1742.
10. N. D. Smith, D. Huang and N. D. P. Cosford, *Organic Letters*, 2002, **4**, 3537-3539.
11. R. D. Rieth, N. P. Mankad, E. Calimano and J. P. Sadighi, *Organic Letters*, 2004, **6**, 3981-3983.
12. N. P. Pavri and M. L. Trudell, *The Journal of Organic Chemistry*, 1997, **62**, 2649-2651.
13. A. Alvarez, A. Guzman, A. Ruiz, E. Velarde and J. M. Muchowski, *The Journal of Organic Chemistry*, 1992, **57**, 1653-1656.
14. R. J. Armstrong, M. D'Ascenzio and M. D. Smith, *Synlett*, 2016, **27**, 6-10.
15. E. M. Beck, N. P. Grimster, R. Hatley and M. J. Gaunt, *Journal of the American Chemical Society*, 2006, **128**, 2528-2529.
16. C. Dong, S. Flecks, S. Unversucht, C. Haupt, K.-H. v. Pée and J. H. Naismith, *Science*, 2005, **309**, 2216-2219.
17. E. Yeh, S. Garneau and C. T. Walsh, *Proceedings of the National Academy of Sciences*, 2005, **102**, 3960-3965.
18. S. Mori, A. H. Pang, N. Thamban Chandrika, S. Garneau-Tsodikova and O. V. Tsodikov, *Nature Communications*, 2019, **10**, 1255.
