# Supplementary material for: Site-selective chlorination of pyrrolic heterocycles by flavin dependent enzyme PrnC
Source: Commun Chem. 2024 Jan 5;7:7. doi: 10.1038/s42004-023-01083-1 (PMC10770391; doi:10.1038/s42004-023-01083-1)
Supplement: Supplementary file 2 — Supplementary Data 1 [file 42004_2023_1083_MOESM2_ESM.pdf]

a. Representative examples of halogenation site determination on pyrrolic compounds.

Pyrrole derivatives were chlorinated and worked-up at larger scale using PrnC cell lysate or *in-vivo* protocol. The halogenation site was identified by a combination of ^{1}H NMR, 2D and ^{13}C spectroscopy methods.

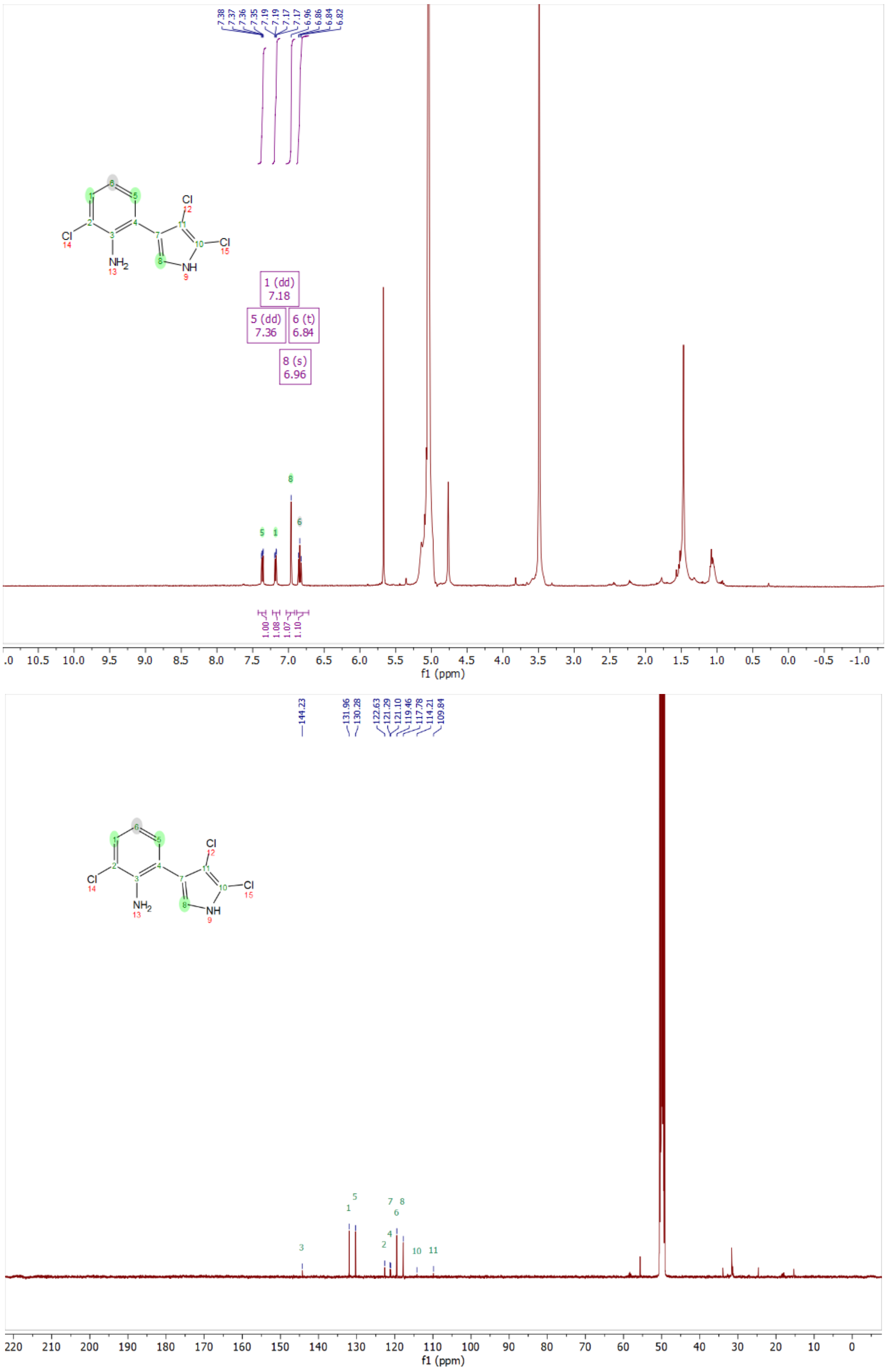

Figure S25. ^{1}H and ^{13}C NMR spectrum for 2Cl-**1**.

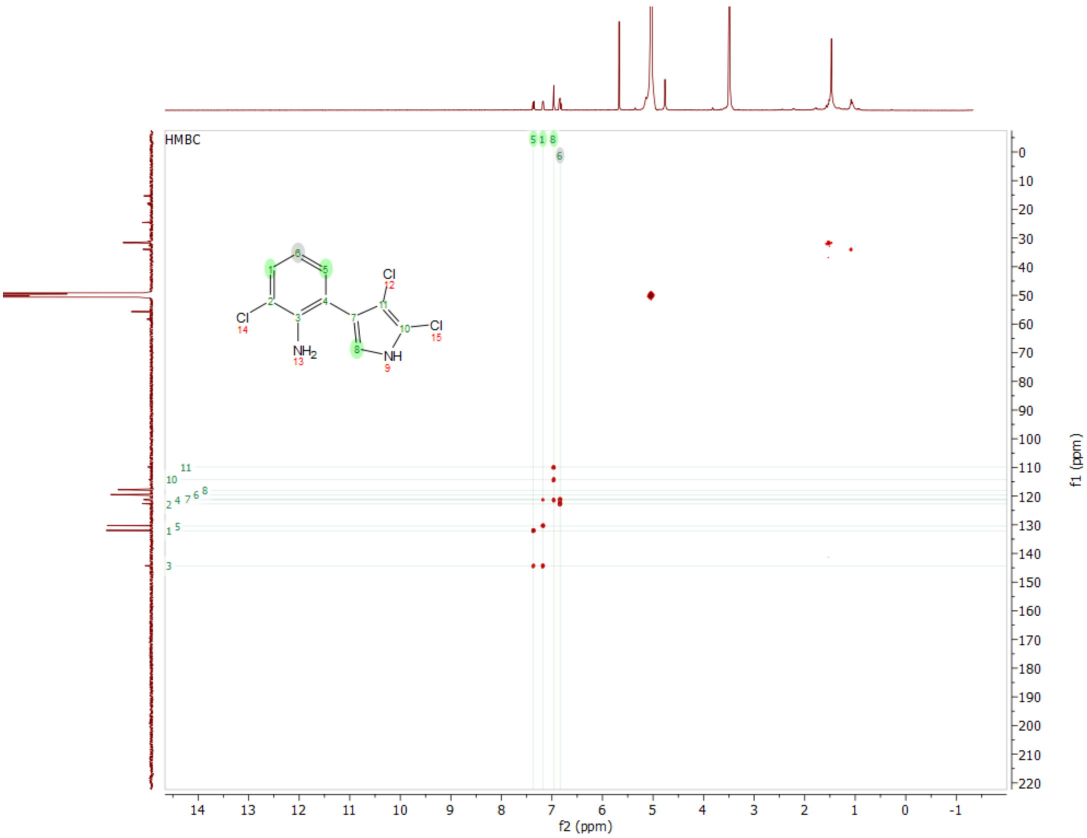

Figure S26. ^{1}H-^{13}C HMBC NMR spectrum for product 2Cl-**1**.

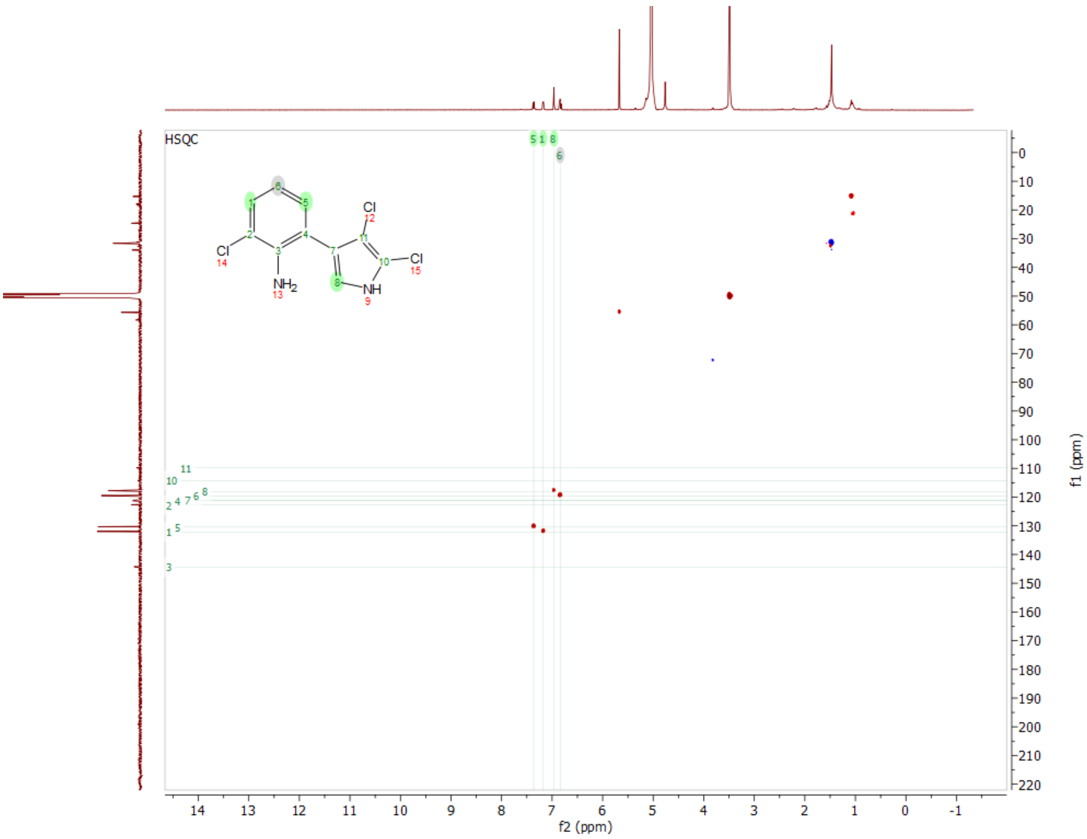

Figure S27. ^{1}H-^{13}C HSQC NMR spectrum for product 2Cl-**1**.

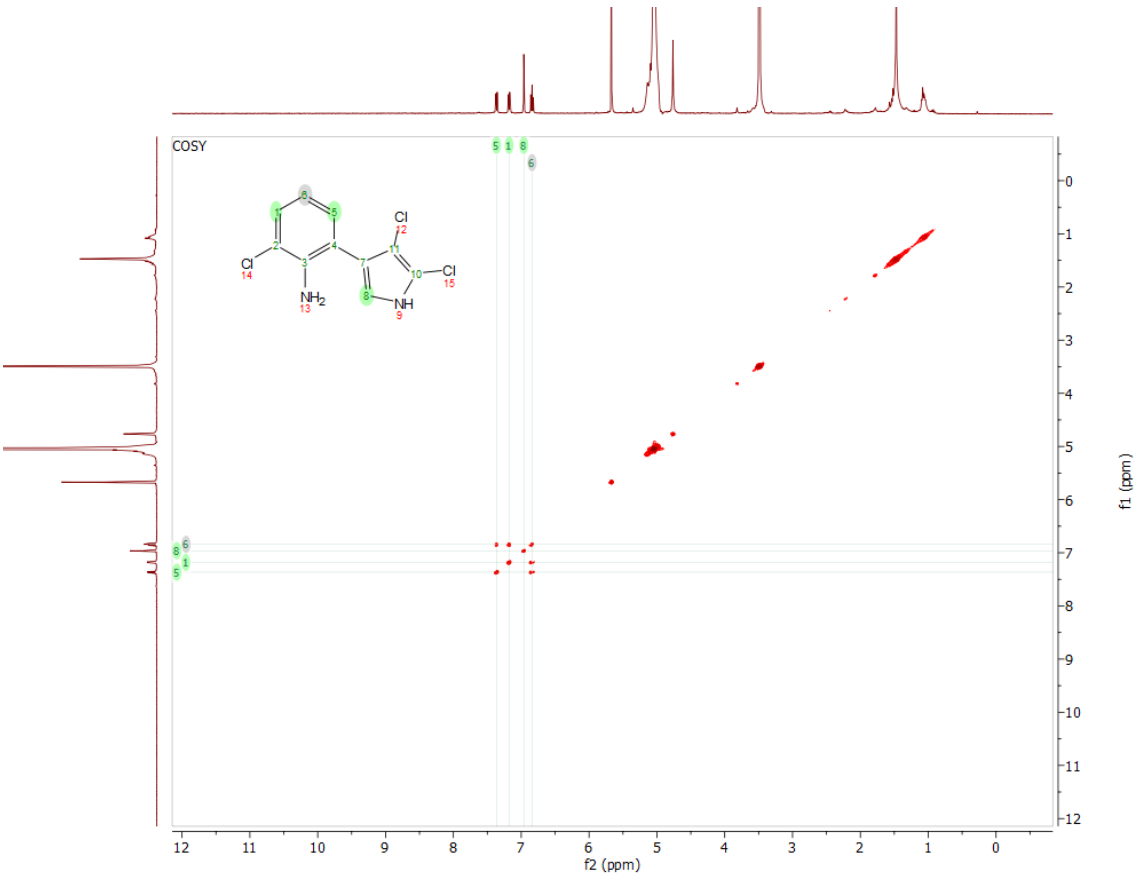

Figure S28. ^{1}H-^{1}H COSY NMR spectrum for product 2Cl-**1**.

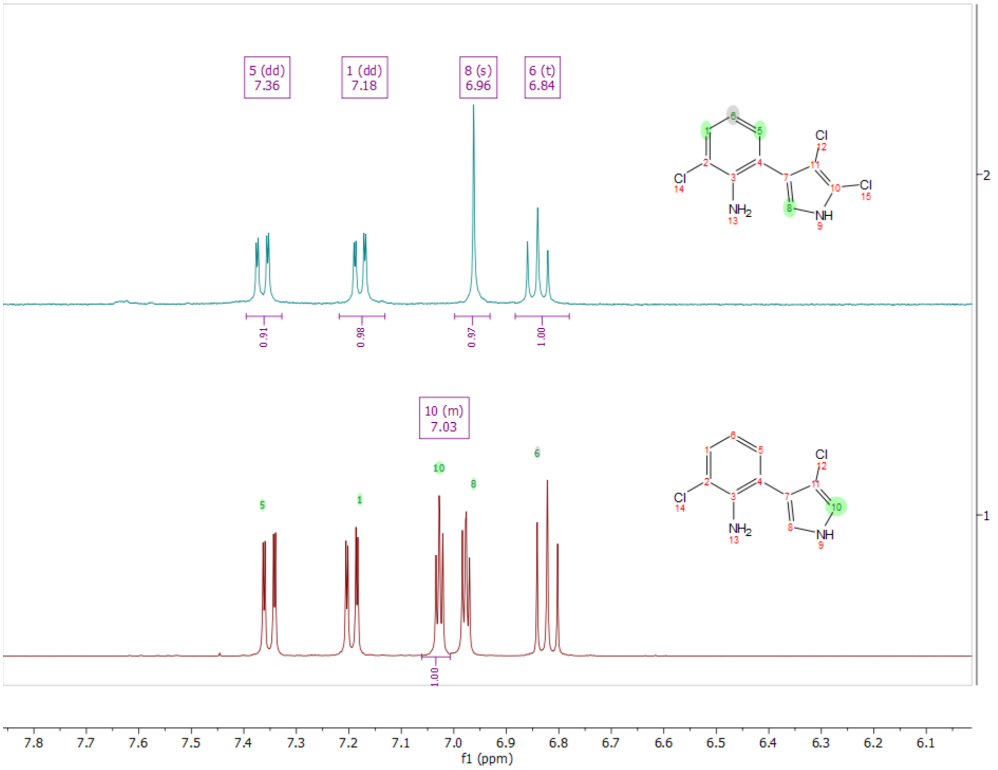

Figure S29. Comparative stacking of NMR spectrum for product **1** and 2Cl-**1**.

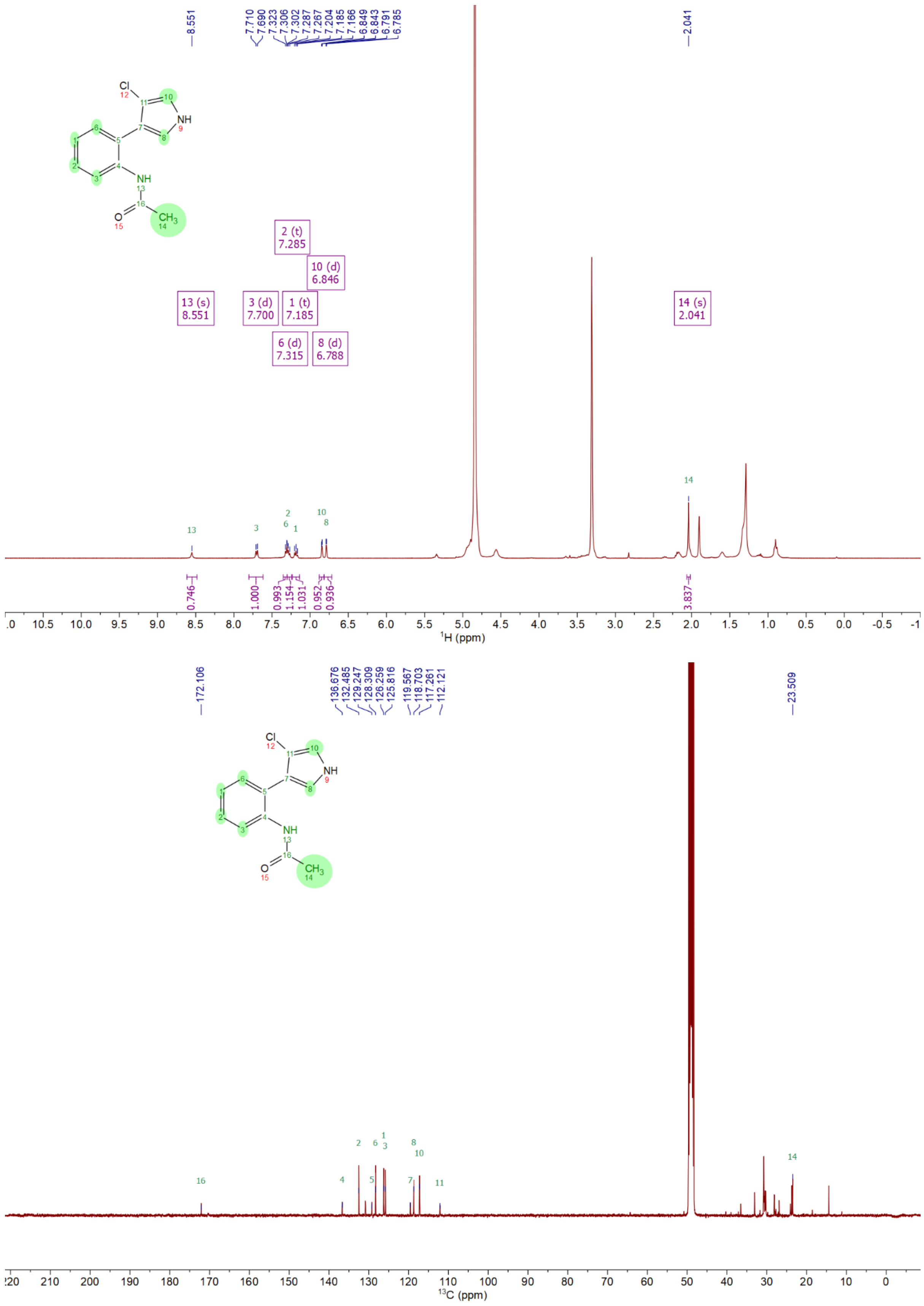

Figure S30. ^{1}H and ^{13}C NMR spectrum for product **2**

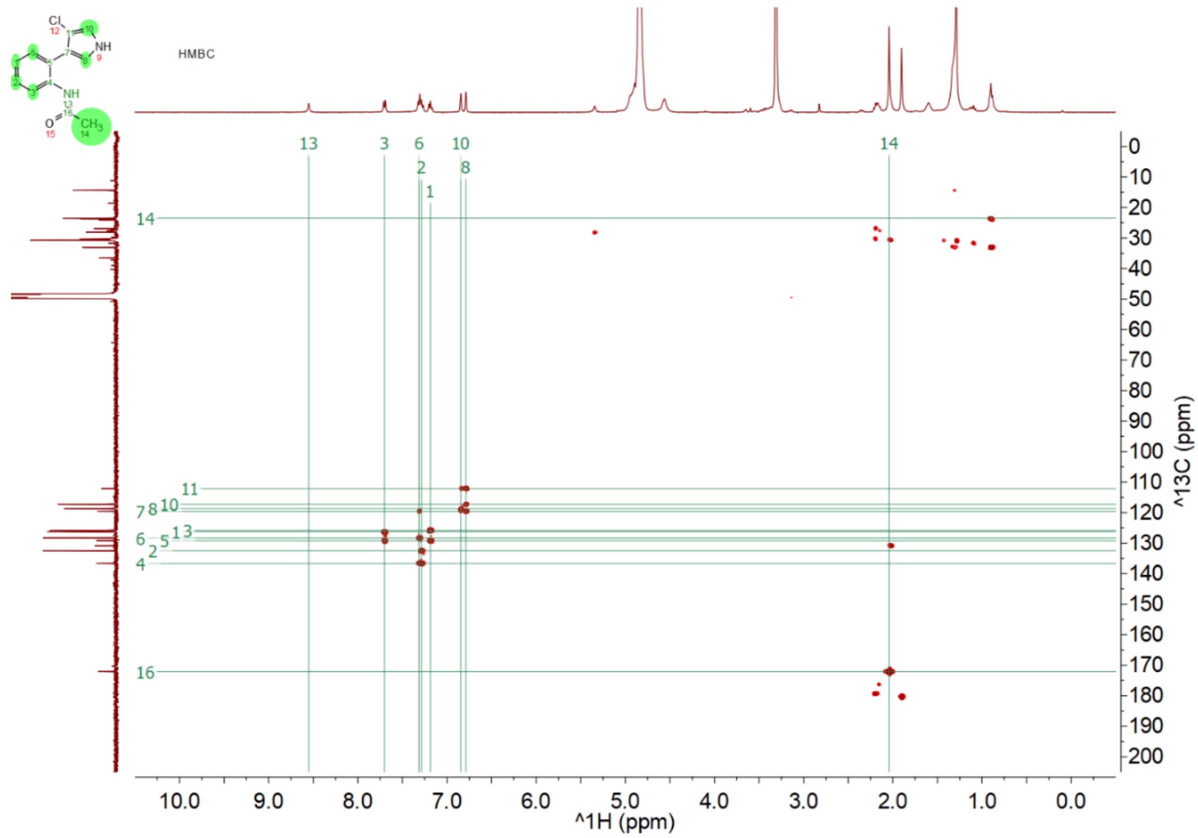

Figure S31. ^{1}H-^{13}C HMBC spectrum for product **2**

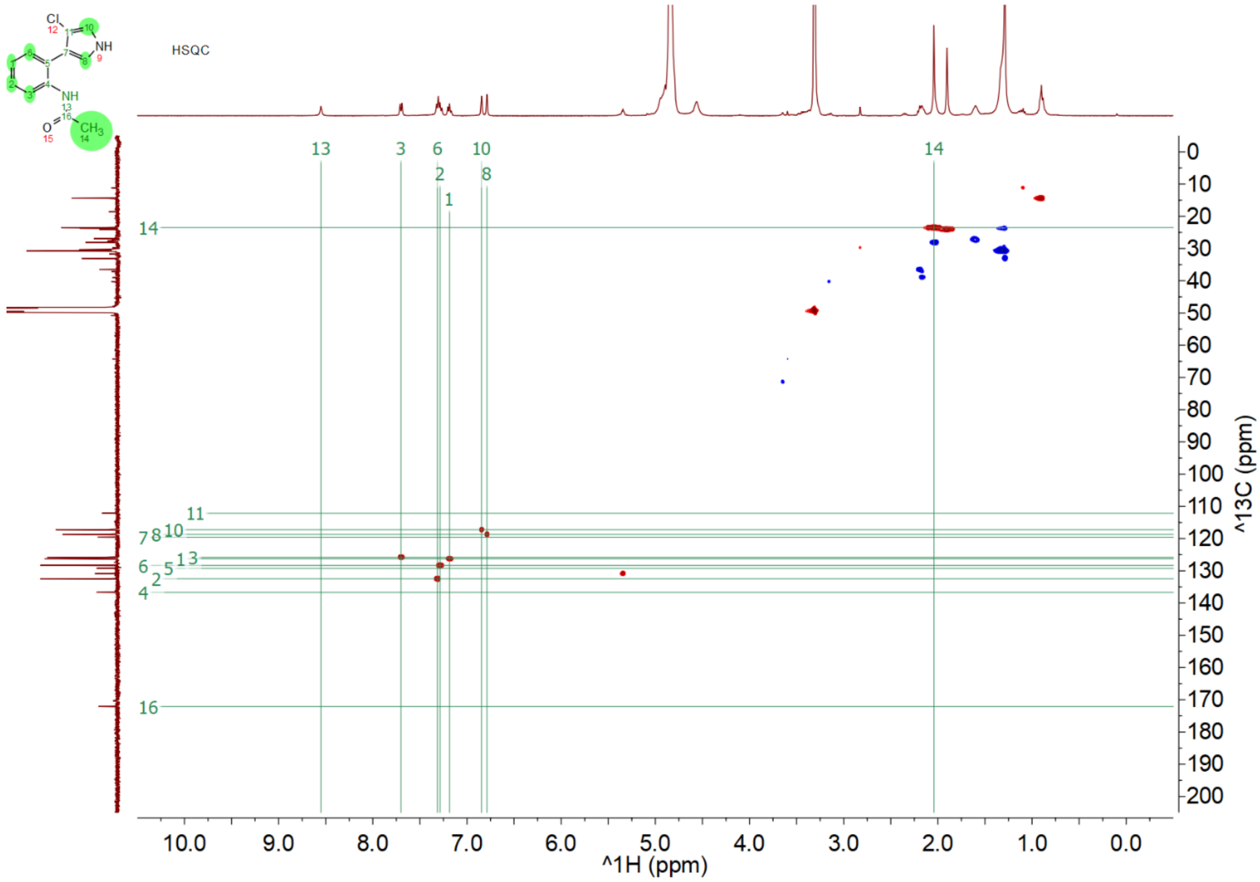

Figure S32. ^{1}H-^{13}C HSQC spectrum for product **2**

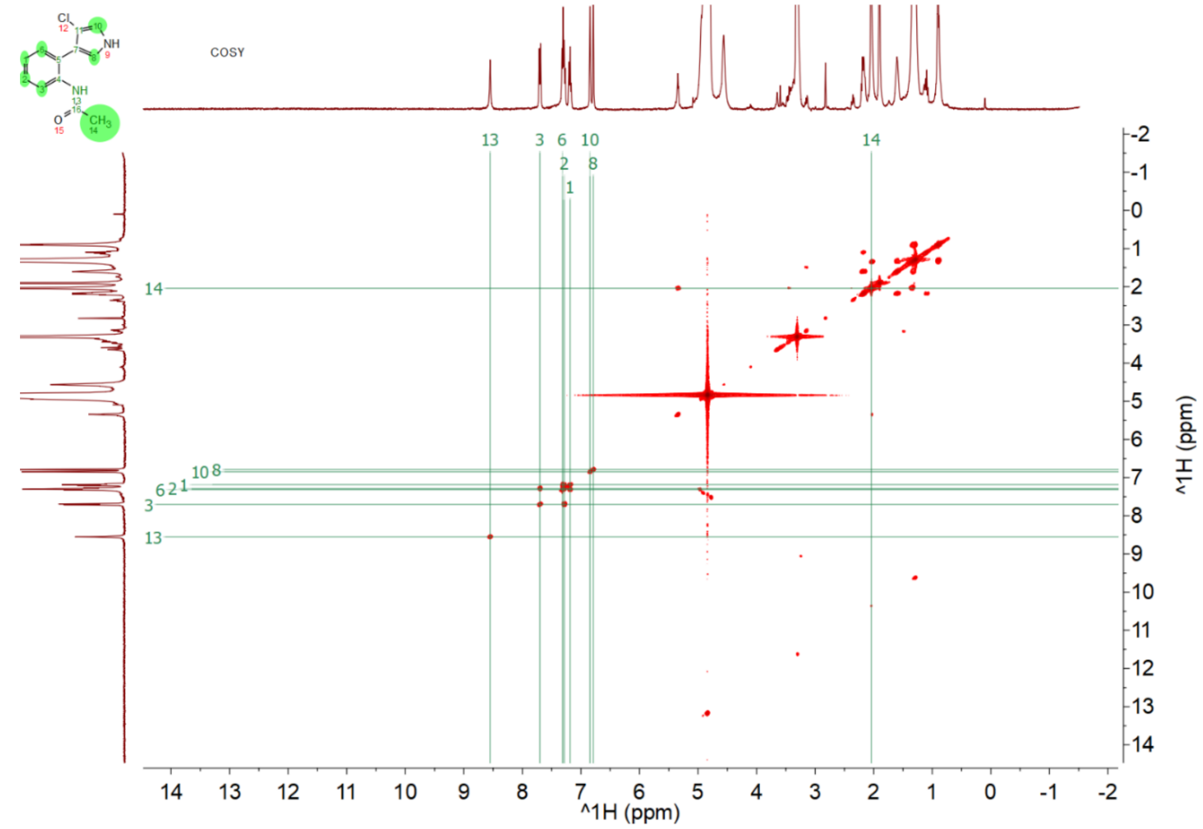

Figure S33. ^{1}H-^{1}H COSY spectrum for product **2**

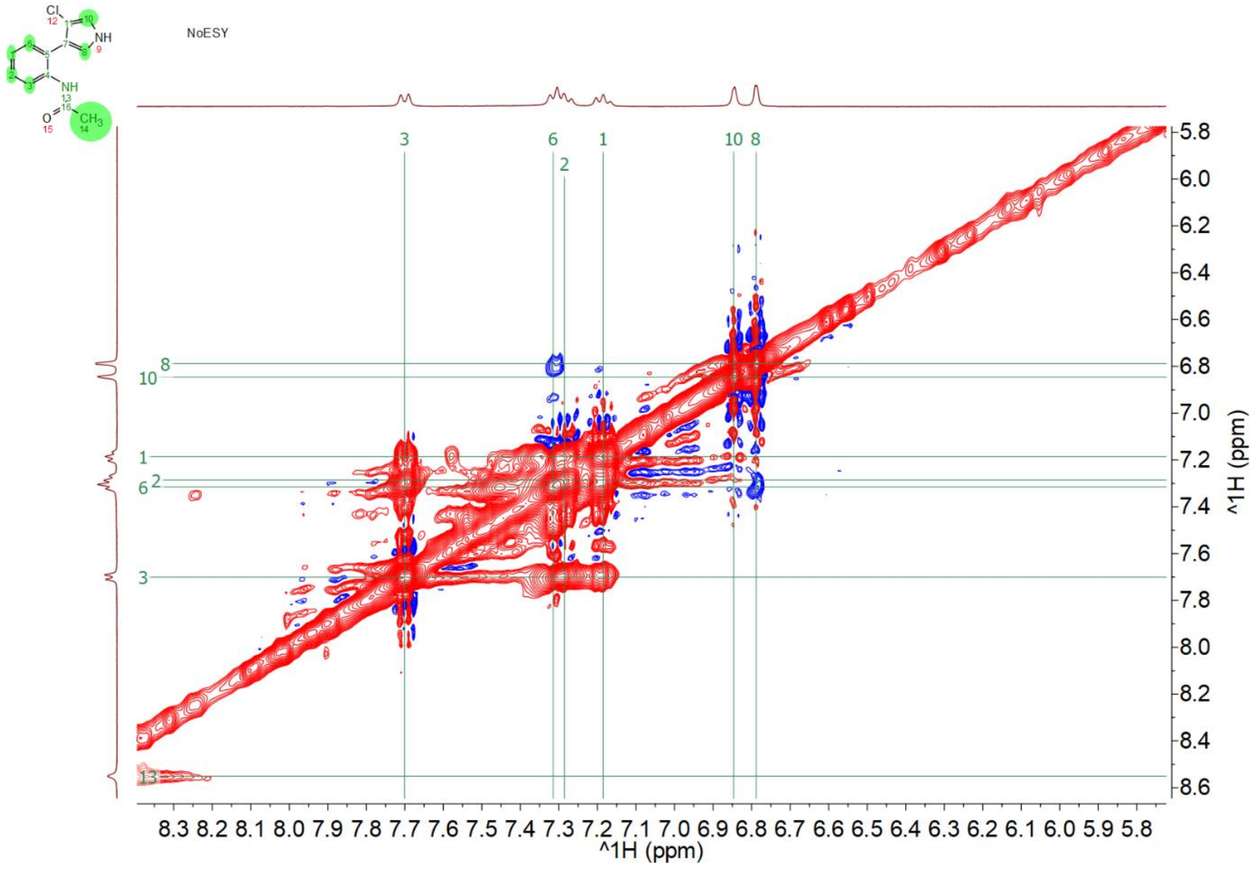

Figure S34. ^{1}H-^{1}H NOESY spectrum for product **2**

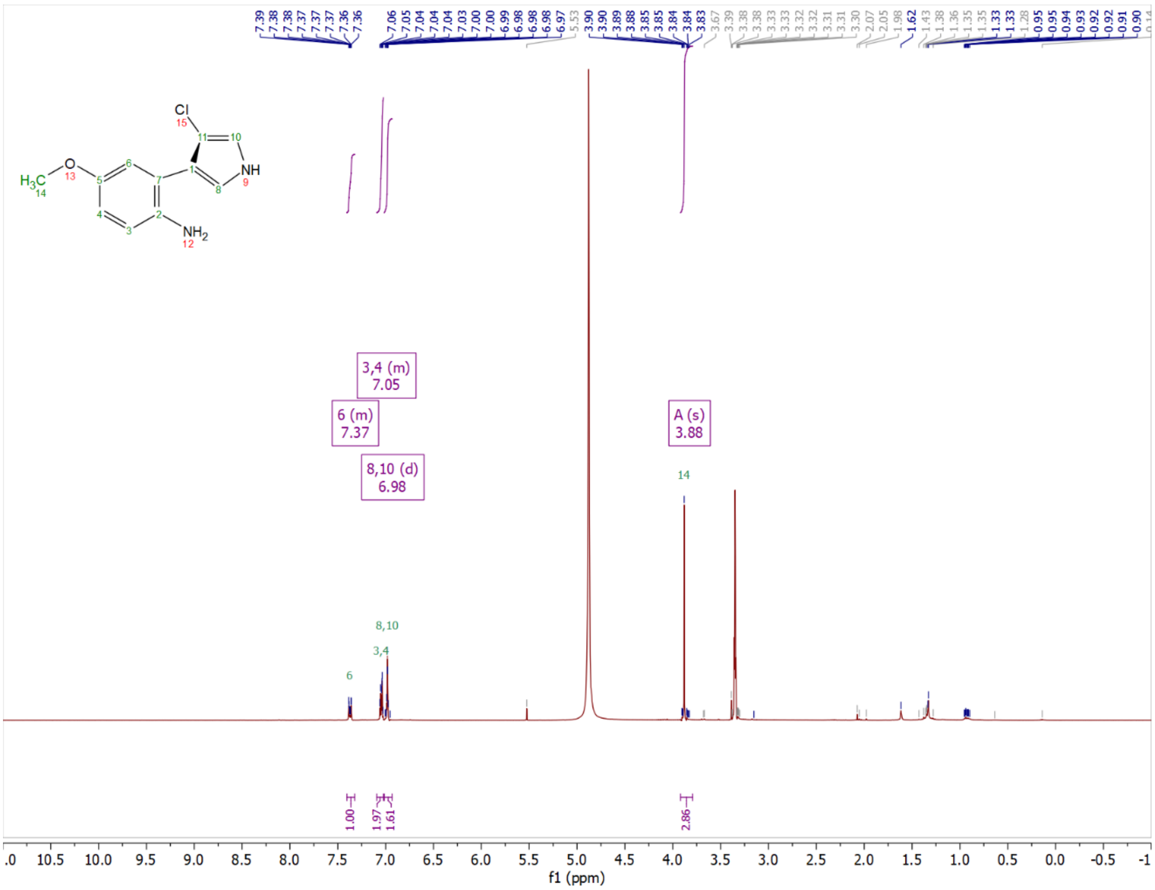

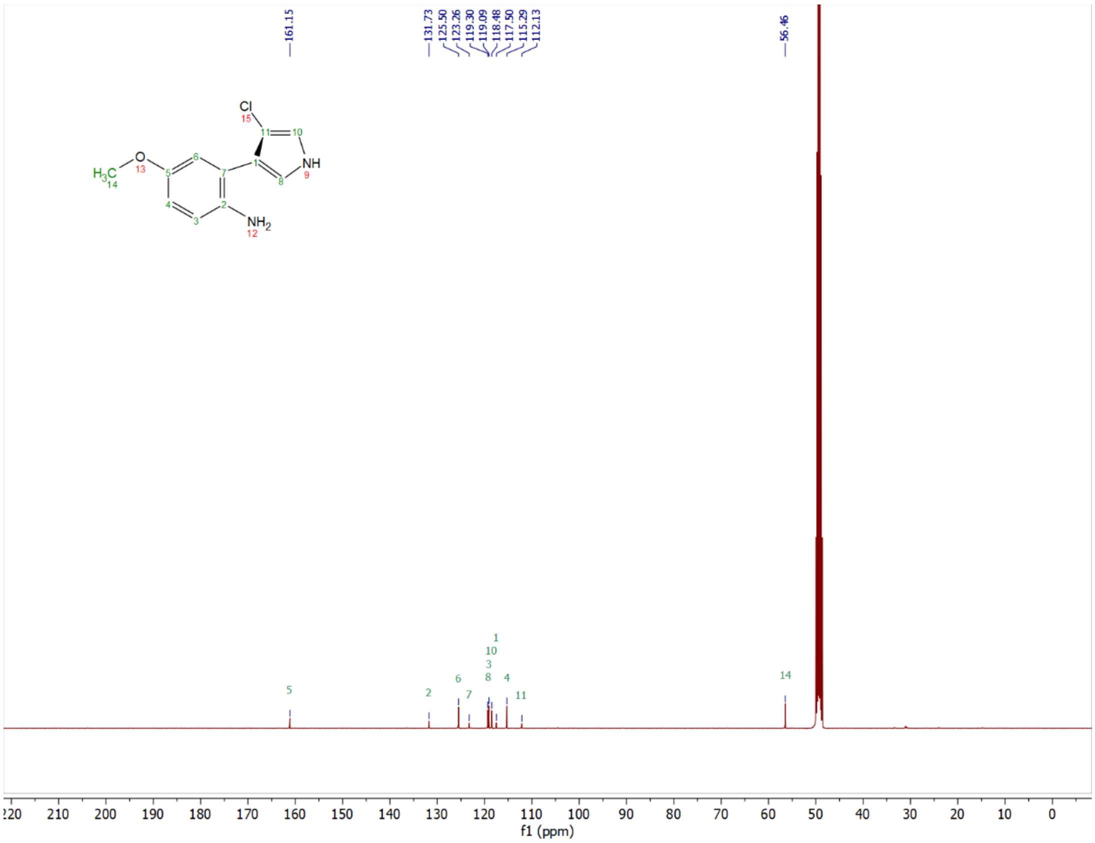

Figure S35. ^{1}H and ^{13}C NMR spectrum for product **3**

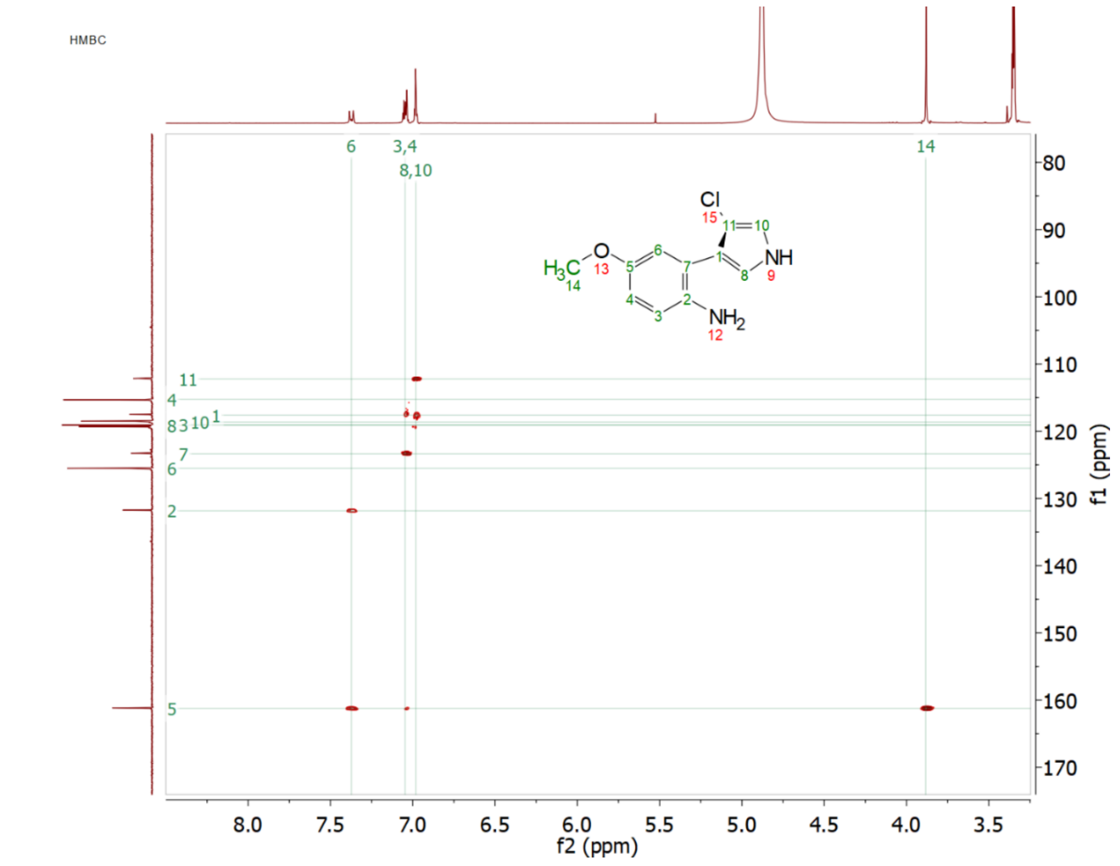

Figure S36. ^{1}H-^{13}C HMBC spectrum for product **3**

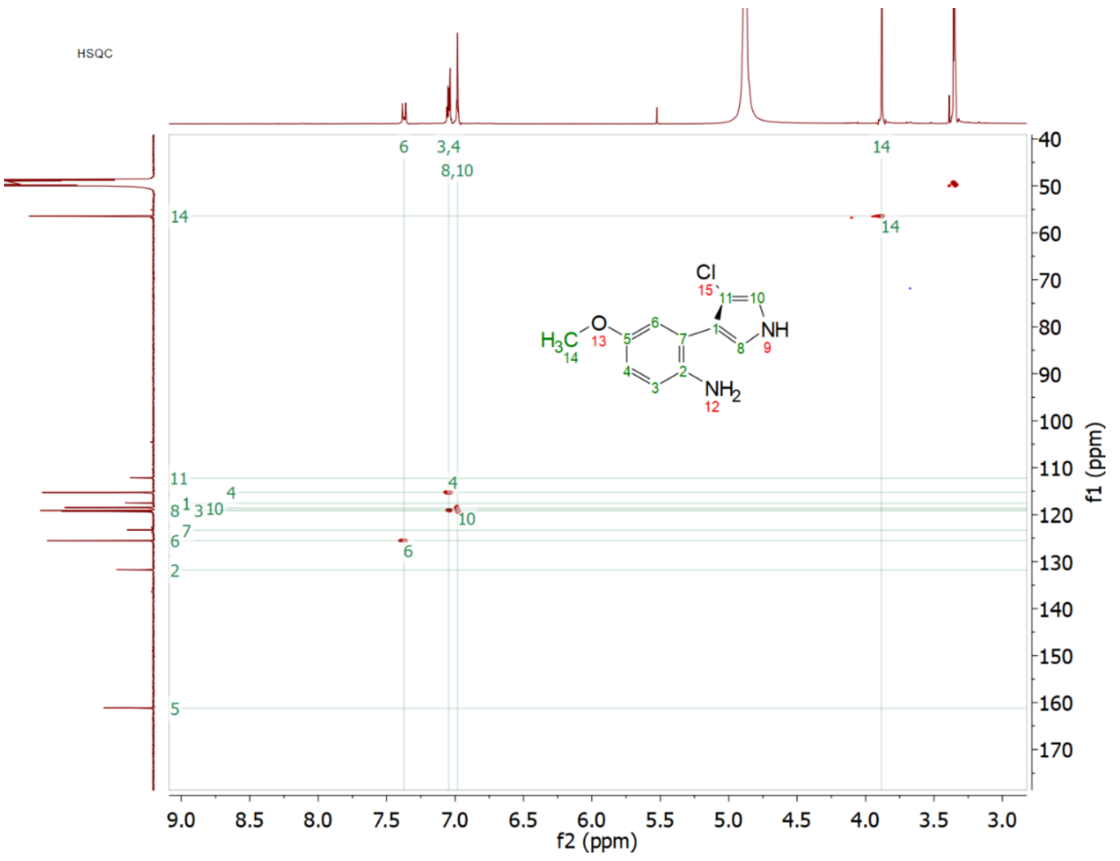

Figure S37. ^{1}H-^{13}C HSQC spectrum for product **3**

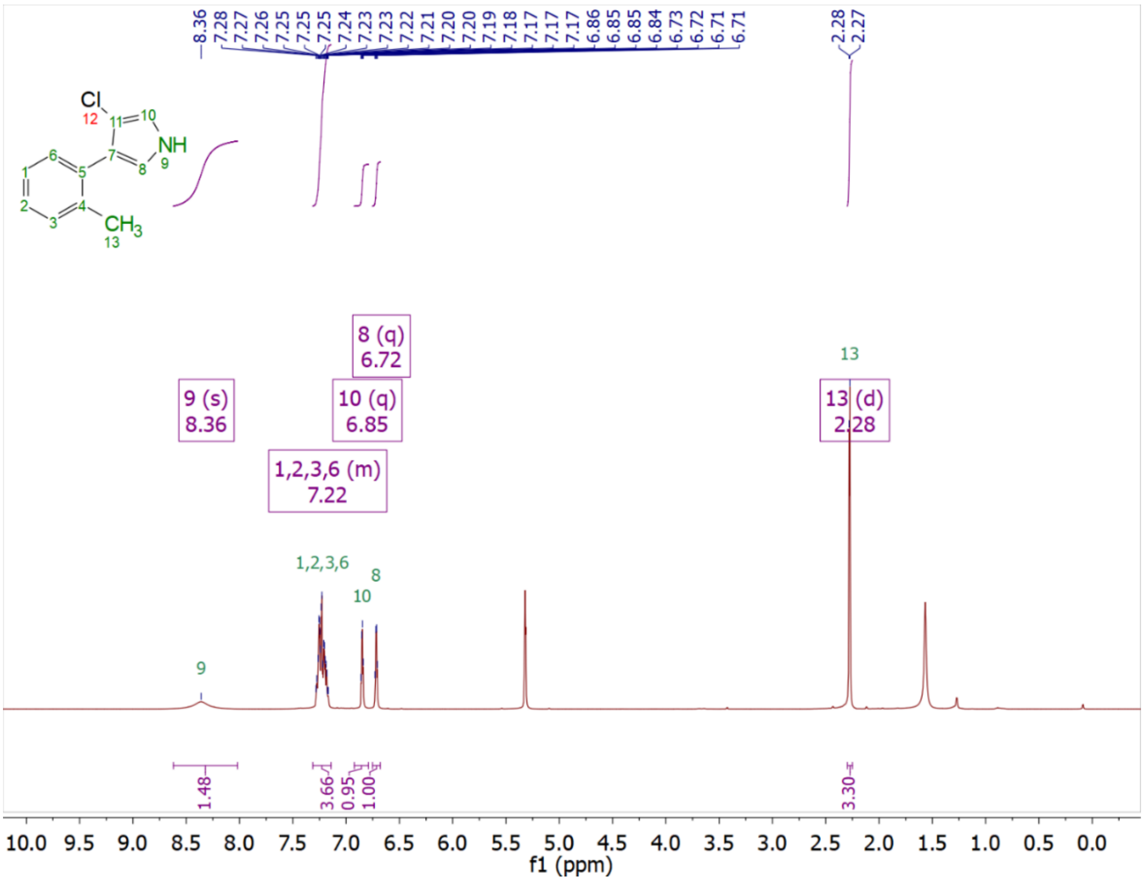

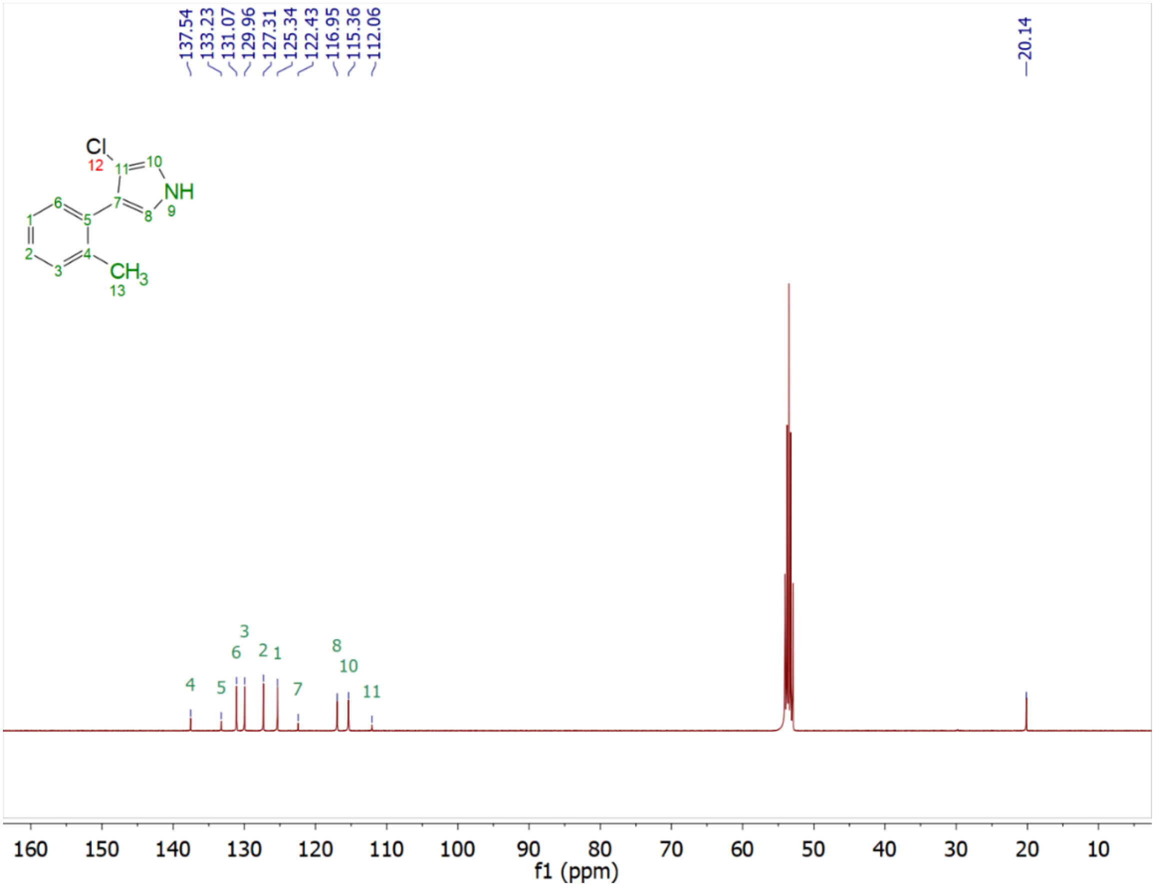

Figure S38. ^{1}H and ^{13}C NMR spectrum for product **4**

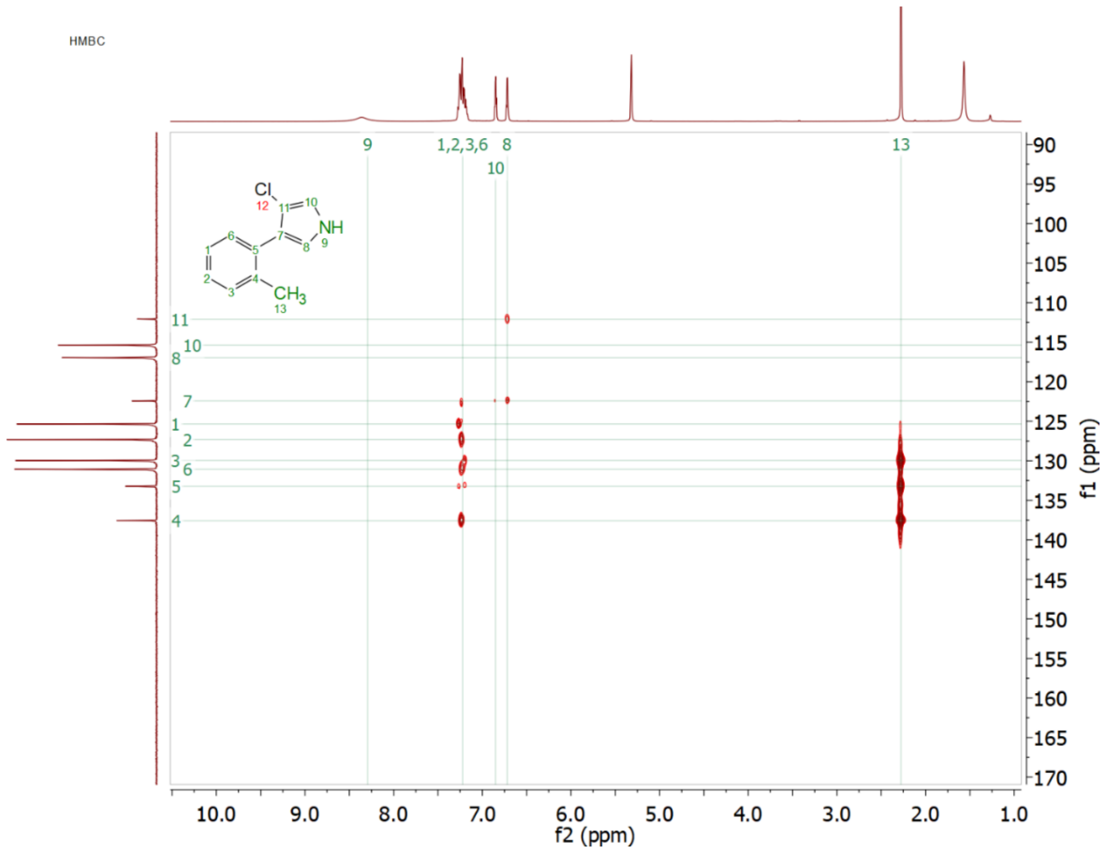

Figure S39. ^{1}H-^{13}C HMBC spectrum for product **4**

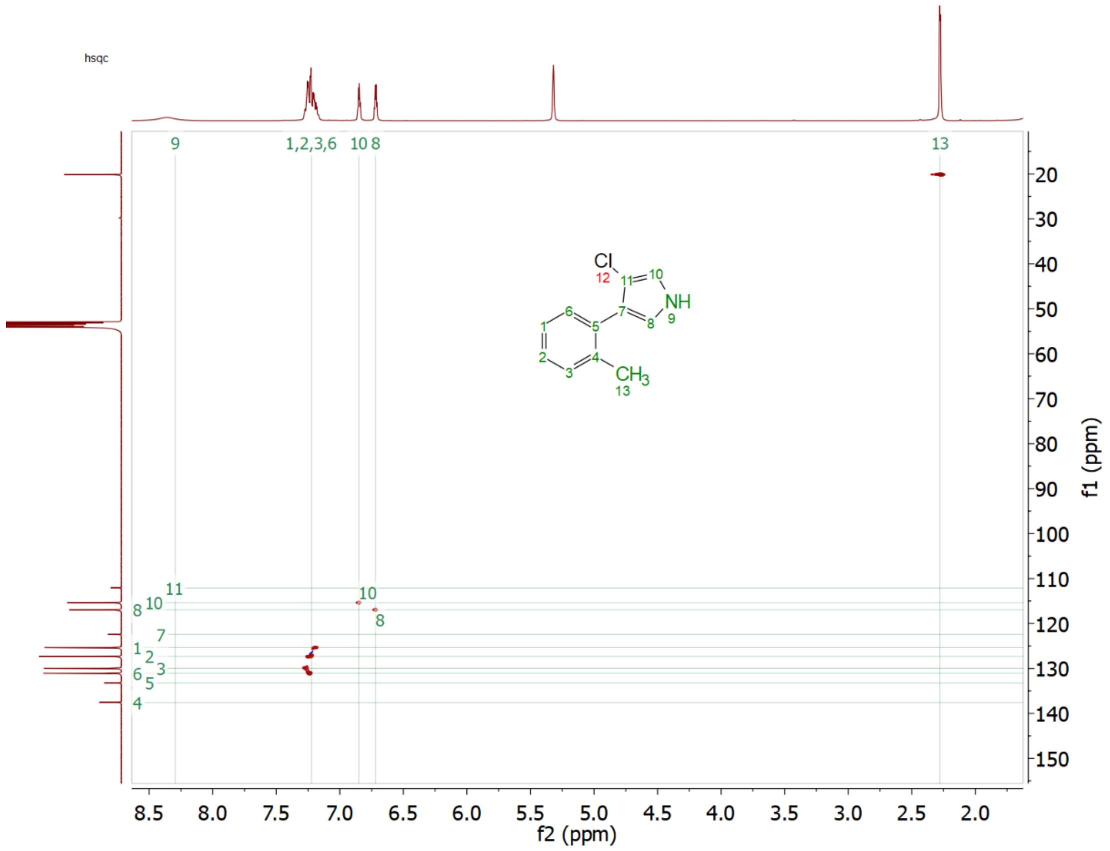

Figure S40. ^{1}H-^{13}C HSQC spectrum for product **4**

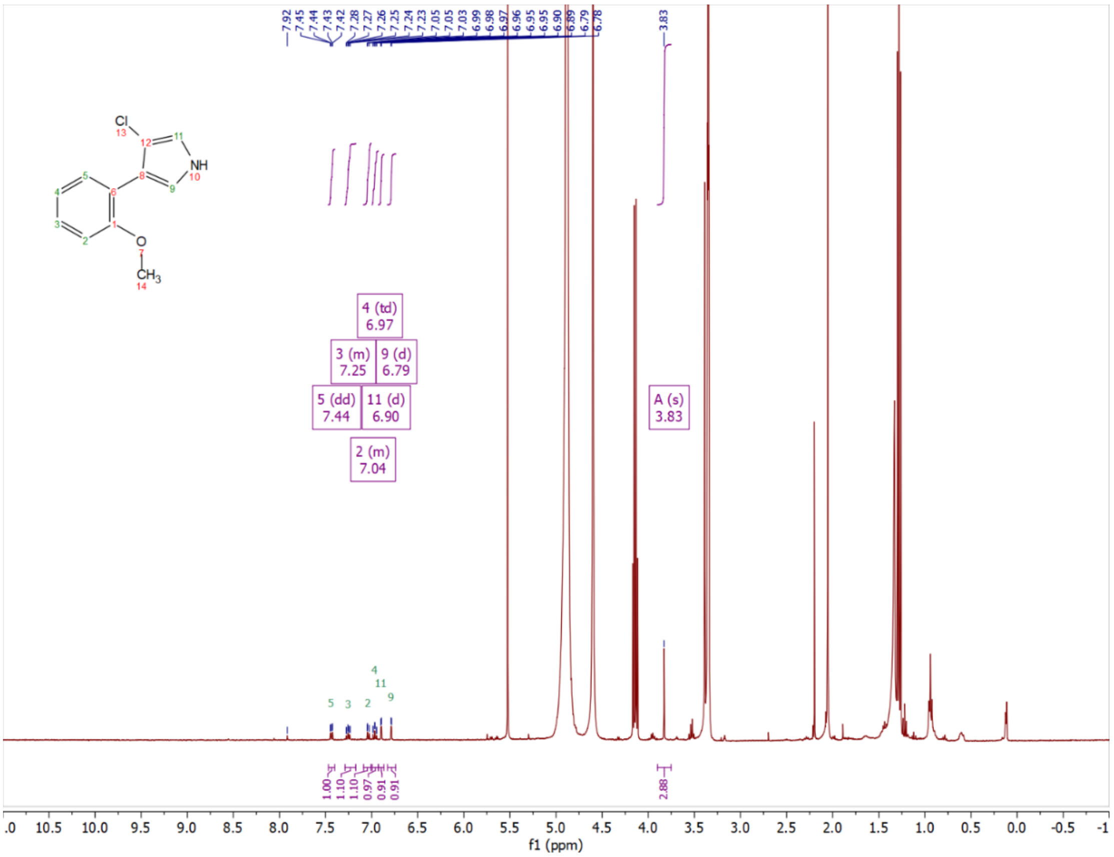

Figure S41. ^{1}H NMR spectrum for product **5**.

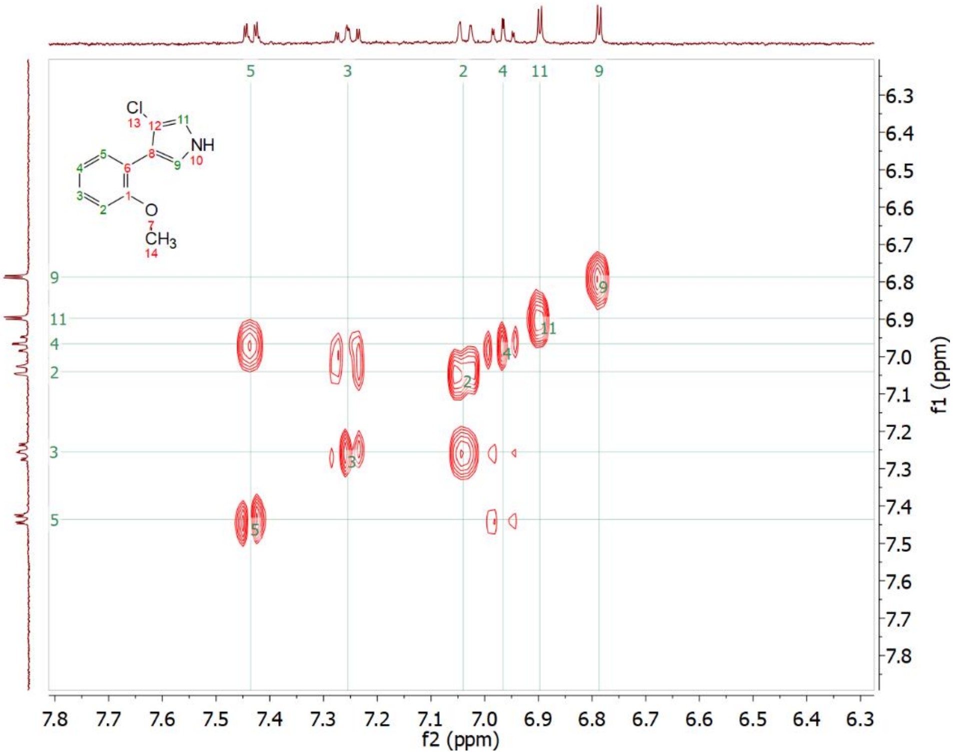

Figure S42. ^{1}H-^{1}H COSY NMR spectrum for product **5**.

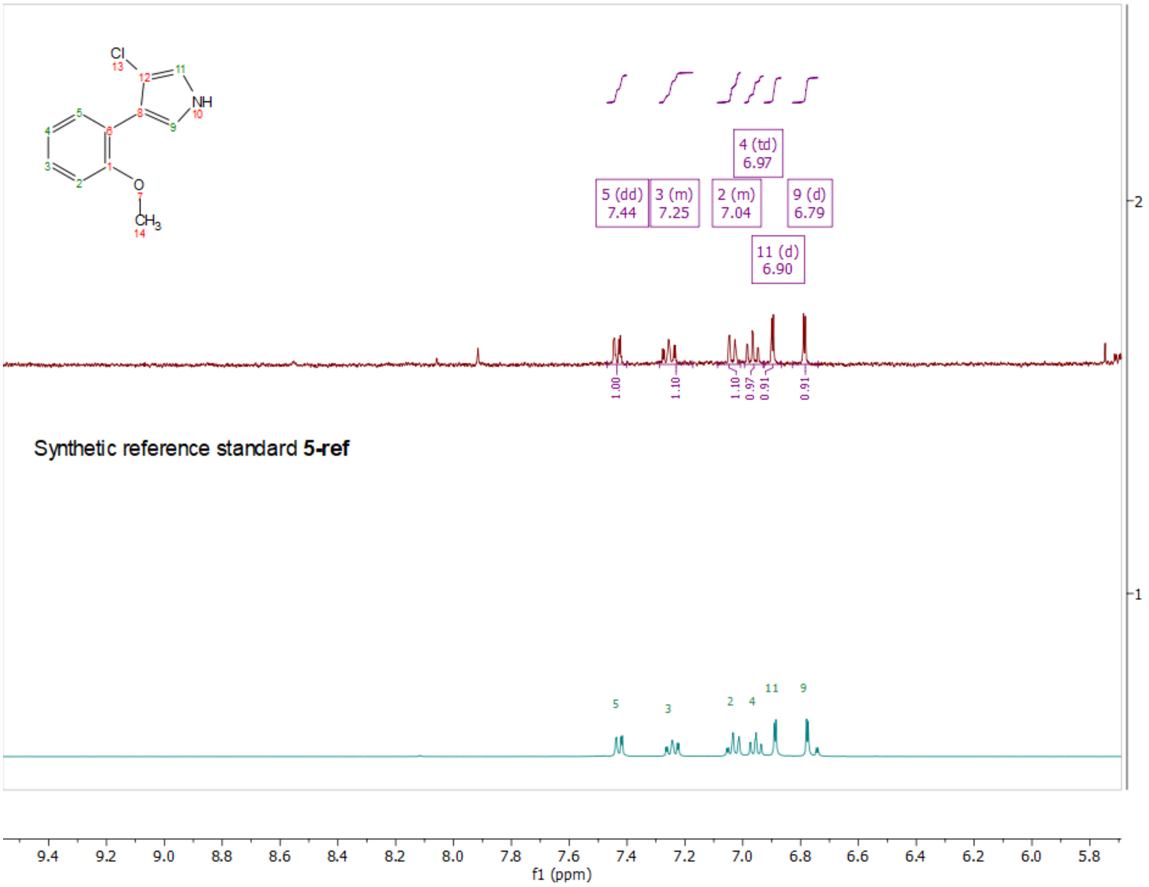

Figure S43. Comparative stacking of NMR spectrum for product **5** and synthetic reference standard **5-ref**

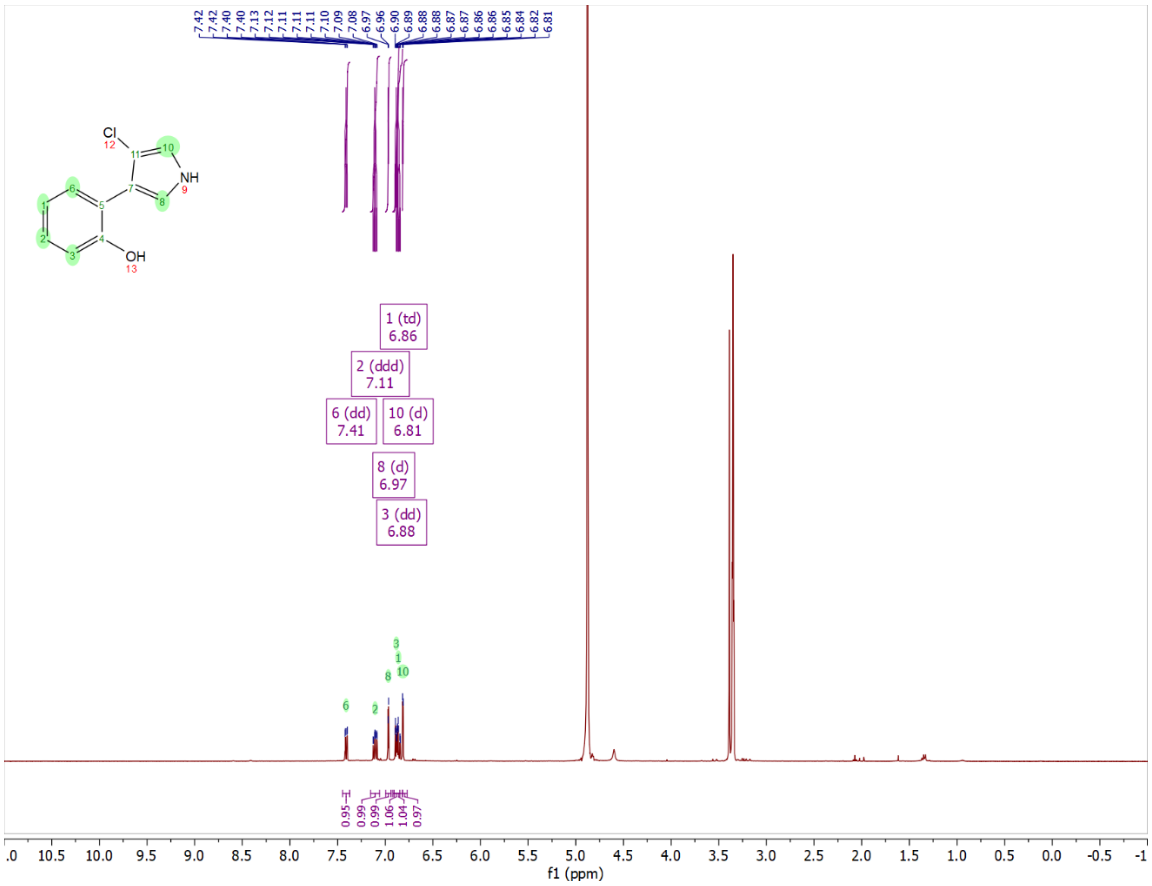

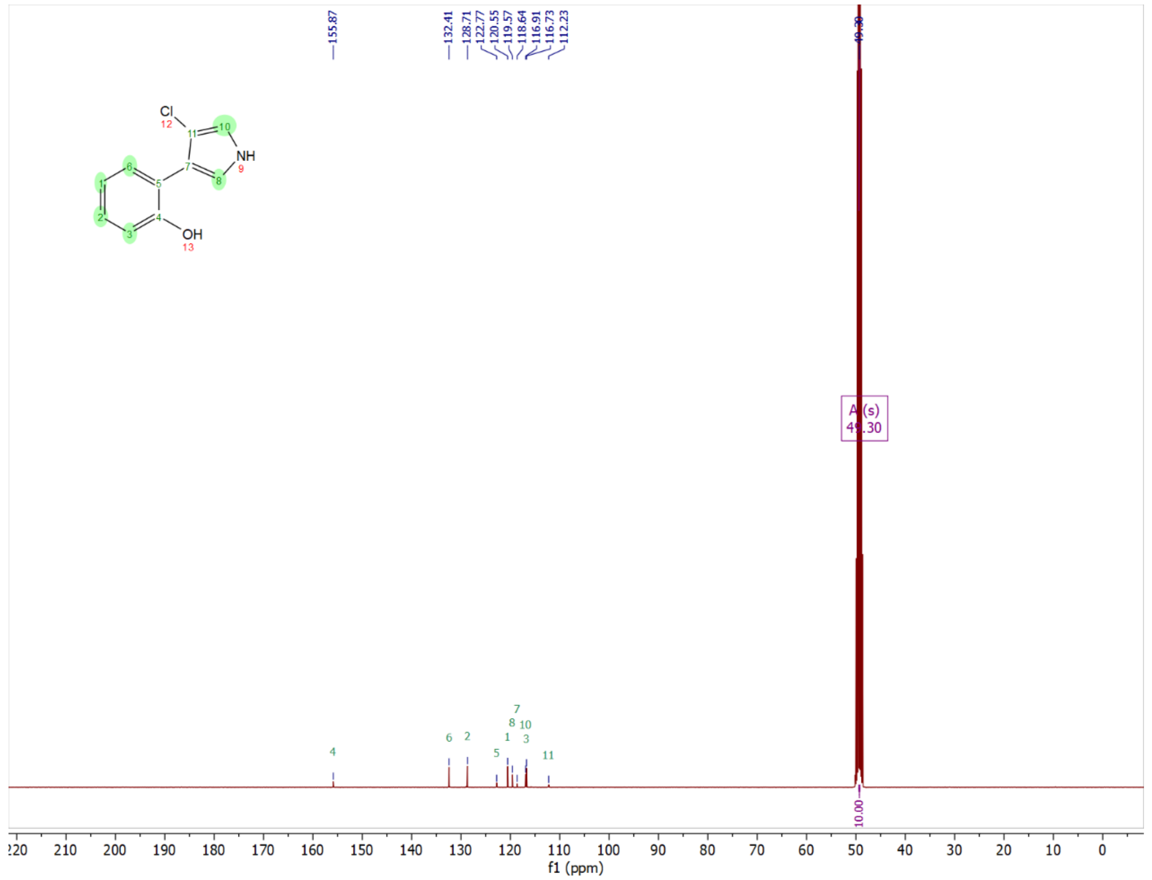

Figure S44. ^{1}H and ^{13}C NMR spectrum for product **6**.

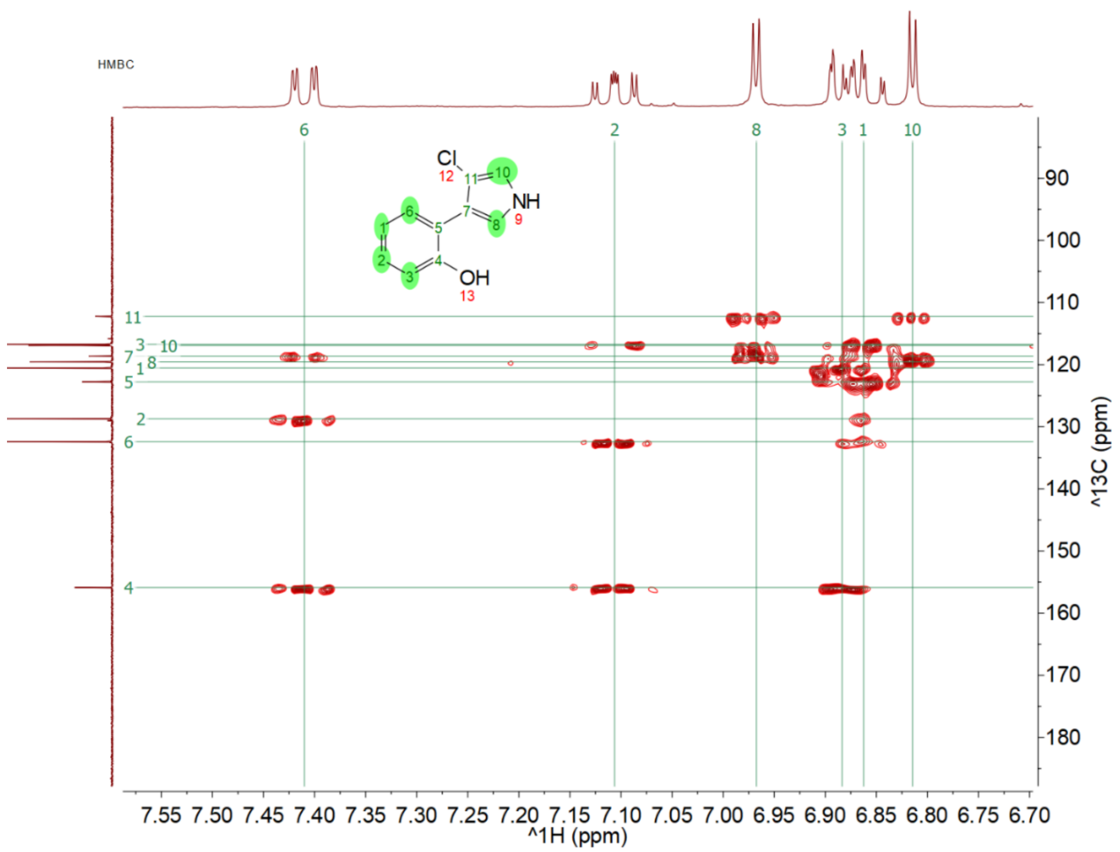

Figure S45. ^{1}H-^{13}C HMBC spectrum for product **6**

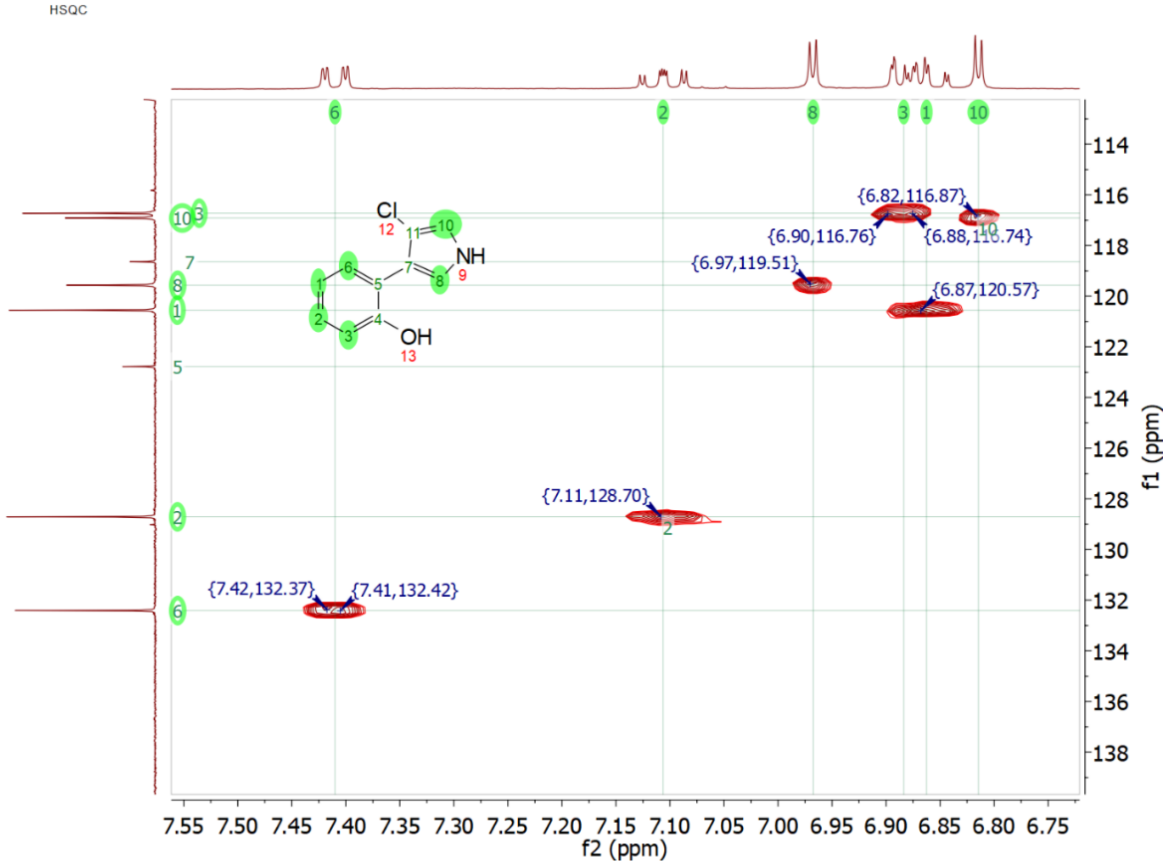

Figure S46. ^{1}H-^{13}C HSQC spectrum for product **6**

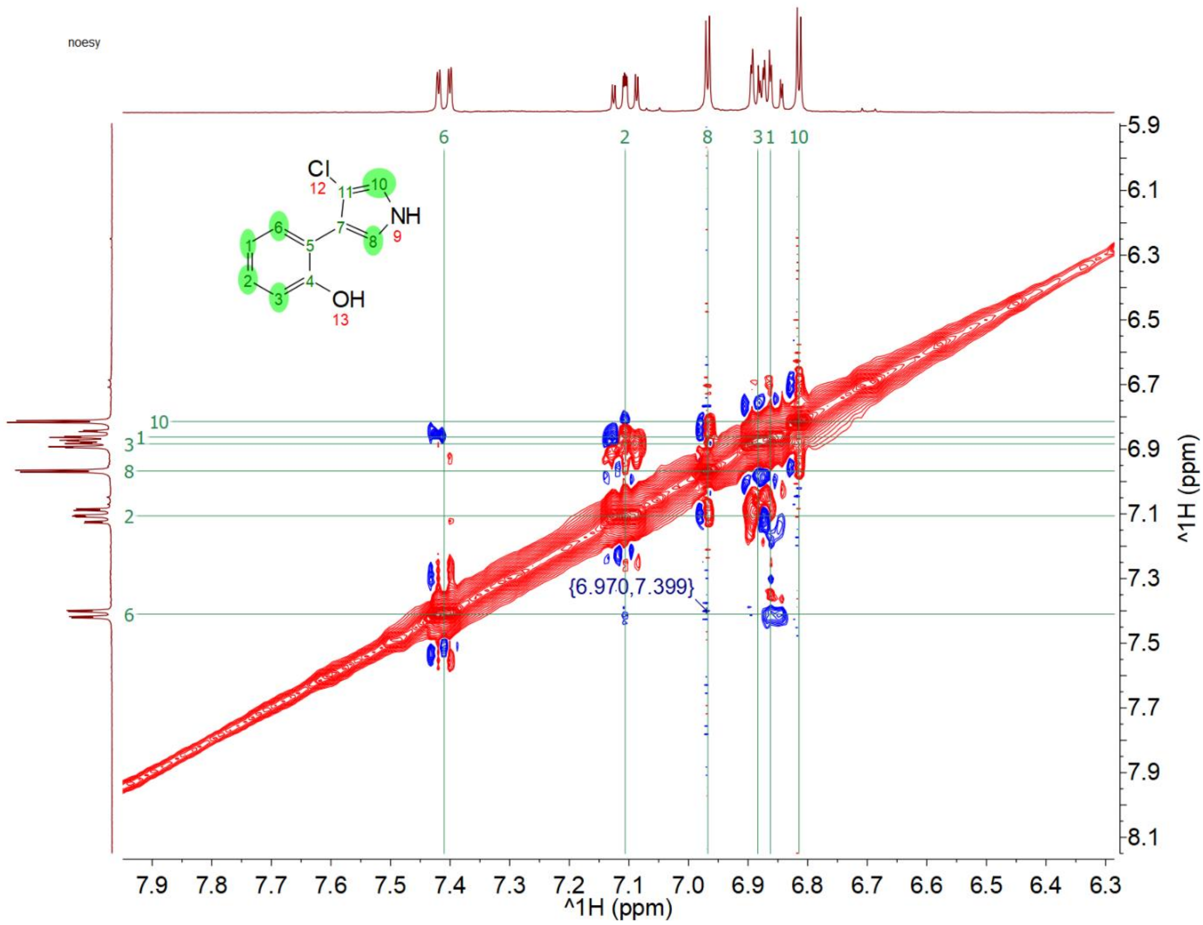

Figure S47. ^{1}H-^{1}H NOESY spectrum for product **6**

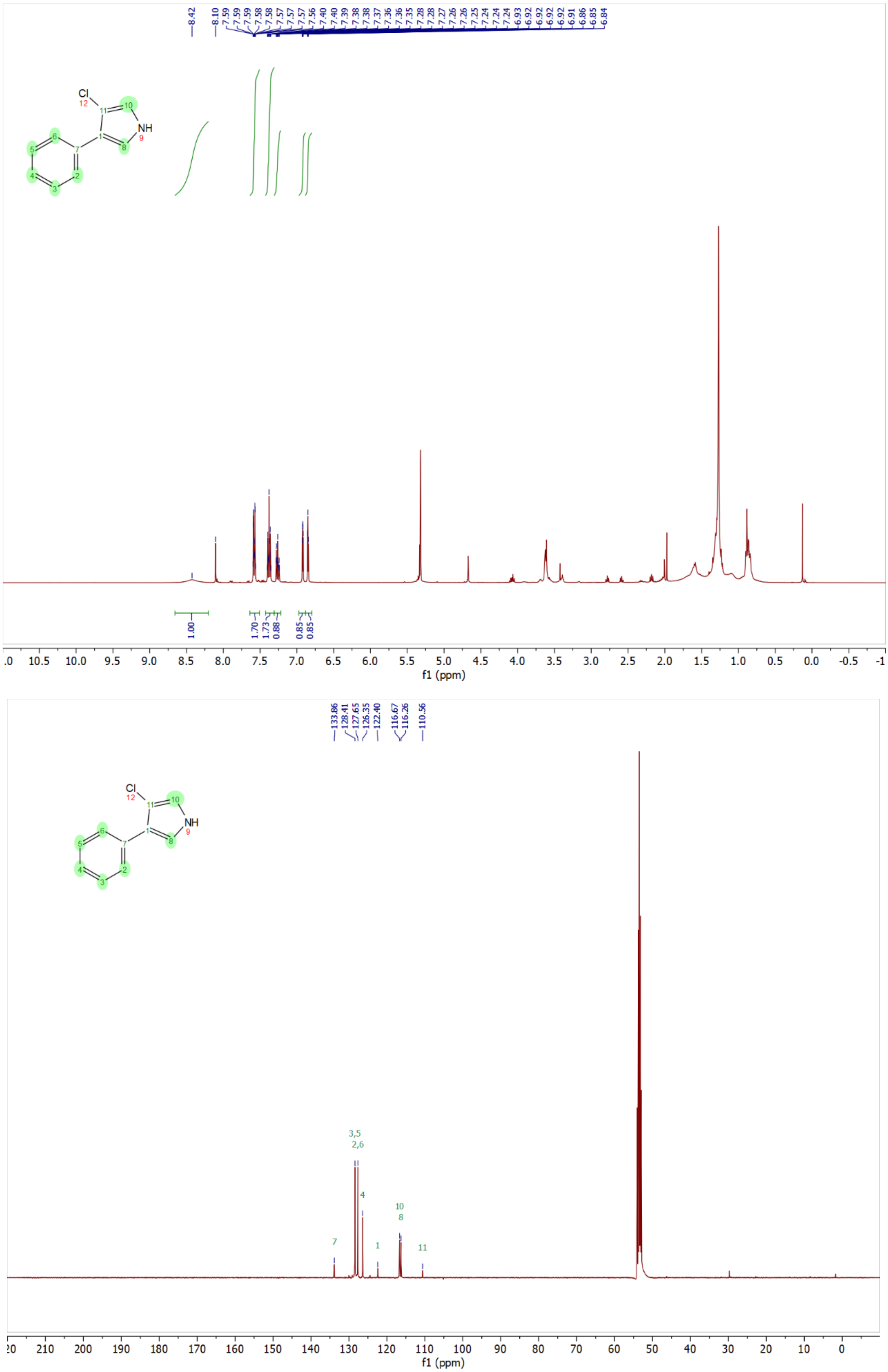

Figure S48. ^{1}H and ^{13}C NMR spectrum for product **7**

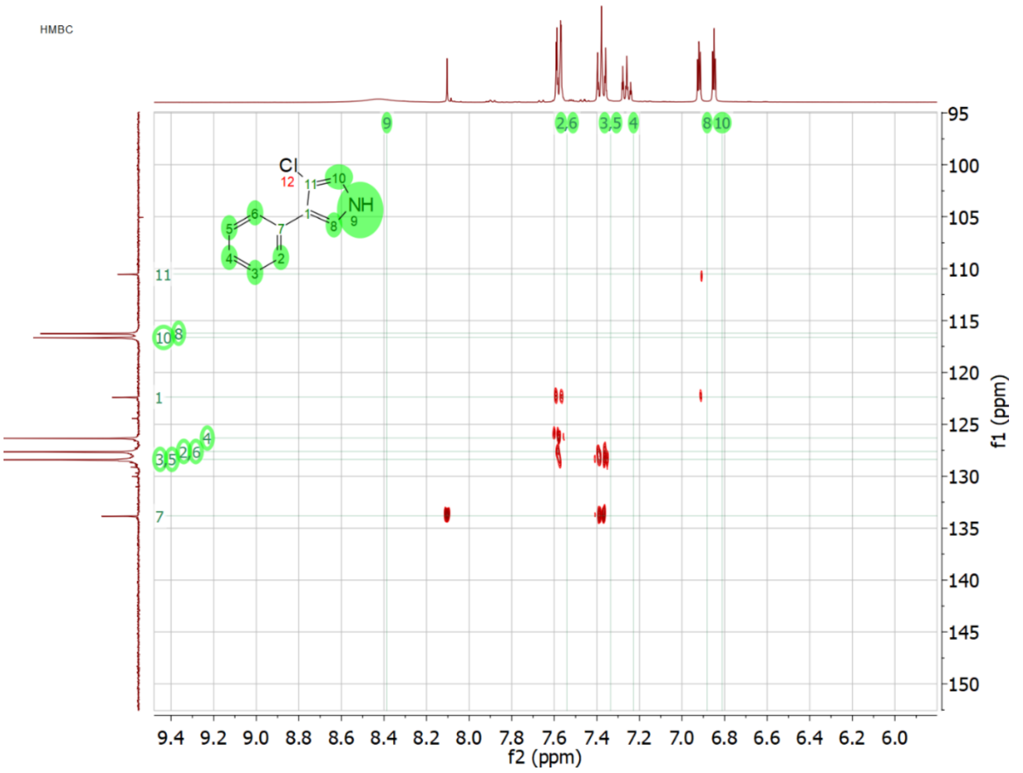

Figure S49. ^{1}H-^{13}C HMBC spectrum for product **7**

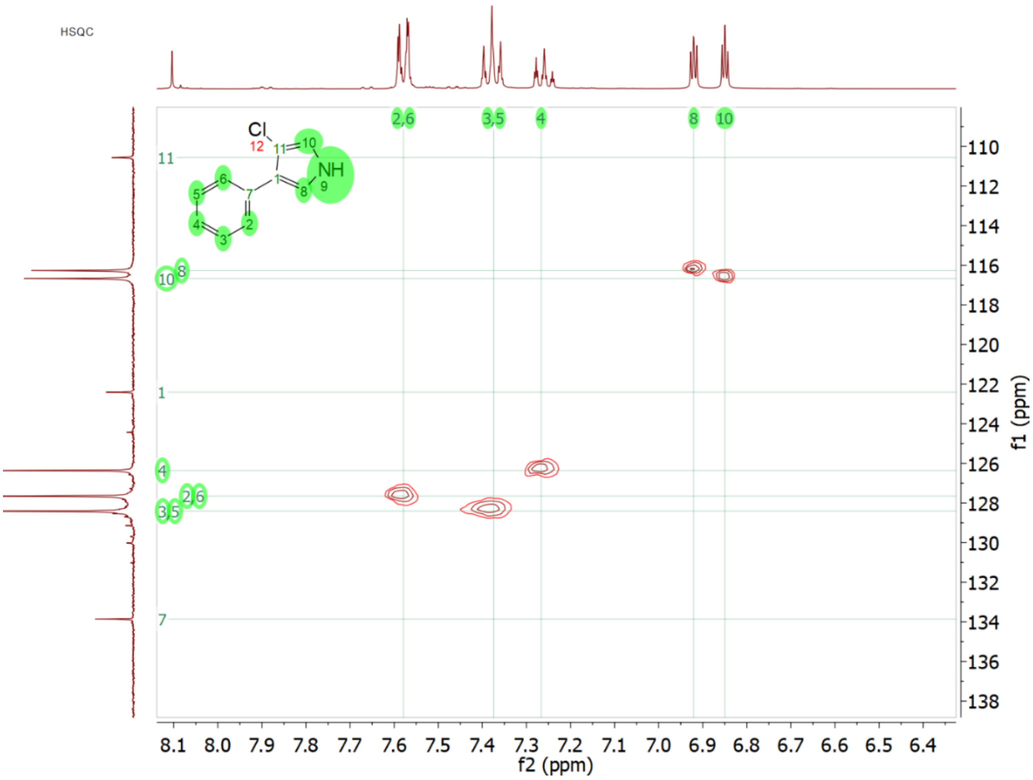

Figure S50. ^{1}H-^{13}C HSQC spectrum for product **7**

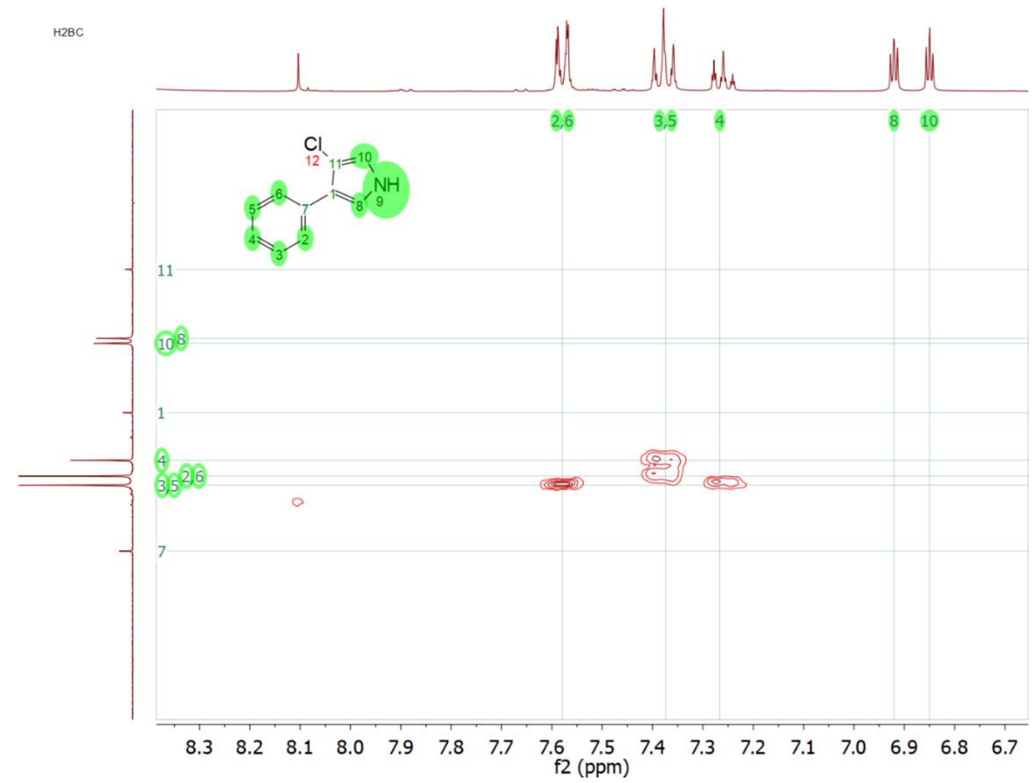

Figure S51. ^{1}H-^{13}C H2BC spectrum for product **7**

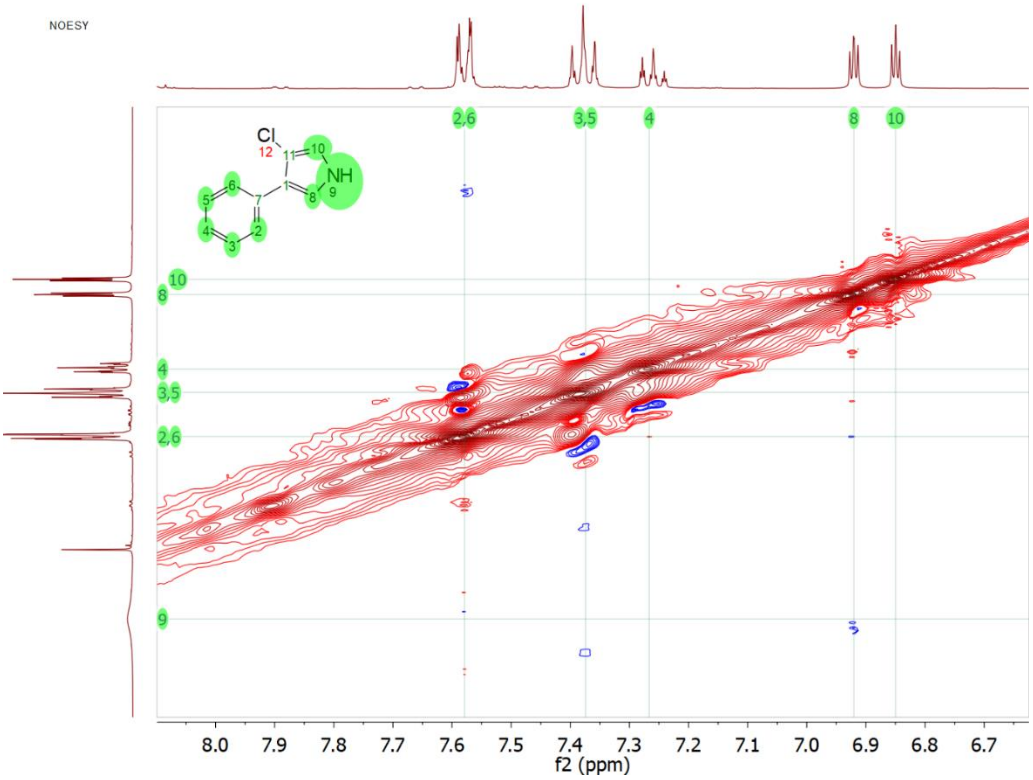

Figure S52. ^{1}H-^{1}H NOESY spectrum for product **7**

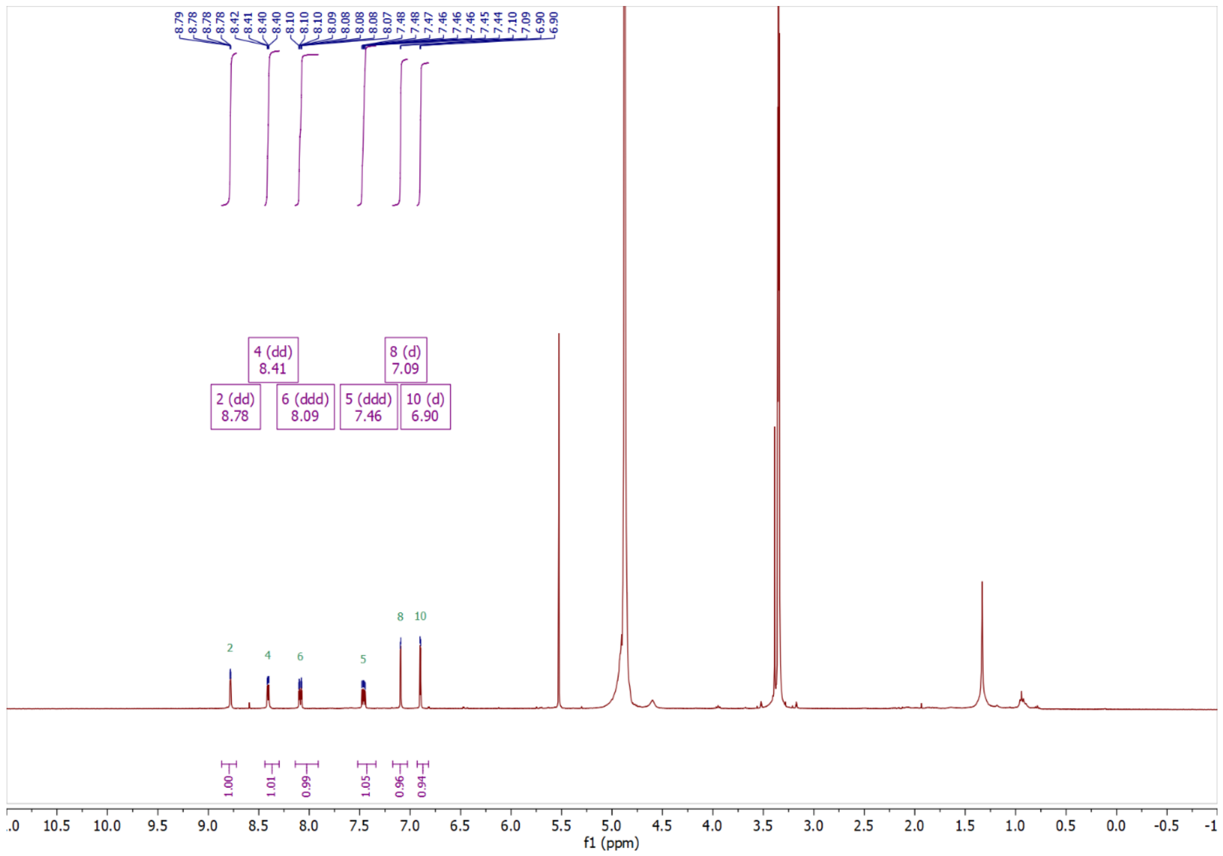

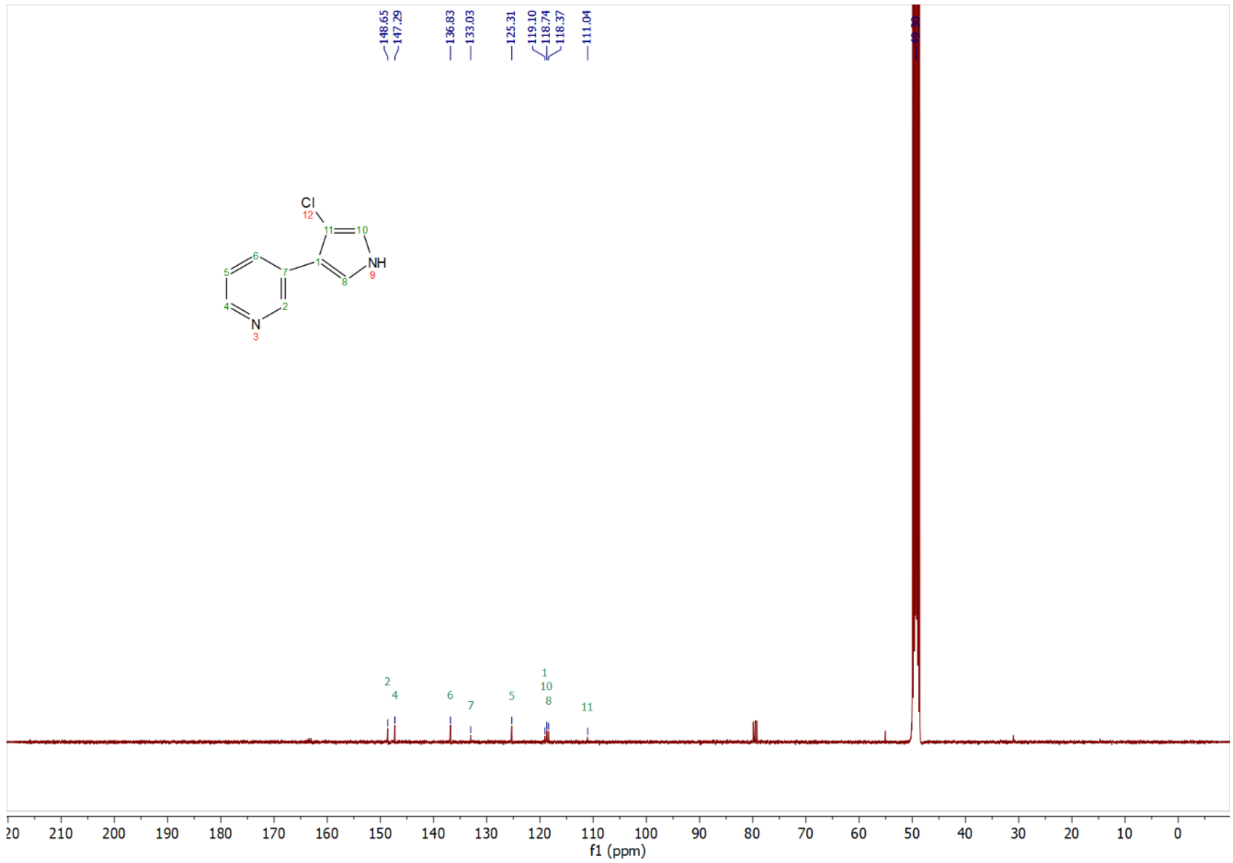

Figure S53. ^{1}H and ^{13}C NMR spectrum for product **8**.

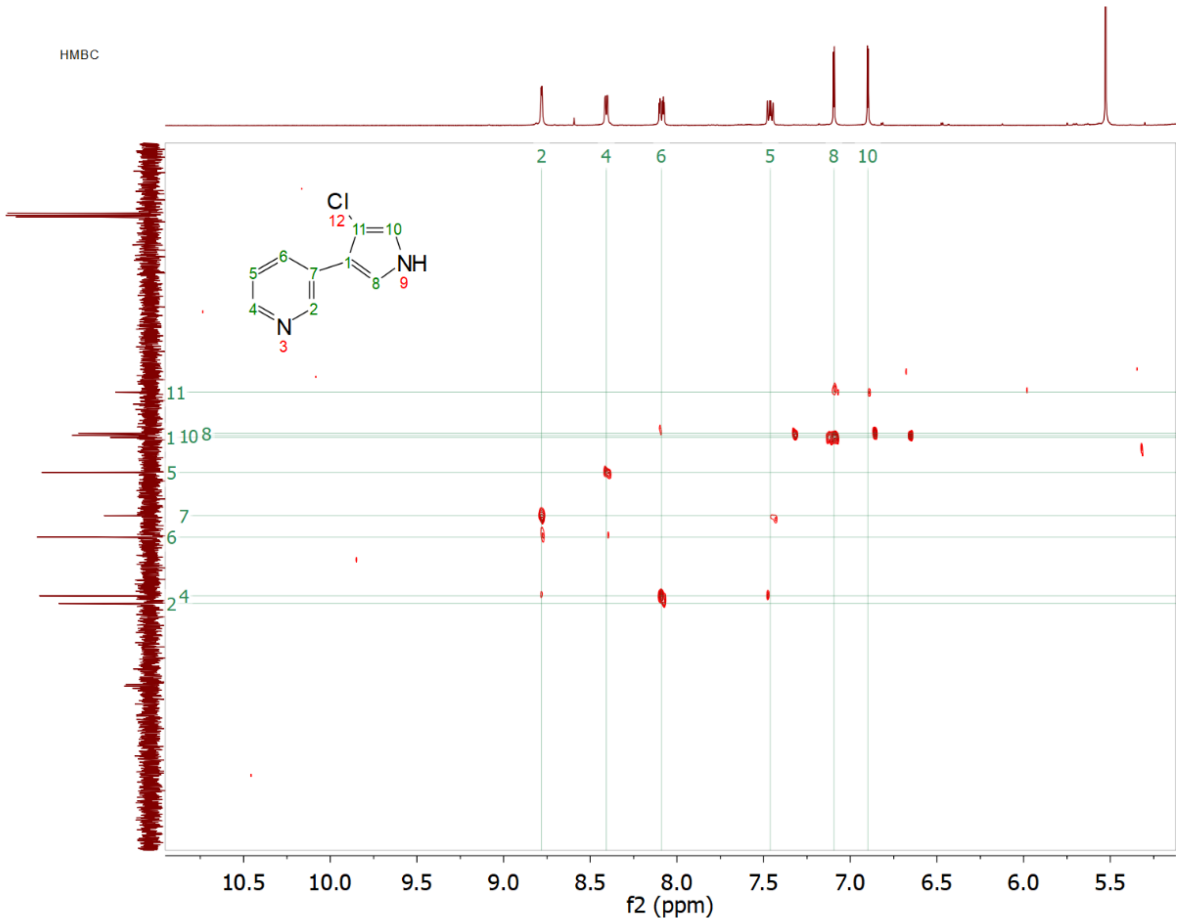

Figure S54. ^{1}H-^{13}C HMBC NMR spectrum for product **8**.

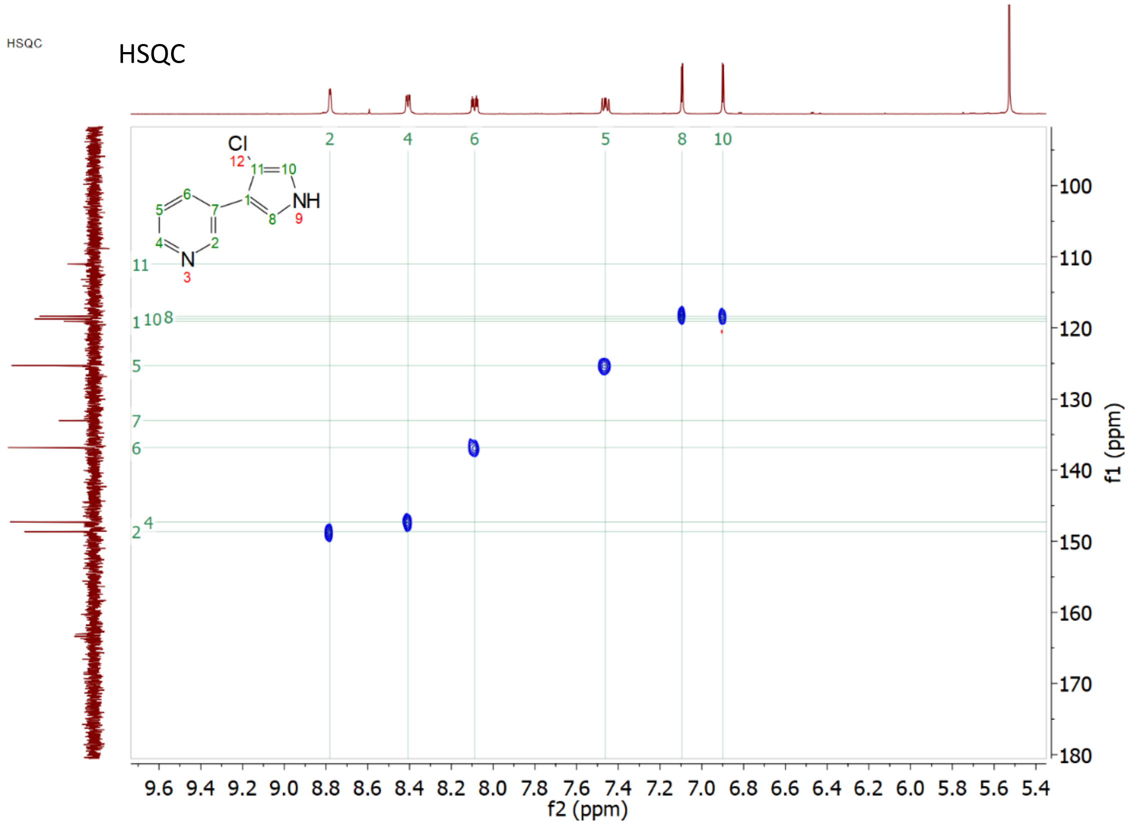

Figure S55. ^{1}H-^{13}C HSQC NMR spectrum for product **8**.

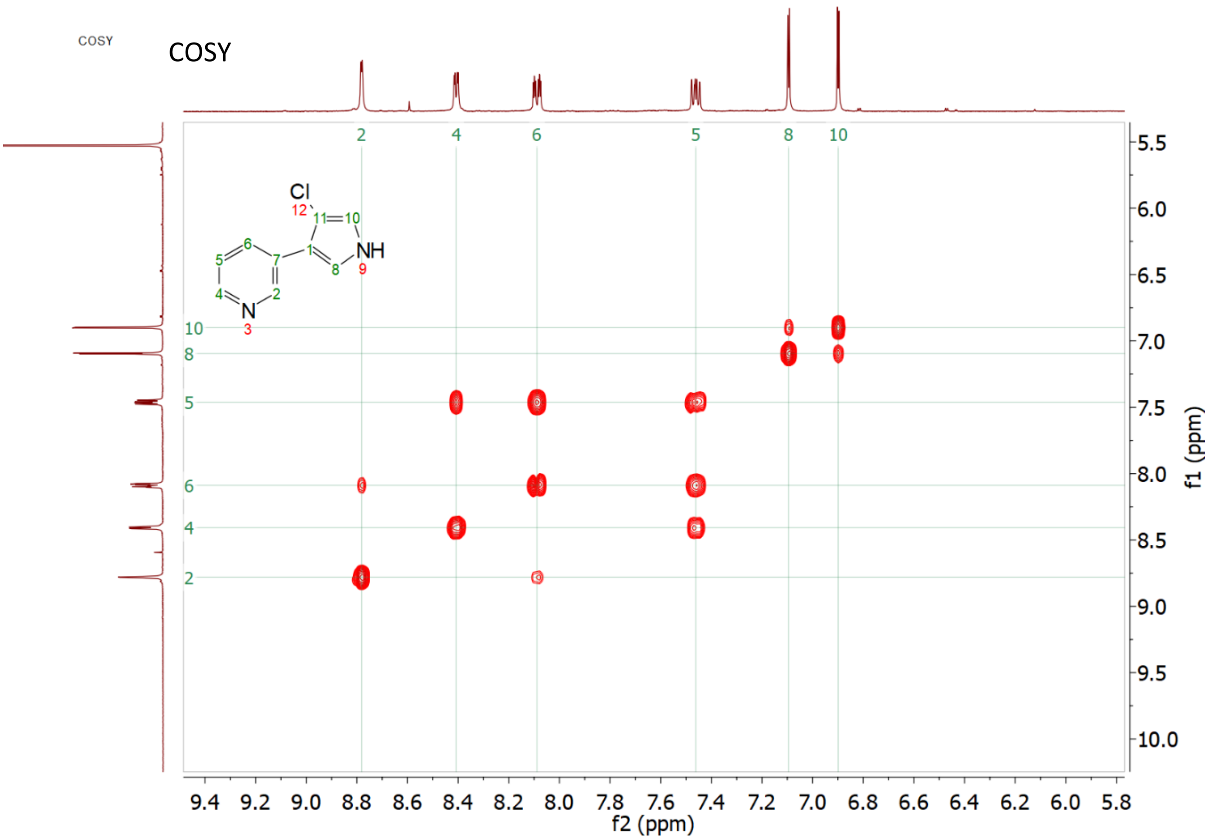

Figure S56. ^{1}H-^{1}H COSY NMR spectrum for product **8**.

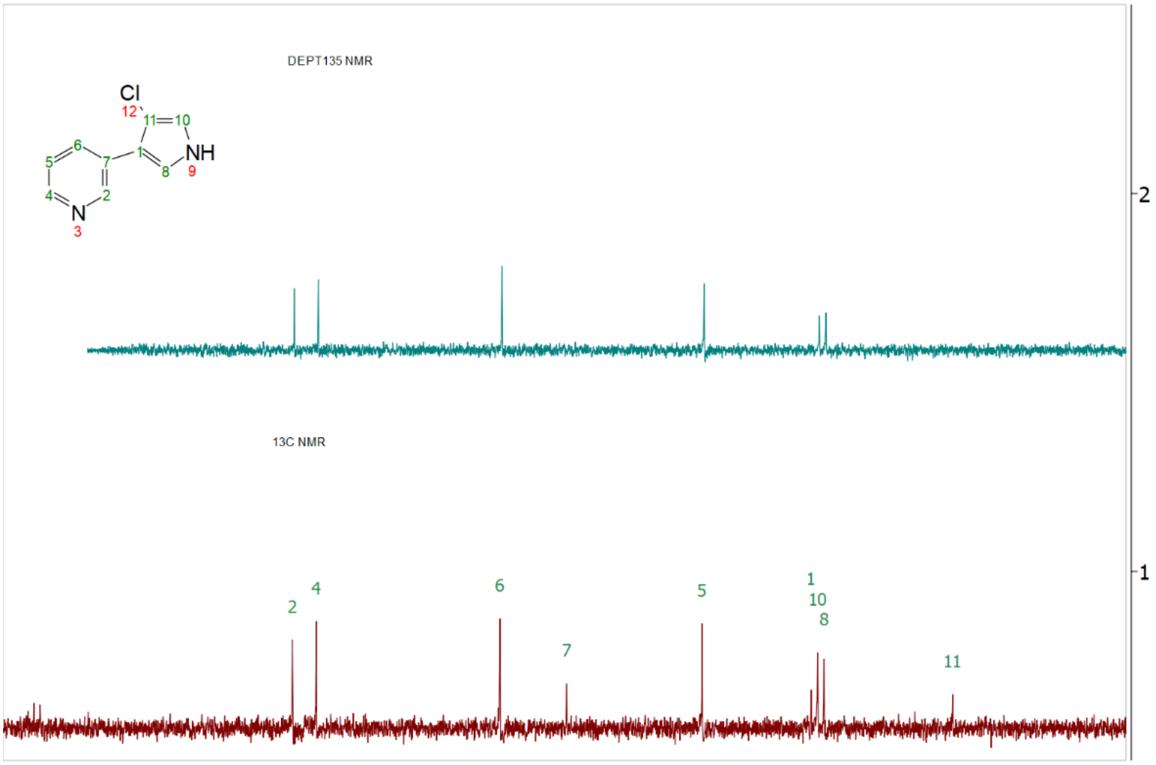

Figure S57. DEPT 135 NMR spectrum for product **8**.

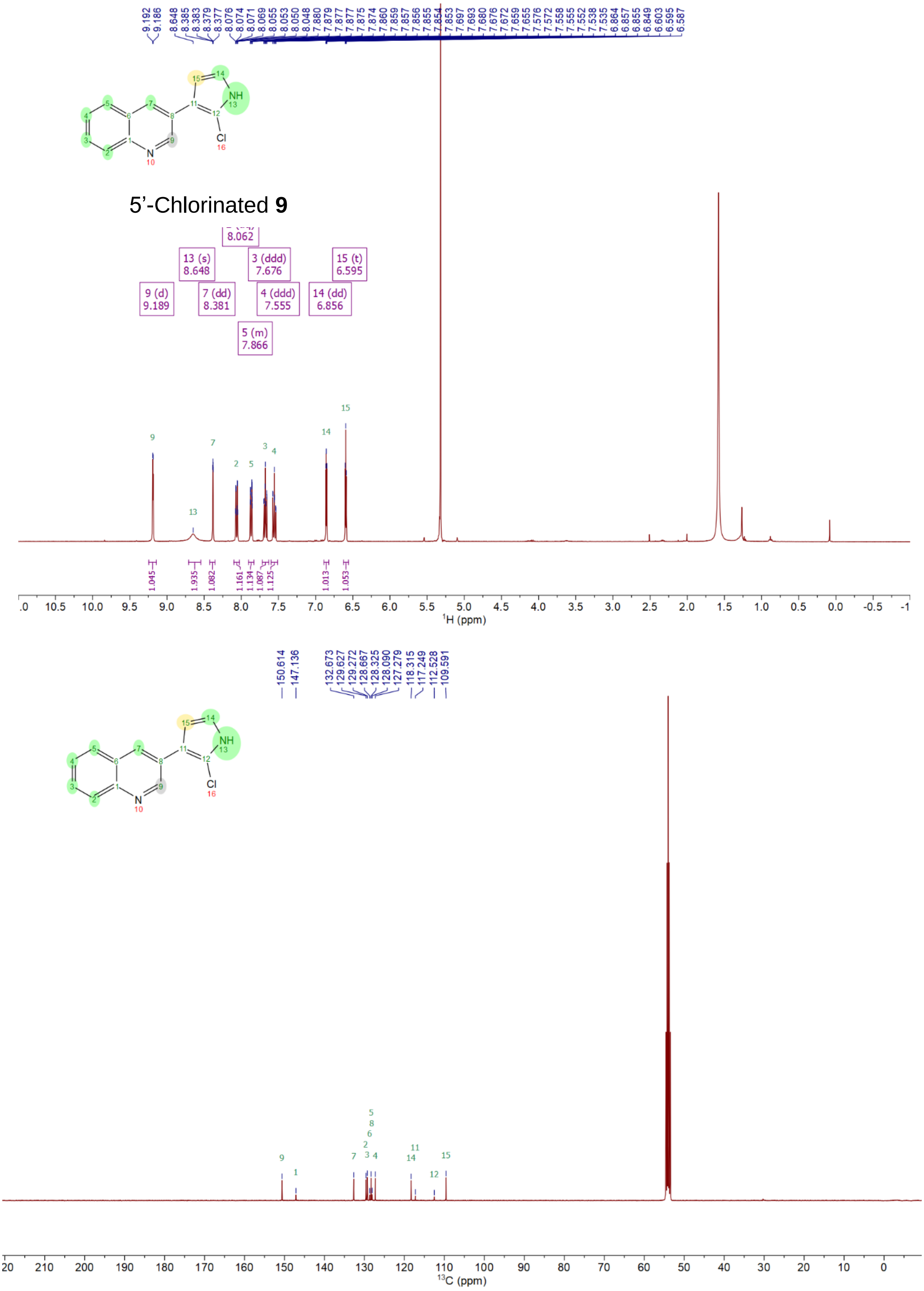

Figure S58. ^{1}H and ^{13}C NMR spectrum for product 5'-Chlorinated-**9**.

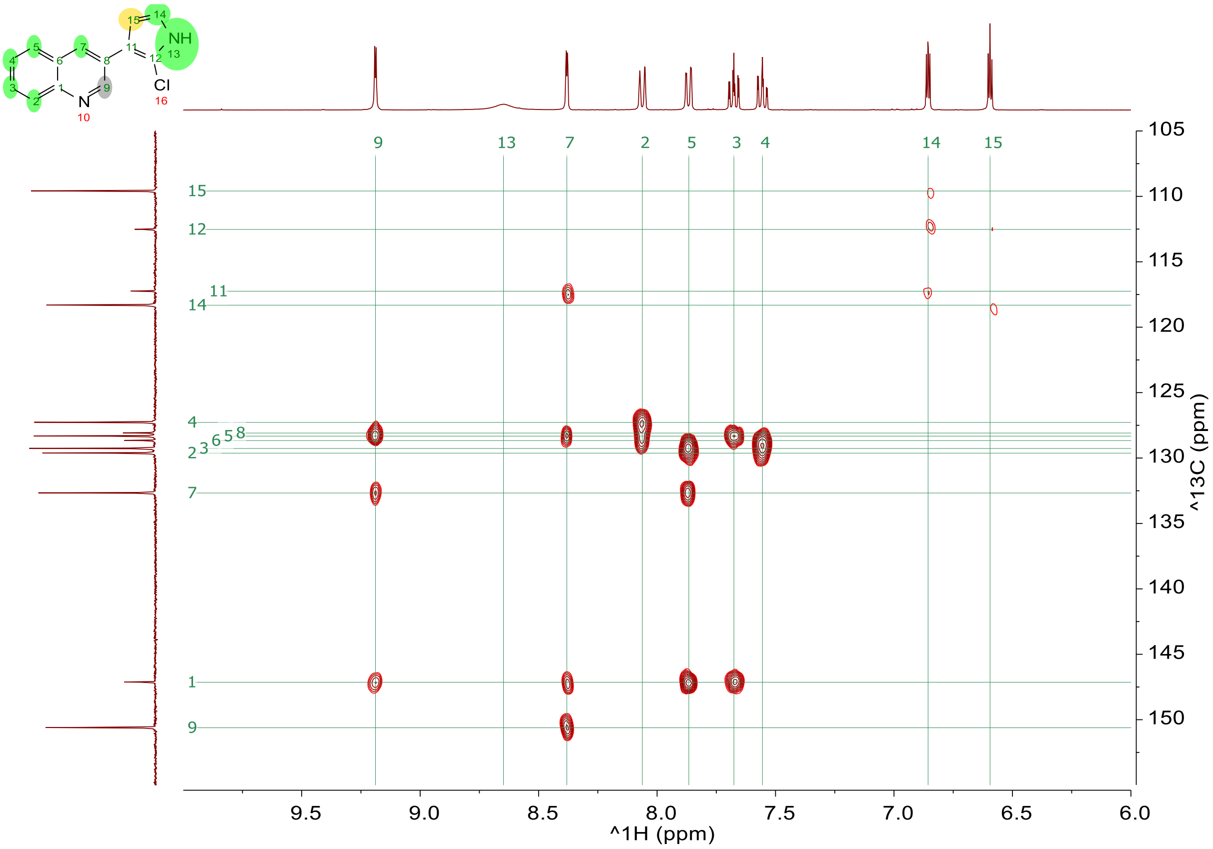

Figure S59. ^{1}H-^{13}C HMBC spectrum for product 5'-Chlorinated-**9**.

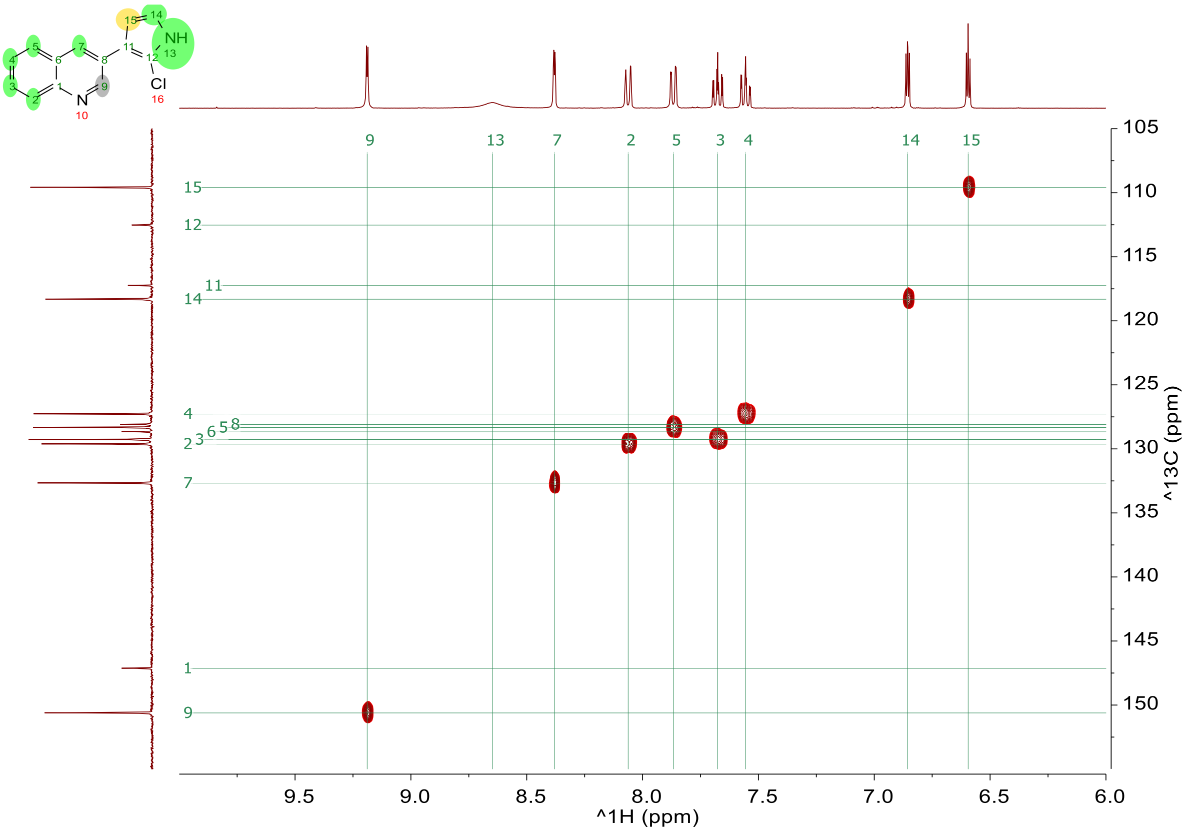

Figure S60. ^{1}H-^{13}C HSQC spectrum for product 5'-Chlorinated-**9**.

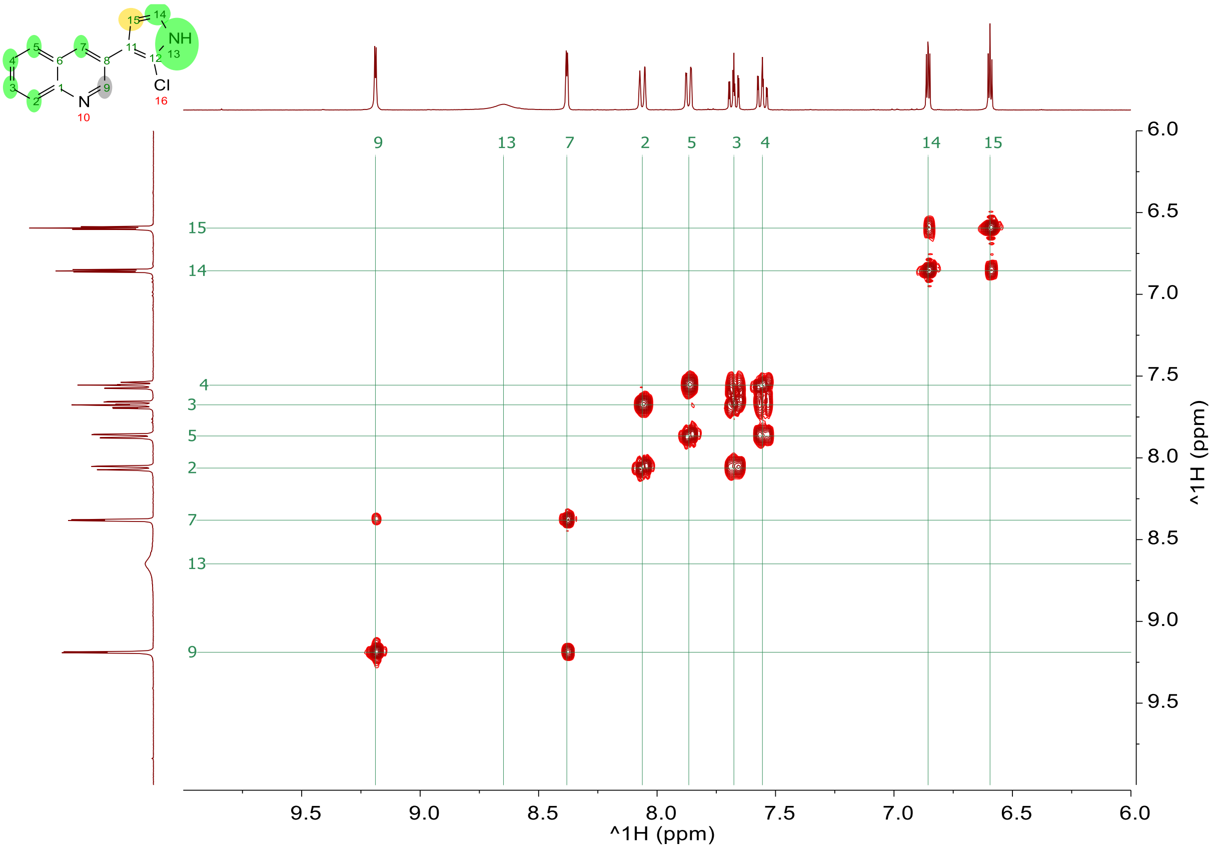

Figure S61. ^{1}H-^{1}H COSY spectrum for product 5'-Chlorinated-**9**.

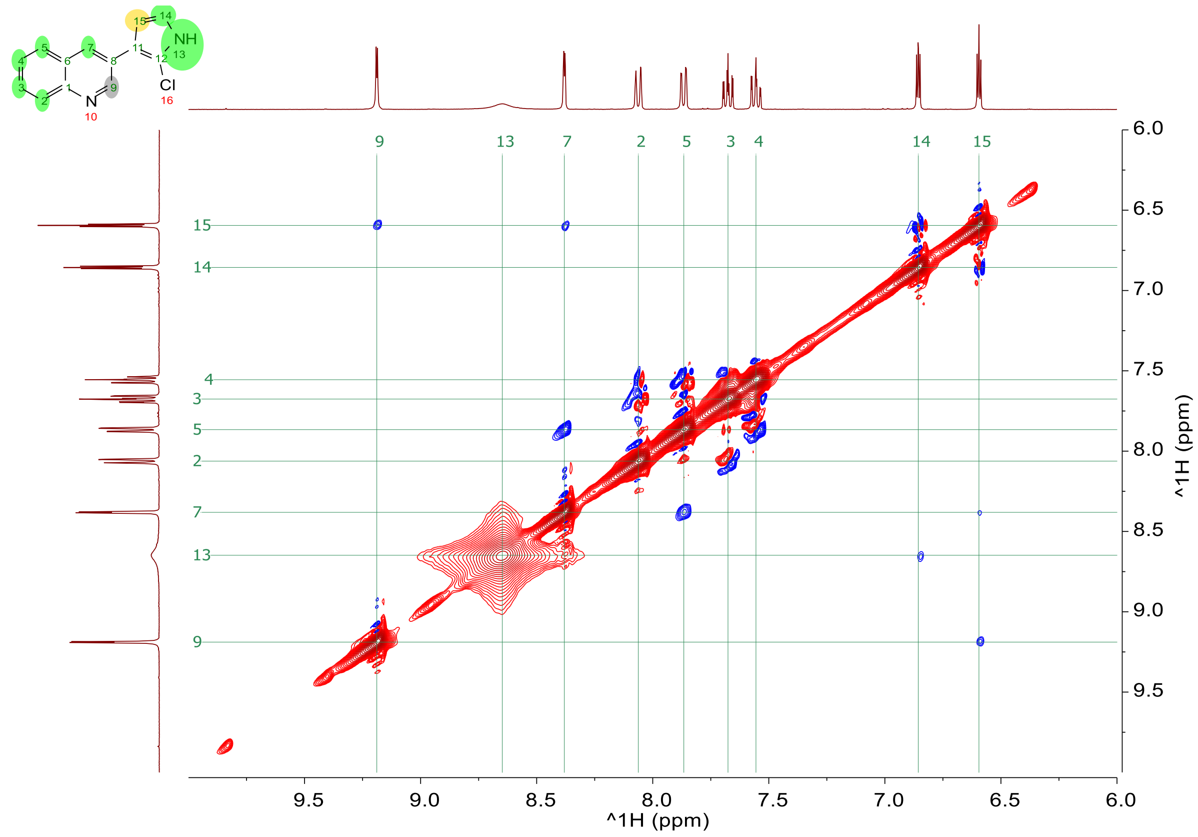

Figure S62. ^{1}H-^{1}H NOESY spectrum for product 5'-Chlorinated-**9**.

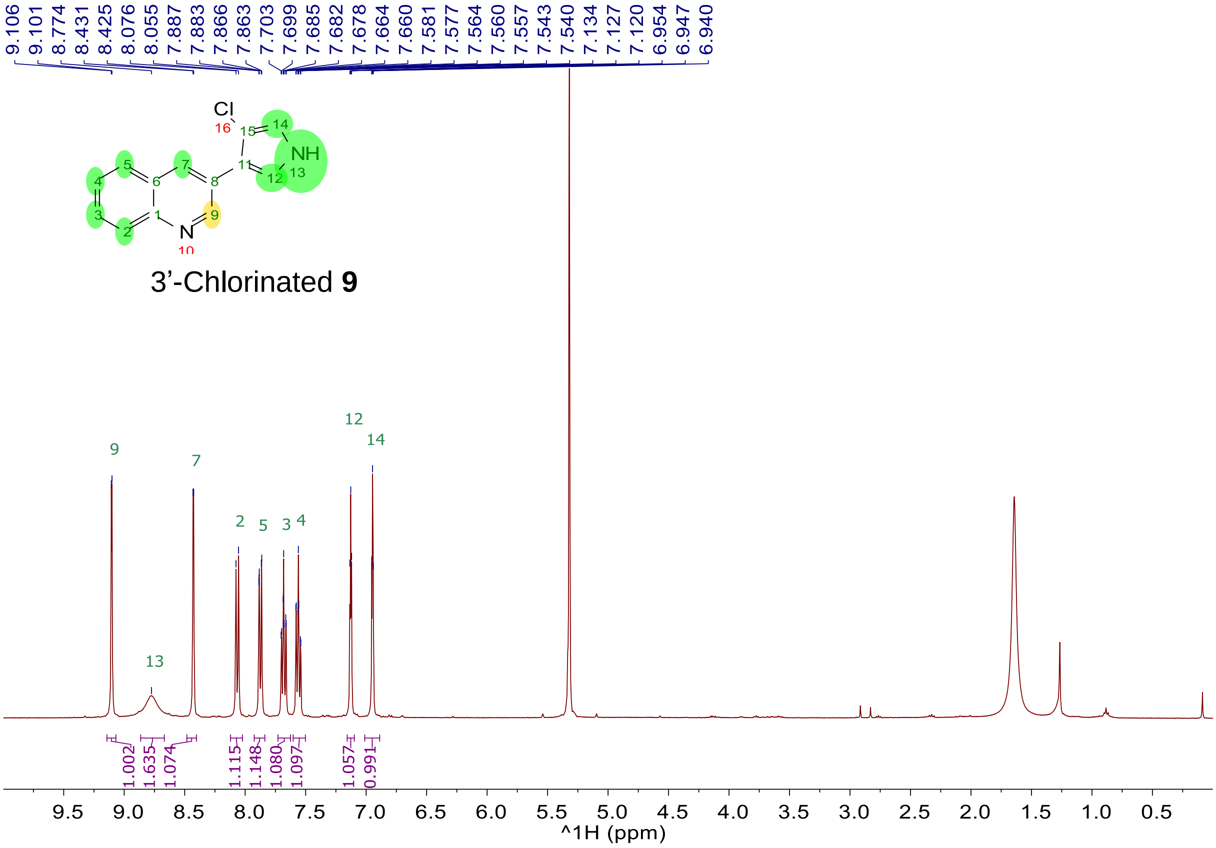

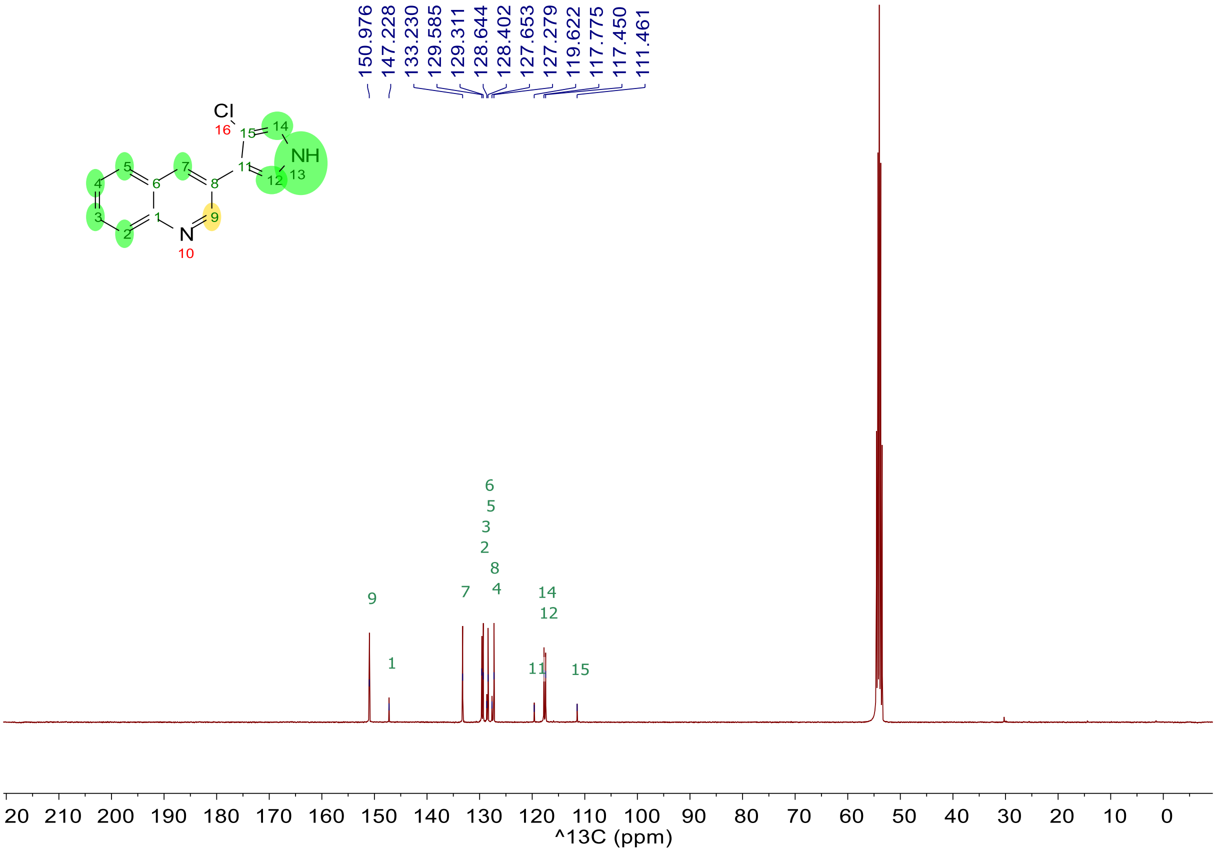

Figure S63. ^{1}H and ^{13}C NMR spectrum for product 3'-Chlorinated-**9**.

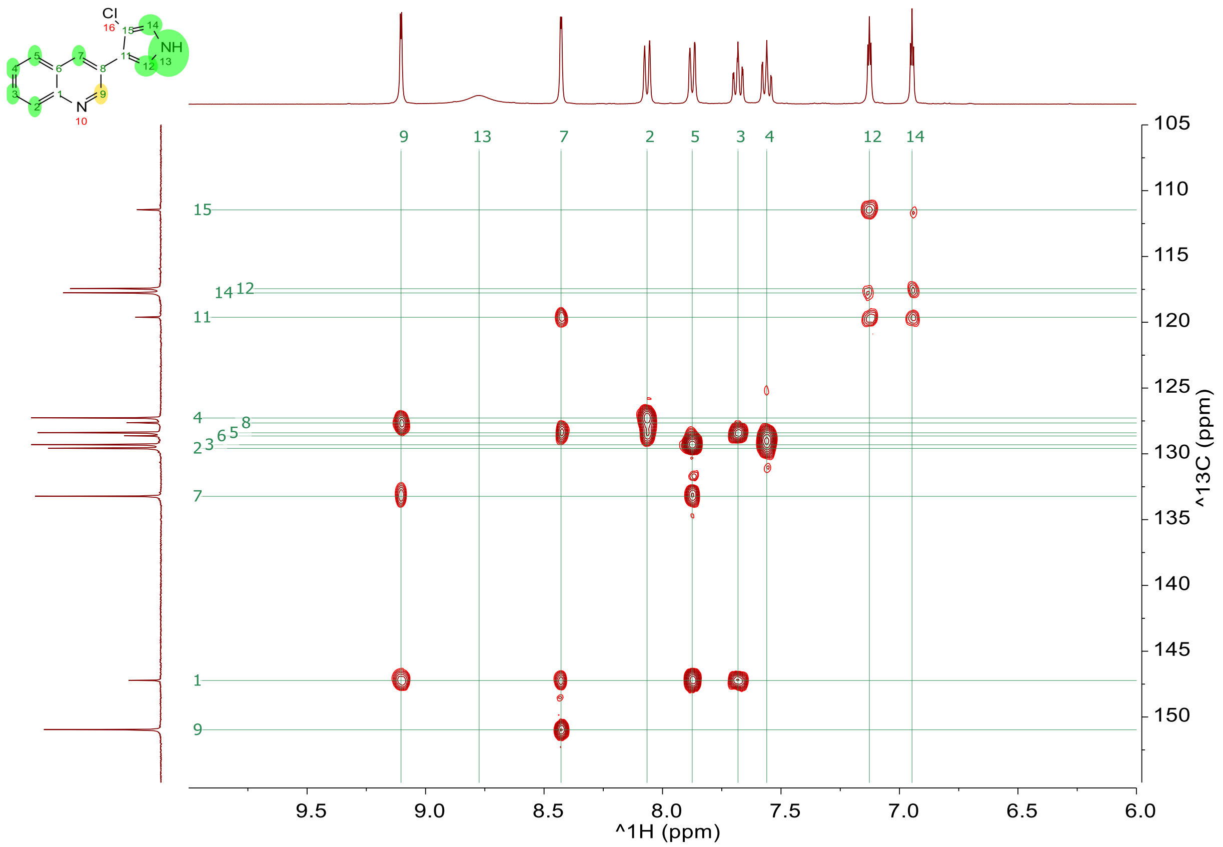

Figure S64. ^{1}H-^{13}C HMBC spectrum for product 3'-Chlorinated-**9**.

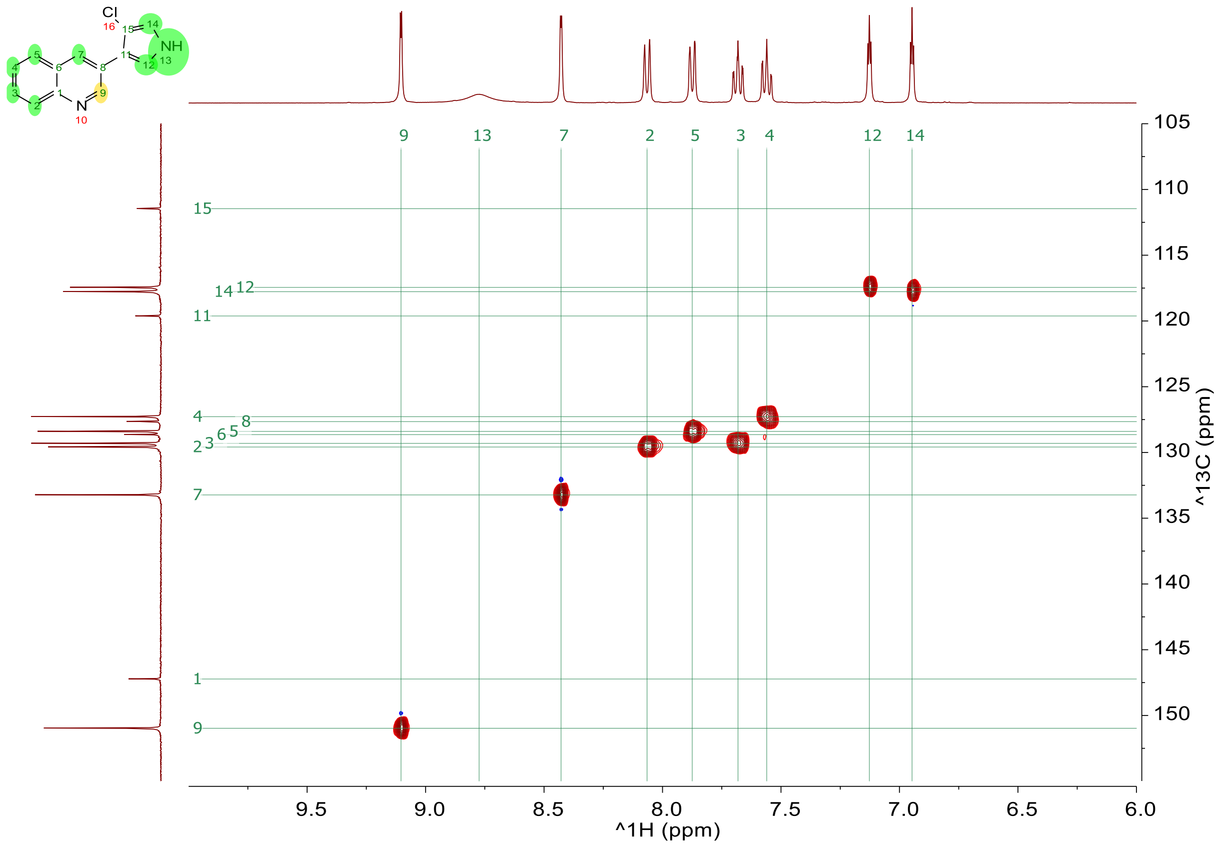

Figure S65. ^{1}H-^{13}C HSQC spectrum for product 3'-Chlorinated-**9**.

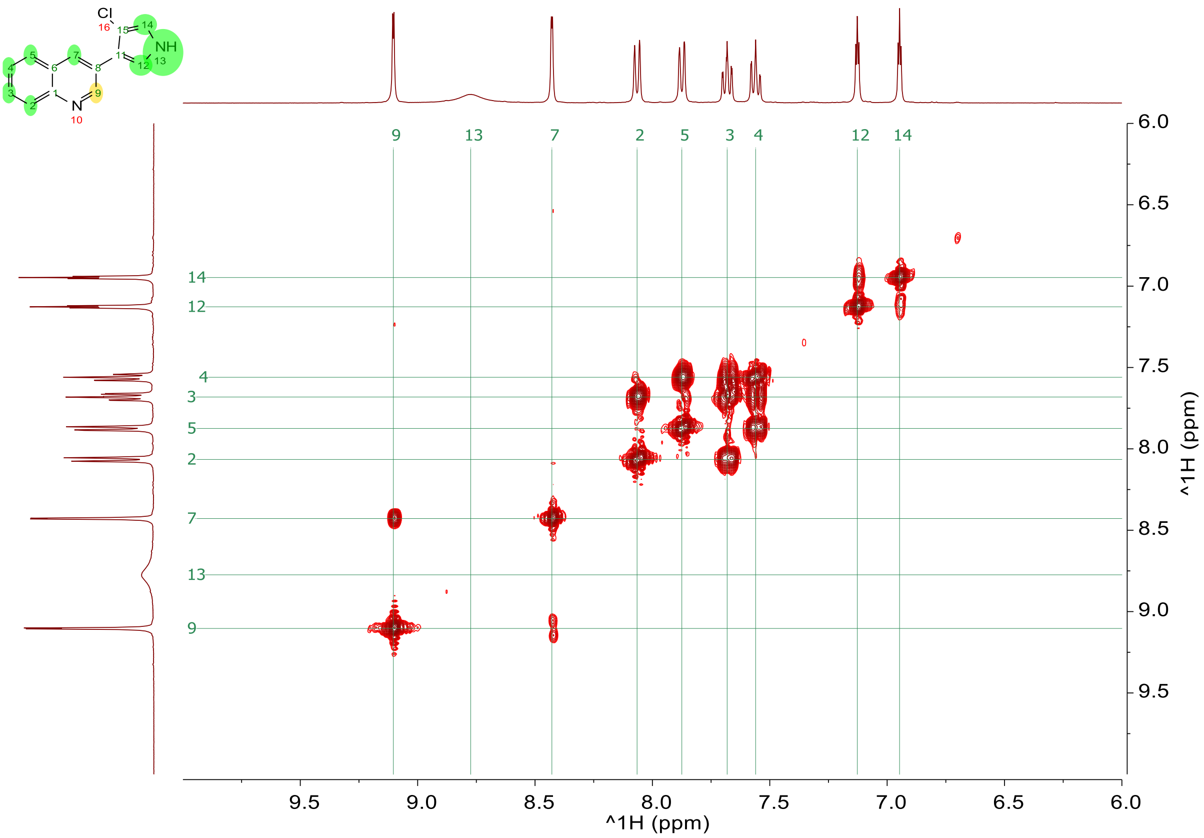

Figure S66. ^{1}H-^{1}H COSY spectrum for product 3’-Chlorinated-**9**.

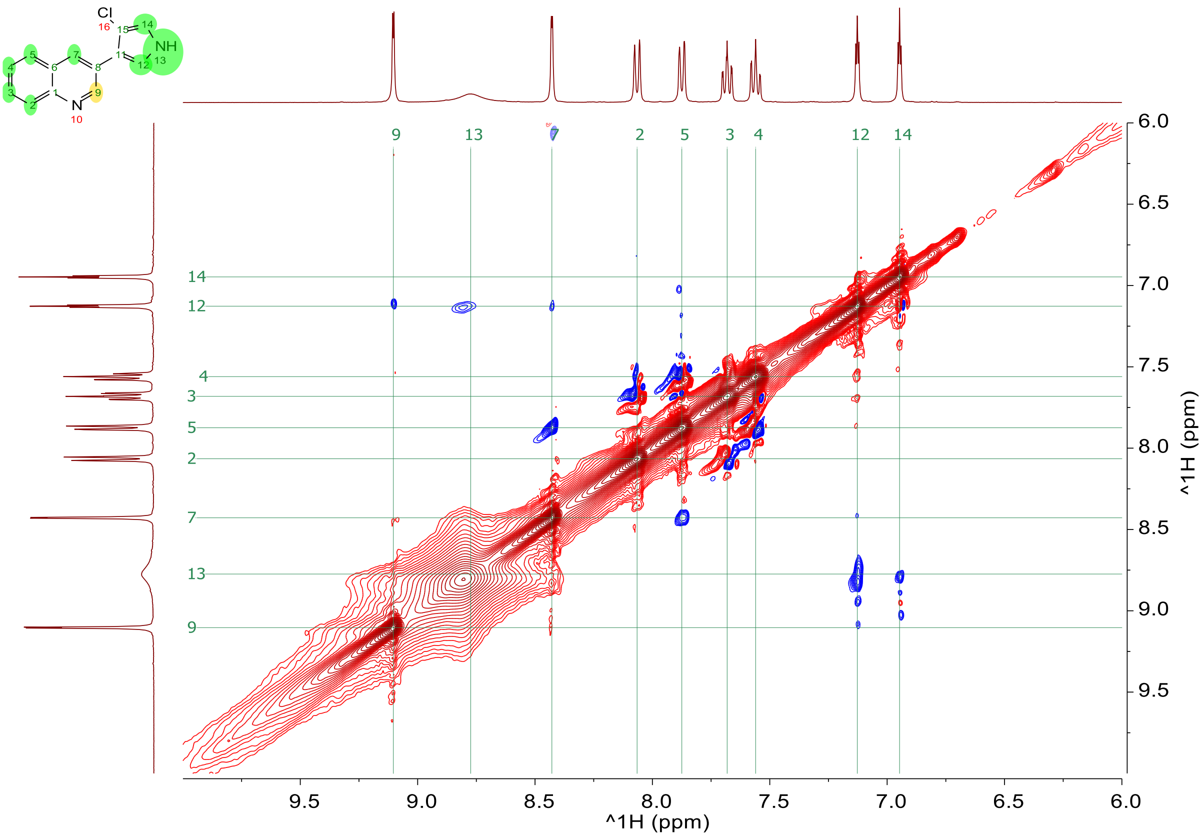

Figure S67. ^{1}H-^{1}H NOESY spectrum for product 3’-Chlorinated-**9**.

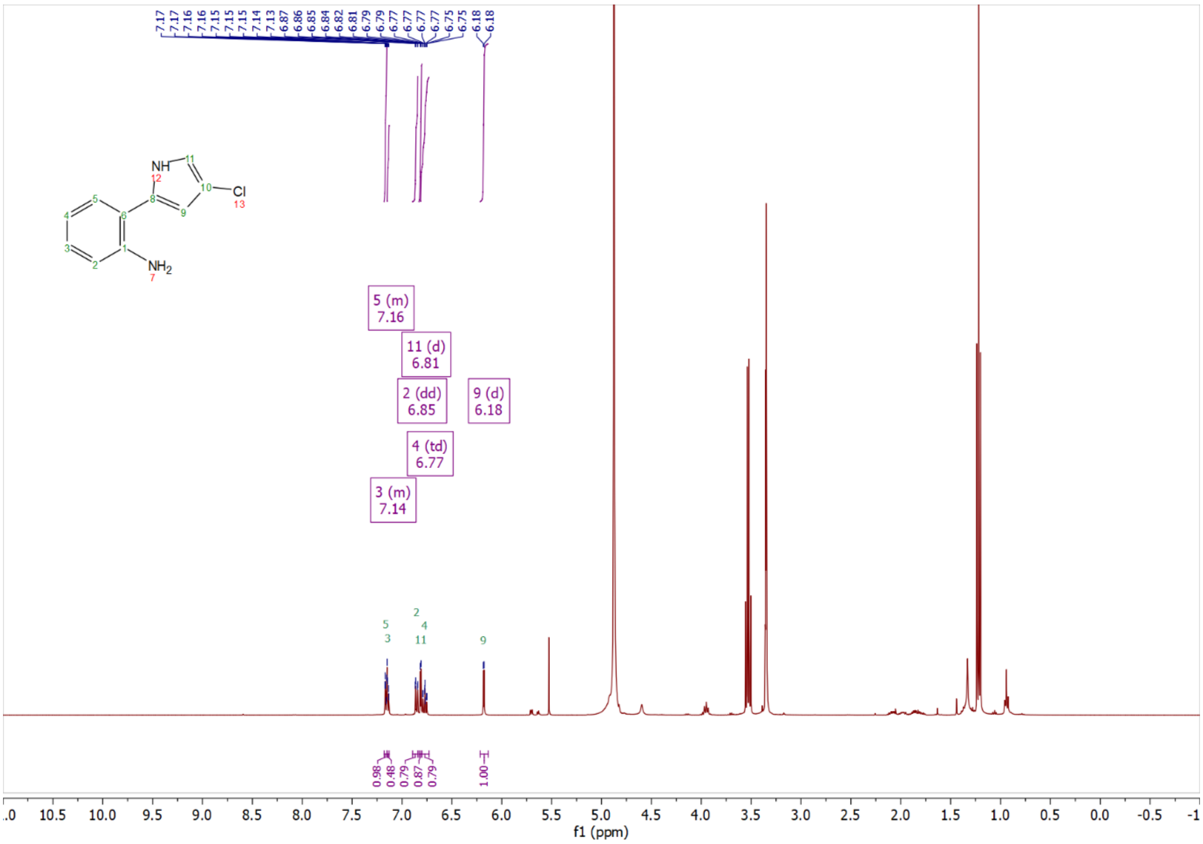

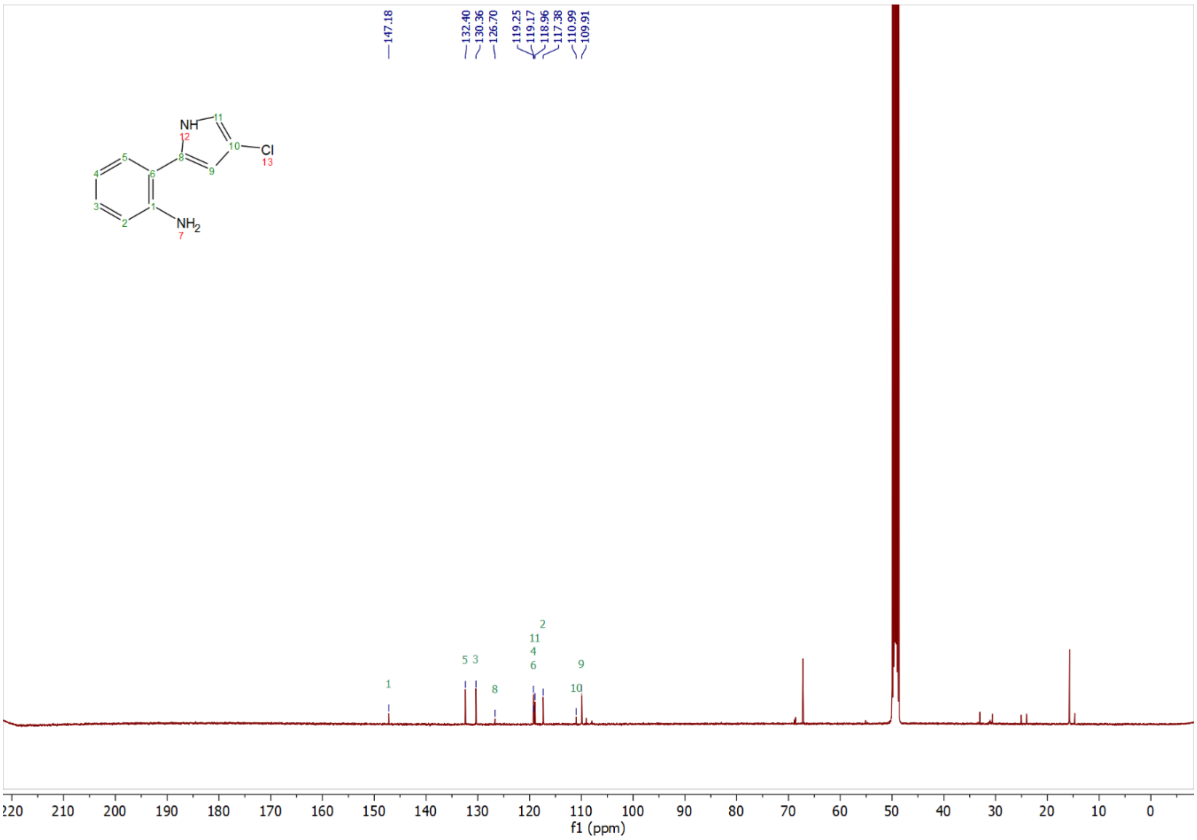

Figure S68. ^{1}H and ^{13}C NMR spectrum for product **10**.

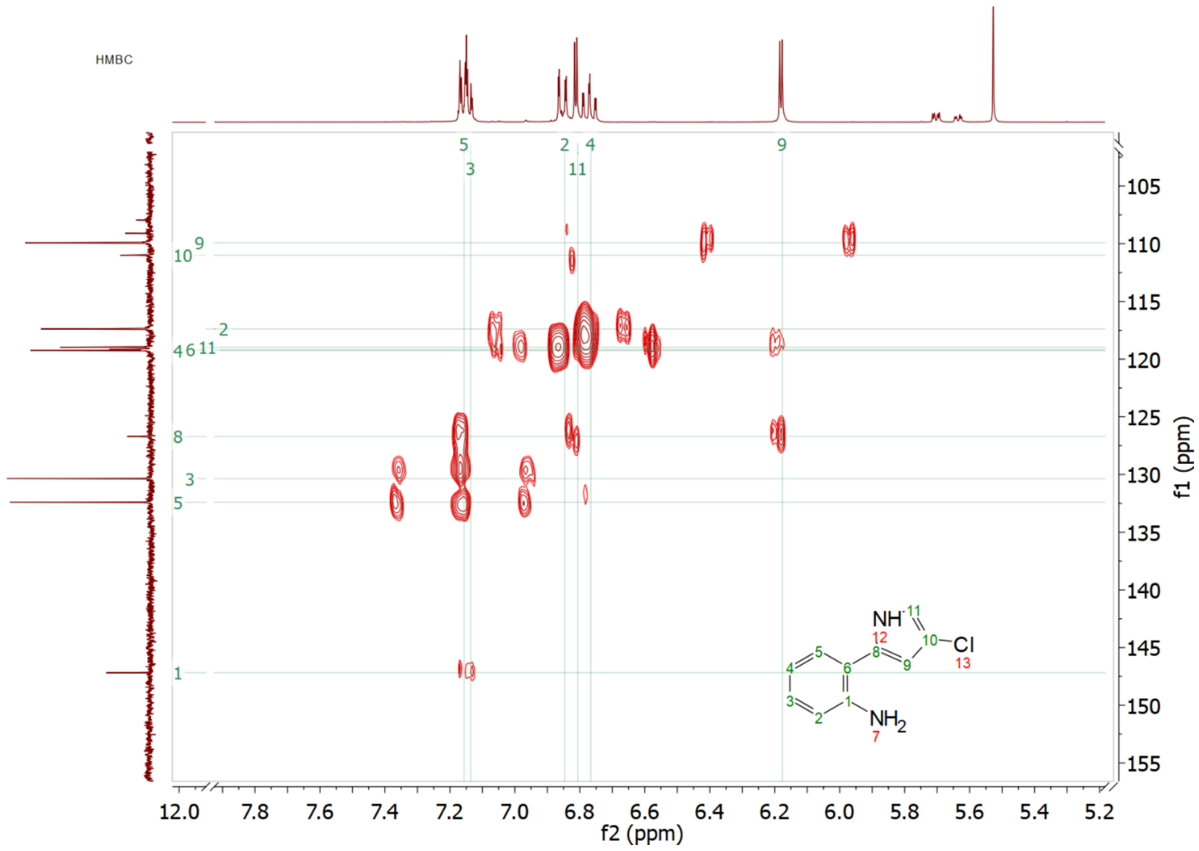

Figure S69. ^{1}H-^{13}C HMBC NMR spectrum for product **10**.

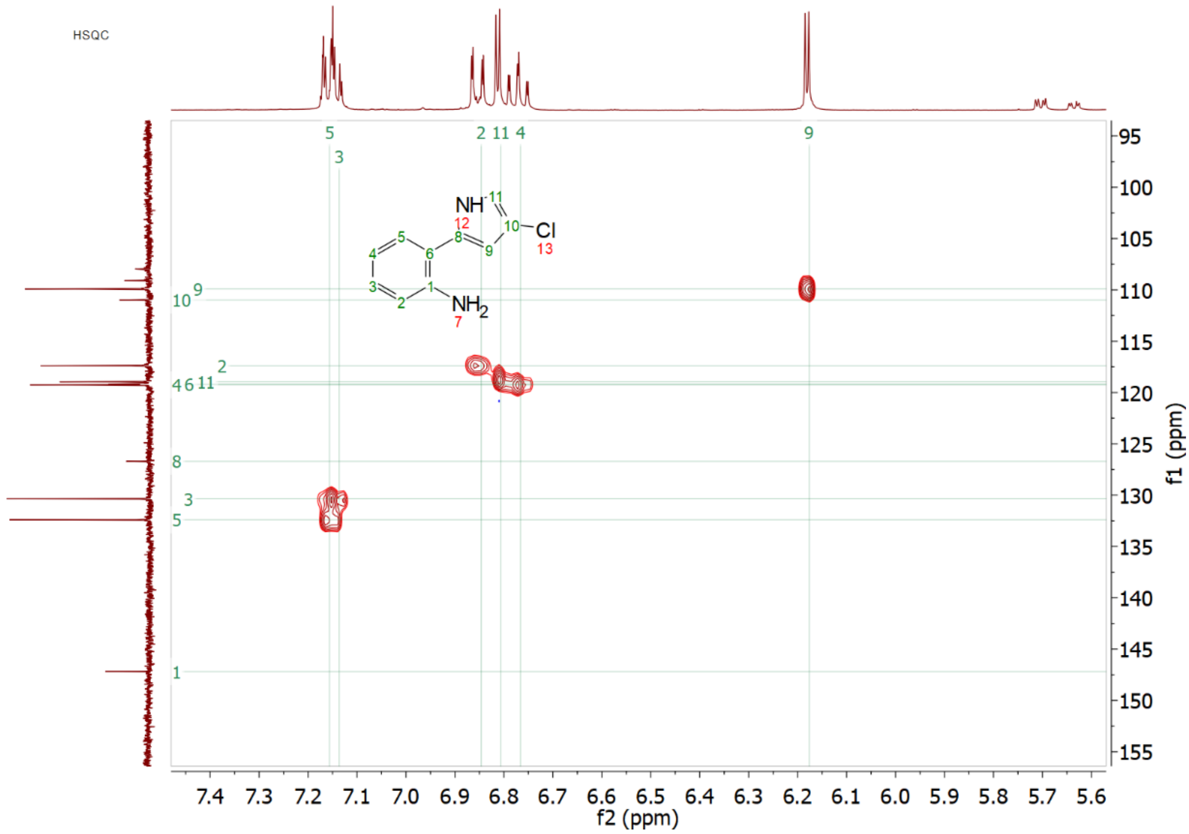

Figure S70. ^{1}H-^{13}C HSQC NMR spectrum for product **10**.

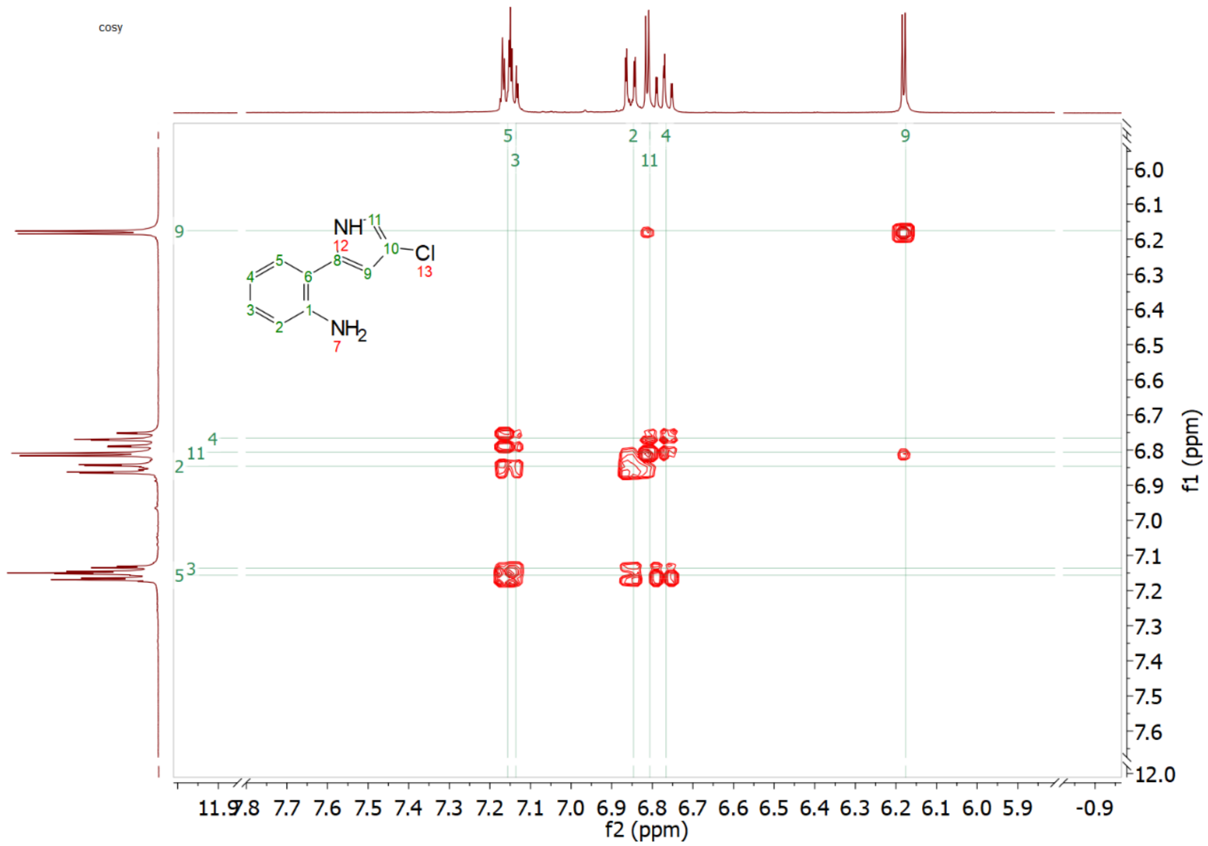

Figure S71. ^{1}H-^{1}H COSY NMR spectrum for product **10**.

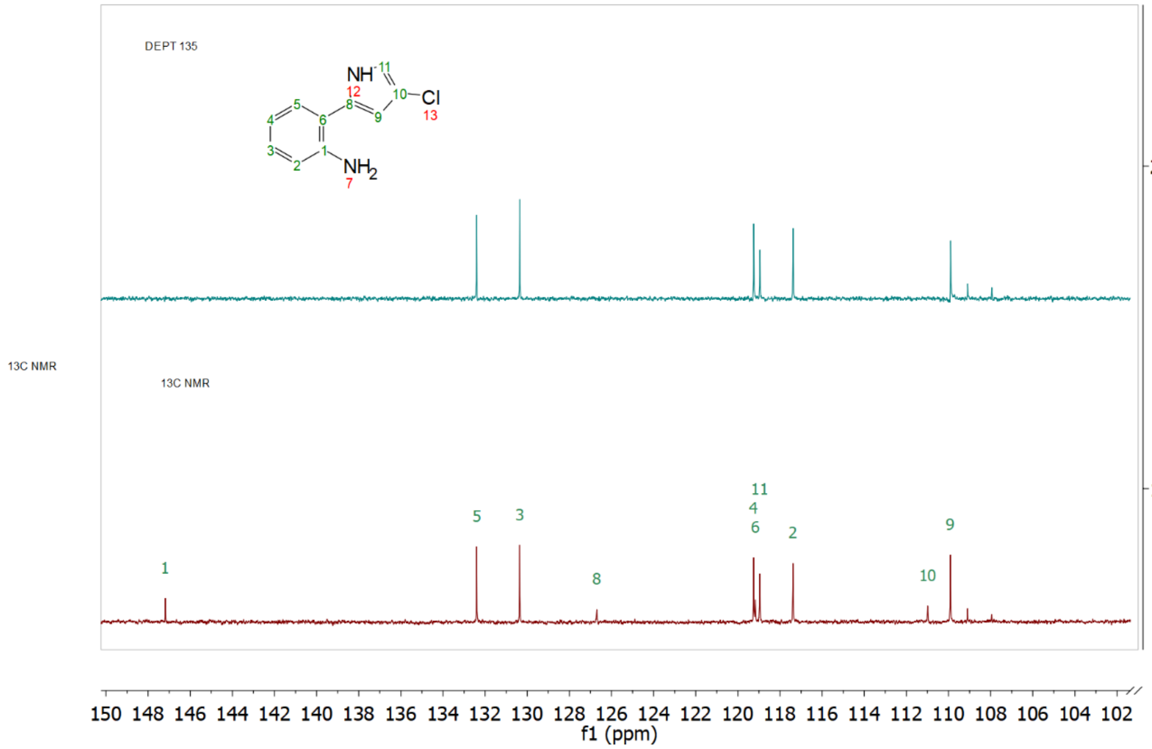

Figure S72. DEPT 135 NMR spectrum for product **10**.

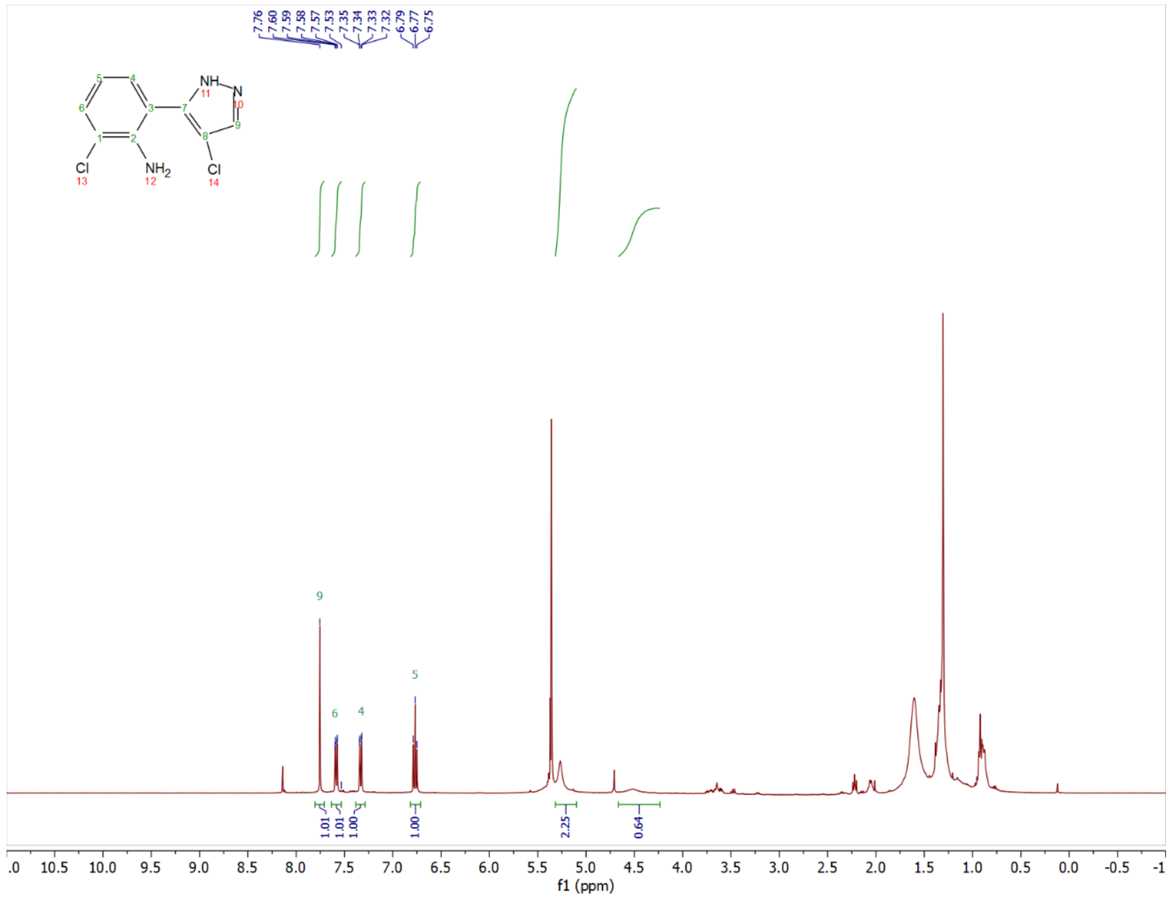

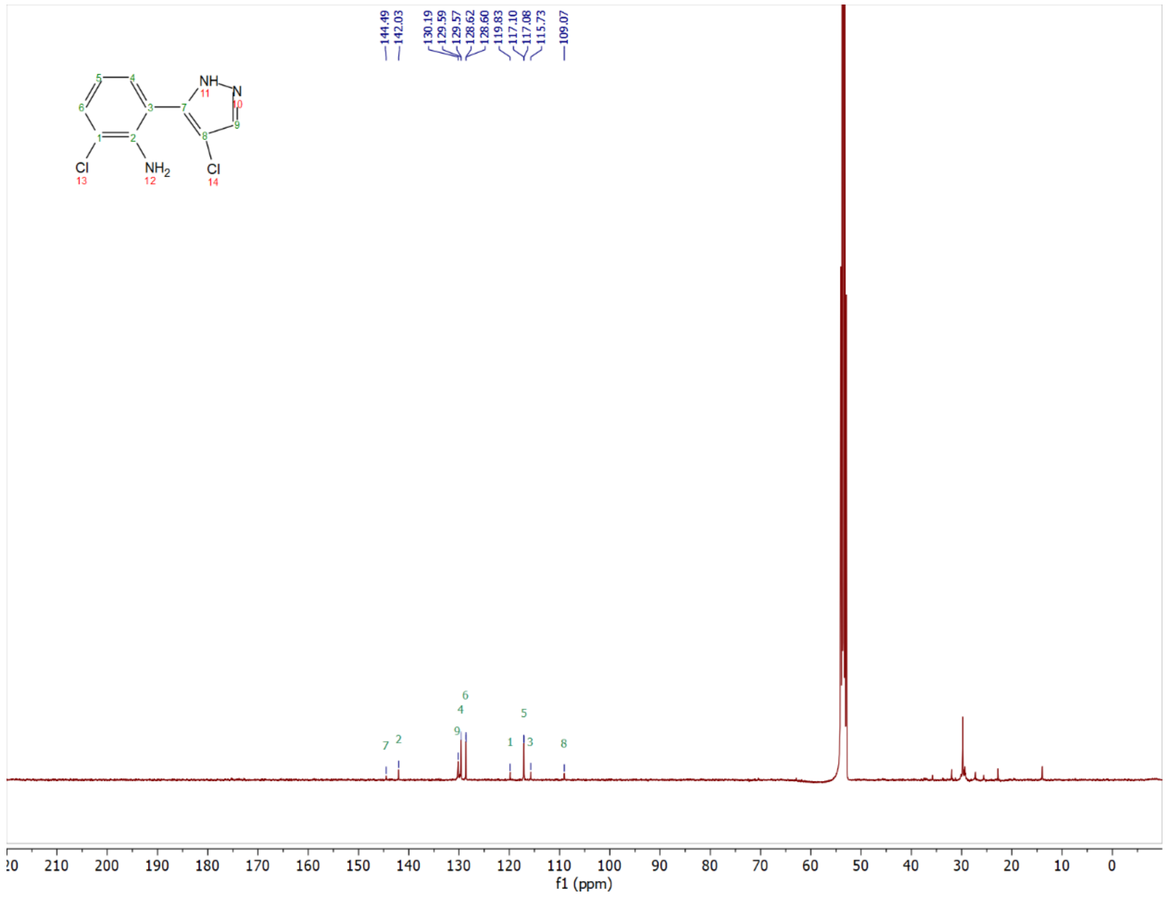

Figure S73. ^{1}H and ^{13}C NMR spectrum for product **11**.

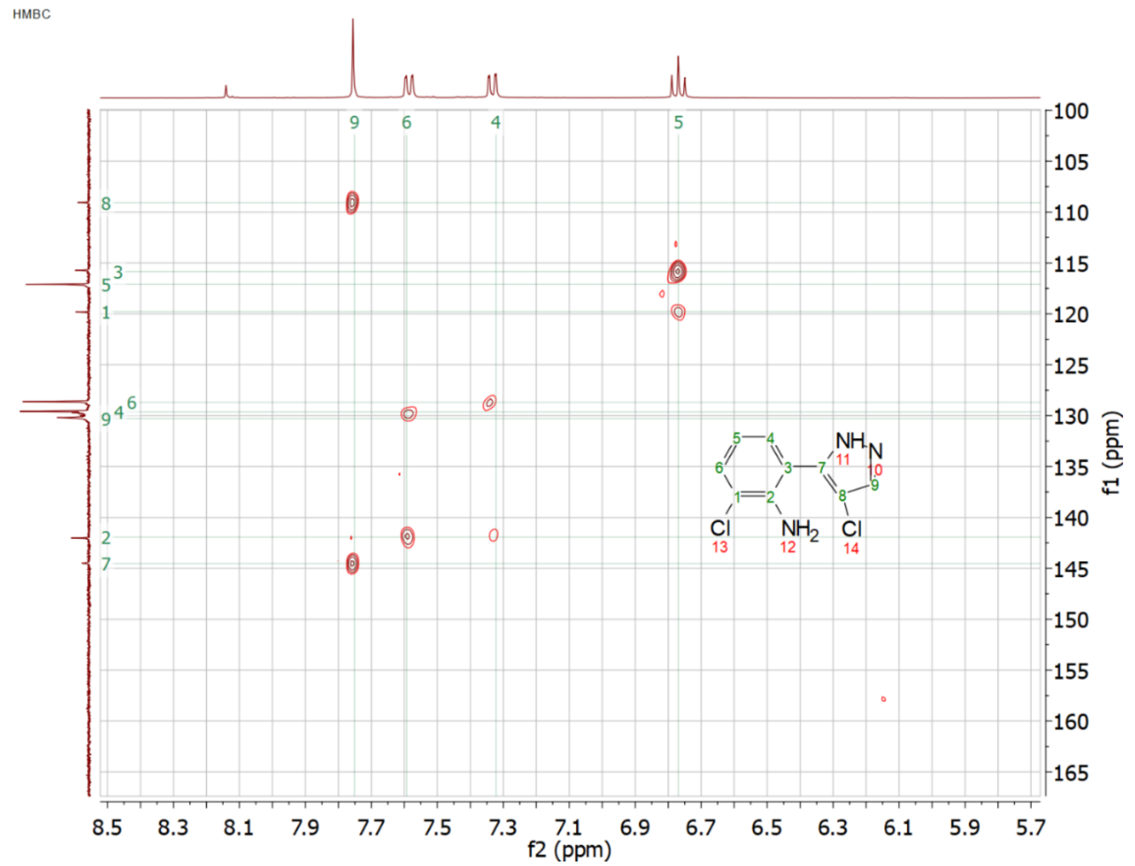

Figure S74. ^{1}H-^{13}C HMBC NMR spectrum for product **11**.

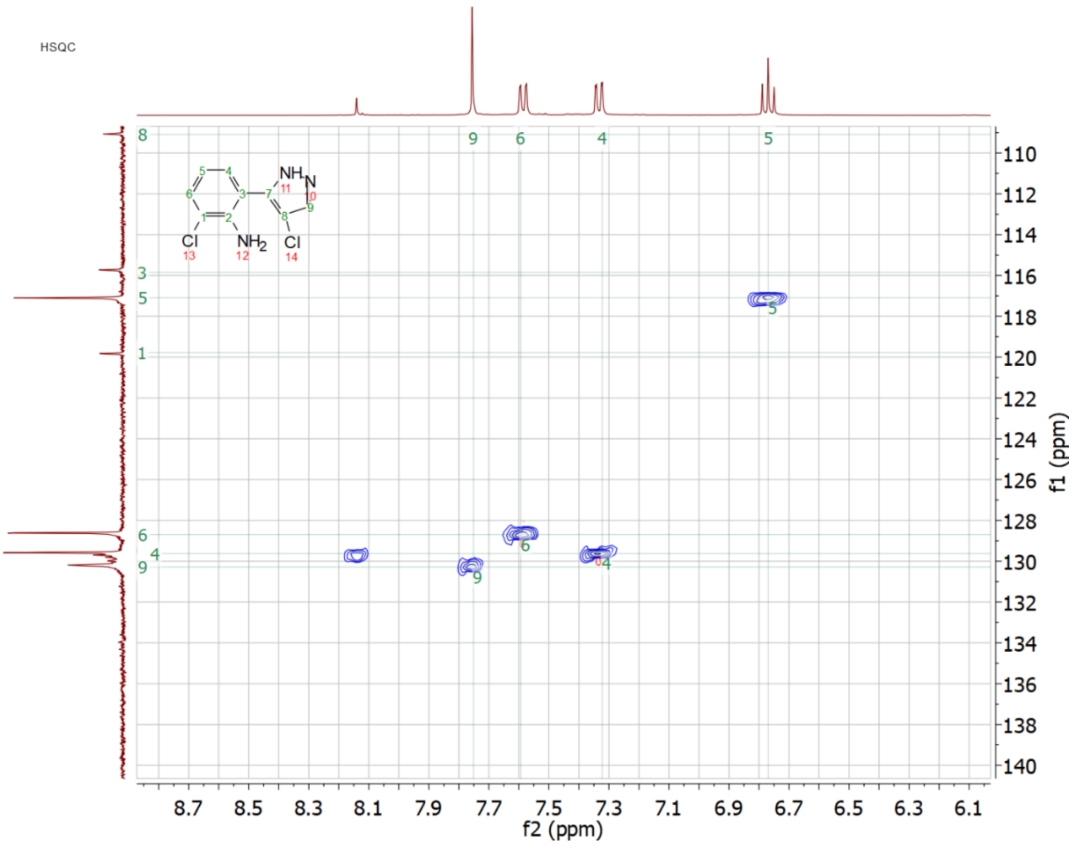

Figure S75. ^{1}H-^{13}C HSQC NMR spectrum for product **11**.

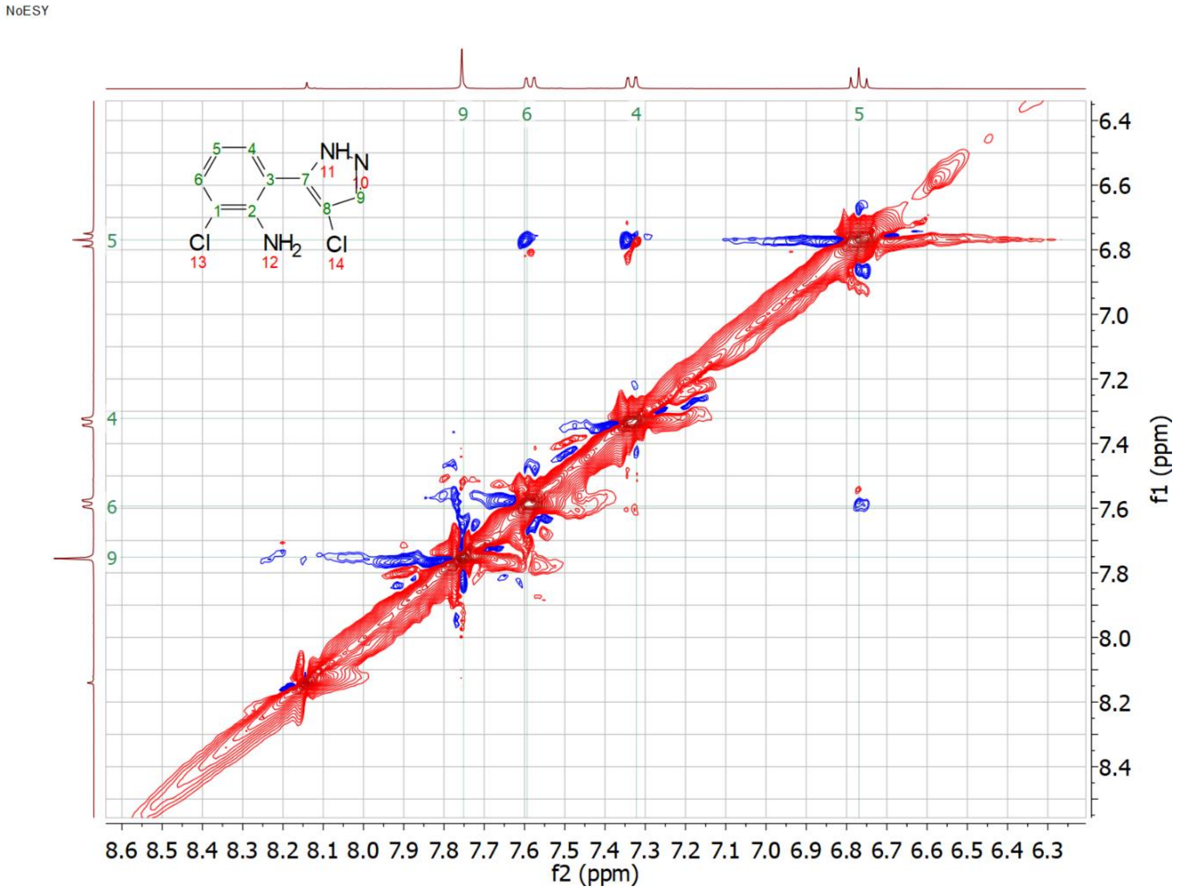

Figure S76. ^{1}H-^{1}H NOESY NMR spectrum for product **11**.

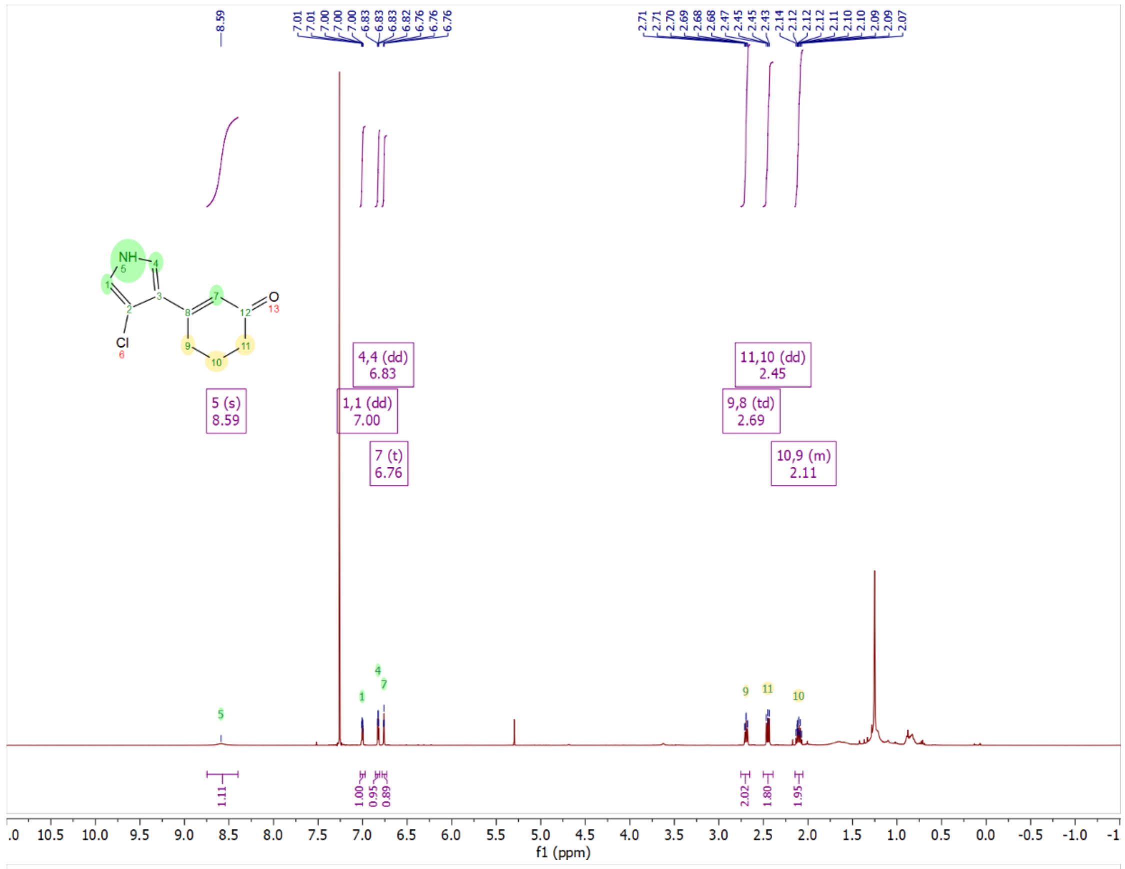

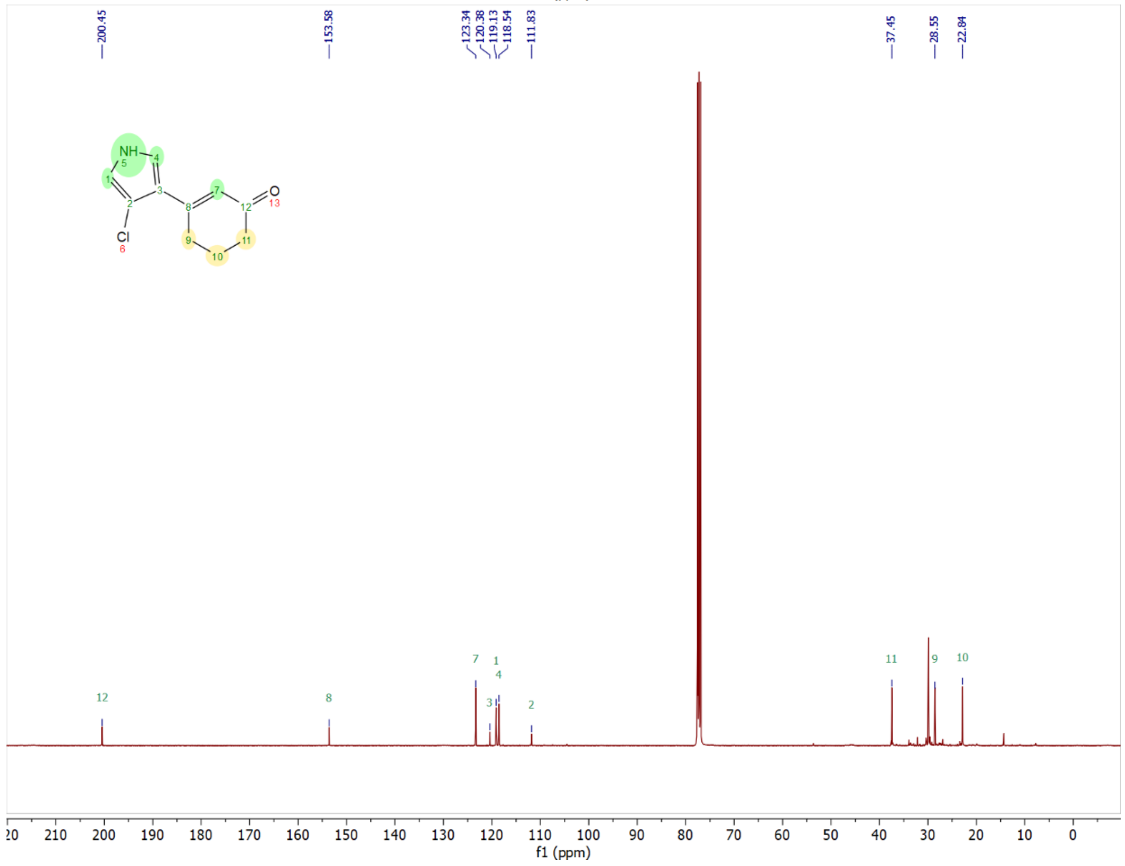

Figure S77. ^{1}H and ^{13}C NMR spectrum for **13**.

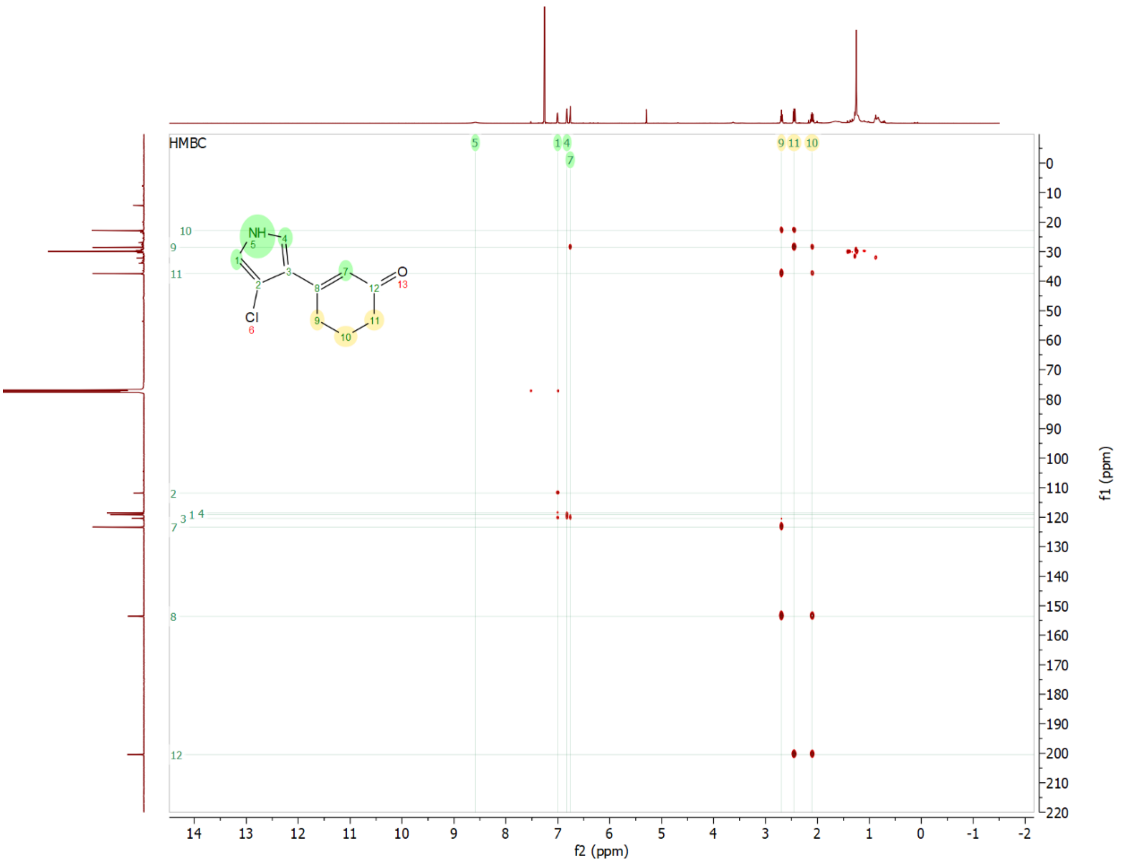

Figure S78. ^{1}H-^{13}C HMBC NMR spectrum for product **13.**

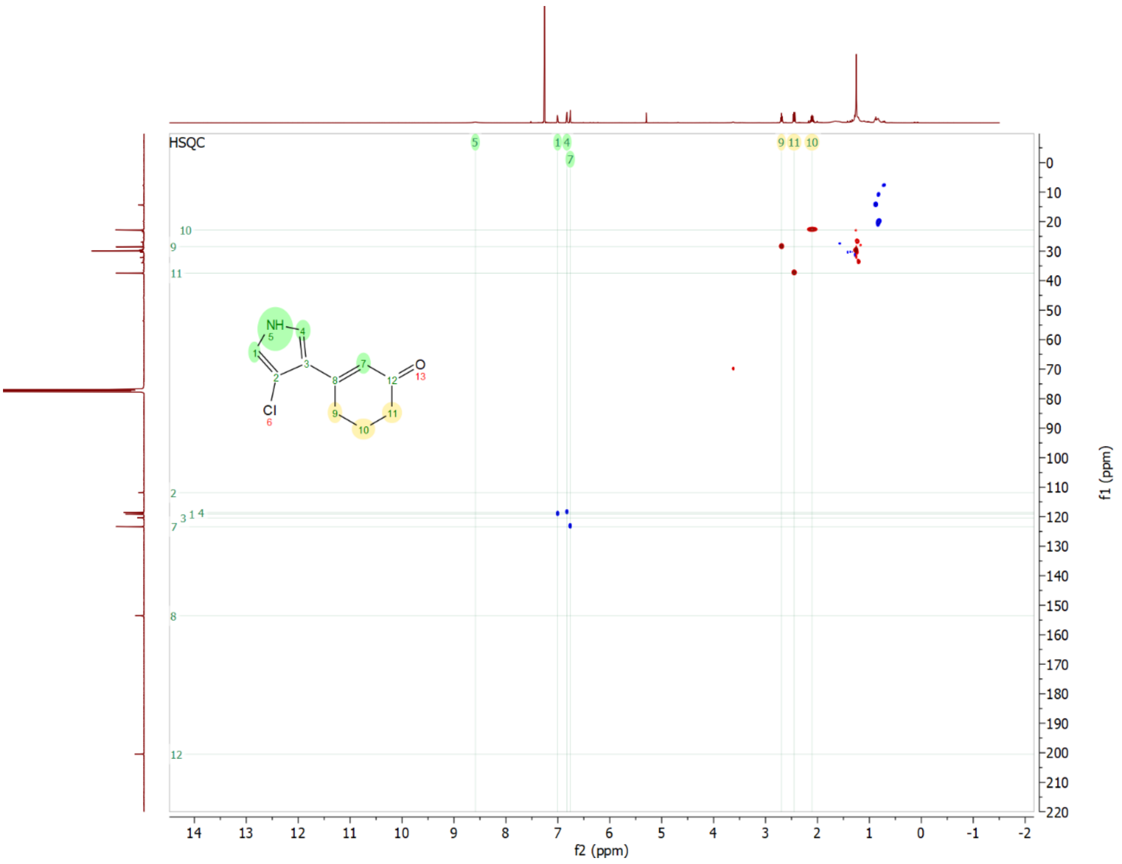

Figure S79. ^{1}H-^{13}C HSQC NMR spectrum for product **13.**

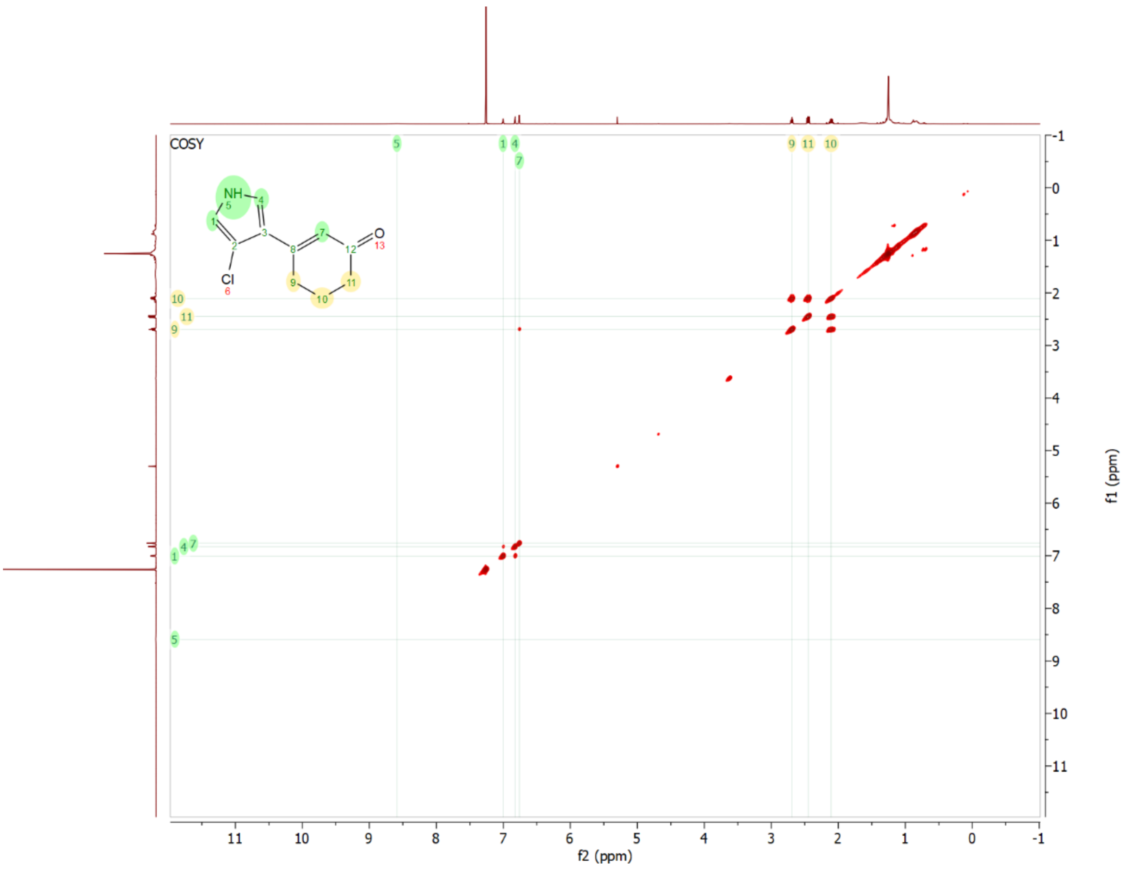

Figure S80. ^{1}H-^{1}H COSY NMR spectrum for product **13.**

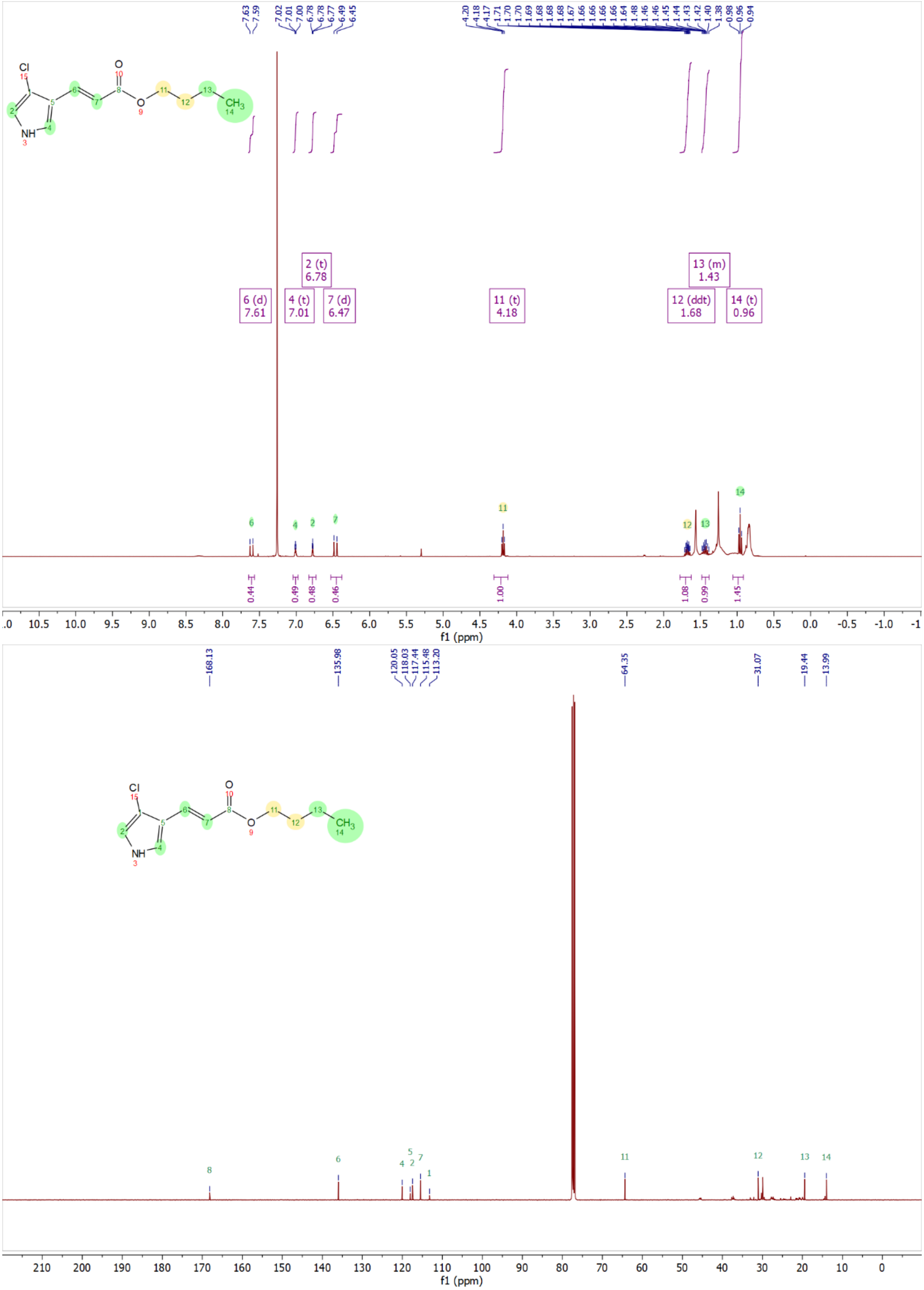

Figure S81. ^{1}H and ^{13}C NMR spectrum for **14**.

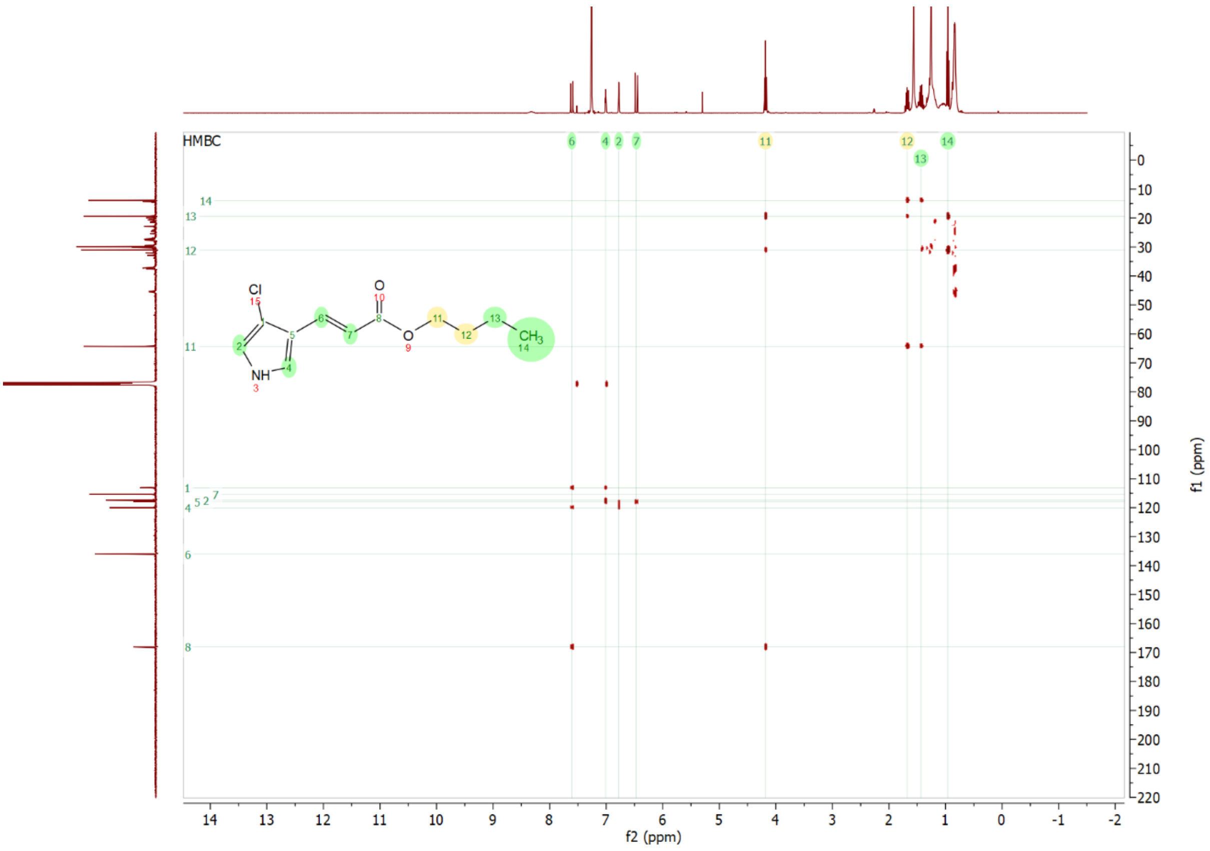

Figure S82. ^{1}H-^{13}C HMBC NMR spectrum for product **14.**

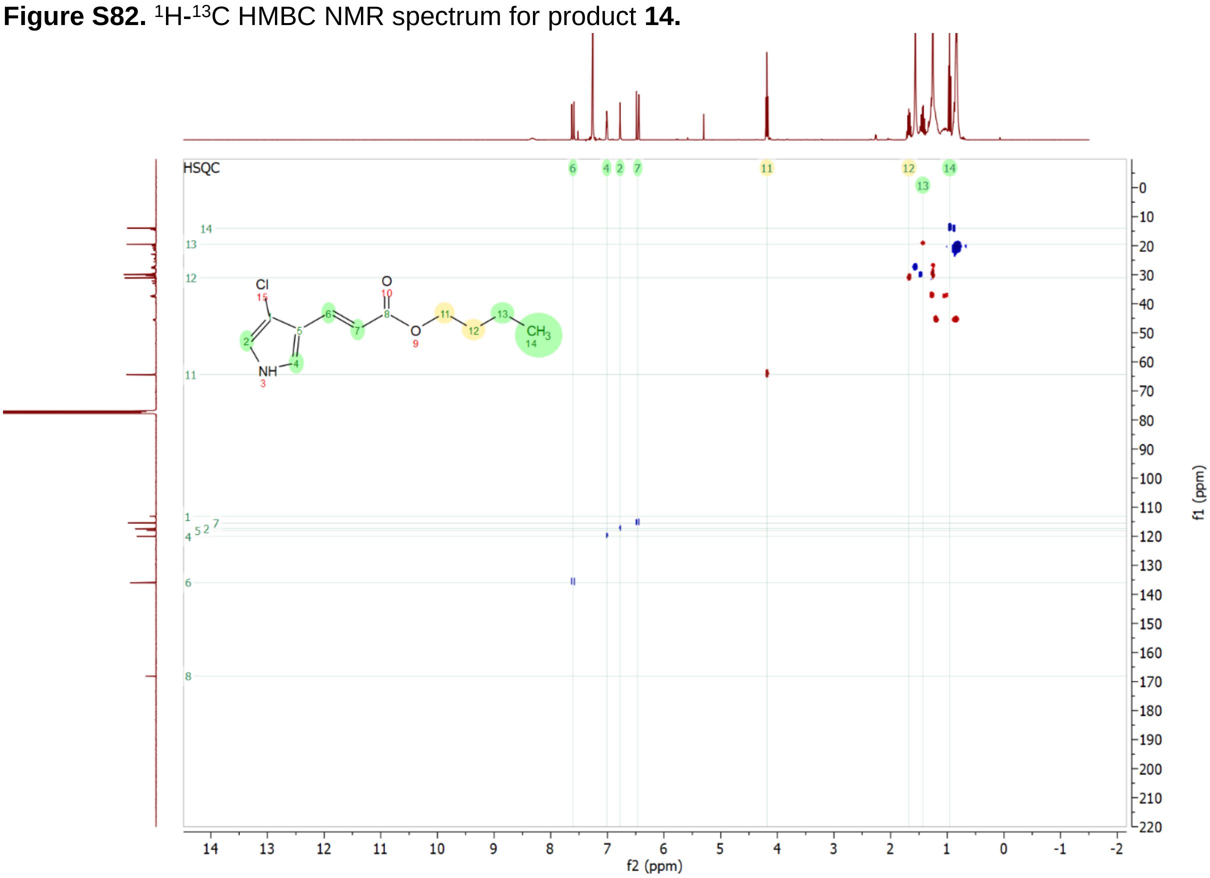

Figure S83. ^{1}H-^{13}C HSQC NMR spectrum for product **14.**

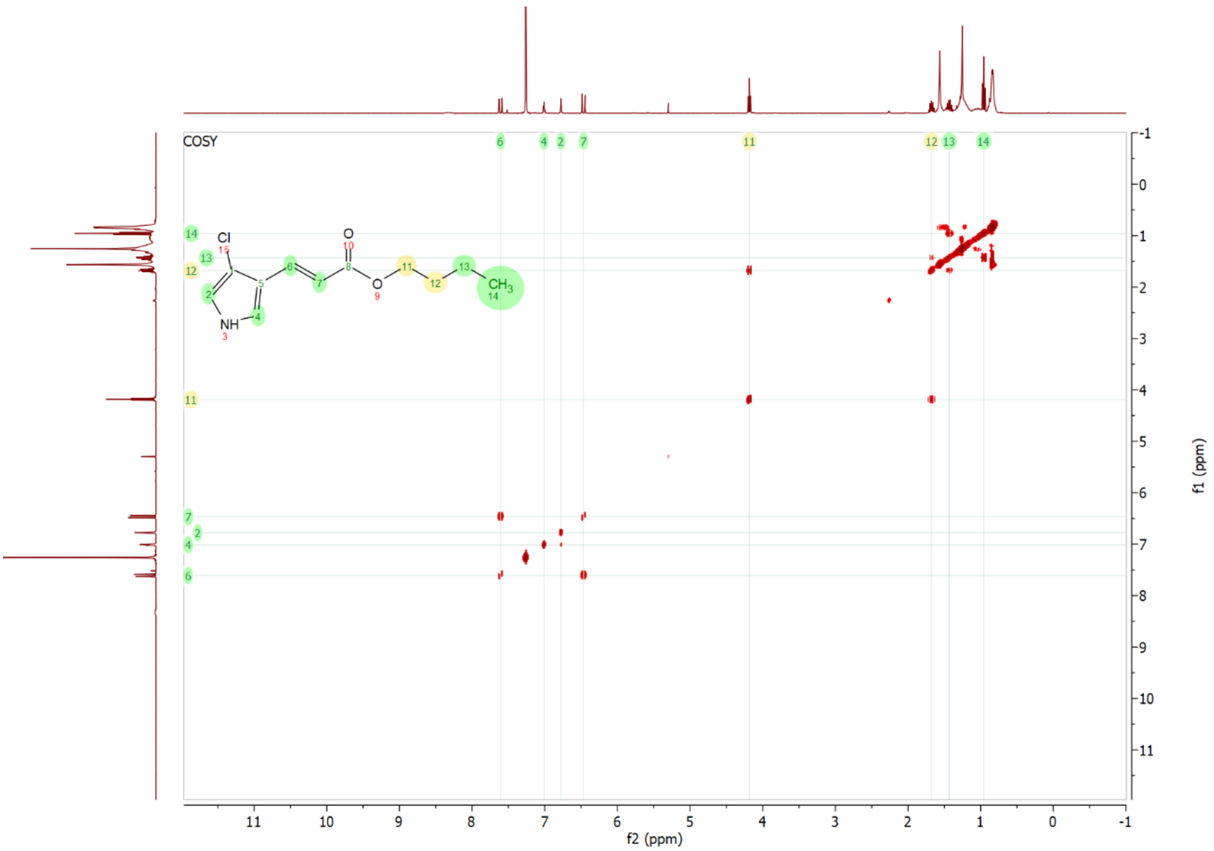

Figure S84. ^{1}H-^{1}H COSY NMR spectrum for product **14.**

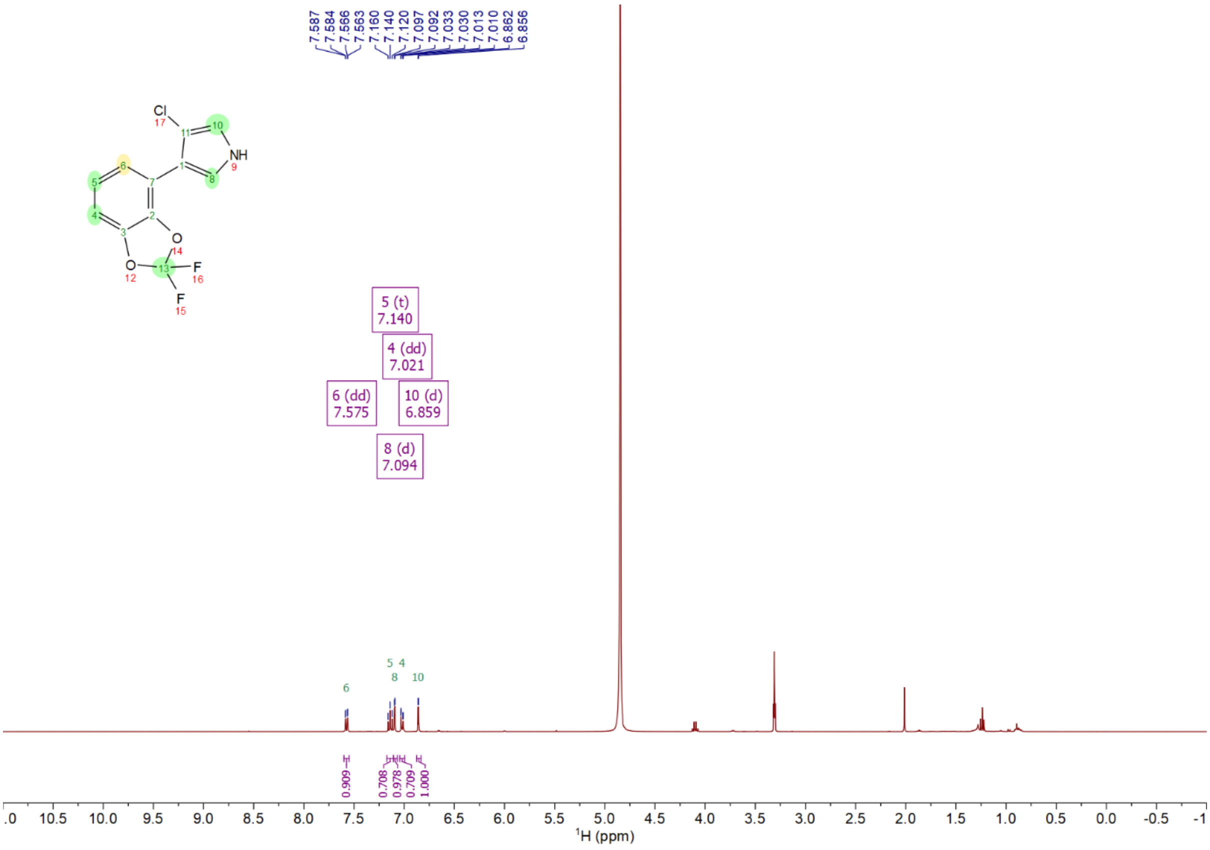

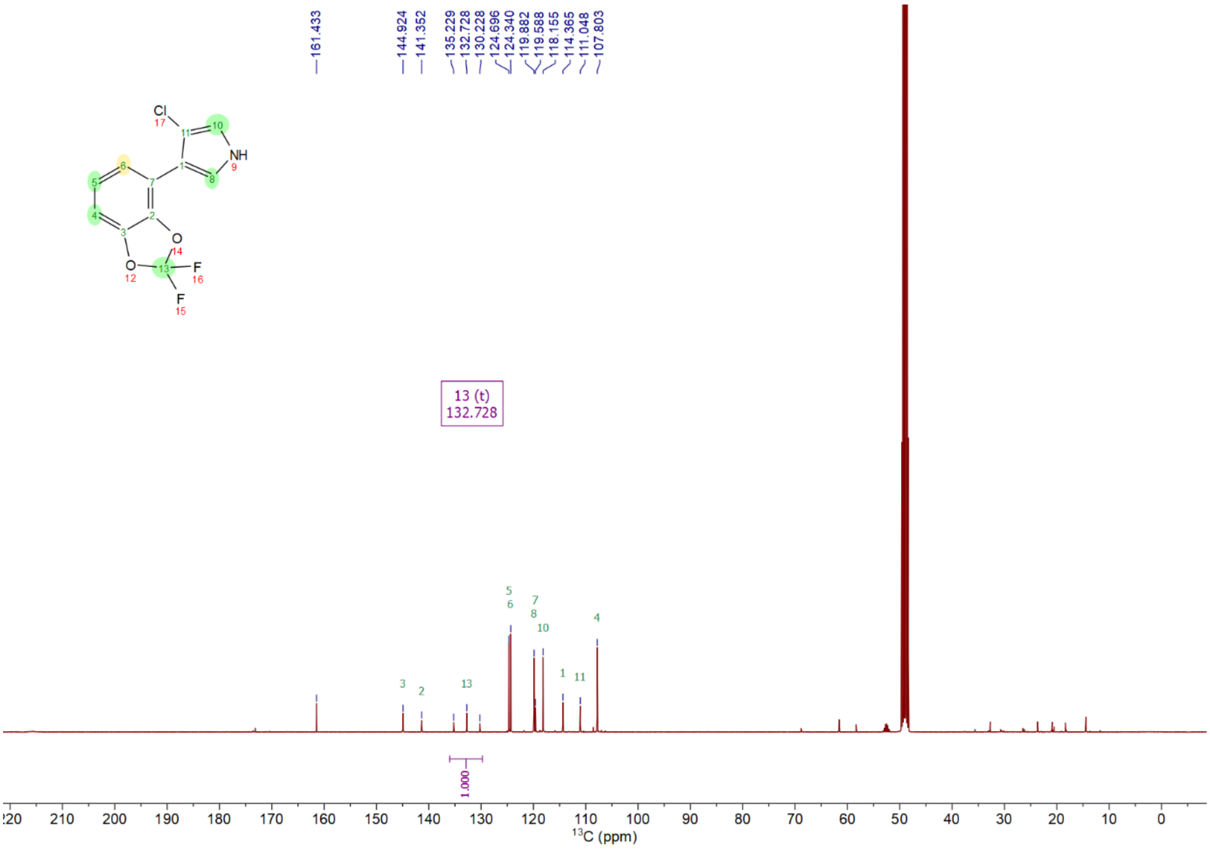

Figure S85. ^{1}H and ^{13}C NMR spectrum for product **17**.

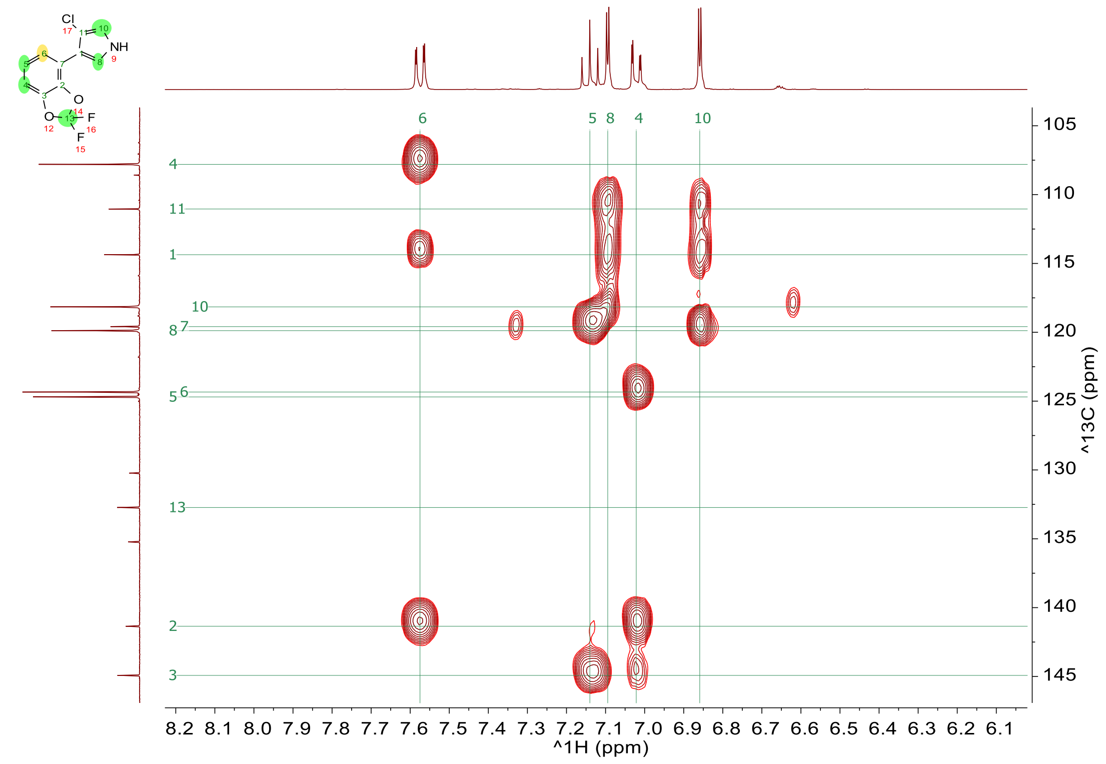

Figure S86. ^{1}H-^{13}C HMBC spectrum for product **17**

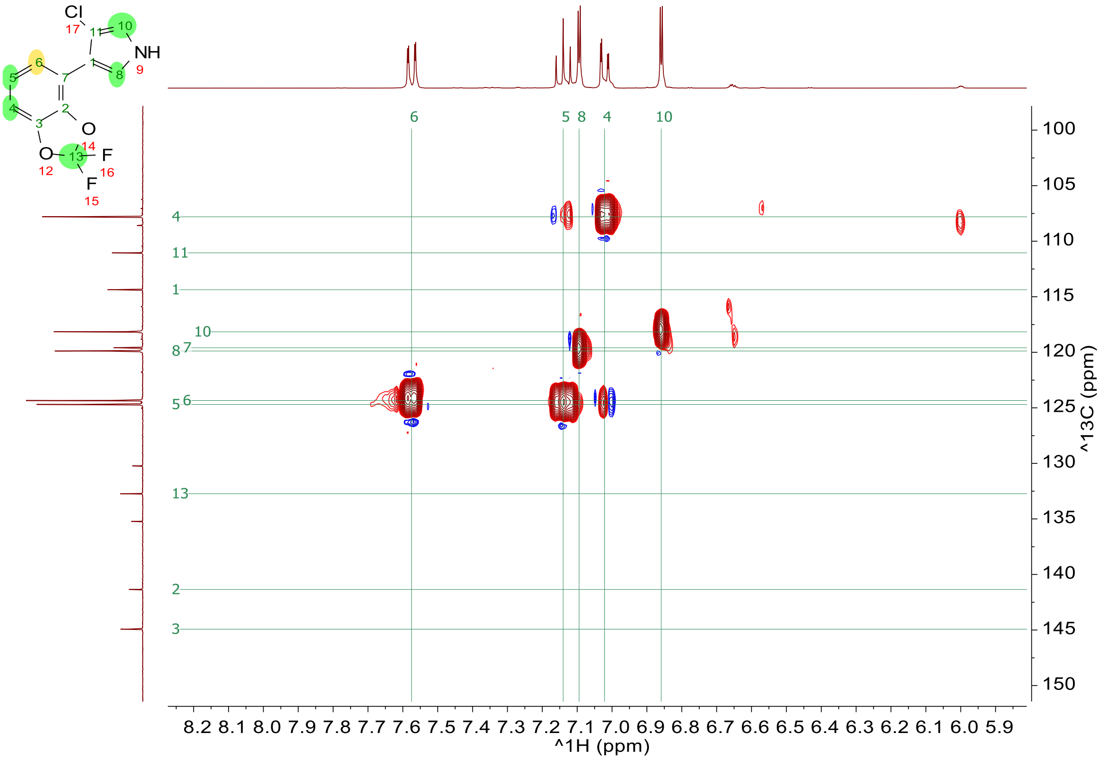

Figure S87. ^{1}H-^{13}C HSQC spectrum for product **17**

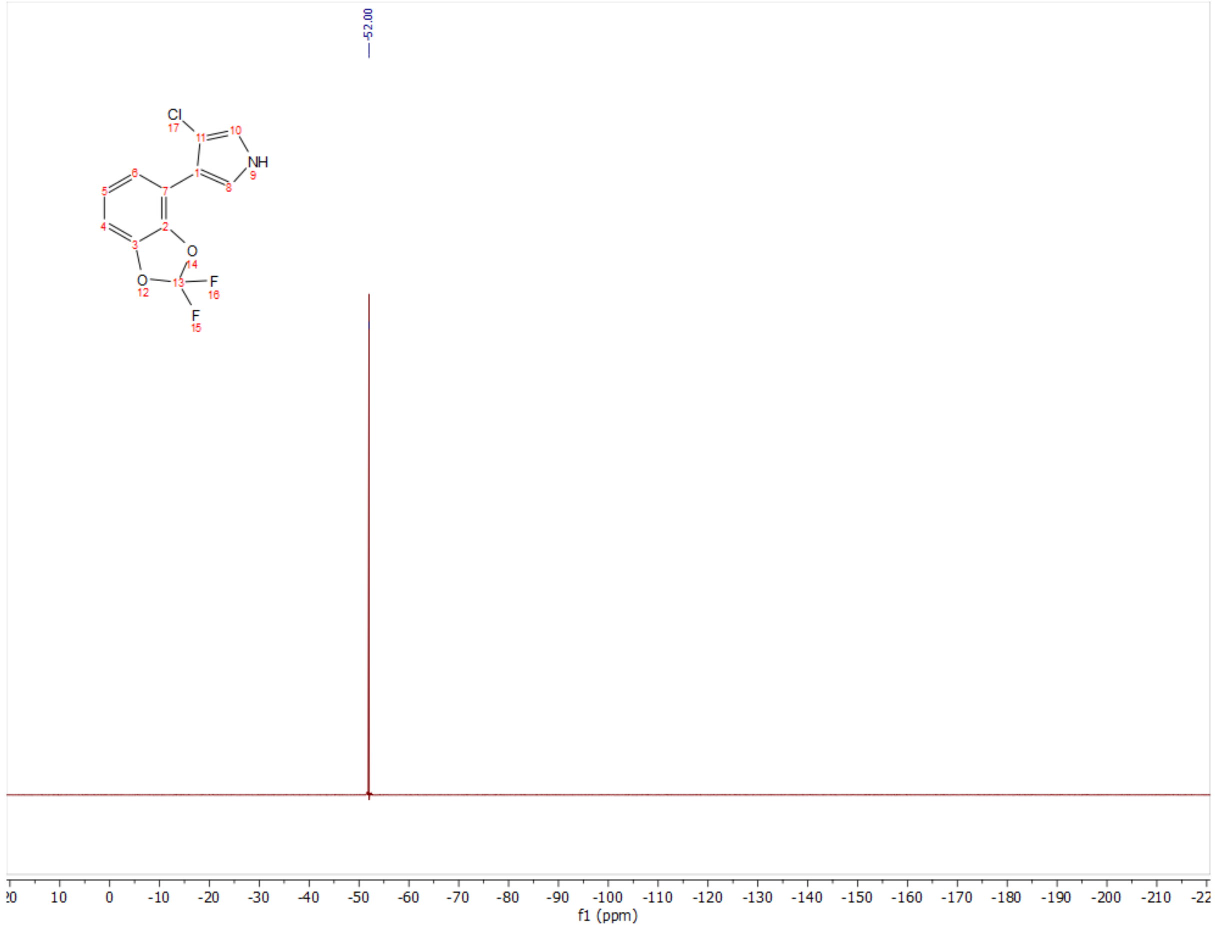

Figure S88. ^{19}F NMR spectrum for product **17**.

b. NMR Spectra of PmC Substrates & Standard

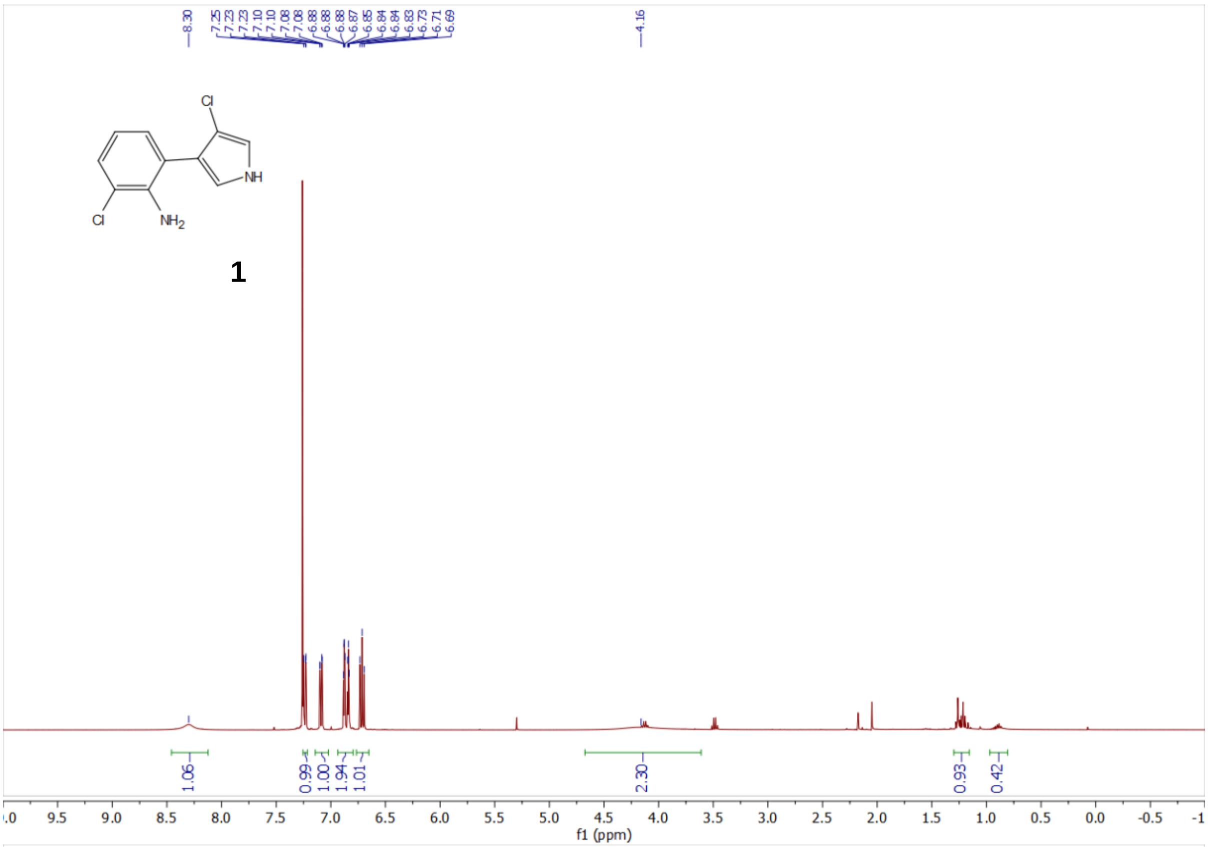

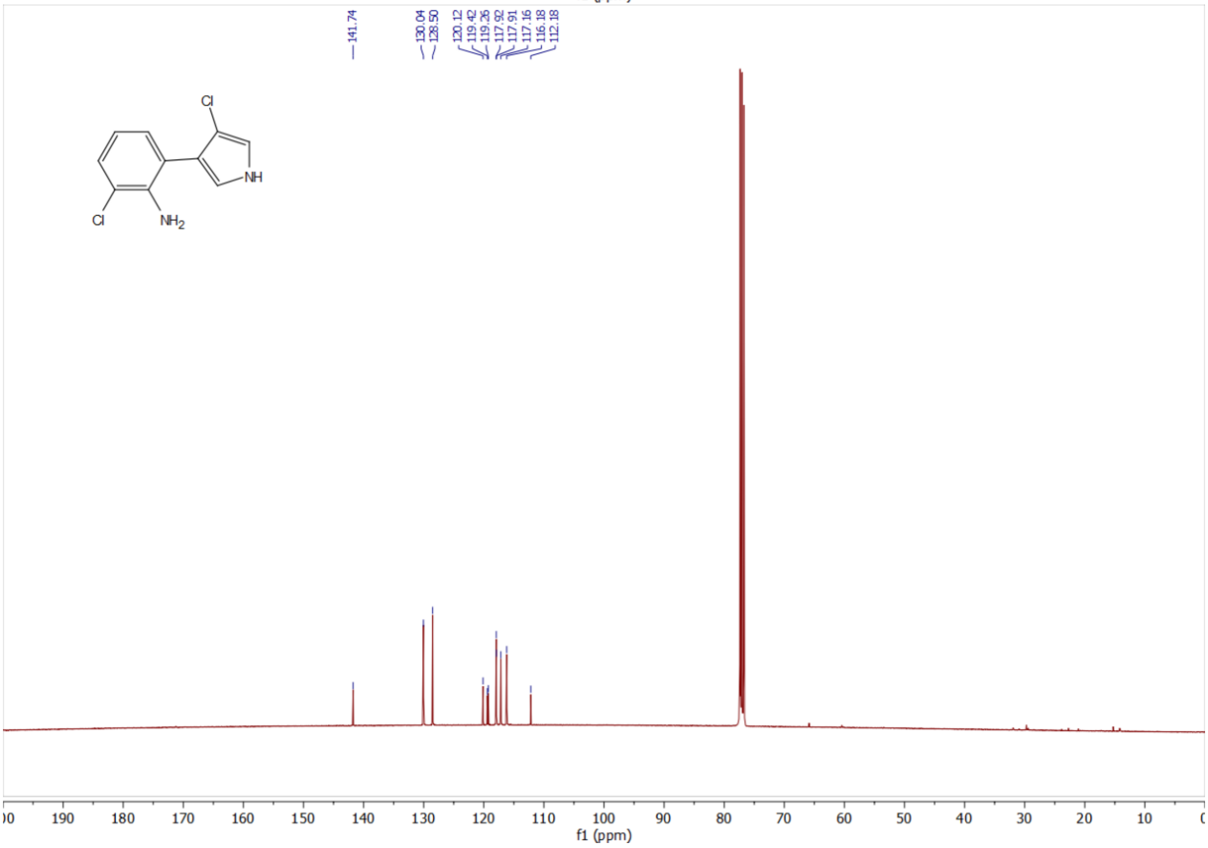

Figure S89. ^{1}H and ^{13}C NMR spectrum for **1**.

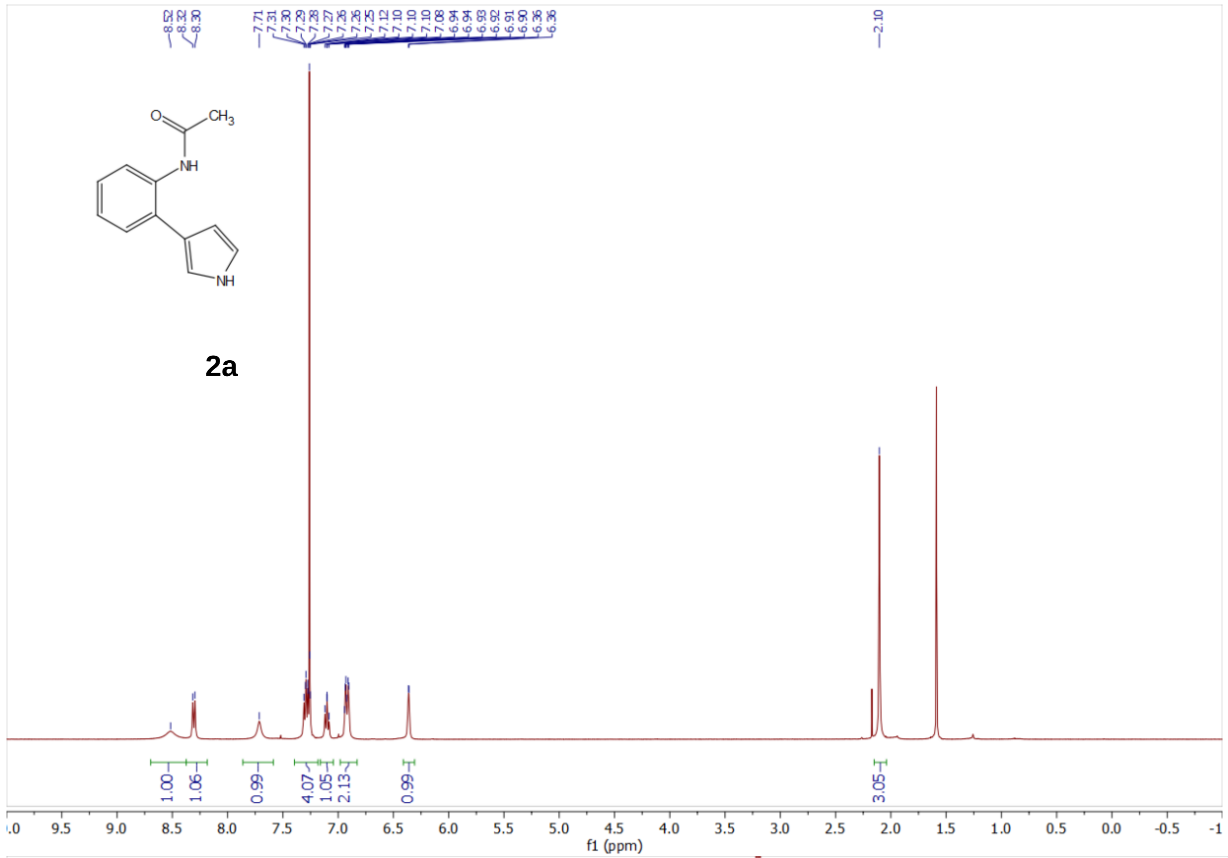

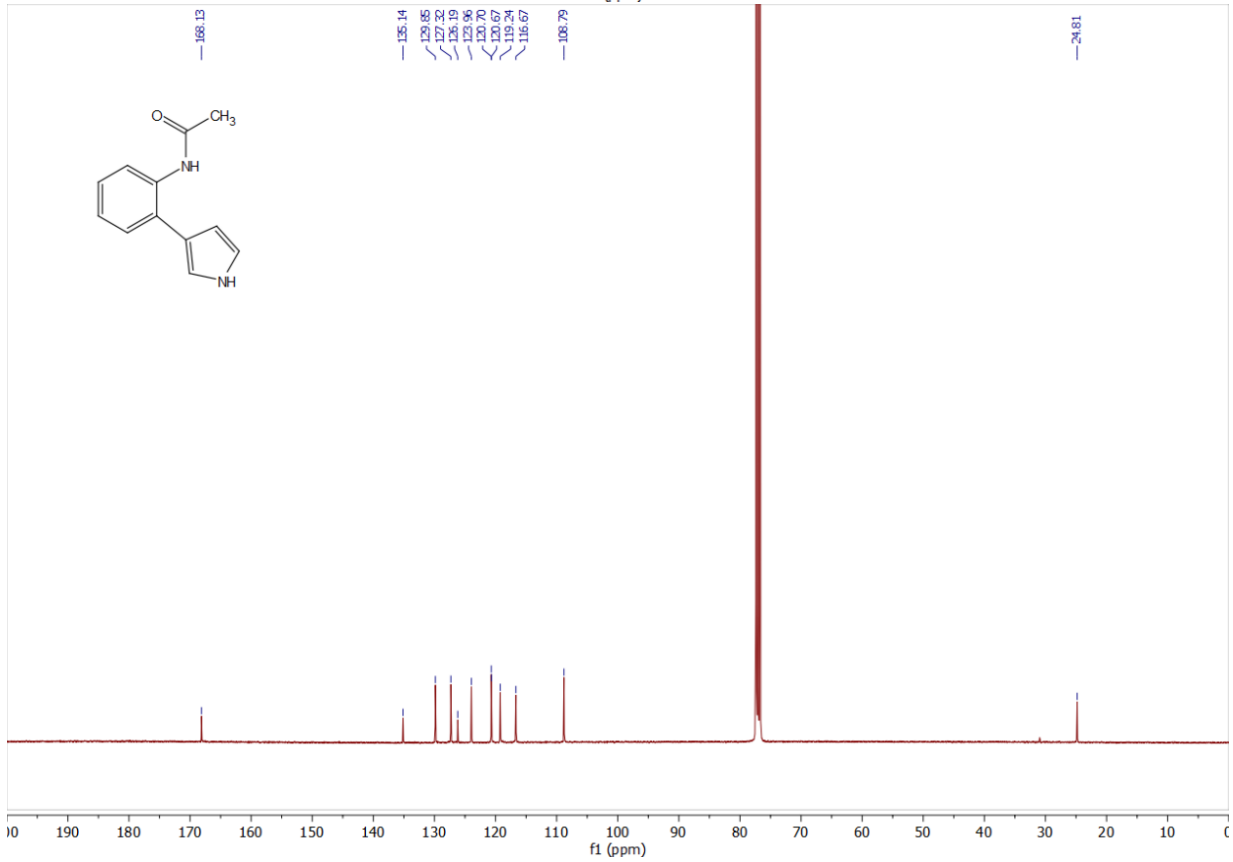

Figure S90. ^{1}H and ^{13}C NMR spectrum for **2a**.

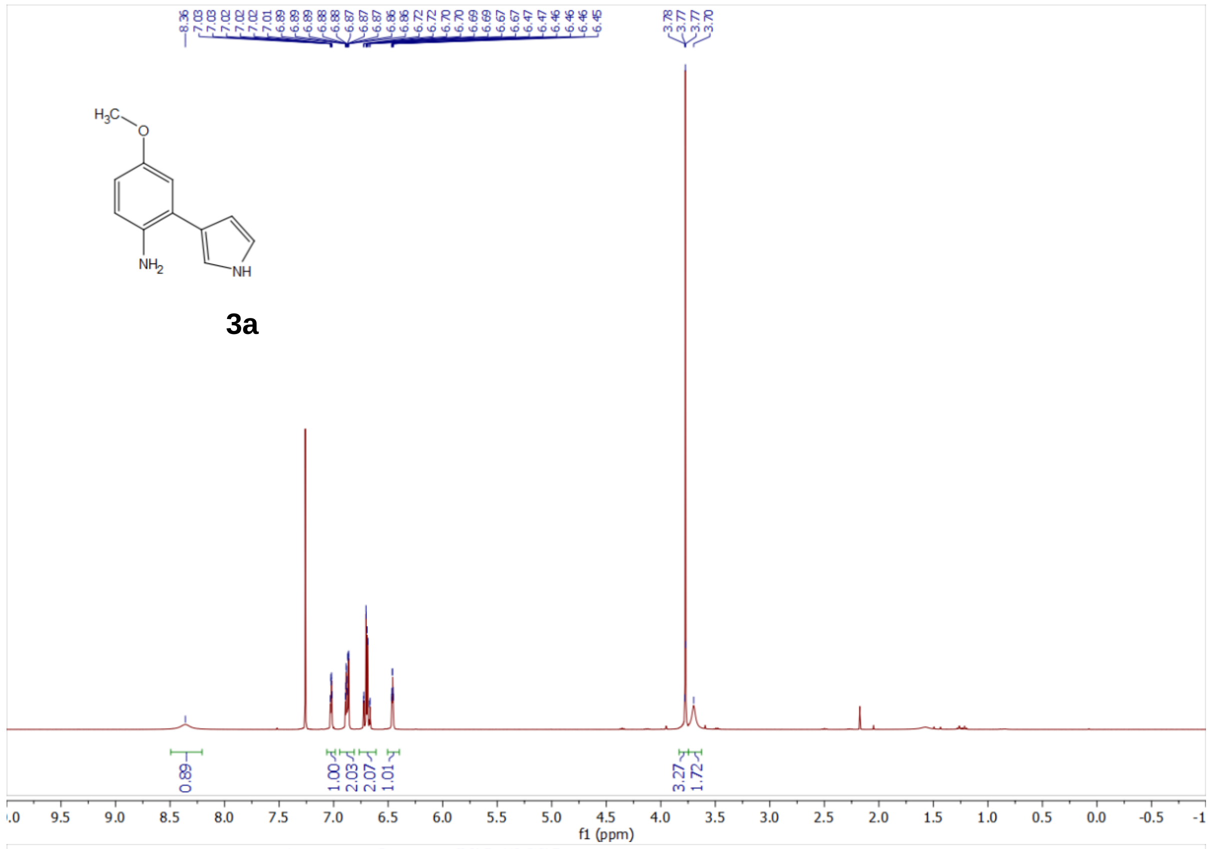

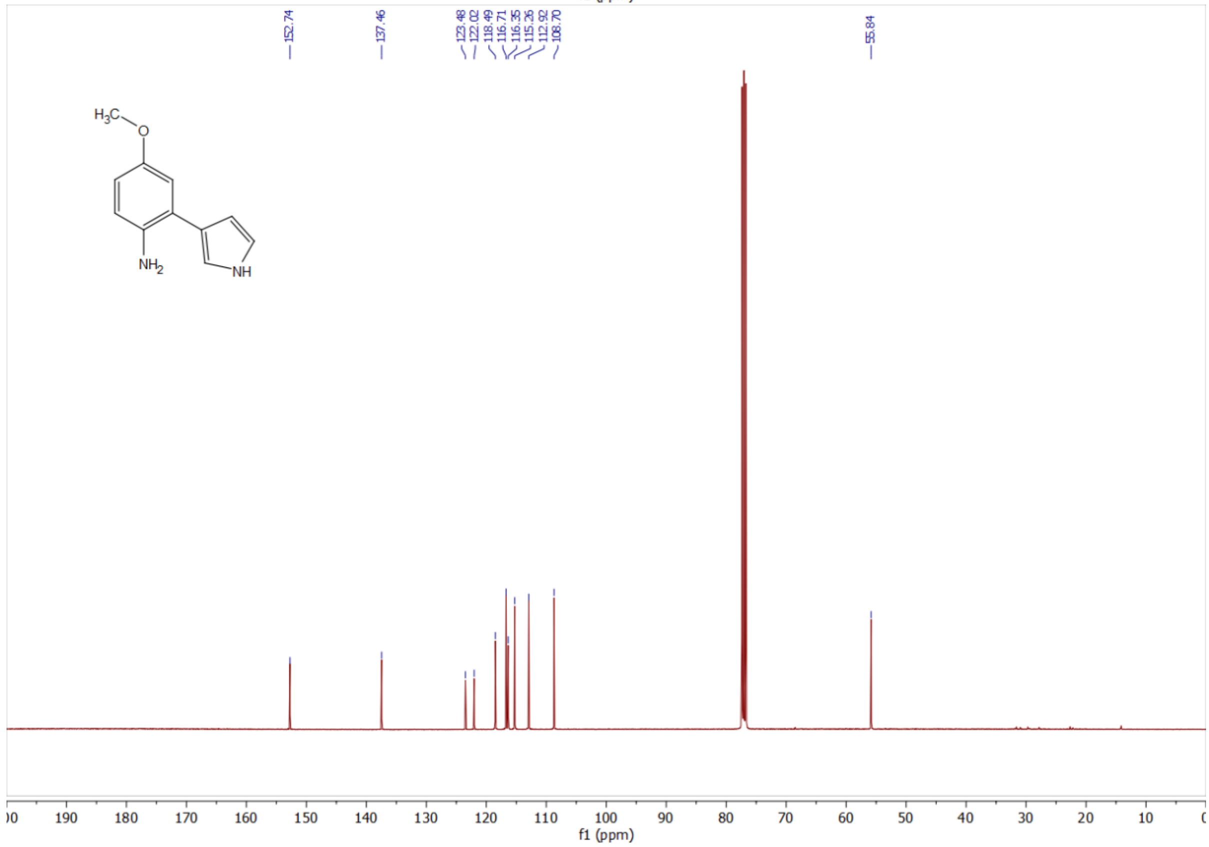

Figure S91. ^{1}H and ^{13}C NMR spectrum for **3a**.

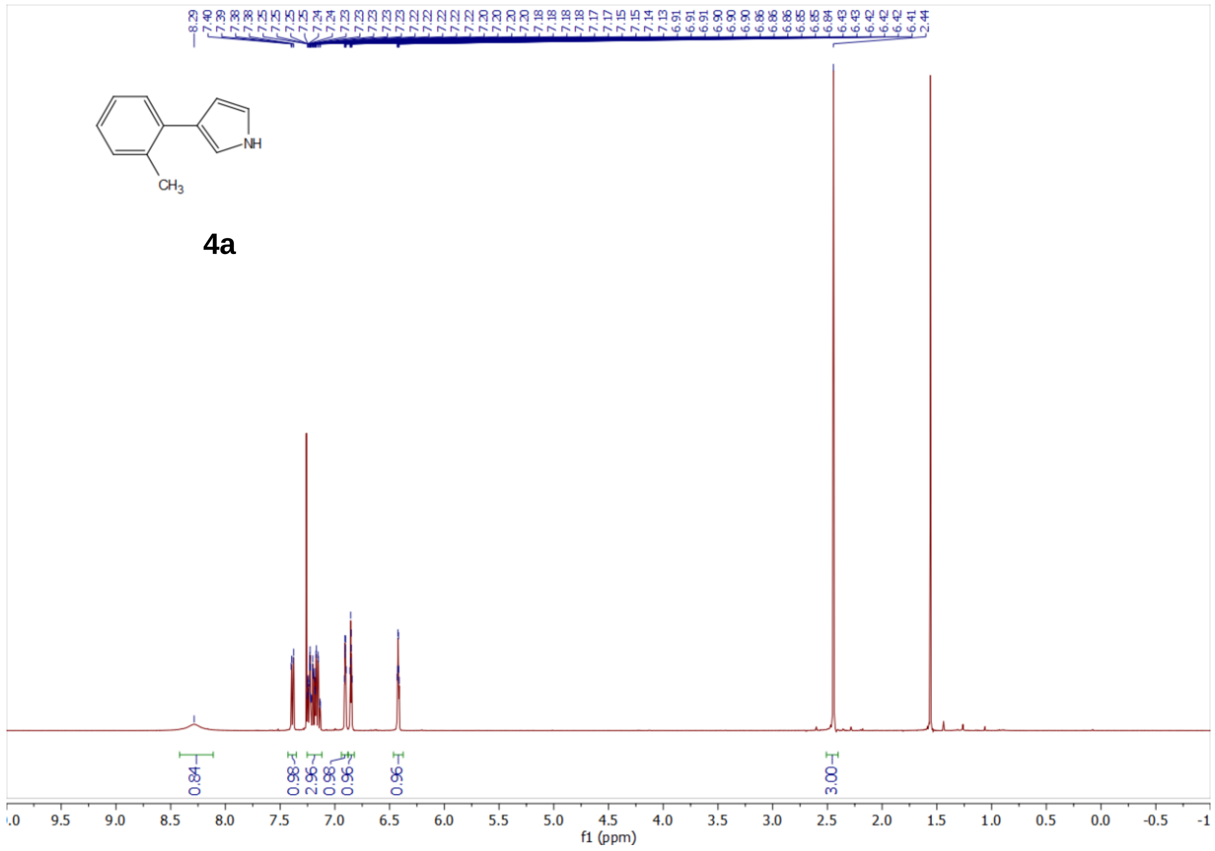

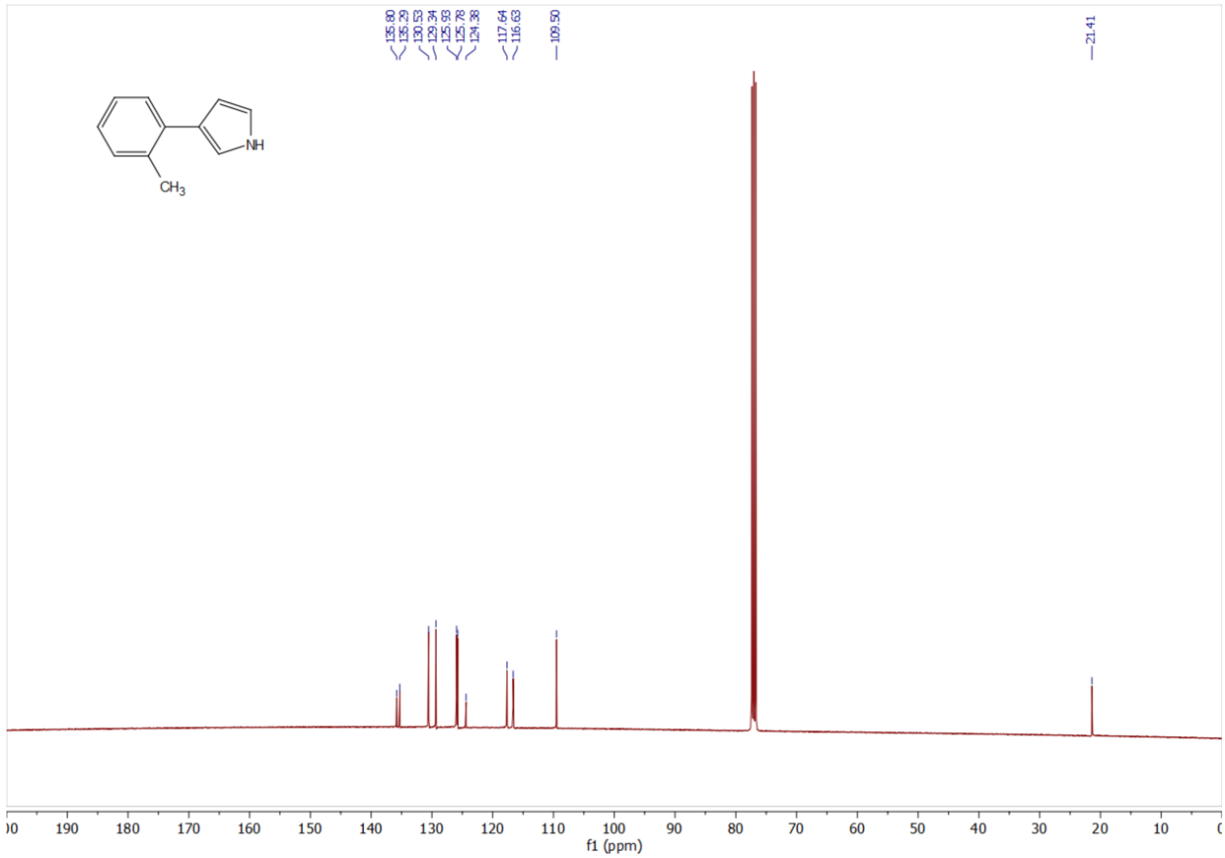

Figure S92. ^{1}H and ^{13}C NMR spectrum for **4a**.

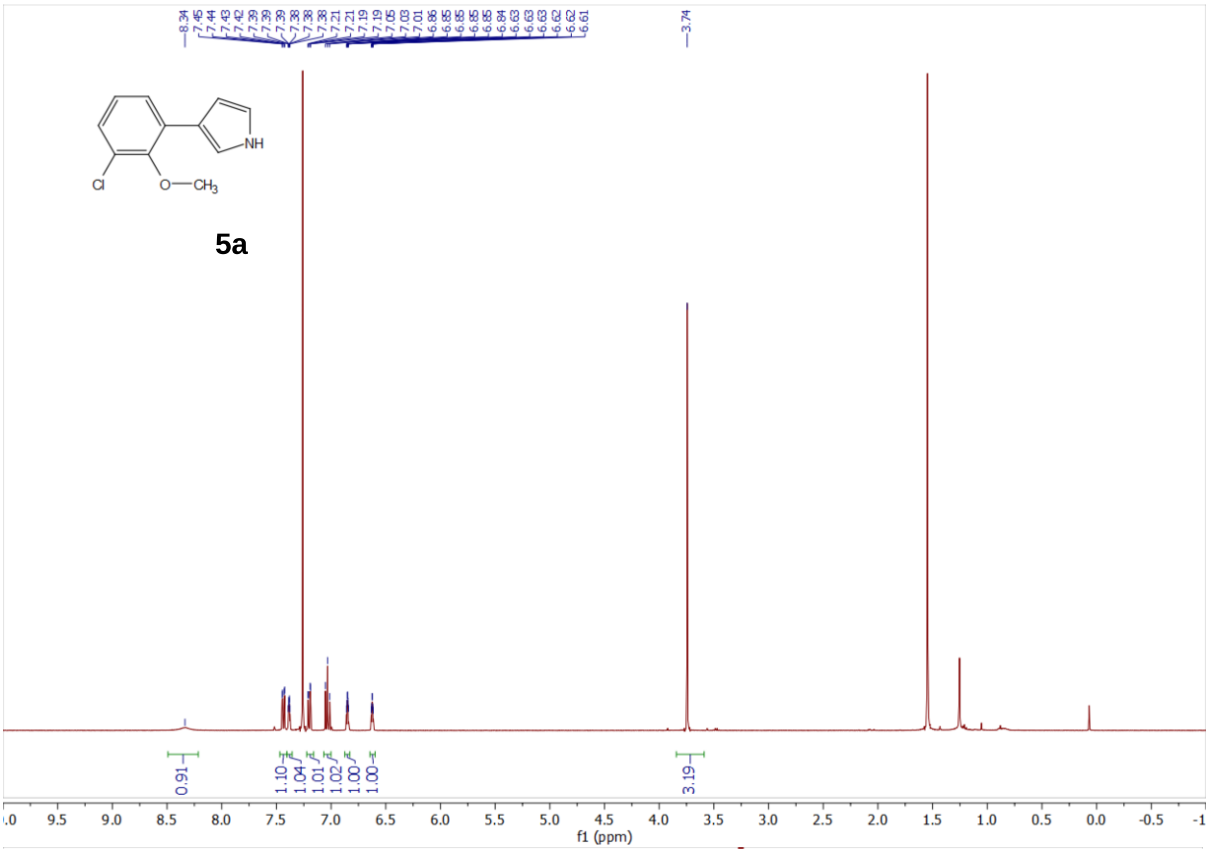

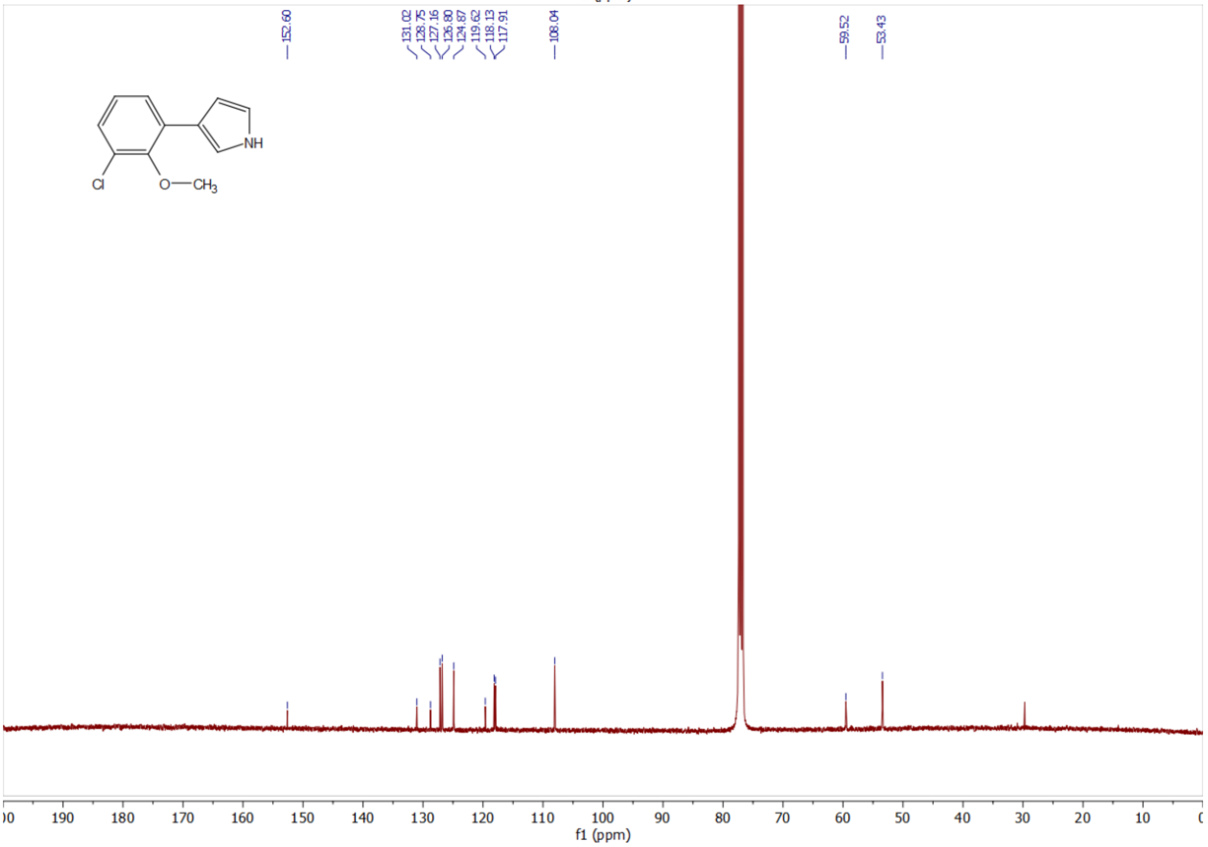

Figure S93. ^{1}H and ^{13}C NMR spectrum for **5a**.

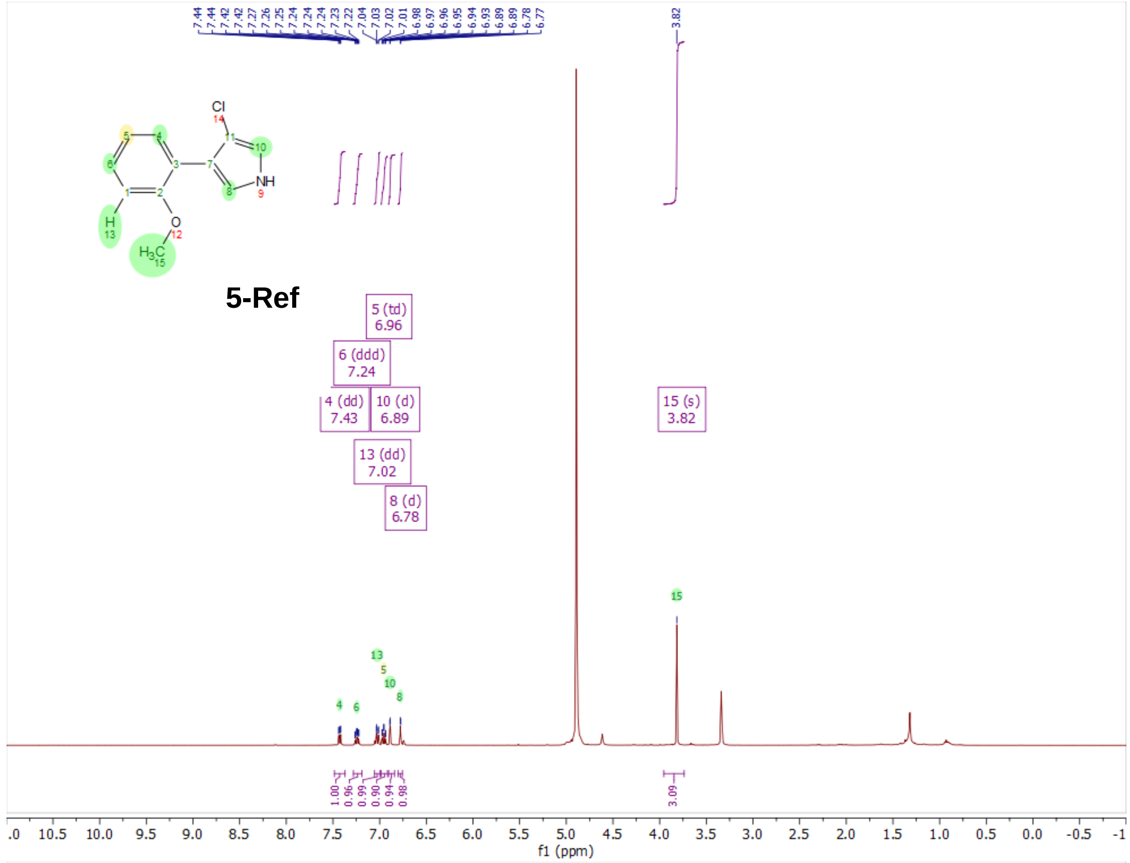

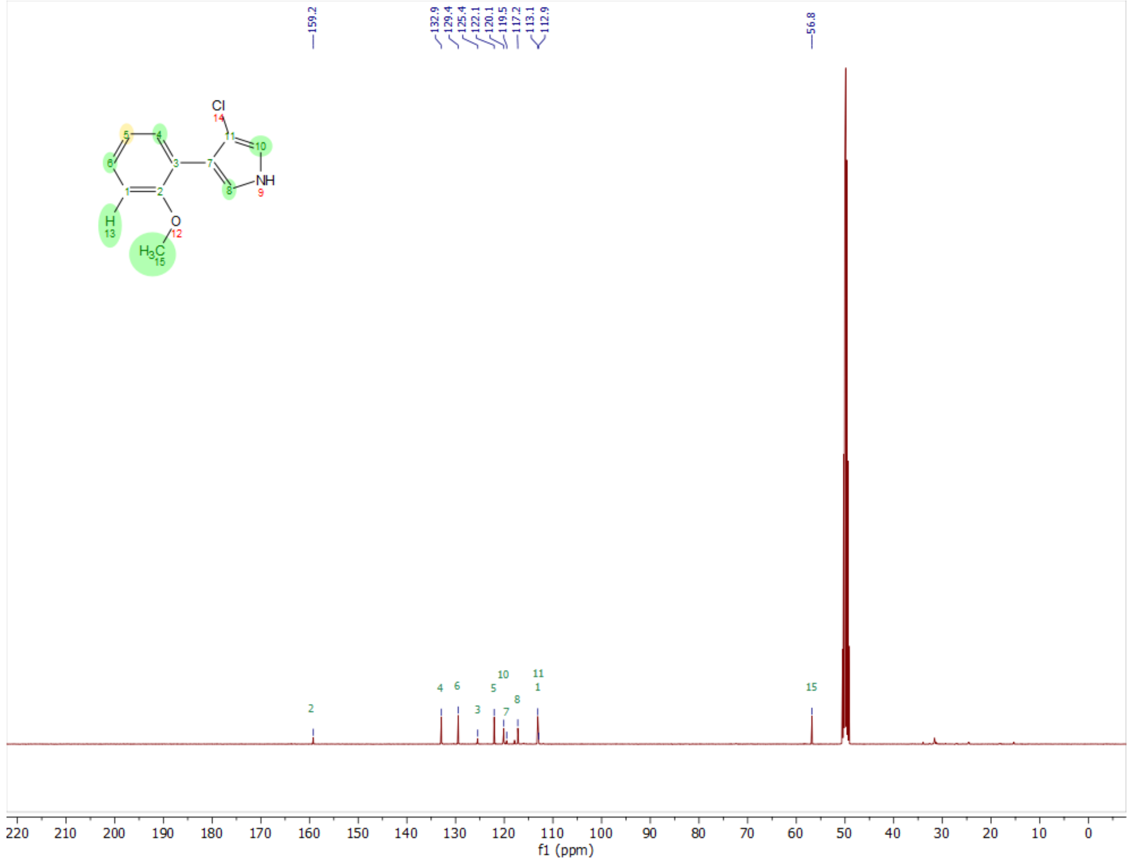

Figure S94. ^{1}H and ^{13}C NMR spectrum for standard **5-Ref**

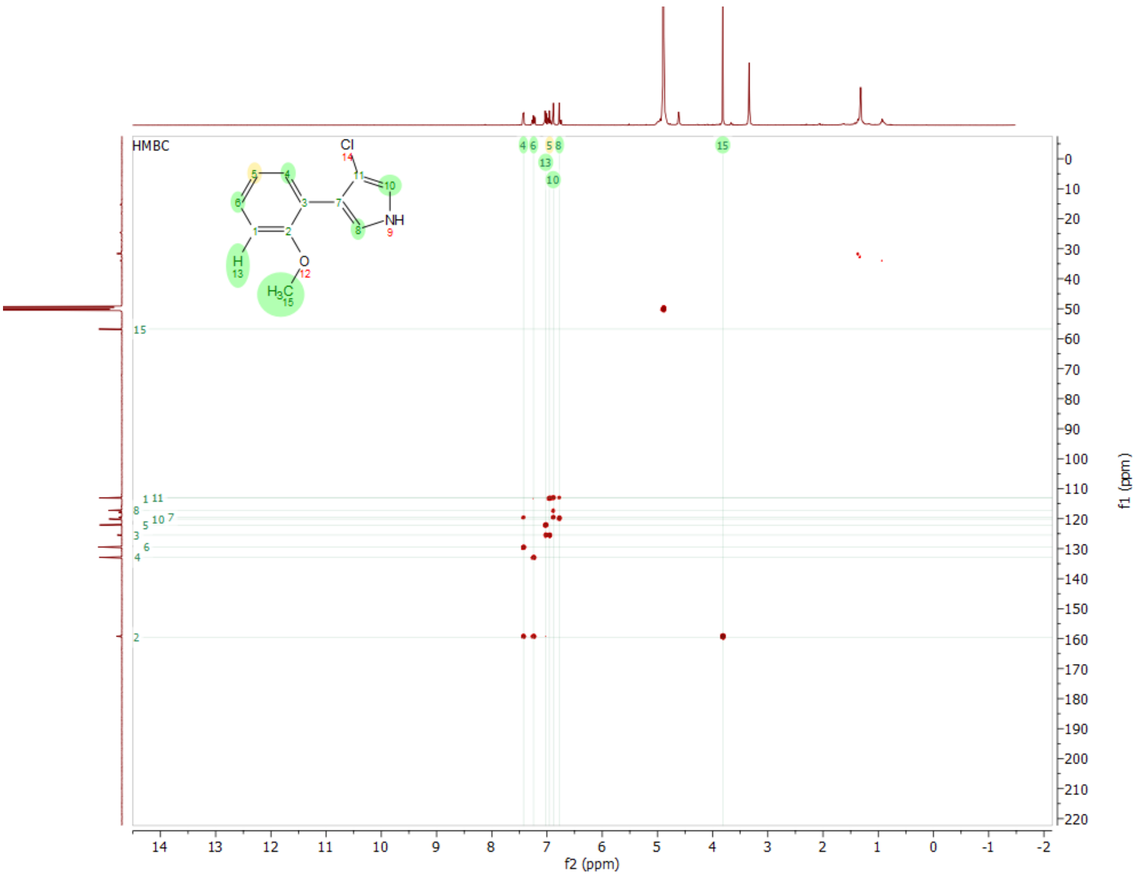

Figure S95. ^{1}H-^{13}C HMBC spectrum for standard **5-Ref**

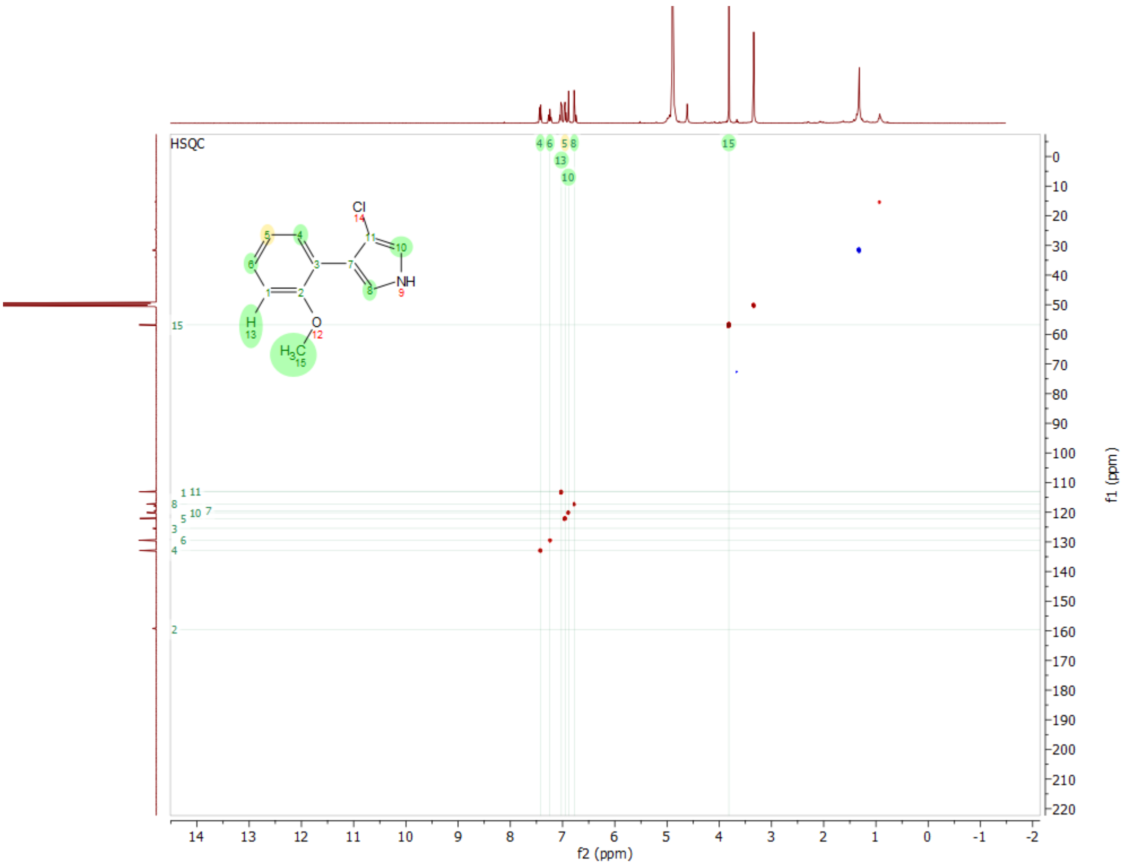

Figure S96. ^{1}H-^{13}C HSQC spectrum for standard **5-Ref**

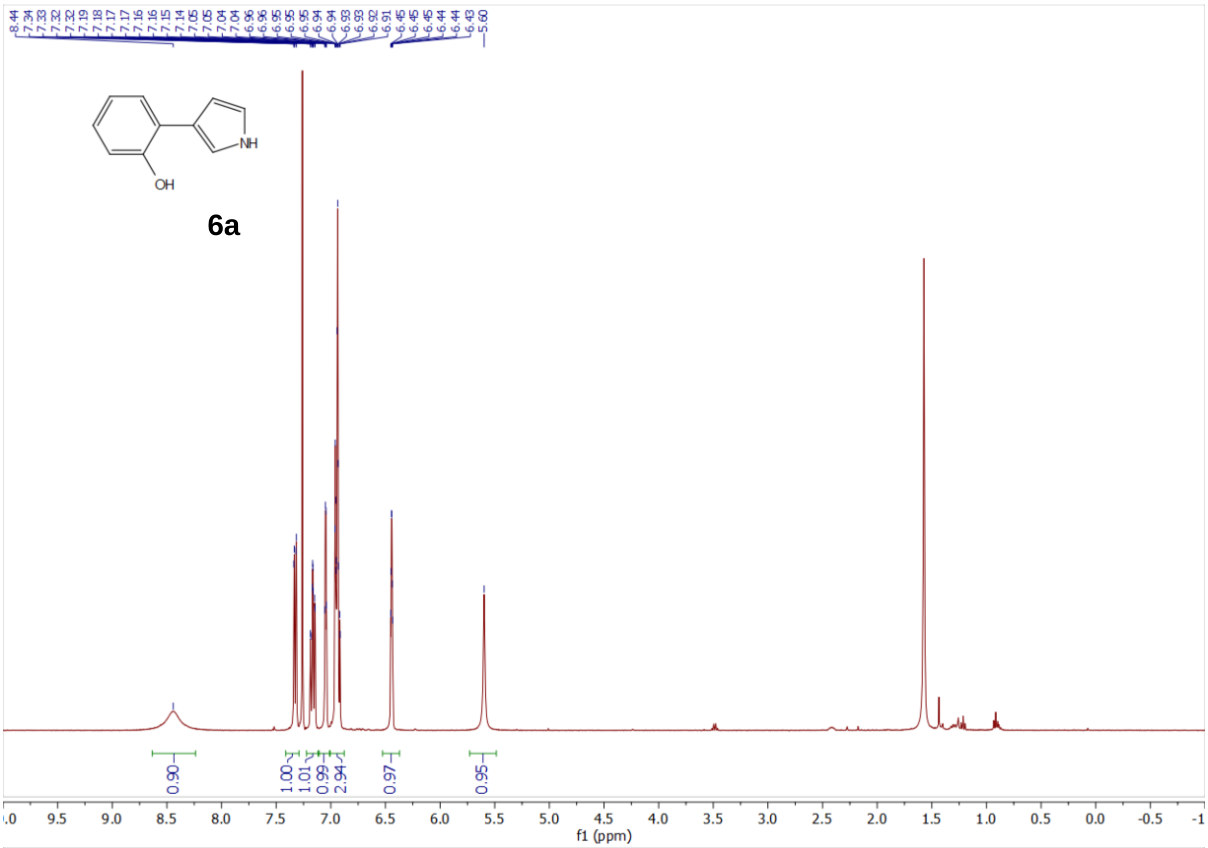

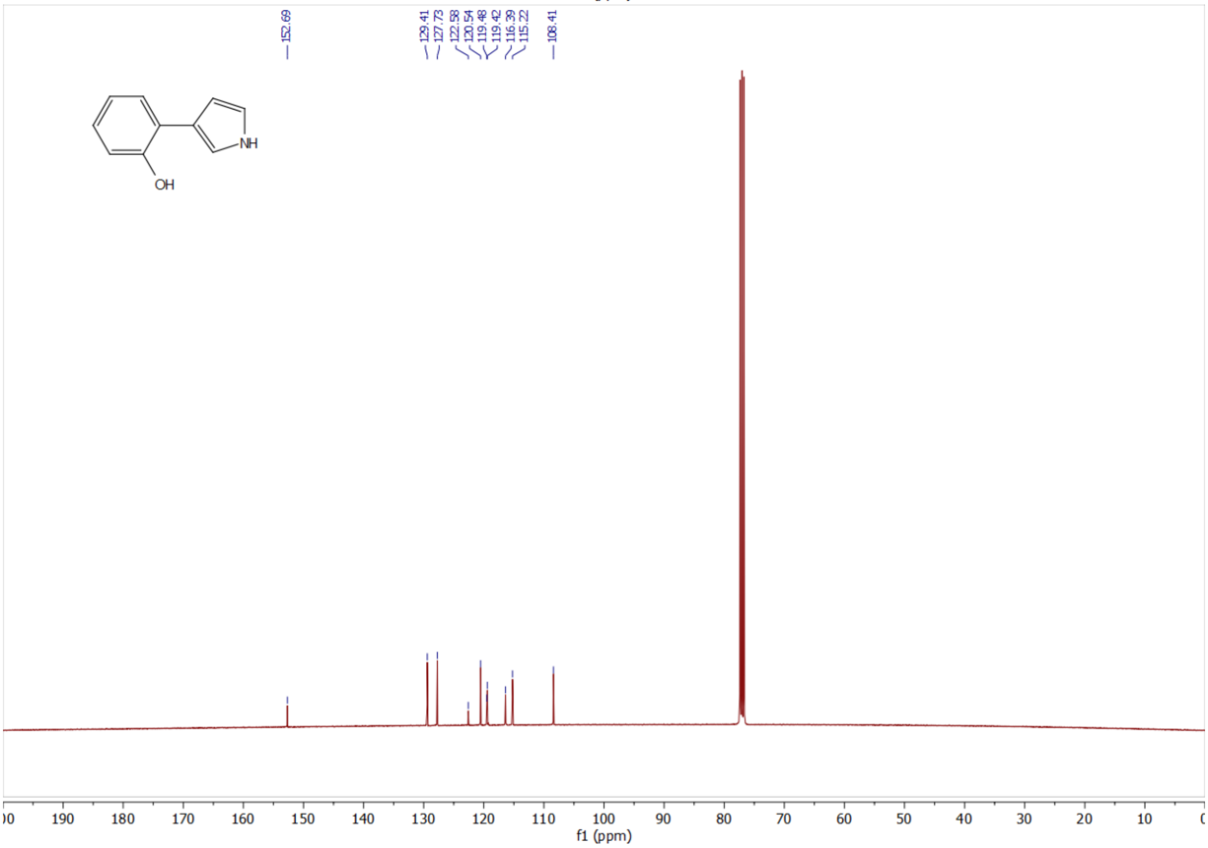

Figure S97. ^{1}H and ^{13}C NMR spectrum for **6a**.

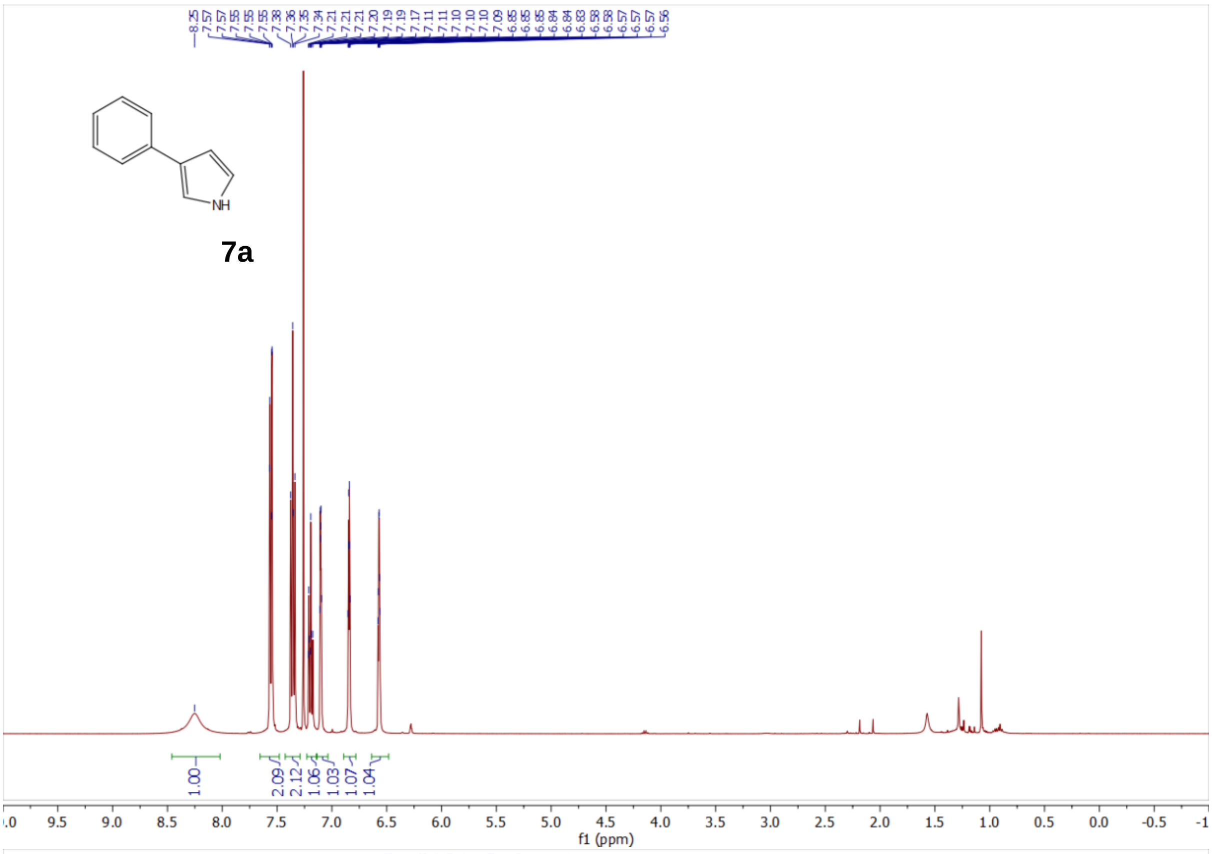

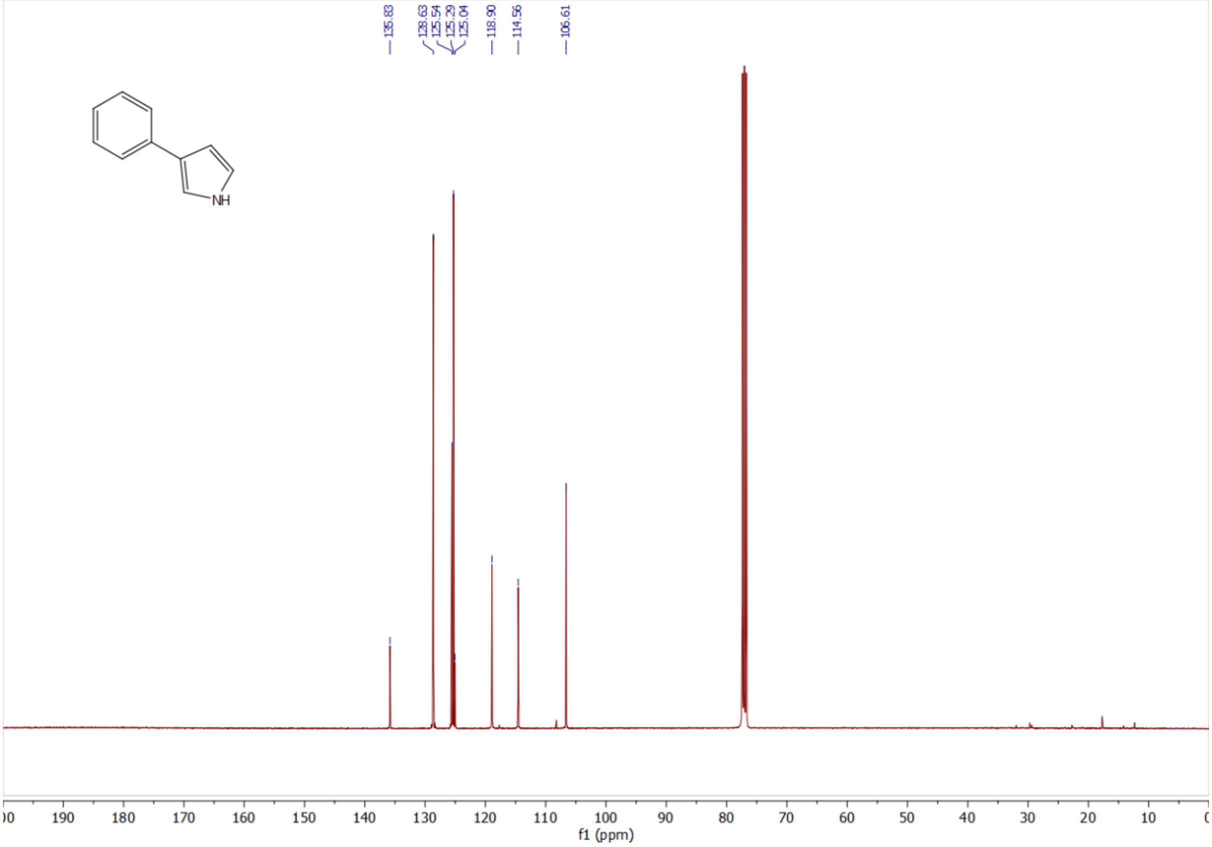

Figure S98. ^{1}H and ^{13}C NMR spectrum for **7a**.

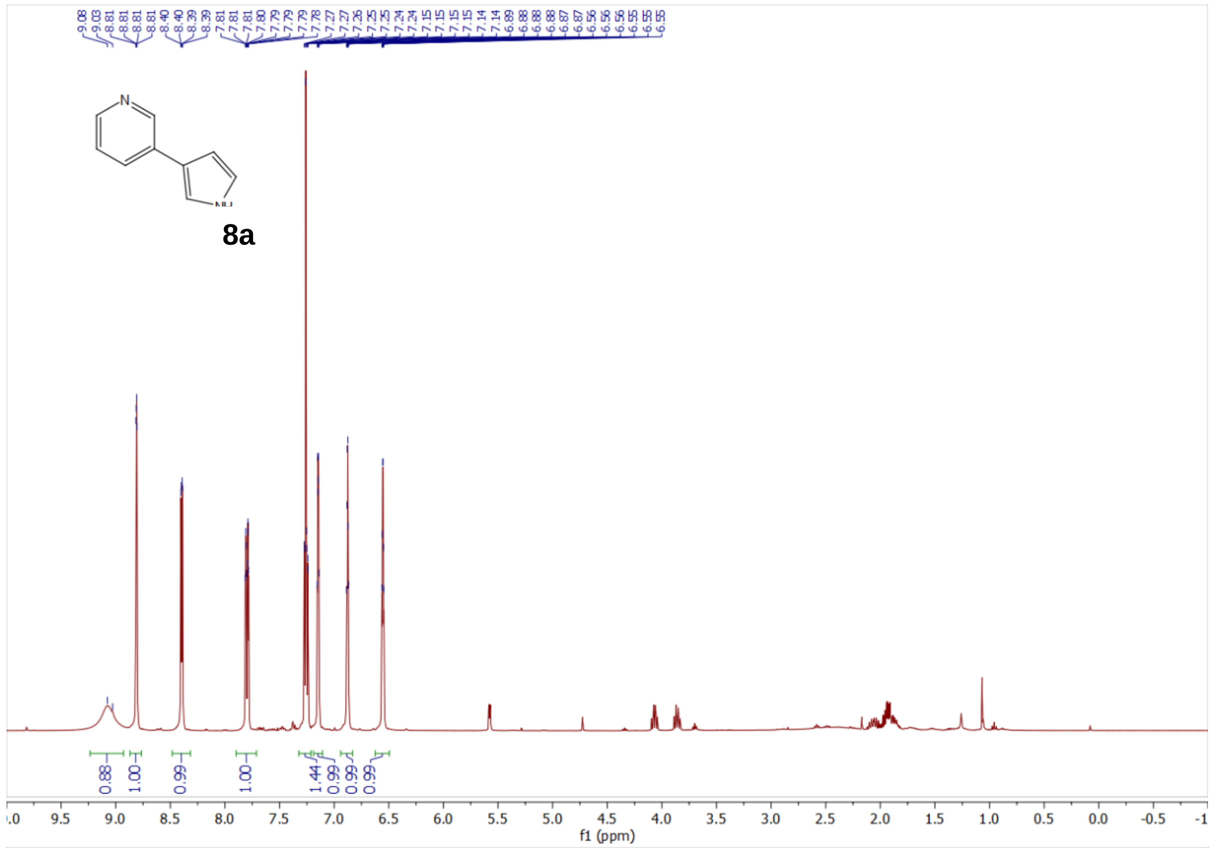

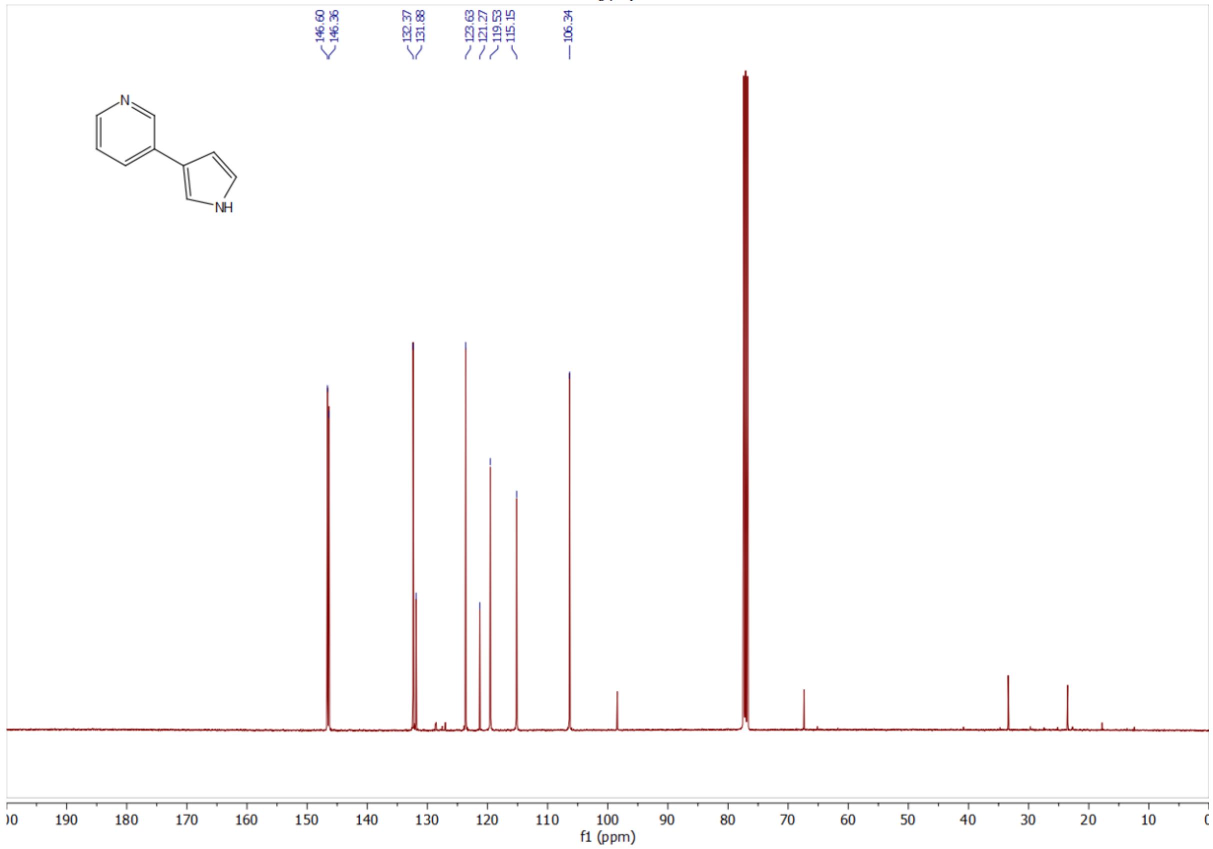

Figure S99. ^{1}H and ^{13}C NMR spectrum for **8a**.

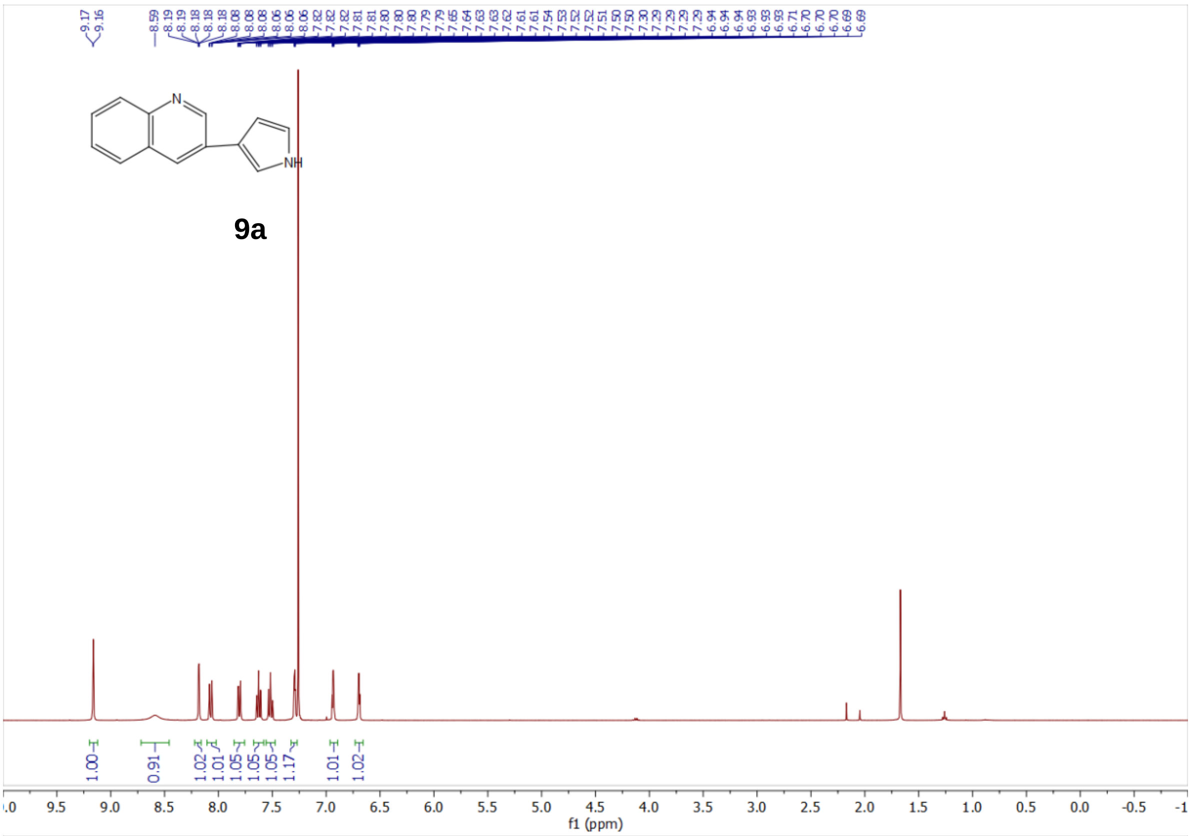

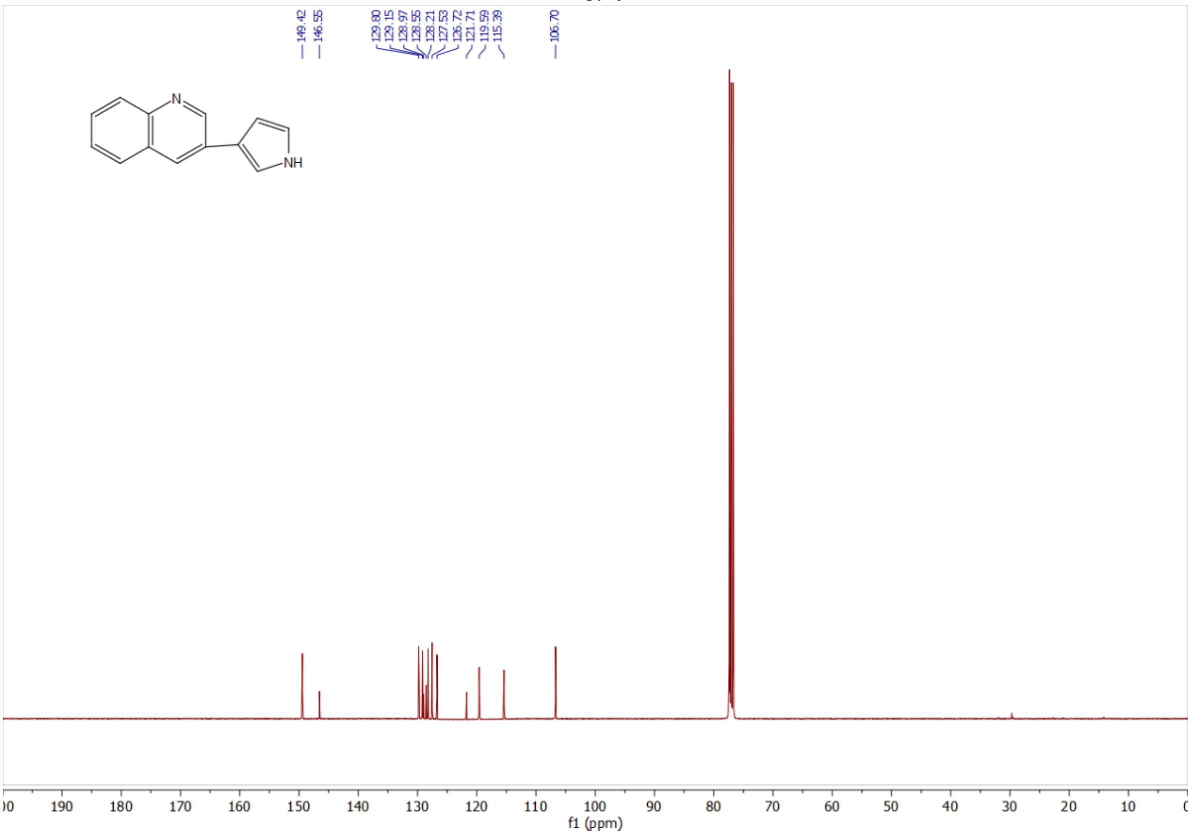

Figure S100. ^{1}H and ^{13}C NMR spectrum for **9a**.

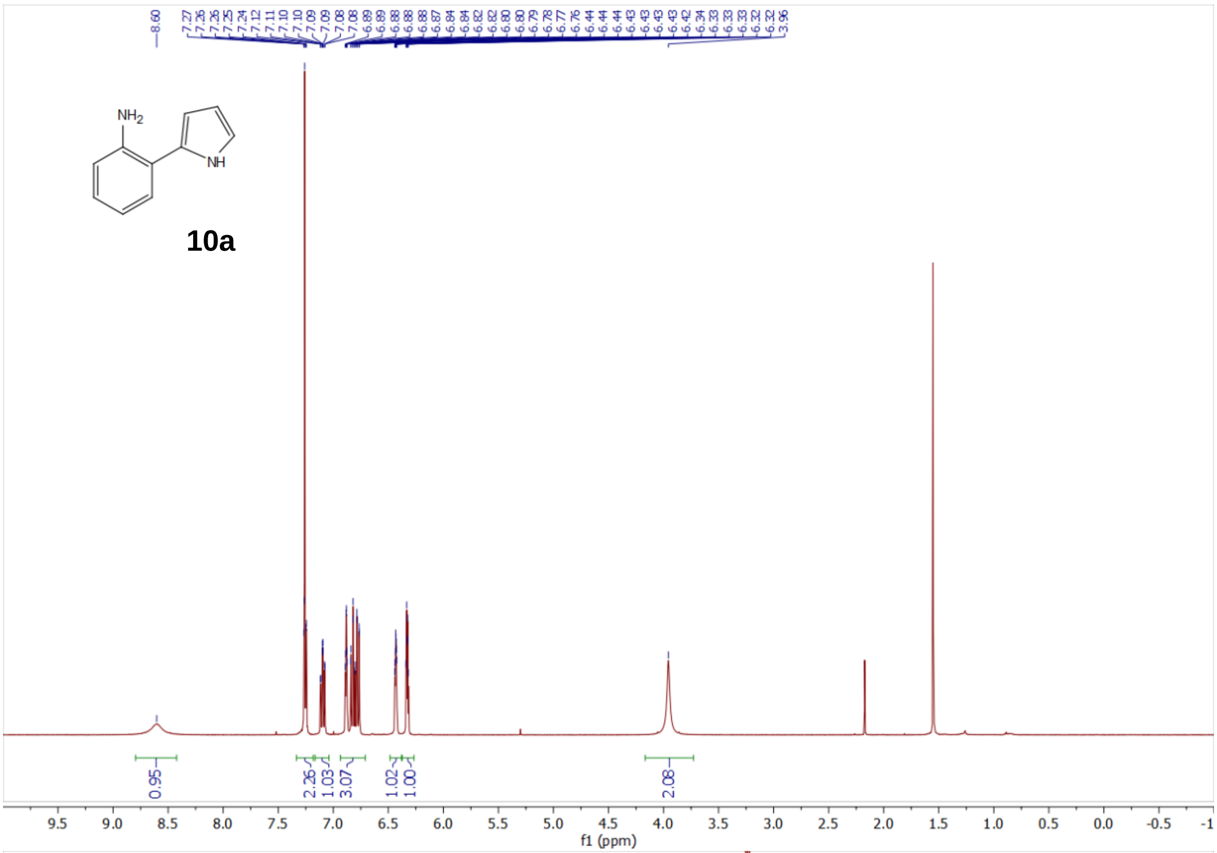

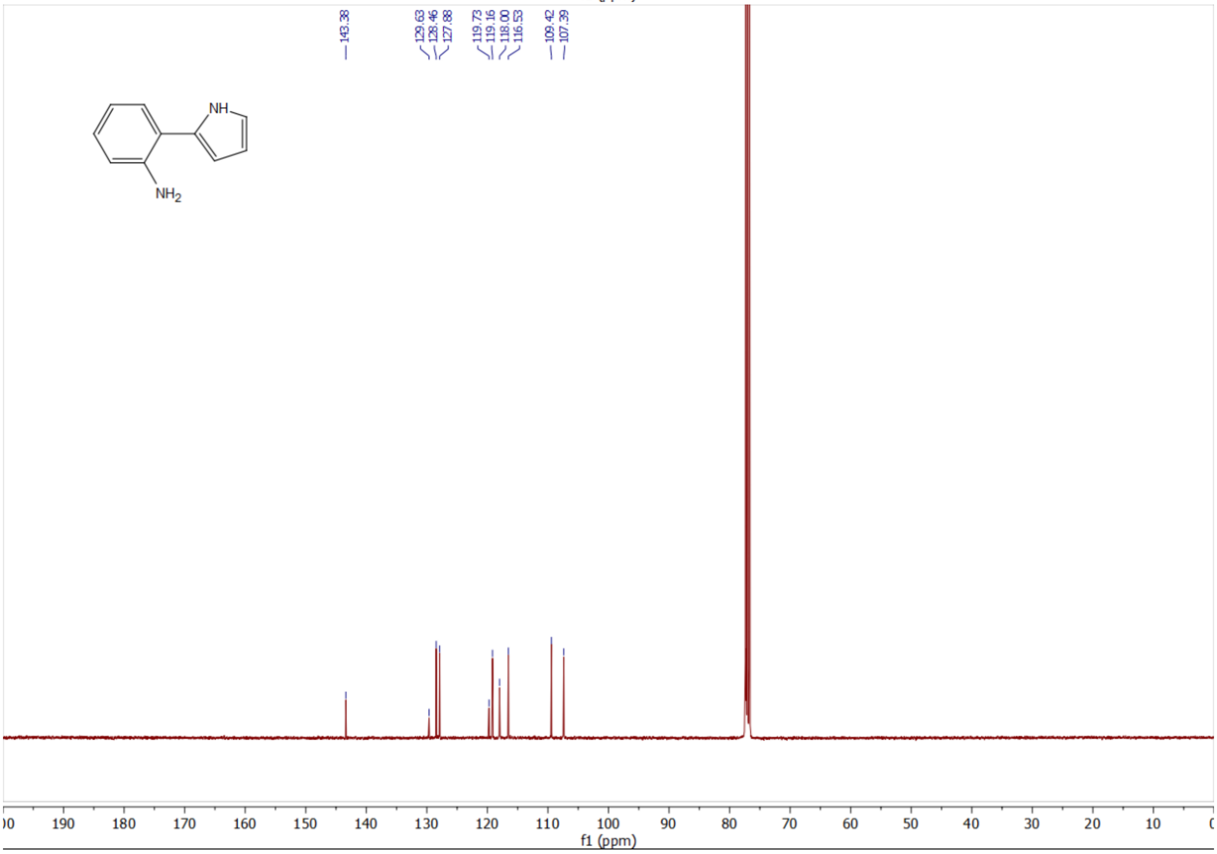

Figure S101. ^{1}H and ^{13}C NMR spectrum for **10a**.

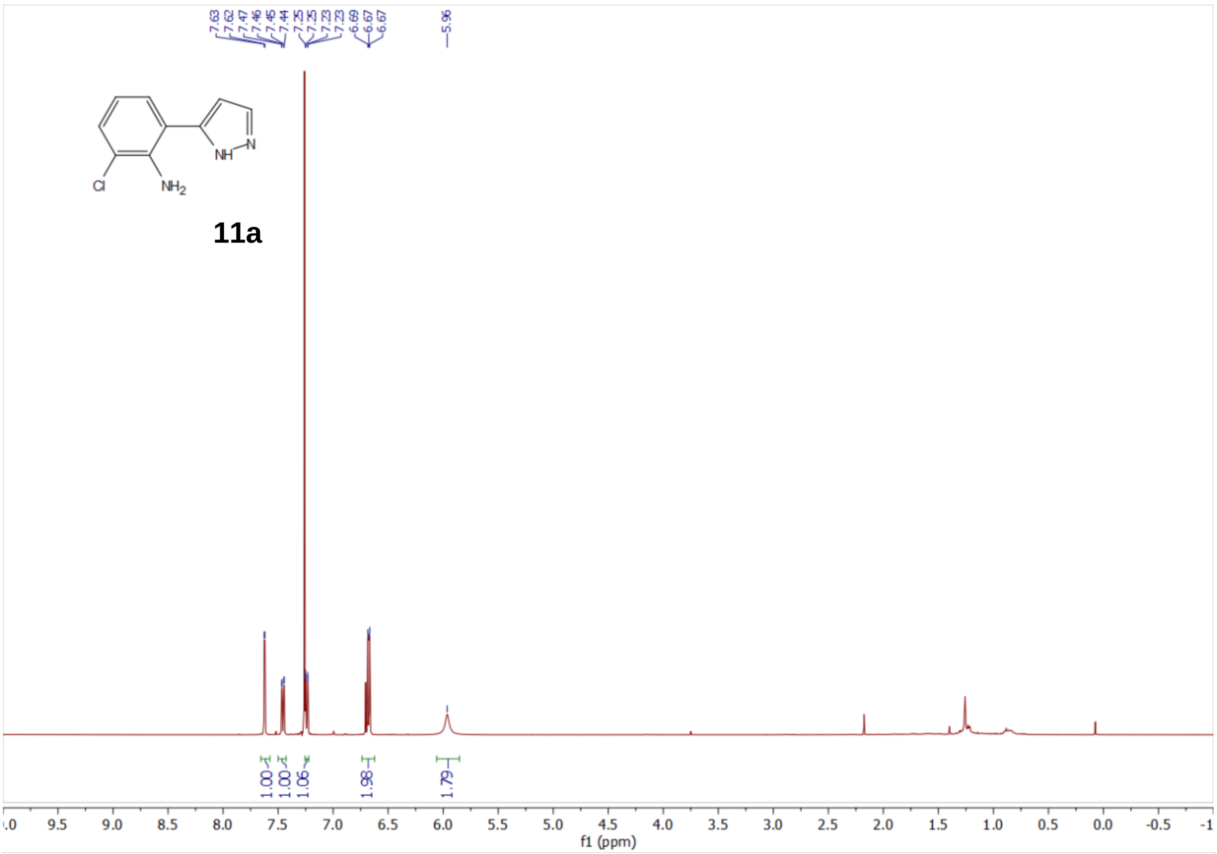

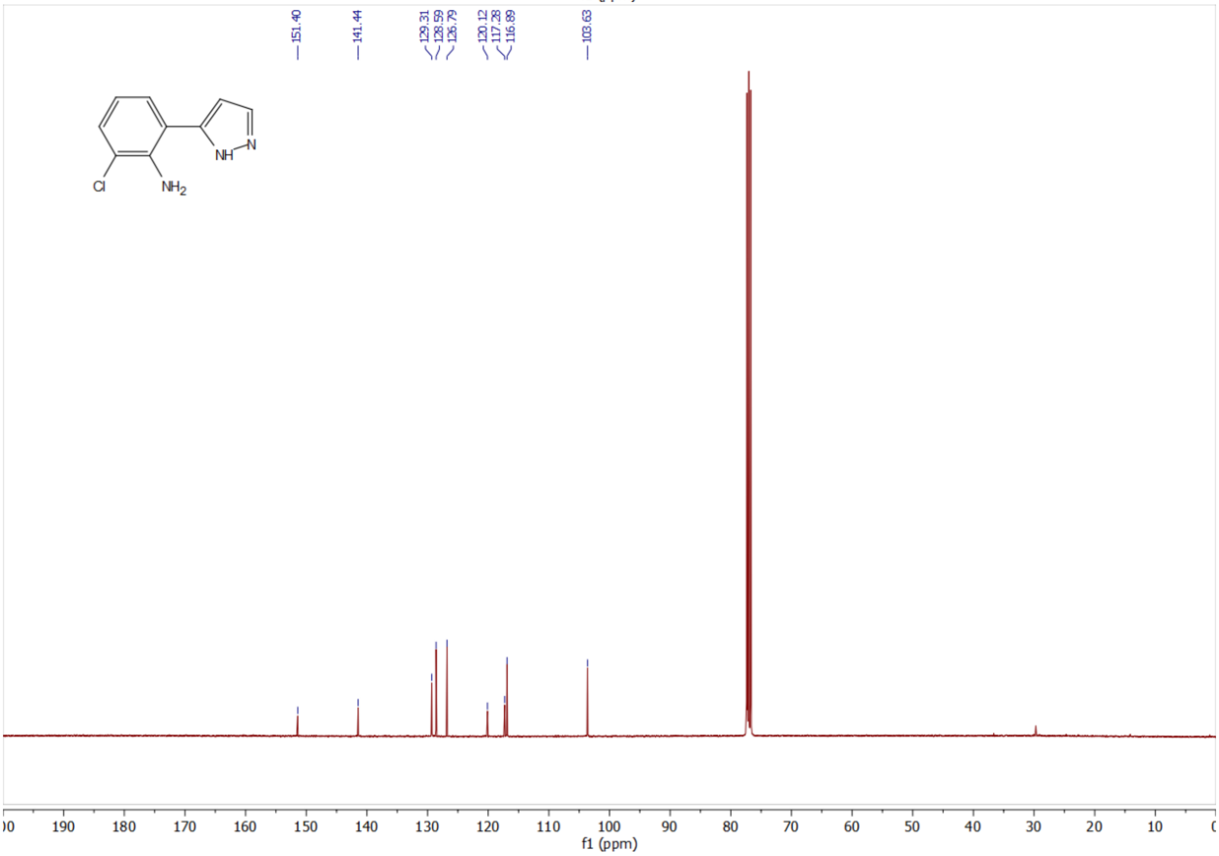

Figure S102. ^{1}H and ^{13}C NMR spectrum for **11a**.

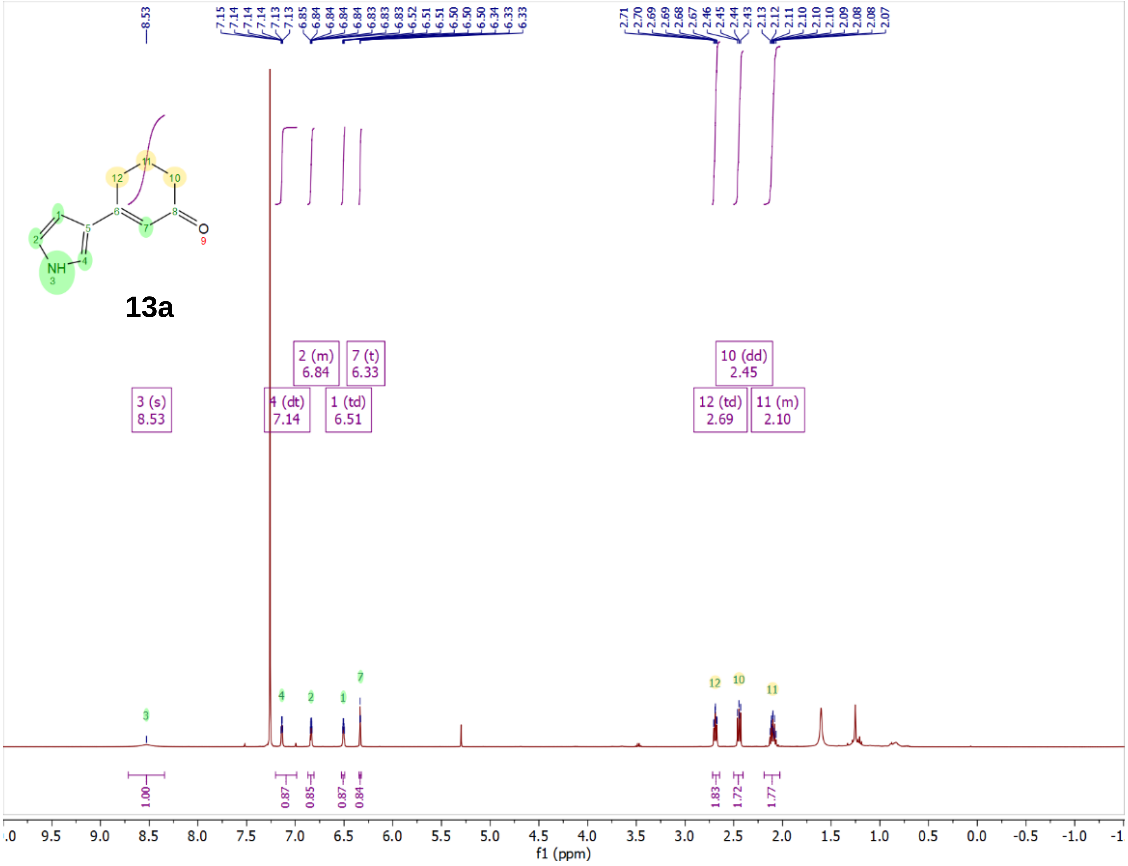

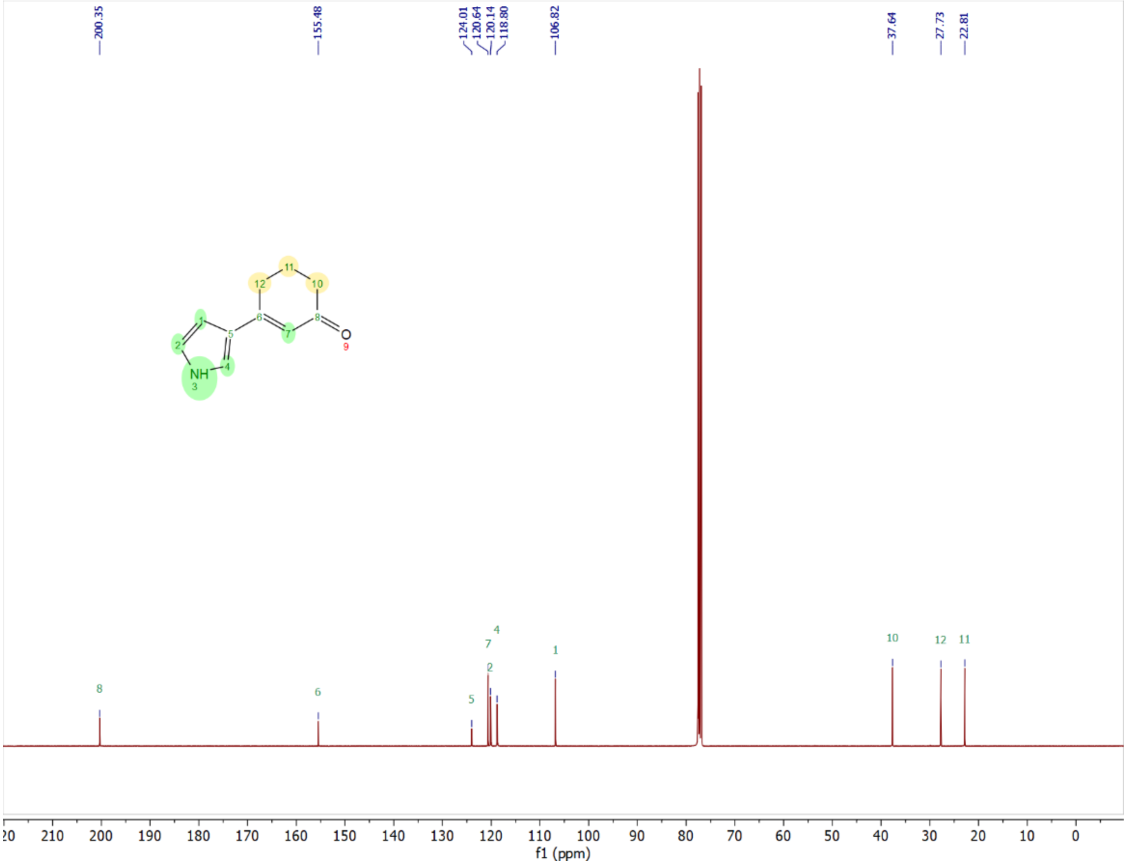

Figure S103. ^{1}H and ^{13}C NMR spectrum for **13a**.

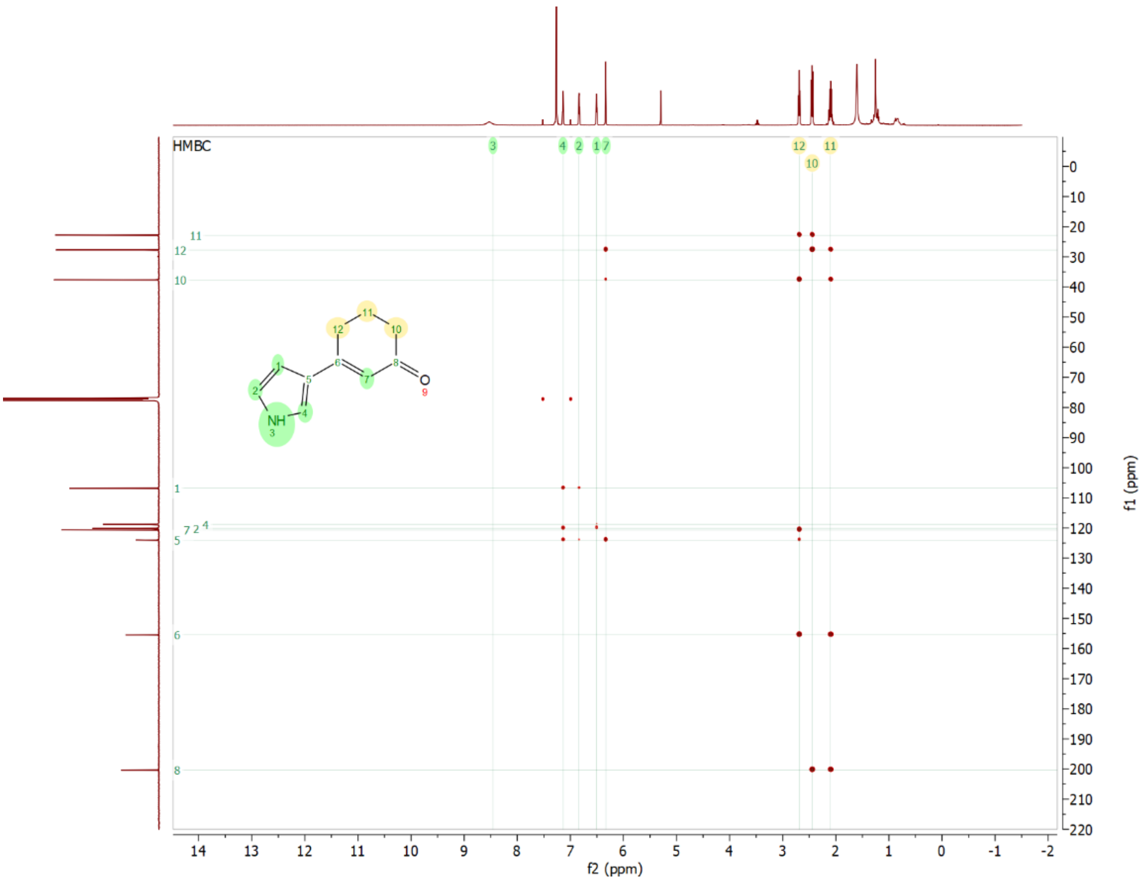

Figure S104. ^{1}H-^{13}C HMBC NMR spectrum for product **13a.**

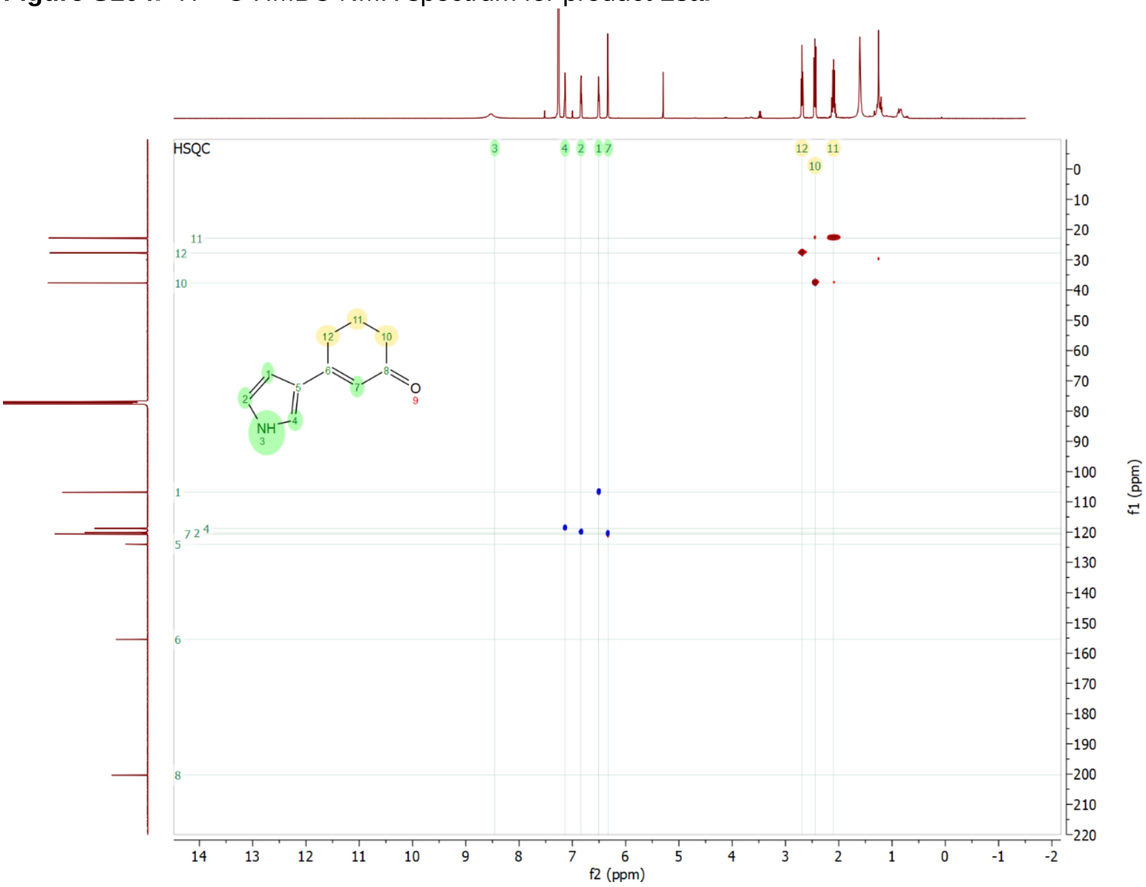

Figure S105. ^{1}H-^{13}C HSQC NMR spectrum for product **13a.**

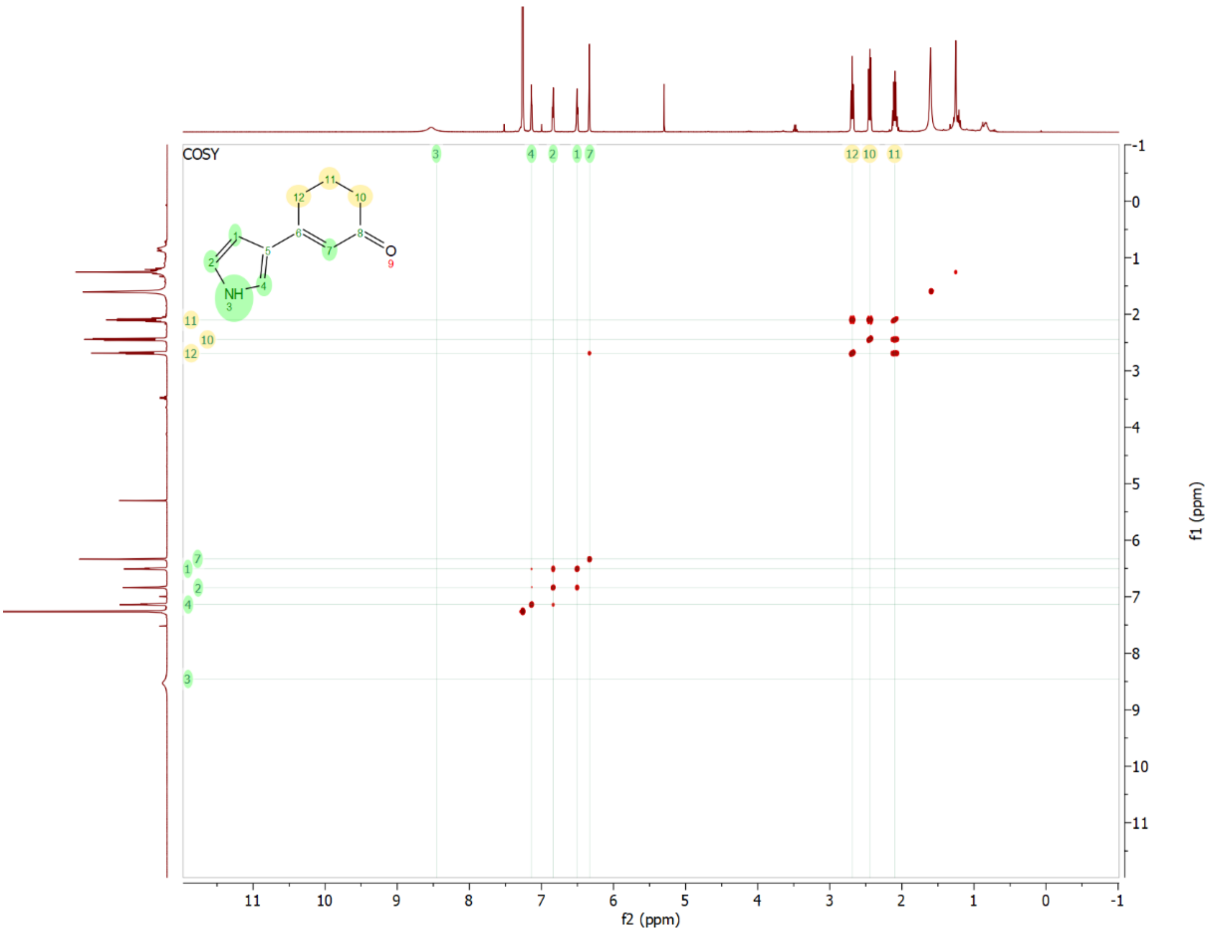

Figure S106. ^1H-^1H COSY NMR spectrum for product **13a.**

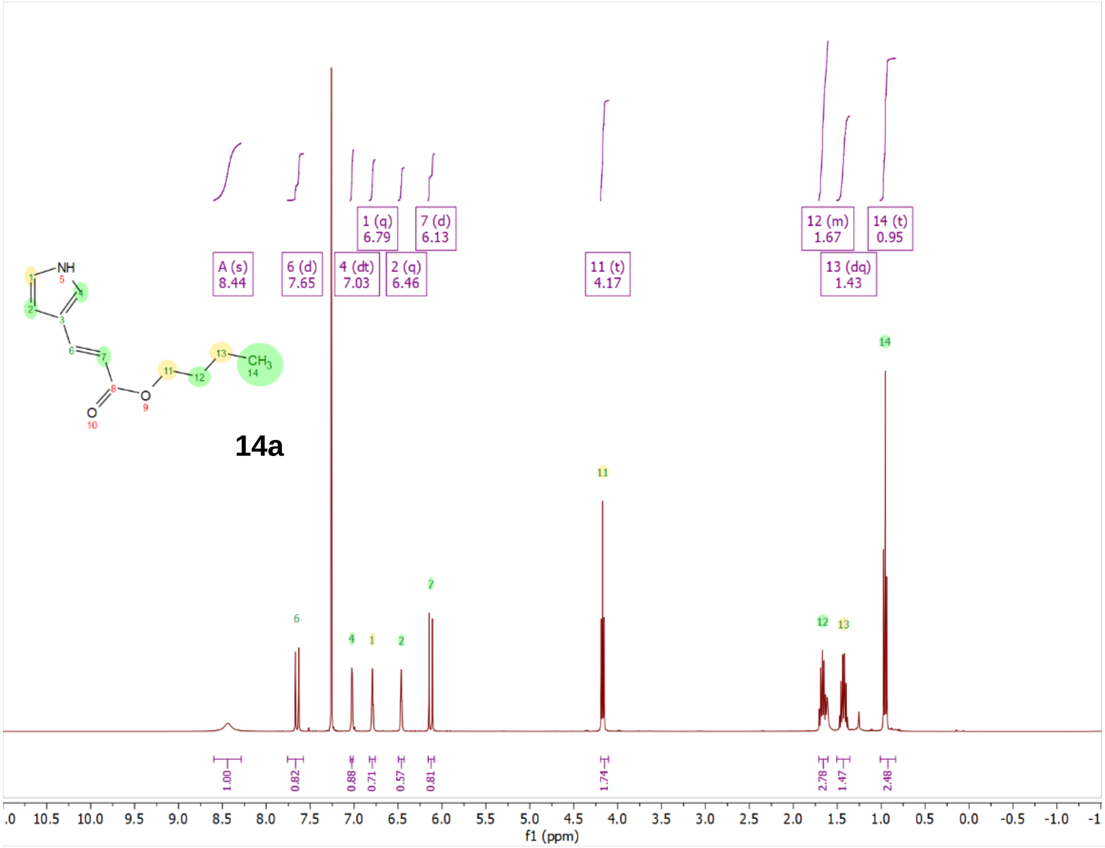

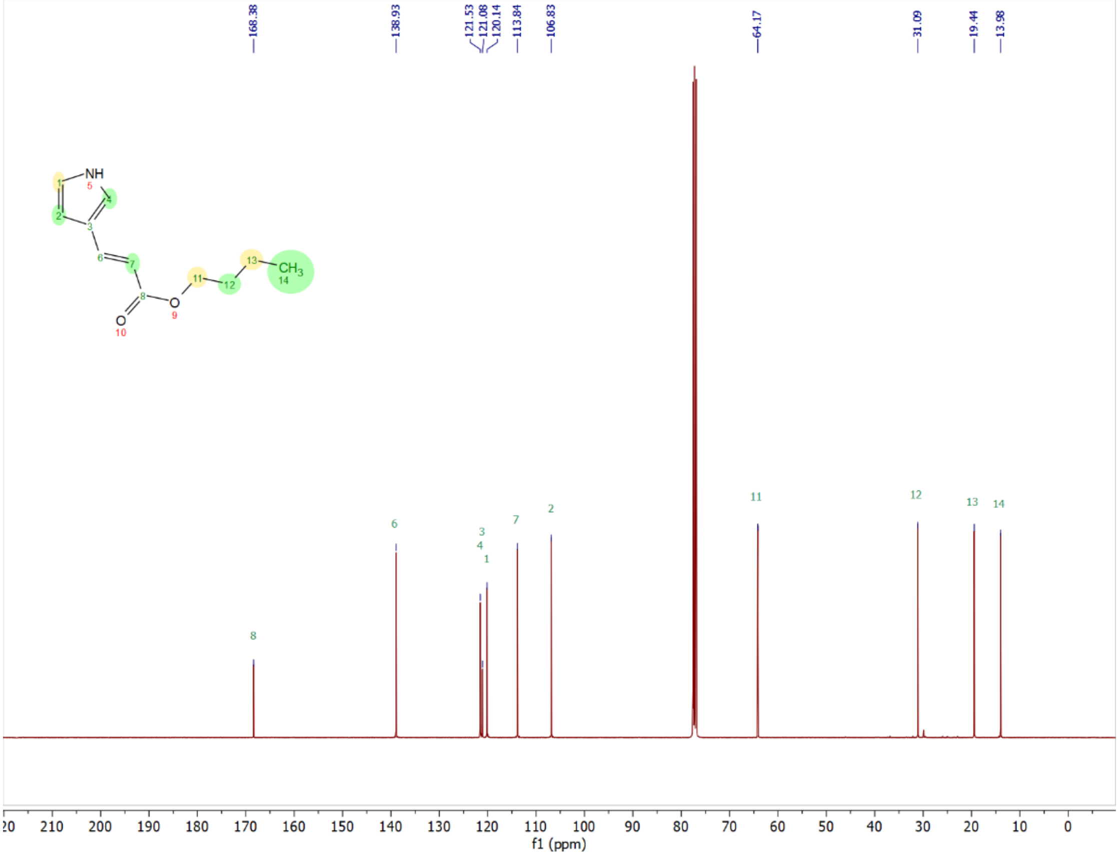

Figure S107. ^{1}H and ^{13}C NMR spectrum for **14a**.

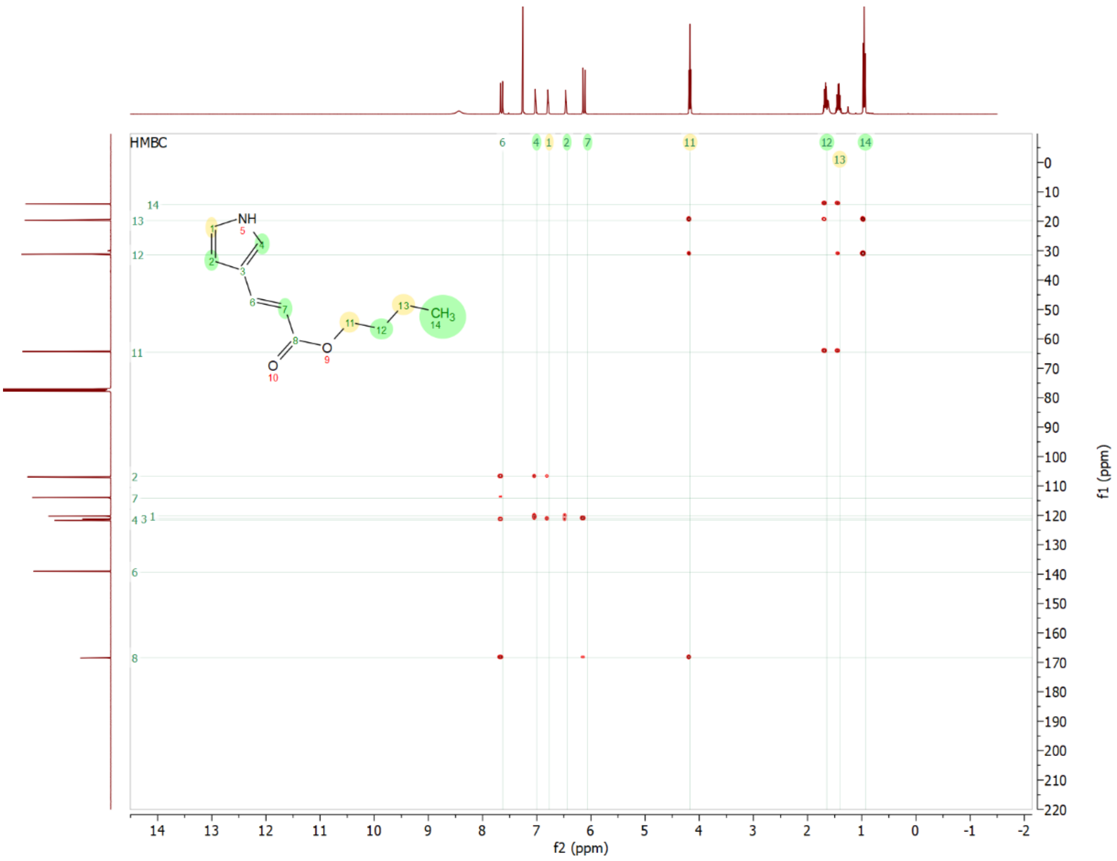

Figure S108. ^1H-^{13}C HMBC NMR spectrum for product **14a.**

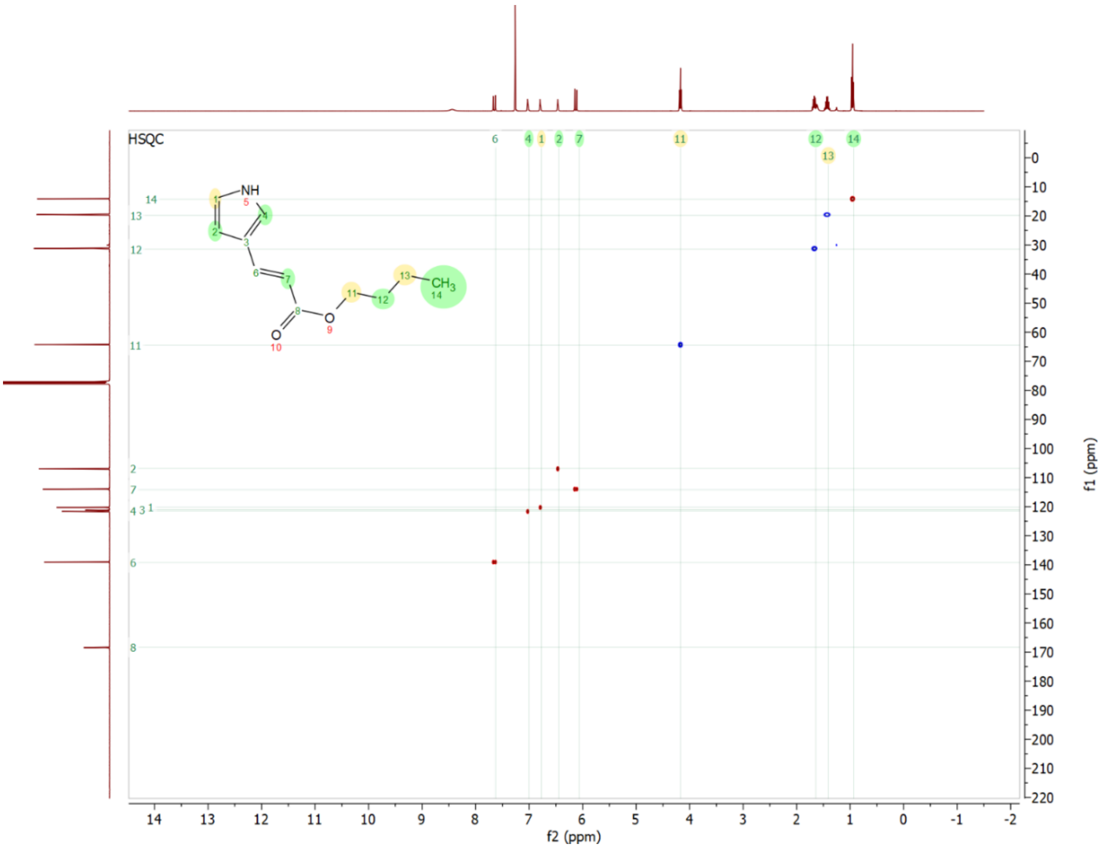

Figure S109. ^1H-^{13}C HSQC NMR spectrum for product **14a.**

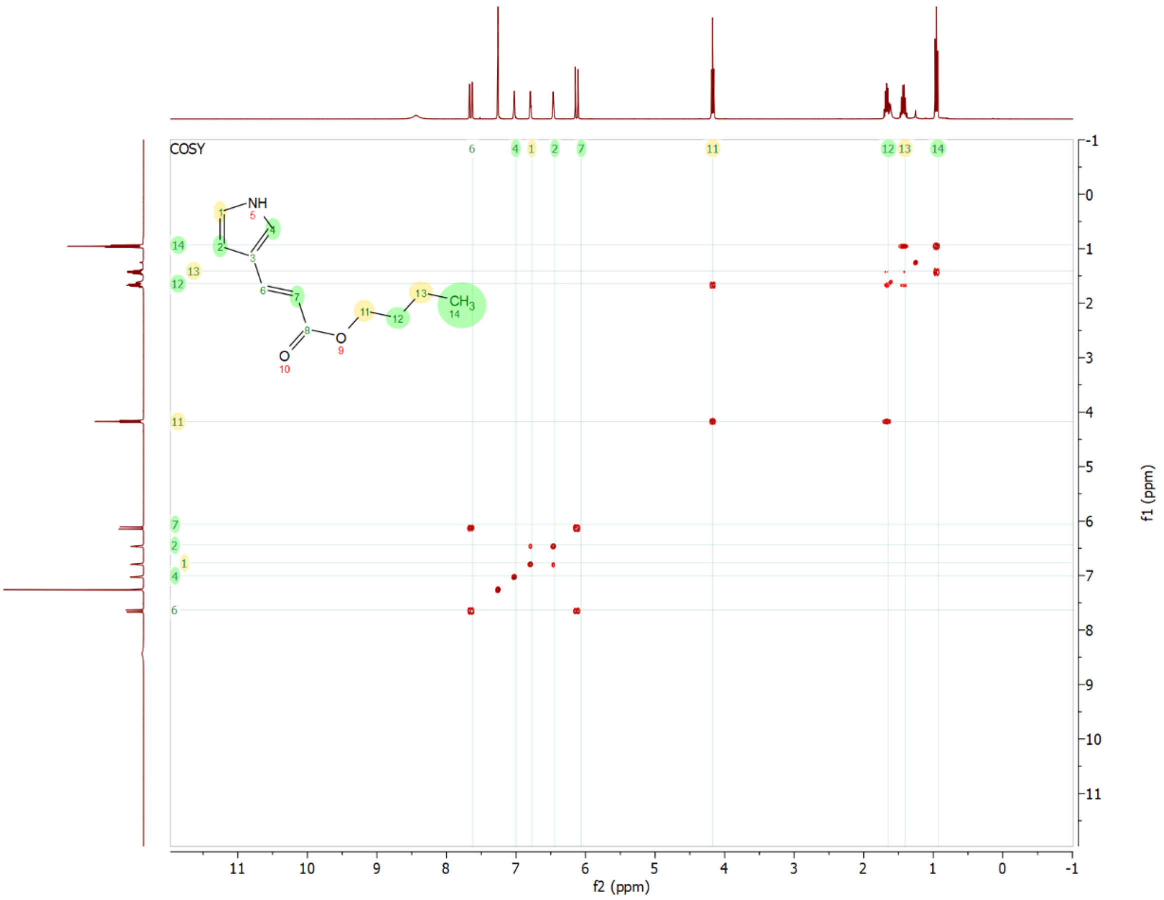

Figure S110. ^{1}H-^{1}H COSY NMR spectrum for product **14a**

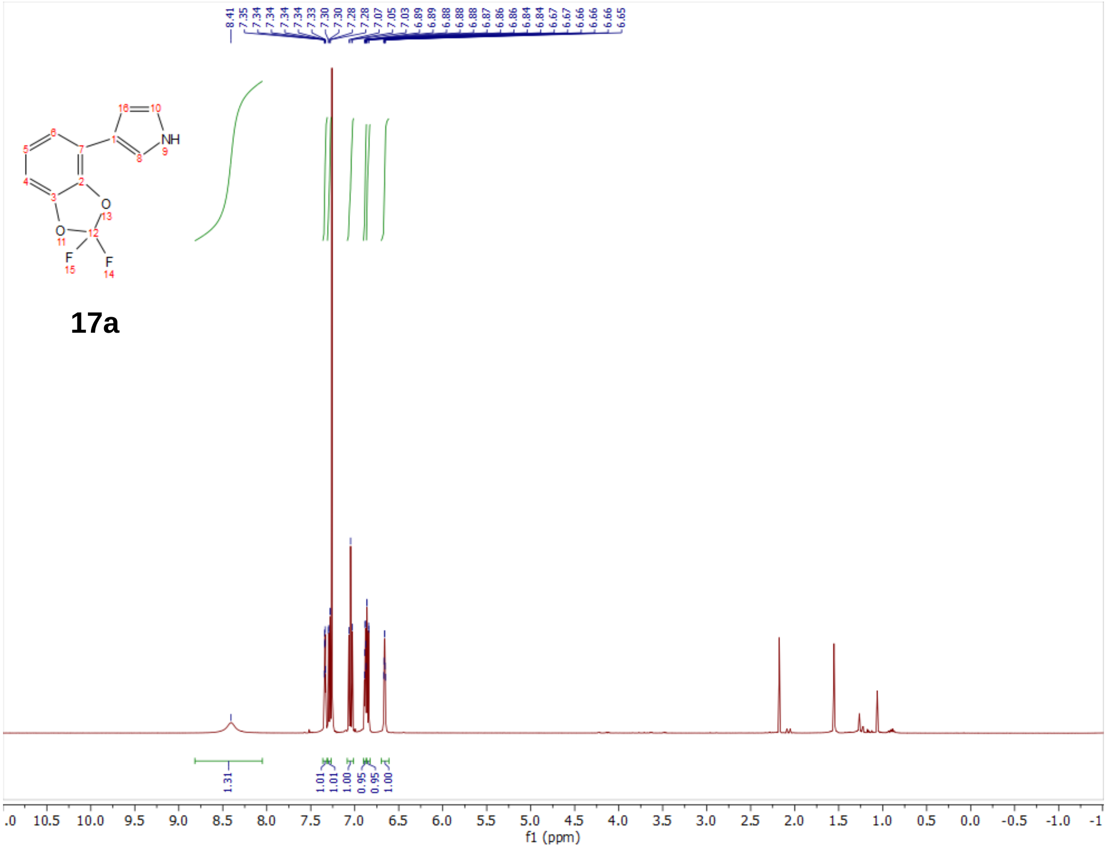

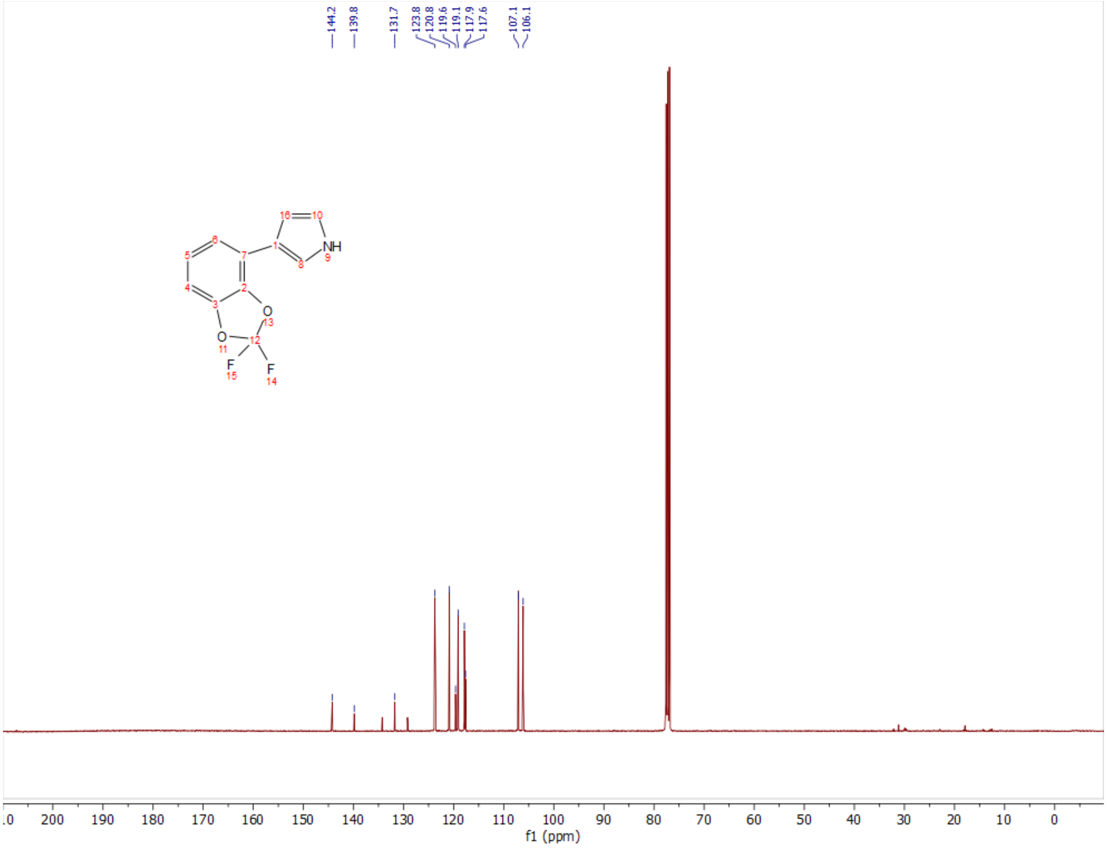

Figure S111. ^{1}H and ^{13}C NMR spectrum for **17a**.

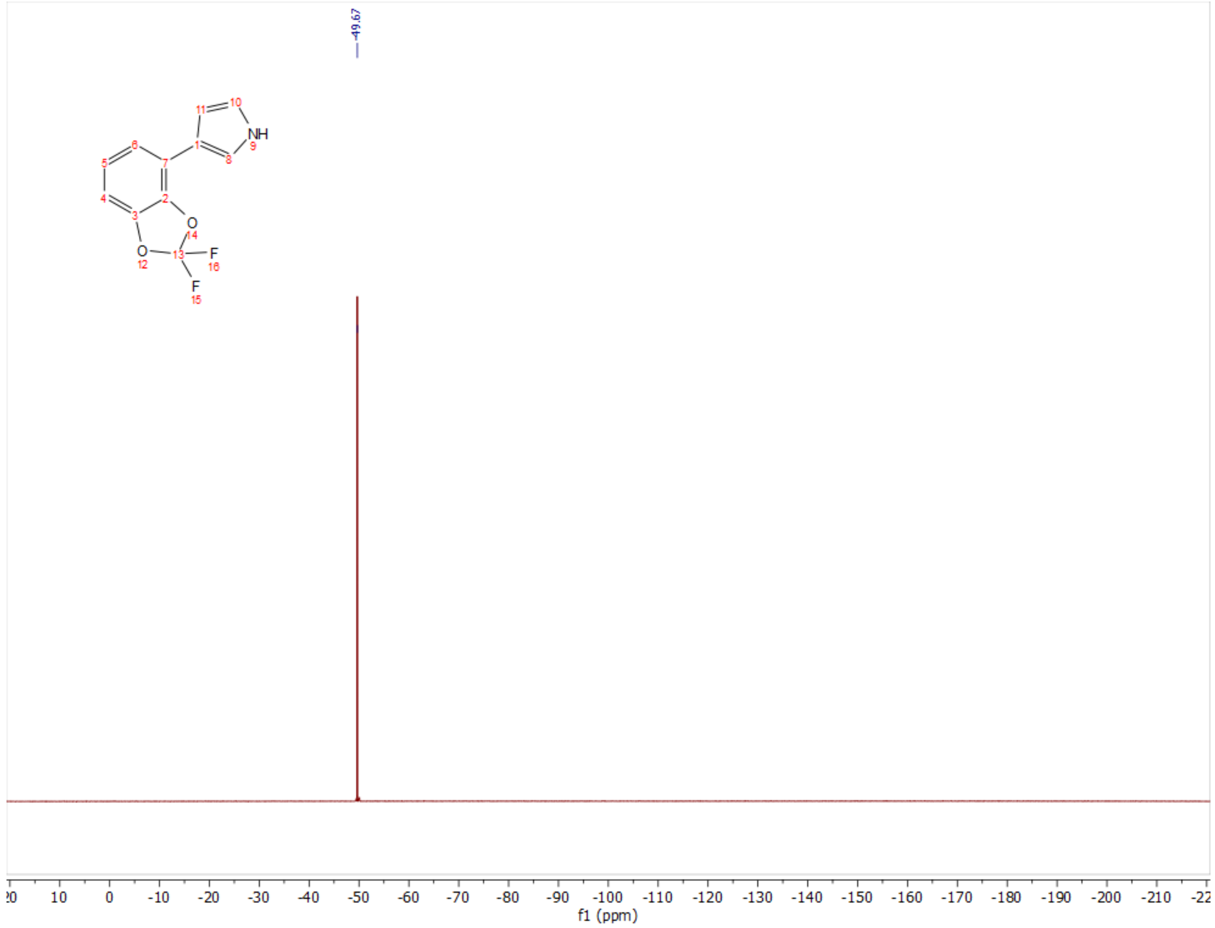

Figure S112. ^{19}F NMR spectrum for **17a**.
