# Supplementary material for: Site-selective chlorination of pyrrolic heterocycles by flavin dependent enzyme PrnC
Source: Commun Chem. 2024 Jan 5;7:7. doi: 10.1038/s42004-023-01083-1 (PMC10770391; doi:10.1038/s42004-023-01083-1)
Supplement: Supplementary file 3 — Description of Additional Supplementary Files [file 42004_2023_1083_MOESM3_ESM.pdf]

Description of Additional Supplementary Files

File name: Supplementary Data 1

Description: ^{1}H, ^{13}C and 2D NMR data spectra of the isolated new compounds.
