# Supplementary material for: Site-selective chlorination of pyrrolic heterocycles by flavin dependent enzyme PrnC
Source: Commun Chem. 2024 Jan 5;7:7. doi: 10.1038/s42004-023-01083-1 (PMC10770391; doi:10.1038/s42004-023-01083-1)
Supplement: Supplementary file 4 — Reporting Summary [file 42004_2023_1083_MOESM4_ESM.pdf]

nature portfolio

Corresponding author(s): Fong Tian Wong, Ee Lui Ang, Yee Hwee Lim

Last updated by author(s): Nov 9, 2023

Reporting Summary

Nature Portfolio wishes to improve the reproducibility of the work that we publish. This form provides structure for consistency and transparency in reporting. For further information on Nature Portfolio policies, see our Editorial Policies and the Editorial Policy Checklist.

Statistics

For all statistical analyses, confirm that the following items are present in the figure legend, table legend, main text, or Methods section.

n/a	Confirmed	
☐	☒	The exact sample size (*n*) for each experimental group/condition, given as a discrete number and unit of measurement
☒	☐	A statement on whether measurements were taken from distinct samples or whether the same sample was measured repeatedly
☒	☐	The statistical test(s) used AND whether they are one- or two-sided<br>*Only common tests should be described solely by name; describe more complex techniques in the Methods section.*
☒	☐	A description of all covariates tested
☒	☐	A description of any assumptions or corrections, such as tests of normality and adjustment for multiple comparisons
☒	☐	A full description of the statistical parameters including central tendency (e.g. means) or other basic estimates (e.g. regression coefficient) AND variation (e.g. standard deviation) or associated estimates of uncertainty (e.g. confidence intervals)
☒	☐	For null hypothesis testing, the test statistic (e.g. *F*, *t*, *r*) with confidence intervals, effect sizes, degrees of freedom and *P* value noted<br>*Give P values as exact values whenever suitable.*
☒	☐	For Bayesian analysis, information on the choice of priors and Markov chain Monte Carlo settings
☒	☐	For hierarchical and complex designs, identification of the appropriate level for tests and full reporting of outcomes
☒	☐	Estimates of effect sizes (e.g. Cohen's *d*, Pearson's *r*), indicating how they were calculated

Our web collection on statistics for biologists contains articles on many of the points above.

Software and code

Policy information about availability of computer code

Data collection	Liquid chromatography mass spectrometry (LC-MS) analysis was conducted using a 1260 Infinity II HPLC system coupled with a G6125B single Quadrupole mass detector operated through Agilent OpenLAB CDS ChemStation (Ver 2.3.5). Cryo-EM data was collected using SerialEM4. High resolution (HRMS) mass spectra were collected using Agilent 6210 Time-of-Flight (Q-TOF). Nuclear magnetic resonance (NMR) data were acquired on either a Bruker AVIII 400 MHz spectrometer with a 13C/1H cryoprobe for 1H and 13C NMR, or a Bruker AV Neo 400 MHz spectrometer with a BBFO cryoprobe for 19F NMR.
Data analysis	Data analysis and visualization were executed using Microsoft Excel (Microsoft Office 365 – version 2108 – build 14326.20238). Raw liquid chromatography mass spectrometry (LC-MS) data was processed using Mestrenova (version 12.0.2-20910). The enzyme kinetics curves were plotted with GraphPad Prism program (GraphPad Software, CA). Modeller v9.10 (UCSF, CA) was used to generate the initial models, Sybyl v2.1.1 (St. Louis, MO) was adopted for the model optimization, and CCDC GOLD v2018 (Cambridge, UK) was used for the molecular docking simulations. Macromolecular structures were visualized and analyzed using CryoSPARC 2 and UCSF Chimera. Chemical structures were drawn using PerkinElmer Chemdraw Pro (version 20.1.1.125).

For manuscripts utilizing custom algorithms or software that are central to the research but not yet described in published literature, software must be made available to editors and reviewers. We strongly encourage code deposition in a community repository (e.g. GitHub). See the Nature Portfolio guidelines for submitting code & software for further information.

Data

Policy information about availability of data

All manuscripts must include a data availability statement. This statement should provide the following information, where applicable:

- Accession codes, unique identifiers, or web links for publicly available datasets
- A description of any restrictions on data availability
- For clinical datasets or third party data, please ensure that the statement adheres to our policy

Data that support the findings of this study are included in this paper. 1D and 2D NMR data for full spectroscopic characterization of synthesized starting substrates and products are provide as 'supplementary data 1'.

Human research participants

Policy information about studies involving human research participants and Sex and Gender in Research.

Reporting on sex and gender	No human research was performed in this study.
Population characteristics	No human research was performed in this study.
Recruitment	No human research was performed in this study.
Ethics oversight	No human research was performed in this study.

Note that full information on the approval of the study protocol must also be provided in the manuscript.

Field-specific reporting

Please select the one below that is the best fit for your research. If you are not sure, read the appropriate sections before making your selection.

☒ Life sciences ☐ Behavioural & social sciences ☐ Ecological, evolutionary & environmental sciences

For a reference copy of the document with all sections, see nature.com/documents/nr-reporting-summary-flat.pdf

Life sciences study design

All studies must disclose on these points even when the disclosure is negative.

Sample size	This chemoenzymatic work pertains primarily to synthetic chemistry. No statistical methods were employed to determine the sample size.
Data exclusions	No data was excluded.
Replication	The screening of substrate scope bioconversion was conducted in duplicate. Enzyme kinetic studies and active site mutagenesis studies were carried out in triplicates. Other optimization assays and control experiments were calculated as the mean average of 2 data points.
Randomization	Randomization is not relevant to this study.
Blinding	Blinding is not relevant to this study.

Reporting for specific materials, systems and methods

We require information from authors about some types of materials, experimental systems and methods used in many studies. Here, indicate whether each material, system or method listed is relevant to your study. If you are not sure if a list item applies to your research, read the appropriate section before selecting a response.

Materials & experimental systems

n/a	Involved in the study
☒	☐ Antibodies
☒	☐ Eukaryotic cell lines
☒	☐ Palaeontology and archaeology
☒	☐ Animals and other organisms
☒	☐ Clinical data
☒	☐ Dual use research of concern

Methods

n/a	Involved in the study
☒	☐ ChIP-seq
☒	☐ Flow cytometry
☒	☐ MRI-based neuroimaging
